# Enantioselective Synthesis of Chiral Cyclopent‐2‐enones by Nickel‐Catalyzed Desymmetrization of Malonate Esters

**DOI:** 10.1002/anie.201805578

**Published:** 2018-06-19

**Authors:** Somnath Narayan Karad, Heena Panchal, Christopher Clarke, William Lewis, Hon Wai Lam

**Affiliations:** ^1^ The GlaxoSmithKline Carbon Neutral Laboratories for Sustainable Chemistry University of Nottingham Jubilee Campus Triumph Road Nottingham NG7 2TU UK; ^2^ School of Chemistry University of Nottingham University Park Nottingham NG7 2RD UK

**Keywords:** asymmetric catalysis, carbocycles, cyclization, isomerization, nickel

## Abstract

The enantioselective synthesis of highly functionalized chiral cyclopent‐2‐enones by the reaction of alkynyl malonate esters with arylboronic acids is described. These desymmetrizing arylative cyclizations are catalyzed by a chiral phosphinooxazoline/nickel complex, and cyclization is enabled by the reversible *E*/*Z* isomerization of alkenylnickel species. The general methodology is also applicable to the synthesis of 1,6‐dihydropyridin‐3(2*H*)‐ones.

Chiral cyclopent‐2‐enones are versatile building blocks for synthesis^1^ and are present in many biologically active natural products^1^ such as (+)‐achalensolide,^2^ phorbol,^3^ and (−)‐kjellmanianone^4^ (Figure 1). In view of their broad significance, various methods have been developed for the de novo construction of enantiomerically enriched chiral cyclopent‐2‐enones,^1^, ^5^, ^6^, ^7^ such as Pauson–Khand reactions,^5^ Nazarov cyclizations,^6^ and several other approaches.^7^ However, given the wide structural diversity of chiral cyclopent‐2‐enones in target compounds, the development of new strategies to these structures continues to be highly valuable.

**Figure 1:**
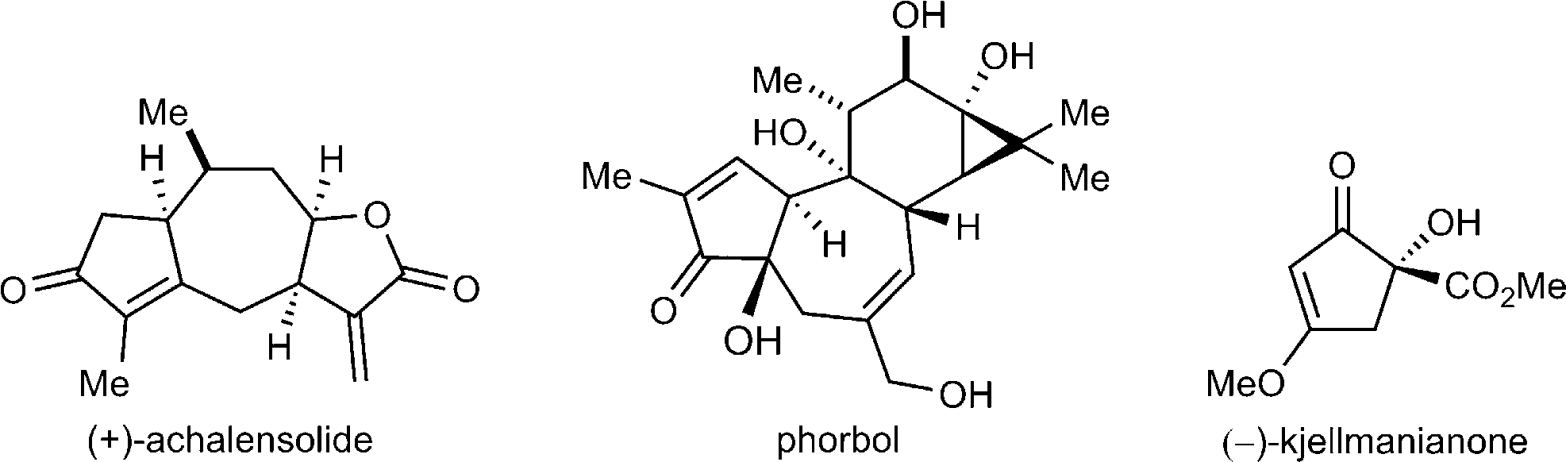
Natural products containing cyclopent‐2‐enones.

We envisaged that chiral cyclopent‐2‐enones might be prepared by the enantioselective nickel‐catalyzed reaction of alkynyl malonate esters (**1**) with arylboronic acids (Scheme 1). Specifically, nickel‐catalyzed *syn* addition of an arylboronic acid to the alkyne of **1** would give the alkenylnickel species (*Z*)‐**2**, which possesses the incorrect stereochemistry for cyclization onto one of the esters. However, reversible *E*/*Z* isomerization^8^, ^9^ of (*Z*)‐**2** would give the alkenylnickel species (*E*)‐**2**, which could now attack an ester in an enantioselective desymmetrization^10^ to give 2,3‐diaryl cyclopent‐2‐enones (**3**).^11^ The 2,3‐diaryl cyclopent‐2‐enone scaffold is present in the highly potent COX‐2 inhibitor **4**,12a as well as in the combretocyclopentenones **5** 12b and related compounds,12c which exhibit antitumor activity. Moreover, there are few asymmetric methods for the de novo construction of cyclopent‐2‐enones with a quaternary stereocenter at the 5‐position (as in **3**).5k, 6g Although our previous work on enantioselective nickel‐catalyzed arylative cyclizations of alkynyl electrophiles showed that ketones8a and activated alkenes8a,8b are competent reaction partners for alkenylnickel species, the ability of less electrophilic esters to undergo analogous cyclizations was less certain. Herein, we report the successful implementation of this strategy. Not only can this methodology produce highly functionalized, enantiomerically enriched chiral cyclopent‐2‐enones, but 1,6‐dihydropyridin‐3(2*H*)‐ones are also accessible.

**Scheme 1:**
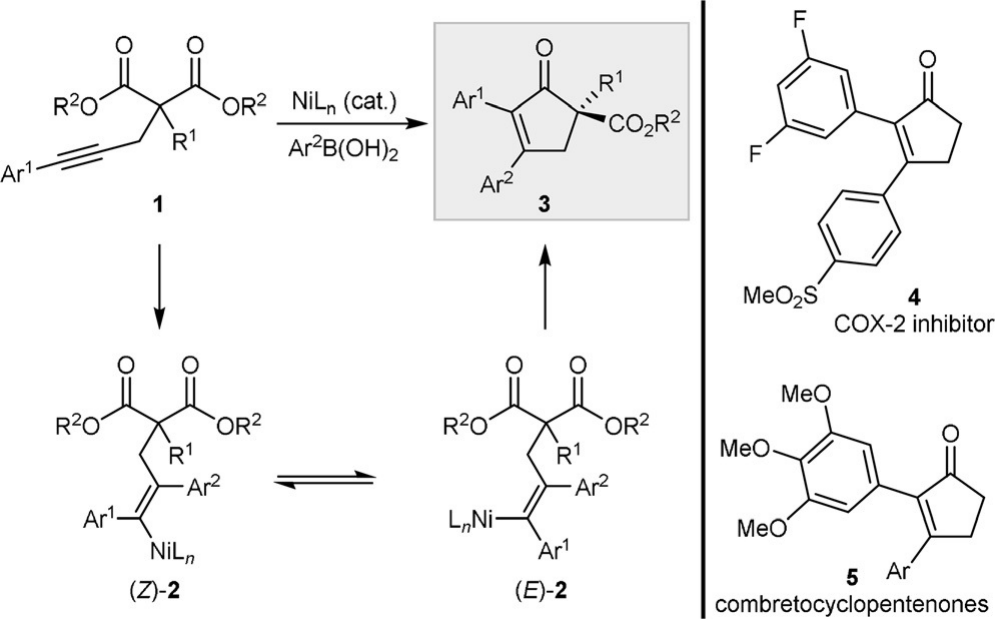
Proposed synthesis of chiral cyclopent‐2‐enones.

Our initial experiments revealed that the substrates **1**, containing ethyl esters, are insufficiently reactive under a range of reaction conditions that are effective in our nickel‐catalyzed *anti*‐carbometallative cyclizations described previously.^8^ However, the more electrophilic bis(2,2,2‐trifluoroethyl) malonate **1 a** reacted successfully with PhB(OH)_2_ (2.0 equiv) in the presence of 10 mol % each of Ni(OAc)_2_⋅4 H_2_O and various chiral P,N‐ligands (**L1–L5**) in 2,2,2‐trifluoroethanol (TFE) to give the cyclopent‐2‐enone **3 aa** (Table 1). At 100 °C, (*R*)‐Ph‐PHOX (**L1**) gave **3 aa** in 98 % yield (by ^1^H NMR analysis) and 91 % *ee* (entry 1).^13^ Reducing the temperature to 80 °C improved the enantioselectivity to 94 % *ee* with no loss of yield (entry 2). Other phosphinooxazolines, **L2**–**L5**, are effective at 80 °C (entries 3–5), but with the exception of **L3** (entry 4), the yields and enantioselectivities are appreciably lower than with **L1**.

**Table 1:** Evaluation of reaction conditions.^[a]^ 
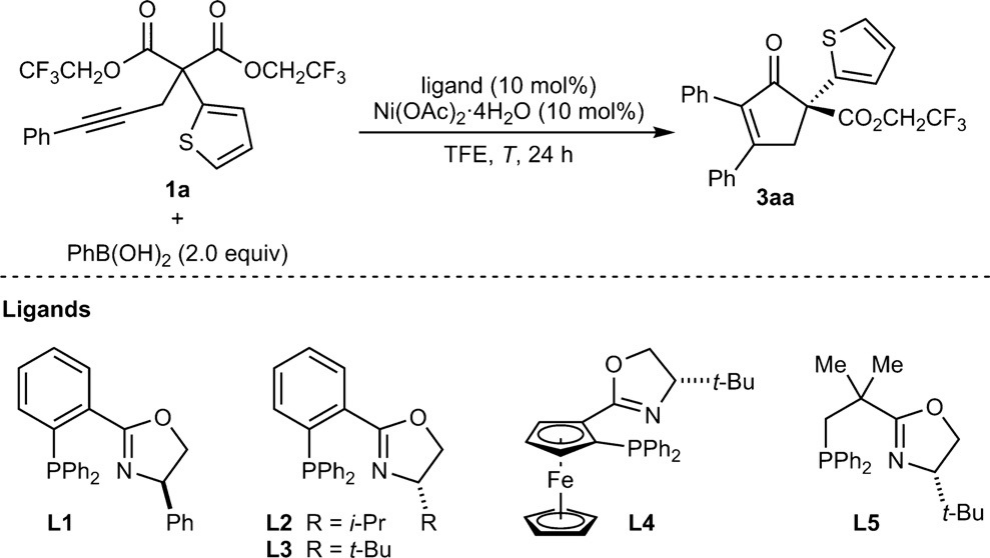

Entry	Ligand	*T* [°C]	Yield [%]^[b]^	*ee* [%]^[c]^
1	**L1**	100	98	91
2	**L1**	80	99	94
3	**L2**	80	94	−81^[d]^
4	**L3**	80	84	−94^[d]^
5	**L4**	80	49	88
6	**L5**	80	61	−78^[d]^

[a] Reactions were conducted with 0.05 mmol of **1 a** in TFE (0.5 mL). [b] Determined by ^1^H NMR analysis using 1,4‐dimethoxybenzene as an internal standard. [c] Determined by HPLC analysis on a chiral stationary phase. [d] These reactions gave *ent*‐**3 aa** as the major enantiomer.

The scope of this process with respect to the alkynyl bis(2,2,2‐trifluoroethyl) malonate was then explored using **L1** as the chiral ligand in reactions with PhB(OH)_2_, which gave cyclopent‐2‐enones (**3 aa**–**pa**) in 46–98 % yield and 77–94 % *ee* (Scheme 2). As well as a 2‐thienyl group (**3 aa**, **3 ia**, and **3 ja**), the substituent at the 2‐position of **1** can be changed to a phenyl group (**3 ba**), mono‐ and disubstituted benzenes with electron‐donating or electron‐withdrawing substituents (**3 ca**–**ga**, **3 ka**, and **3 la**), and a 2‐naphthyl group (**3 ha**). Ethoxy (**3 ma**), benzyloxy (**3 na**), 3‐thienylmethoxy (**3 oa**), and anilino groups (**3 pa**) at this position are also tolerated. The reaction is compatible with various other (hetero)aryl groups at the alkyne, such as 4‐methoxyphenyl (**3 ia**), 3‐methylphenyl (**3 ja**), 4‐chlorophenyl (**3 ka**), and 2‐thienyl (**3 la**). In a few cases, reaction at 100 °C (**3 ca**, **3 ka**, and **3 la**) or use of a 20 mol % catalyst loading (**3 ma** and **3 oa**) were required for complete consumption of the starting material.

**Scheme 2:**
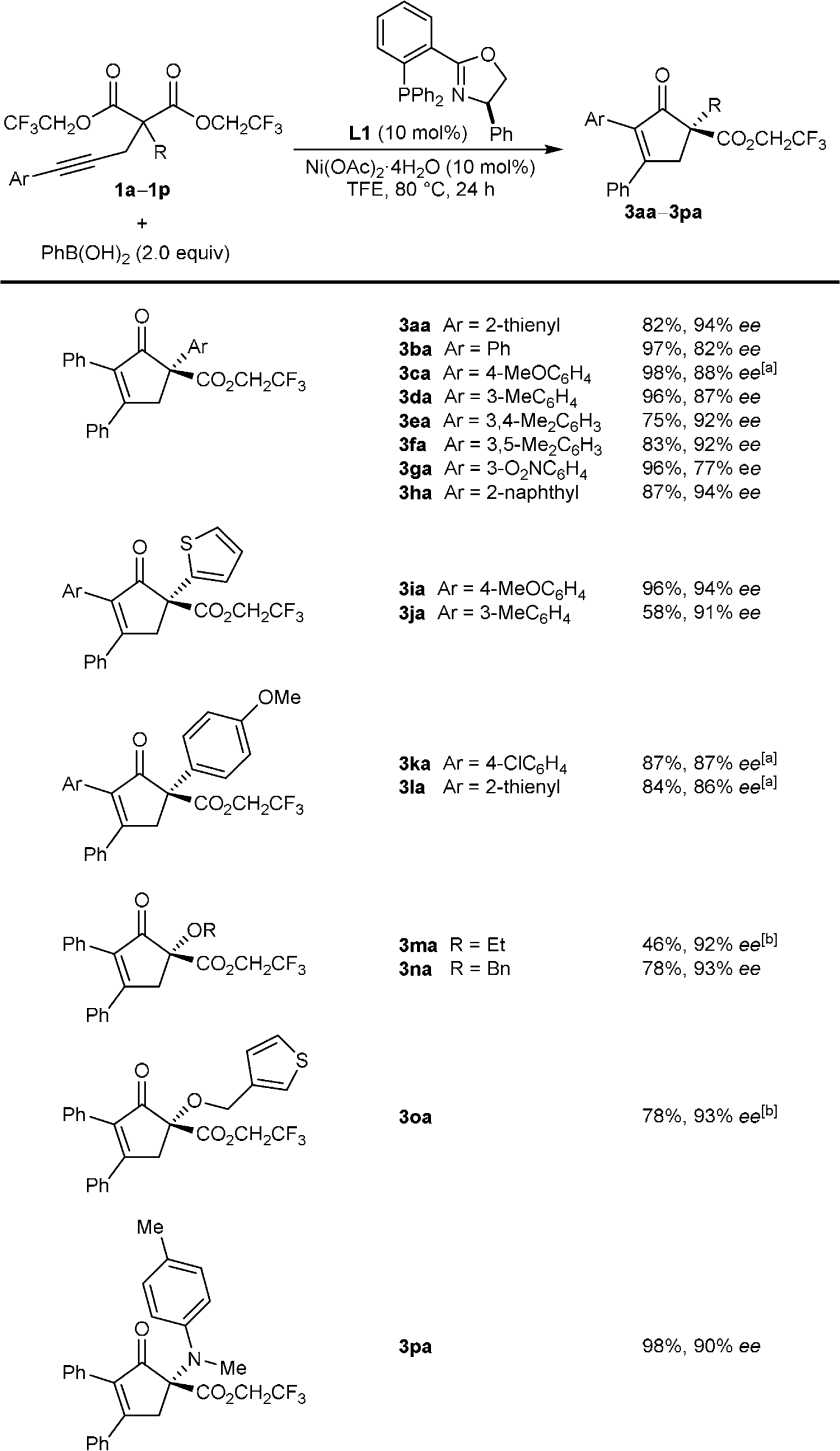
Scope with respect to the alkynyl bis(2,2,2‐trifluoroethyl) malonate. Reactions were conducted with 0.30 mmol of **1 a**–**p** in TFE (3 mL). Yields are those of the isolated products. Enantiomeric excesses were determined by HPLC analysis on a chiral stationary phase. [a] Conducted at 100 °C. [b] Conducted with 20 mol % each of Ni(OAc)_2_⋅4 H_2_O and **L1**.

The process is not limited to aryl groups at the alkyne, as shown by the reaction of the 1,3‐enyne **6** to give the cyclopent‐2‐enone **7** in 76 % yield and 80 % *ee* [Eq. 1]. (*R*)‐Ph‐PHOX (**L1**) is less effective for substrates with alkyl groups at the 2‐position. For example, the cyclization of **1 q** and **1 r** (see [Eqs. (2) and (3) for the structures] gave cyclopent‐2‐enones in 29 and 0 % *ee*, respectively, with **L1** as the ligand. However, somewhat improved results were obtained with (*S*)‐*t*‐Bu‐NeoPHOX (**L5**),^14^ which gave *ent*‐**3 qa** and *ent*‐**3 ra** in 59 and 54 % *ee*, respectively [Eqs. (2) and (3)].
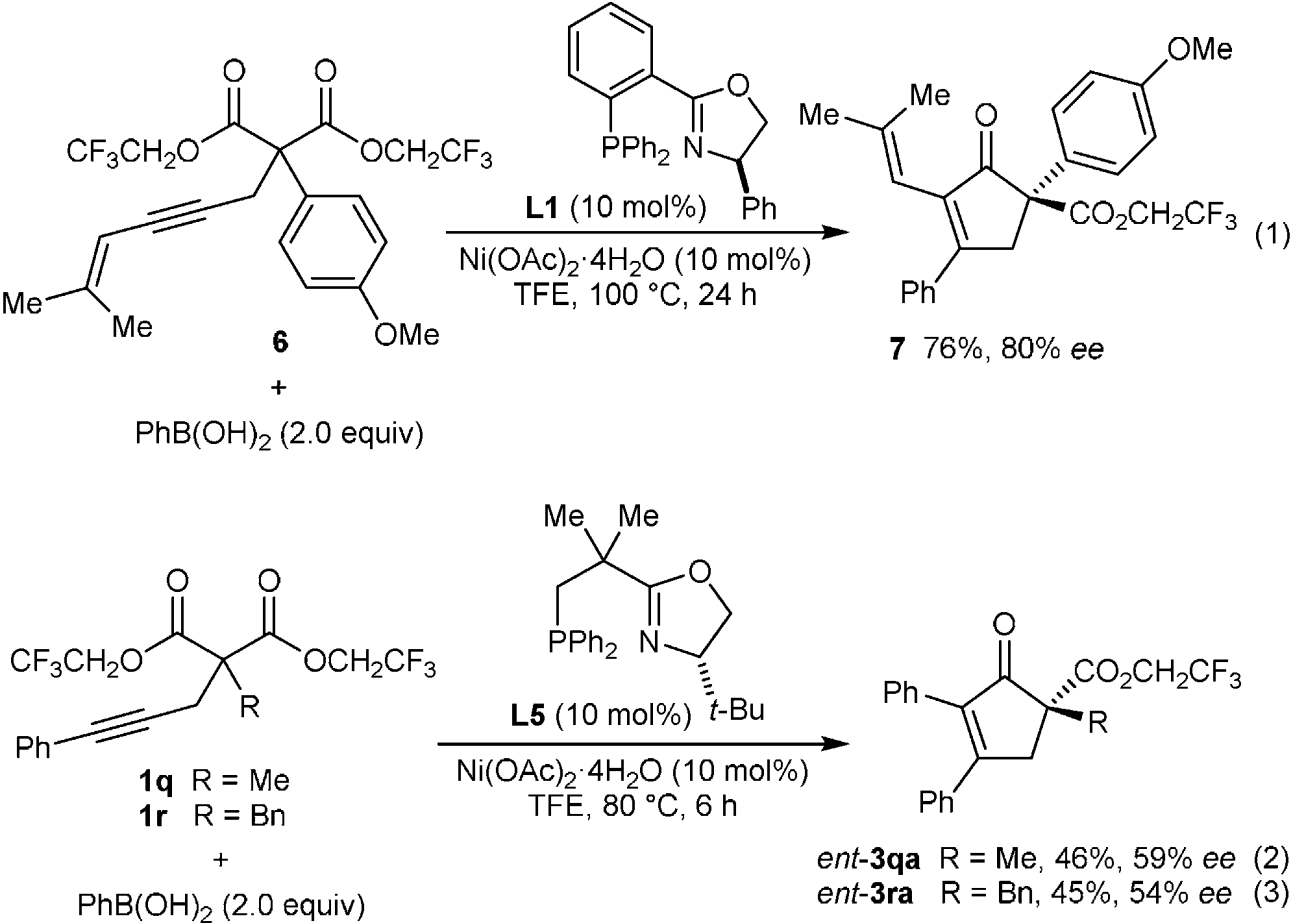



The reactions of a range of (hetero)arylboronic acids with representative substrates, **1 a**, **1 c**, **1 i**, and **1 n**, are presented in Scheme 3. Pleasingly, these reactions gave cyclopent‐2‐enones in generally good yields (73–92 %) and enantioselectivities (80–94 % *ee*). The process is compatible with arylboronic acids containing halide (**3 ab**, **3 cg**, **3 ij**, **3 ik**, and **3 nm**), methyl (**3 ac**, **3 ch** and **3 nm**), carboethoxy (**3 ad**), or alkoxy (**3 cf**, **3 ij**, and **3 nl**) substituents. 2‐Naphthylboronic acid (**3 ii**) and 3‐thienylboronic acid (**3 ae**) are also effective.

**Scheme 3:**
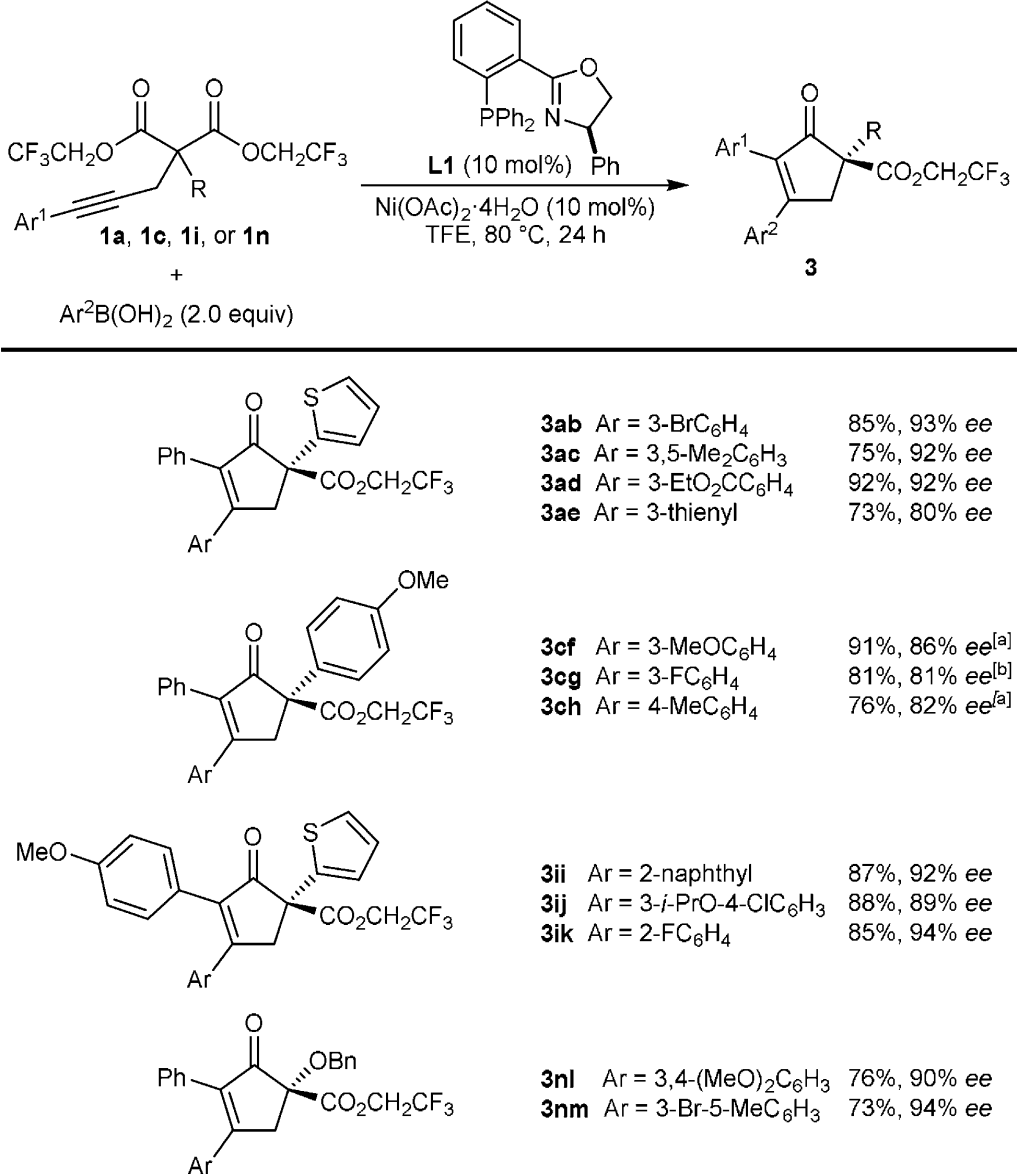
Scope with respect to the boronic acid. Reactions were conducted with 0.30 mmol of **1 a**, **1 c**, **1 i**, or **1 n** in TFE (3 mL). Yields are those of the isolated products. Enantiomeric excesses were determined by HPLC analysis on a chiral stationary phase. [a] Conducted at 100 °C. [b] Conducted at 120 °C.

The process also works well for gram‐scale reactions. For example, the reaction of **1 b** (1.38 g, 3.00 mmol) with PhB(OH)_2_ gave 1.10 grams of **3 ba** (84 % yield) in 80 % *ee* [Eq. 4]. Importantly, by conducting this reaction a higher concentration of 0.4 m, rather than at 0.1 m used in the experiments shown in Scheme 2 and Scheme 3, the catalyst loading was lowered to 3 mol %.
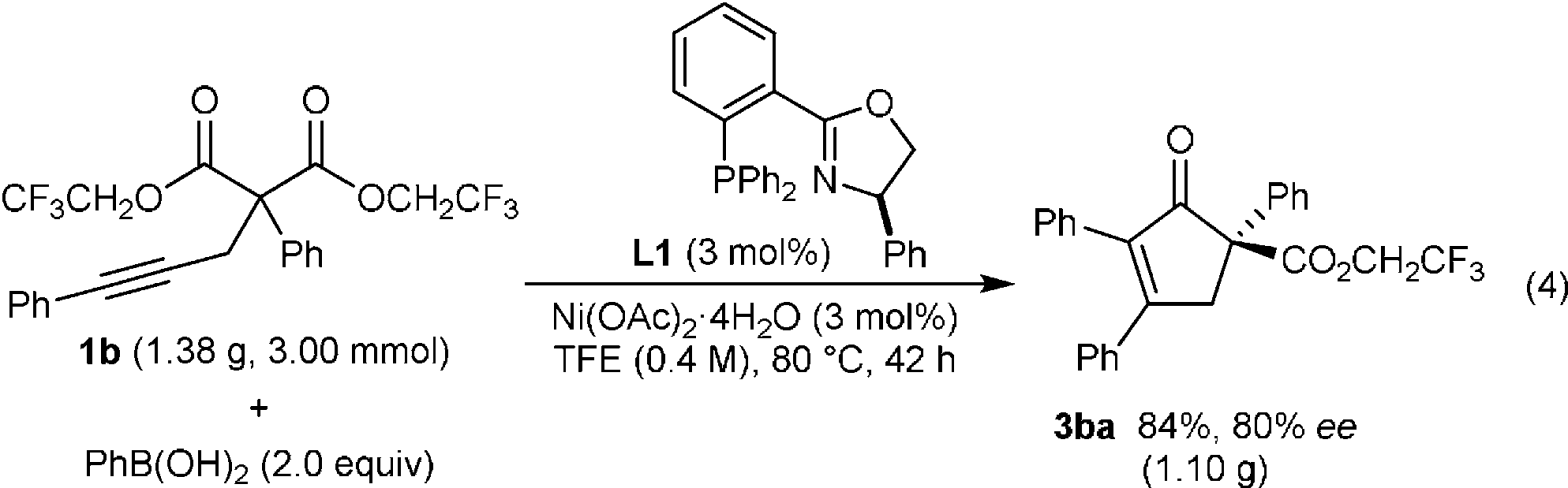



To demonstrate the synthetic utility of the products, further transformations of a representative cyclopent‐2‐enone were conducted. Trifluoroethyl esters are moderately active acylating agents^15^ and could therefore serve as useful functional handles. Indeed, heating **3 ik** with benzylamine (1.5 equiv) in THF at 90 °C smoothly gave the amide **8** in 84 % yield without affecting the enone (Scheme 4). A Luche reduction of **8** then gave allylic alcohol **9** as a single observable diastereomer in 83 % yield.

**Scheme 4:**
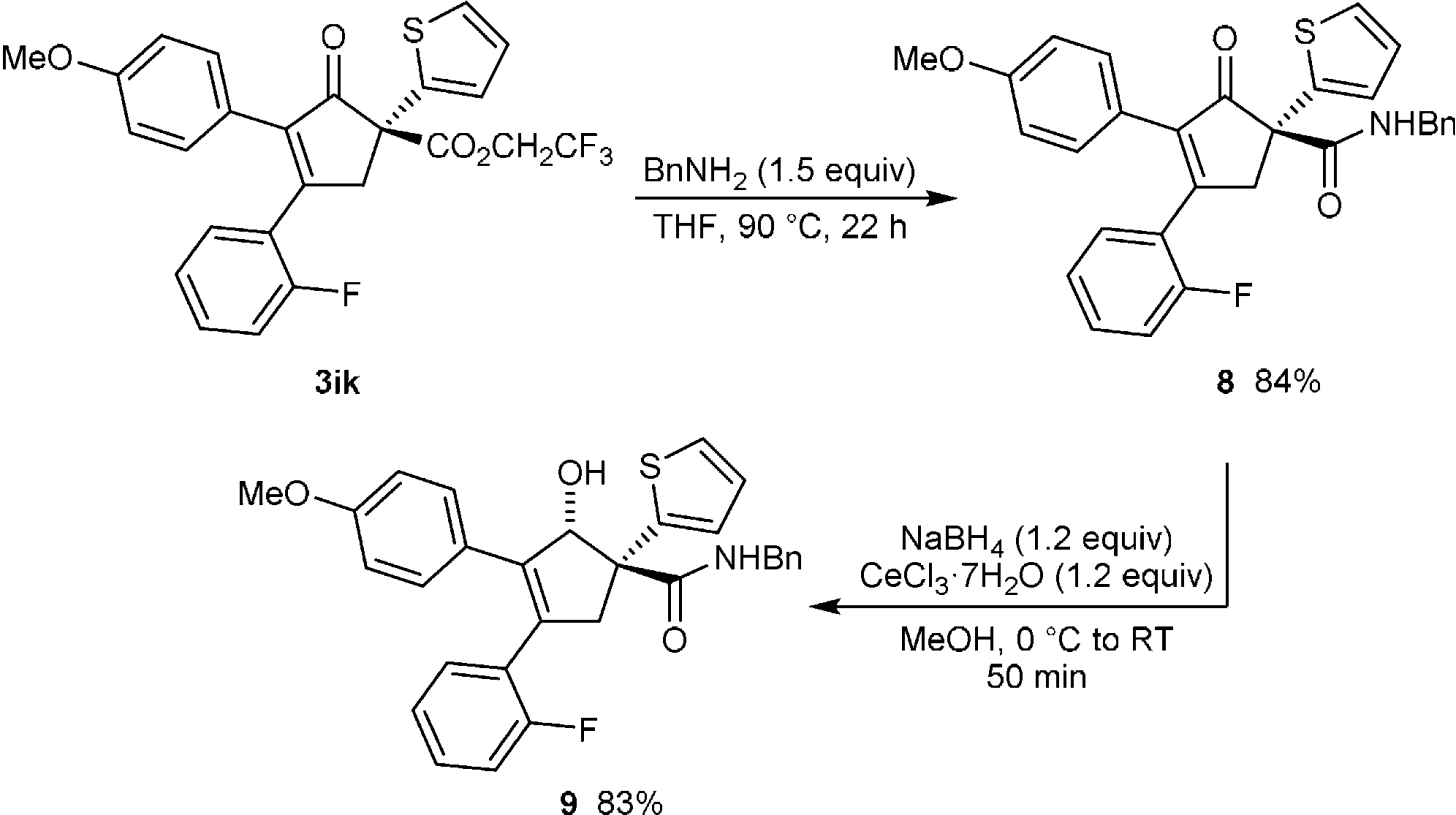
Further transformations of the cyclopent‐2‐enone **3 ik**.

Finally, although chiral cyclopent‐2‐enones were the primary targets of this study, the general methodology can be applied to the synthesis of other products. For example, reaction of the alkynyl phenyl ester **10** with PhB(OH)_2_ using (*S*)‐*i*‐Pr‐NeoPHOX (**L6**)^14^ as the ligand gave a 27:1 mixture of the 1,6‐dihydropyridin‐3(2*H*)‐one **11** together with a minor product (**12**) in 68 % yield [Eq. 5].^16^ Other P,N‐ligands resulted in lower yields and less favorable ratios of **11**:**12**.
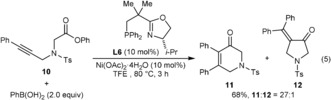



In conclusion, we have reported the enantioselective synthesis of chiral cyclopent‐2‐enones by the nickel‐catalyzed desymmetrizing arylative cyclization of alkynyl bis(2,2,2‐trifluoroethyl) malonates with arylboronic acids. The reactions proceed in good yields and generally high enantioselectivities to give cyclopent‐2‐enones containing a fully substituted alkene and a quaternary stereocenter at the 5‐position. This work further demonstrates the utility of reversible *E*/*Z* isomerization of alkenylnickel species in promoting new domino addition/cyclizations of alkynyl electrophiles, reactions that would otherwise be impossible.^8^, 9d,9e Investigation of this reactivity in other contexts is ongoing and will be reported in due course.

## Conflict of interest

The authors declare no conflict of interest.

## Supporting information

As a service to our authors and readers, this journal provides supporting information supplied by the authors. Such materials are peer reviewed and may be re‐organized for online delivery, but are not copy‐edited or typeset. Technical support issues arising from supporting information (other than missing files) should be addressed to the authors.

Supplementary
