# Supplementary material for: Enantioselective Synthesis of Chiral Cyclopent‐2‐enones by Nickel‐Catalyzed Desymmetrization of Malonate Esters
Source: Angew Chem Int Ed Engl. 2018 Jun 19;57(29):9122–5. doi: 10.1002/anie.201805578 (PMC6485403; doi:10.1002/anie.201805578)
Supplement: Supplementary file 1 — Supplementary [file ANIE-57-9122-s001.pdf]

Angewandte Chemie
Eine Zeitschrift der Gesellschaft Deutscher Chemiker

Supporting Information

Enantioselective Synthesis of Chiral Cyclopent-2-enones by Nickel-Catalyzed Desymmetrization of Malonate Esters

*Somnath Narayan Karad, Heena Panchal, Christopher Clarke, William Lewis, and Hon Wai Lam**

anie_201805578_sm_miscellaneous_information.pdf

Author Contributions

S.K. Conceptualization: Equal; Data curation: Lead; Formal analysis: Lead; Investigation: Lead; Methodology: Lead; Validation: Lead; Writing—review & editing: Supporting
H.P. Data curation: Supporting; Formal analysis: Supporting; Investigation: Supporting; Methodology: Supporting; Validation: Supporting; Writing—review & editing: Supporting
C.C. Data curation: Supporting; Formal analysis: Supporting; Investigation: Supporting; Methodology: Supporting; Validation: Supporting; Writing—review & editing: Supporting
W.L. Data curation: Supporting; Formal analysis: Supporting; Validation: Supporting
H.L. Conceptualization: Equal; Formal analysis: Supporting; Funding acquisition: Lead; Supervision: Lead; Validation: Supporting; Writing—original draft: Lead; Writing—review & editing: Lead.

Supporting Information

1. General Information 2
2. Preparation of Substrates 4
3. Enantioselective Nickel-Catalyzed Desymmetrization of Malonate Esters 24
4. Further Transformations of Cyclopent-2-enone **3ik** 43
5. Nickel-Catalyzed Arylative Cyclization of Substrate **9** 46
6. NMR Spectra 47
7. HPLC Traces 115
8. References 146

1. General Information

All air-sensitive reactions were carried out under an inert atmosphere using oven-dried apparatus. 2,2,2-trifluoroethanol (TFE) was purchased from Alfa Aesar and degassed before use using the freeze-pump-thaw technique (5 × cycles). All commercially available reagents were used as received unless otherwise stated. Petroleum ether refers to Sigma-Aldrich product 24587 (petroleum ether boiling point 40-60 °C). Thin layer chromatography (TLC) was performed on Merck DF-Alufoilien 60F254 0.2 mm precoated plates. Compounds were visualized by exposure to UV light or by dipping the plates into solutions of potassium permanganate or vanillin followed by gentle heating. Flash column chromatography was carried out using silica gel (Fisher Scientific 60 Å particle size 35-70 micron or Fluorochem 60 Å particle size 40-63 micron). Melting points were recorded on a Gallenkamp melting point apparatus and are uncorrected. The solvent of recrystallization is reported in parentheses. Infrared (IR) spectra were recorded on Bruker platinum alpha FTIR spectrometer on the neat compound using the attenuated total refraction technique. NMR spectra were acquired on Bruker AV500, Bruker AV500(III)HD, Bruker AV400, Bruker AV(III)400HD, or Bruker DPX400 spectrometers. ^{1}H and ^{13}C NMR spectra were referenced to external tetramethylsilane via the residual protonated solvent (^{1}H) or the solvent itself (^{13}C). All chemical shifts are reported in parts per million (ppm). For $CDCl_3$, the shifts are referenced to 7.26 ppm for ^{1}H NMR spectroscopy and 77.16 ppm for ^{13}C NMR spectroscopy. Coupling constants (*J*) are quoted to the nearest 0.1 Hz. Assignments were made using the DEPT sequence with secondary pulses at 90° and 135°. High-resolution mass spectra were recorded using electrospray ionization (ESI) or gas chromatography mass spectrometry (GC/MS) techniques. X-ray diffraction data were collected at 120 K on an Agilent SuperNova diffractometer using CuKα radiation. Chiral HPLC analysis was performed on an Agilent 1290 series instrument using 4.6 × 250 mm columns. 2-[2-(Diphenylphosphino)ethyl]pyridine (Sigma-Aldrich product 695599) was used as an achiral ligand to obtain authentic racemic compounds. Ligand (**L5**) was prepared according to the literature procedure.[1]

2. Preparation of Substrates

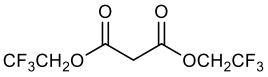

S1

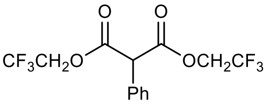

S2

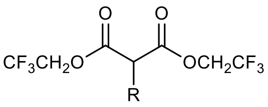

S3a R = Me
S3b R = Bn

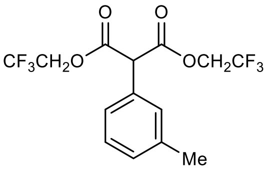

S4

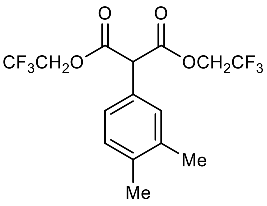

S5

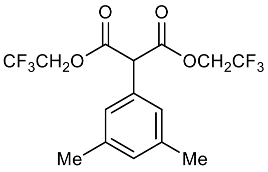

S6

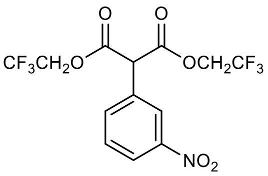

S7

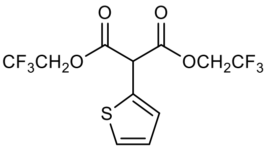

S8

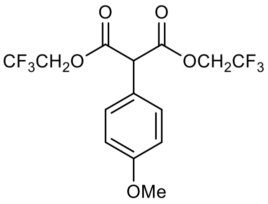

S9

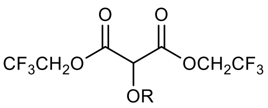

S10a R = Et
S10b R = Bn

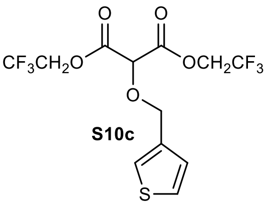

S10c

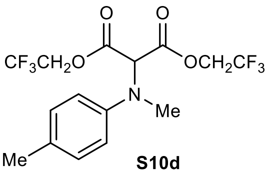

S10d

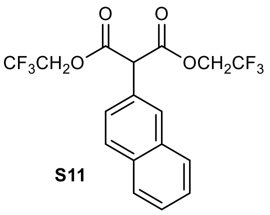

S11

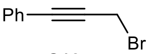

S12

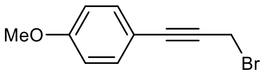

S13

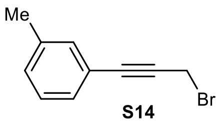

S14

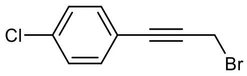

S15

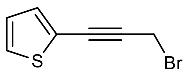

S16

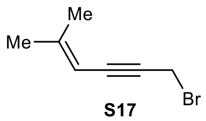

S17

The previously reported procedure for the preparation of **S1**[2] was modified for the synthesis of **S2**, **S3a**, **S3b**, and **S4** (General Procedure A).[2] The substituted bis(2,2,2-trifluoroethyl) malonates **S7** and **S8** were prepared by modification of a previously reported method (General Procedure B).[3,4] Alkynyl bromides **S12**,[5] **S13**,[6] **S14**,[6] **S15**,[5] and **S16**,[7] were prepared by reported procedures. The procedures for the preparation of **S5**, **S6**, **S9-S11** and **S17** are described in detail in the following sections.

General Procedure A[2]: H_2SO_4-Catalyzed Esterification of α-Substituted Malonic Acids

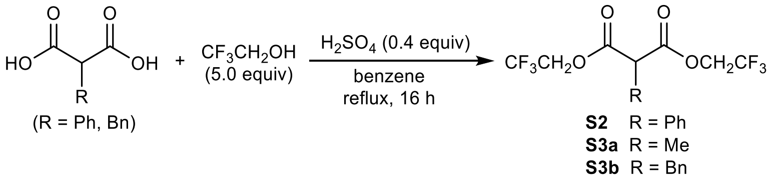

A round bottom flask equipped with a reflux condenser was charged with the appropriate substituted malonic acid (1 equiv), TFE (5 equiv), concentrated H_2SO_4 (0.4 equiv), benzene (1.1 M) and a stirrer bar. The mixture was then heated at reflux overnight. The solution was cooled to room temperature and diluted with benzene (2 mL per mmol of malonic acid). The solution was then washed with saturated aqueous $NaHCO_3$ solution (3 × organic volume), H_2O (1 × organic volume) and brine (1 × organic volume). The organic layer was then dried ($MgSO_4$), filtered and concentrated under reduced pressure to give the title compound (**S2**/**S3a**/**S3b**).

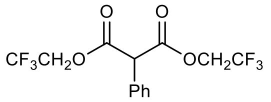

Bis(2,2,2-trifluoroethyl) 2-phenylmalonate (S2). The title compound was prepared according to General Procedure A, using phenylmalonic acid (4.00 g, 22.2 mmol), TFE (8.10 mL, 111.0 mmol), concentrated H_2SO_4 (0.47 mL, 8.8 mmol), and benzene (20 mL) to give a colorless oil (2.56 g, 35%). R_f = 0.20 (5% EtOAc/petroleum ether); IR 2980, 1755 (C=O), 1410, 1275, 1158, 1129, 1056, 979, 727, 547 cm^{-1}; ^{1}H NMR (400 MHz, $CDCl_3$) δ 7.40 (5H, br s, Ar**H**), 4.83 (1H, s, C**H**Ph), 4.61-4.47 (4H, m, 2 × C**H**$_2$CF$_3$); ^{13}C NMR (101 MHz, $CDCl_3$) δ 166.0 (2 × C), 130.8 (C), 129.3 (2 × CH), 129.20 (CH), 129.15 (2 × CH), 122.6 (q, $J_{C\text{-}F}$ = 277.2 Hz, 2 × C), 61.5 (q, $J_{C\text{-}F}$ = 37.3 Hz, 2 × CH_2), 56.8 (CH); ^{19}F NMR (376 MHz, $CDCl_3$) δ −73.8 (t, J = 8.2 Hz, 6 × F); HRMS (ESI) exact mass calculated for $[C_{13}H_{10}F_6NaO_4]^+$ $[M+Na]^+$: 367.0375, found: 367.0362.

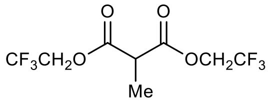

Bis(2,2,2-trifluoroethyl) 2-methylmalonate (S3a). The title compound was prepared according to General Procedure A, using methylmalonic acid (4.00 g, 33.9 mmol), TFE (12.19 mL, 169.4 mmol), concentrated H_2SO_4 (0.72 mL, 13.5 mmol), and benzene (20 mL) to give a colorless oil (2.48 g, 29%). R_f = 0.20 (4% EtOAc/petroleum ether). IR 2982, 1756 (C=O), 1413, 1277, 1158, 1087, 973, 841, 650, 552 cm^{-1}; ^{1}H NMR (400 MHz, $CDCl_3$) 4.59-4.46 (4H, m, 2 × C**H**$_2$CF$_3$), 3.66 (1H, q, J = 7.3 Hz, CH$_3$C**H**), 1.51 (3H, d, J = 7.3 Hz, C**H**$_3$CH); ^{13}C NMR (101 MHz, $CDCl_3$) δ 167.9 (2 × C), 122.7 (q, $J_{C\text{-}F}$ = 276.6 Hz, 2 × C), 61.2 (q, $J_{C\text{-}F}$ = 37.1 Hz, 2 × CH_2), 45.4 (CH), 13.4 (CH_3); ^{19}F NMR (376 MHz, $CDCl_3$) δ −74.1 (t, J = 8.2 Hz, 6 × F); HRMS (ESI) exact mass calculated for $[C_8H_7F_6O_4]^-$ $[M-H]^-$: 281.0254, found: 281.0255.

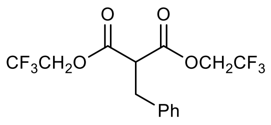

Bis(2,2,2-trifluoroethyl) 2-benzylmalonate (S3b). The title compound was prepared according to General Procedure A, using benzylmalonic acid (4.00 g, 20.6 mmol), TFE (7.52 mL, 103.0 mmol), concentrated H_2SO_4 (0.44 mL, 8.25 mmol), and benzene (20 mL) to give a colorless oil (3.76 g, 51%). R_f = 0.21 (4% EtOAc/petroleum ether); IR 1755 (C=O), 1445, 1347, 1279, 1219, 1159, 1135, 1070, 977, 699 cm^{-1}; ^{1}H NMR (400 MHz, $CDCl_3$) δ 7.22-7.08 (5H, m, Ar**H**), 4.45-4.30 (4H, m, 2 × C**H**$_2$CF$_3$), 3.79 (1H, t, J = 7.9 Hz, PhCH$_2$C**H**), 3.19 (2H, d, J = 7.9 Hz, ArC**H**$_2$); ^{13}C NMR (101 MHz, $CDCl_3$) δ 166.6 (2 × C), 136.5 (C), 128.9 (2 × CH), 128.8 (2 × CH), 127.4 (CH), 122.6 (q, $J_{C\text{-}F}$ = 277.3 Hz, 2 × C), 61.3 (q, $J_{C\text{-}F}$ = 37.3 Hz, 2 × CH_2), 53.0 (CH), 34.6 (CH_2); ^{19}F NMR (376 MHz, $CDCl_3$) δ −73.9 (t, J = 8.2 Hz, 6 × F); HRMS (ESI) exact mass calculated for $[C_{14}H_{12}F_6NaO_4]^+$ $[M+Na]^+$: 381.0532, found: 381.0539.

Bis(2,2,2-trifluoroethyl) 2-(3,4-dimethylphenyl)malonate (S5)

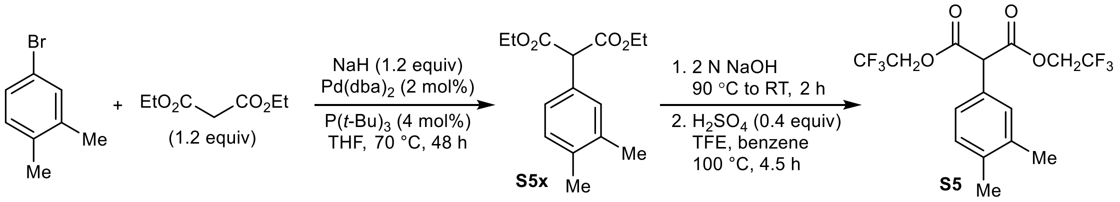

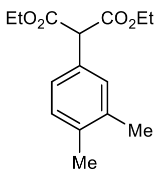

Diethyl 2-(3,4-dimethylphenyl)malonate (S5x).[8] Diethyl malonate (3.45 mL, 22.7 mmol) was added to a suspension of NaH (60% dispersion in mineral oil, 907 mg, 22.7 mmol) in THF (18 mL) over 20 min. The mixture was stirred at room temperature for 10 min, followed addition of 4-bromo-*o*-xylene (2.55 mL, 18.9 mmol), P(t-Bu)$_3$ (1.0 M solution in toluene, 750 μL, 0.76 mmol), Pd(dba)$_2$ (218 mg, 0.38 mmol), and THF (36 mL). The resulting solution was stirred at 70 °C for 48 h. The reaction was cooled to room temperature, quenched with a saturated aqueous NH_4Cl solution (50 mL), and extracted with EtOAc (3 × 50 mL). The combined organic layers were washed with brine (50 mL), dried ($MgSO_4$), filtered, and concentrated under reduced pressure. The residue was purified by column chromatography (0% to 5% EtOAc/petroleum ether) to give the title compound as a colorless oil (1.87 g, 37%). R_f = 0.34 (10% EtOAc/petroleum ether); IR 1730 (C=O), 1503, 1241, 1174, 1140, 1031, 862, 749, 607, 417 cm^{-1}; ^{1}H NMR (500 MHz, $CDCl_3$) δ 7.16 (1H, s, Ar**H**), 7.13-7.12 (2H, m, Ar**H**), 4.55 (1H, s, ArC**H**), 4.27-4.16 (4H, m, 2 × C**H**$_2$CH$_3$), 2.26 (3H, s, ArC**H**$_3$), 2.25 (3H, s, ArC**H**$_3$), 1.27 (3H, t, J = 7.2 Hz, CH$_2$C**H**$_3$); ^{13}C NMR (125 MHz, $CDCl_3$) δ 168.5 (2 × C), 137.0 (C), 136.8 (C), 130.5 (CH), 130.3 (C), 130.0 (CH), 126.7 (CH), 61.8 (2 × CH_2), 57.7 (CH), 19.9 (CH_3), 19.6 (CH_3), 14.2 (2 × CH_3); HRMS (ESI) Exact mass calculated for $[C_{15}H_{20}NaO_4]^+$ $[M+Na]^+$: 287.1254, found 287.1252.

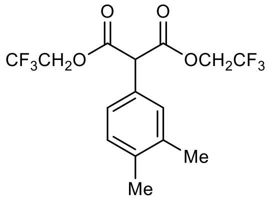

Bis(2,2,2-trifluoroethyl) 2-(3,4-dimethylphenyl)malonate (S5). A suspension of diethyl 2-(3,4-dimethylphenyl)malonate (1.87 g, 7.08 mmol) in 2 N NaOH (15 mL) was vigorously stirred at 90 °C for 2 h. The aqueous mixture was cooled to room temperature, washed with Et_2O (2 × 20 mL), acidified to pH 2 with 6 M aqueous HCl solution and extracted with EtOAc (5 × 40 mL). The combined organic extracts were washed with brine (50 mL), dried ($MgSO_4$), filtered, and concentrated under reduced pressure to give malonic acid as an off-white solid (1.21 g) that was used in the next step without further purification. A microwave vial fitted with a stirrer bar was charged with this malonic acid (1.21 g, 5.81 mmol), TFE (30 mL), and concentrated H_2SO_4 (124 µL, 2.32 mmol). The vial was then capped with a crimp capped PTFE seal and stirred at 100 °C for 4.5 h. The reaction was cooled to room temperature and diluted with a mixture of Et_2O (150 mL). The solution was then washed with a saturated aqueous $NaHCO_3$ solution (3 × 50 mL) and brine (50 mL). The organic layer was dried ($MgSO_4$), filtered, and concentrated under reduced pressure. The residue was purified by column chromatography (0% to 5% EtOAc/petroleum ether) to give the title compound **S5** (1.16 g, 65% over two steps) as a colorless oil. R_f = 0.52 (10% EtOAc/petroleum ether); IR 1755 (C=O), 1409, 1279, 1259, 1125, 1058, 979, 754, 650, 442 cm^{-1}; ^{1}H NMR (400 MHz, $CDCl_3$) δ 7.17-7.10 (3H, m, Ar**H**), 4.76 (1H, s, ArC**H**), 4.62-4.46 (4H, m, 2 × C**H**$_2$CF$_3$), 2.28 (3H, s, ArC**H**$_3$), 2.27 (3H, s, ArC**H**$_3$); ^{13}C NMR (101 MHz, $CDCl_3$) δ 166.3 (2 × C), 137.8 (C), 137.6 (C), 130.4 (CH), 130.3 (CH), 128.1 (C), 126.6 (CH), 122.7 (q, J_{C-F} = 277.3 Hz, 2 × C), 61.4 (q, J_{C-F} = 37.1 Hz, 2 × CH_2), 56.5 (CH), 19.9 (CH_3), 19.6 (CH_3); ^{19}F NMR (376 MHz, $CDCl_3$) δ –73.8 (t, J = 8.2 Hz, 6 × F); HRMS (ESI) Exact mass calculated for $[C_{15}H_{14}F_6NaO_4]^+$ $[M+Na]^+$: 395.0688, found 395.0690.

Bis(2,2,2-trifluoroethyl) 2-(3,5-dimethylphenyl)malonate (S6)

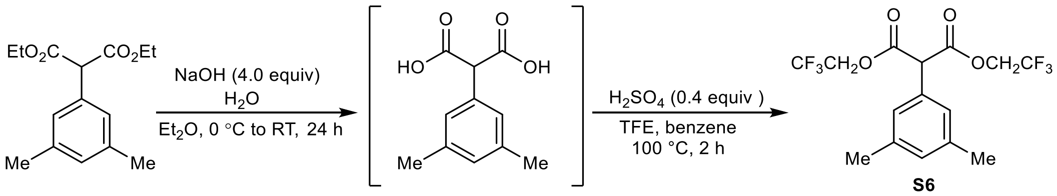

To a stirred solution of diethyl 2-(3,5-dimethylphenyl)malonate[4] (3.10 g, 11.7 mmol) in Et_2O (21 mL) at 0 °C was added a solution of NaOH (1.88 g, 46.9 mmol) in H_2O (40 mL) and the resulting mixture was stirred vigorously at room temperature for 24 h. The aqueous layer was separated and washed with Et_2O (2 × 50 mL), acidified to pH 2 with 6 M aqueous HCl solution, and extracted with EtOAc (5 × 50 mL). The combined organic extracts were washed with brine (50 mL), dried ($MgSO_4$), filtered, and concentrated under reduced pressure to leave the malonic acid as an off white solid (1.80 g) that was used in the next step without further purification. A microwave

vial fitted with a stirrer bar was charged with this malonic acid (1.80 g, 8.65 mmol), TFE (15 mL), benzene (15 mL) and concentrated H_2SO_4 (184 µL, 3.46 mmol). The vial was then capped with a crimp capped PTFE seal and stirred at 100 °C for 5 h. The reaction was cooled to room temperature and diluted with a mixture of Et_2O (150 mL). The solution was then washed with saturated aqueous $NaHCO_3$ solution (3 × 75 mL) and brine (50 mL). The organic layer was dried ($MgSO_4$), filtered and concentrated under reduced pressure. The residue was purified by column chromatography (0% to 2% EtOAc/petroleum ether) to give the title compound **S6** (1.12 g, 25% over two steps) as a pale yellow oil. R_f = 0.35 (10% EtOAc/petroleum ether); IR 1756 (C=O), 1410, 1279, 1251, 1161, 1127, 959, 842, 651, 449 cm^{-1}; ^{1}H NMR (400 MHz, $CDCl_3$) δ 7.03-6.99 (3H, m, Ar**H**), 4.74 (1H, s, ArC**H**), 4.62-4.46 (4H, m, 2 × C**H**$_2$CF$_3$), 2.33 (3H, s, ArC**H**$_3$), 2.32 (3H, s, ArC**H**$_3$); ^{13}C NMR (101 MHz, $CDCl_3$) δ 166.2 (2 × C), 138.8 (2 × C), 130.9 (CH), 130.5 (C), 127.0 (2 × CH), 122.7 (q, $J_{C\text{-}F}$ = 277.4 Hz, 2 × C), 61.4 (q, $J_{C\text{-}F}$ = 37.2 Hz, 2 × CH_2), 56.7 (CH), 21.4 (2 × CH_3); ^{19}F NMR (376 MHz, $CDCl_3$) δ –73.8 (t, J = 8.2 Hz, 6 × F); HRMS (ESI) Exact mass calculated for $[C_{15}H_{14}F_6NaO_4]^+$ $[M+Na]^+$: 395.0688, found 395.0679.

Bis(2,2,2-trifluoroethyl) 2-(naphthalen-2-yl)malonate (S11)

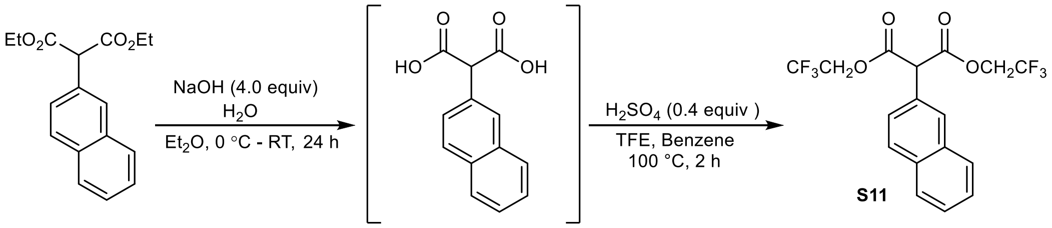

A solution of diethyl 2-(naphthalen-2-yl)malonate[8] (3.00 g, 10.5 mmol) in 2 N aqueous NaOH solution (25 mL) was vigorously stirred at 90 °C for 1 h. The mixture was cooled to room temperature, washed with Et_2O (2 × 50 mL), acidified to pH 2 with 6 M aqueous HCl solution and extracted with EtOAc (5 × 50 mL). The combined organic extracts were washed with brine (50 mL), dried ($MgSO_4$), filtered, and concentrated under reduced pressure to leave the malonic acid as a pale green solid (2.01 g) that was used in the next step without further purification. A microwave vial fitted with a stirrer bar was charged with this malonic acid (2.01 g, 8.73 mmol), TFE (40 mL), and concentrated H_2SO_4 (186 µL, 3.49 mmol). The vial was then capped with a crimp capped PTFE seal and stirred at 100 °C for 1.5 h. The reaction was cooled to room temperature and diluted with Et_2O (300 mL). The solution was then washed with saturated aqueous $NaHCO_3$ solution (3 × 150 mL) and brine (50 mL). The organic layer was then dried ($MgSO_4$), filtered and concentrated under reduced pressure. The residue was purified by column chromatography (0% to 2% EtOAc/petroleum ether) to give the title compound **S11** (1.22 g, 44% over two steps) as a white solid. R_f = 0.37 (10% EtOAc/petroleum ether); m.p. 49-50 °C (CH_2Cl_2/petroleum ether); IR 1765

(C=O), 1753 (C=O), 1408, 1269, 1155, 1127, 978, 823, 756, 654 cm^{-1}; ^{1}H NMR (400 MHz, $CDCl_3$) δ 7.90-7.83 (4H, m, Ar**H**), 7.55-7.50 (3H, m, Ar**H**), 5.00 (1H, s, ArC**H**), 4.65-4.49 (4H, m, 2 × C**H**$_2$CF$_3$); ^{13}C NMR (101 MHz, $CDCl_3$) δ 166.1 (2 × C), 133.4 (C), 133.3 (C), 129.1 (CH), 129.0 (CH), 128.2 (CH), 128.1 (C), 127.9 (CH), 127.1 (CH), 126.8 (CH), 126.2 (CH), 122.6 (q, J_{C-F} = 277.3 Hz, 2 × C), 61.5 (q, J_{C-F} = 37.1 Hz, 2 × CH_2), 57.0 (CH); ^{19}F NMR (376 MHz, $CDCl_3$) δ –73.7 (t, J = 8.2 Hz, 6 × F); HRMS (ESI) Exact mass calculated for $[C_{17}H_{12}F_6NaO_4]^+$ $[M+Na]^+$: 417.0532, found 417.0522.

General Procedure B[3,4]: Copper Catalyzed α-Arylation of Malonate Ester S1

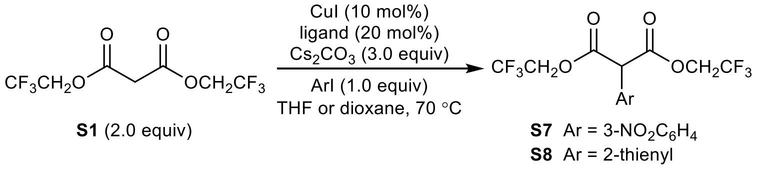

An oven-dried Schlenk tube equipped with a magnetic stir bar and a Teflon stopcock was evacuated while hot and then cooled under argon. The tube was charged sequentially with CuI (10 mol %), ligand (20 mol %), Cs_2CO_3 (3.0 equiv), and the aryl iodide (1.00 equiv), if a solid. The tube was evacuated and backfilled with argon (3 times), and the Teflon stopcock was replaced with a rubber septum. The aryl iodide (if a liquid) was added volumetrically (1.00 equiv), followed by bis(2,2,2-trifluoroethyl) malonate **S1**[2] (2.0 equiv) and anhydrous THF or dioxane. The septum was replaced by the Teflon stopcock under a positive pressure of argon, and the sealed tube was placed in an oil bath preheated to 70 ˚C. After stirring for the designated time period, the reaction was cooled to room temperature and then partitioned between EtOAc (3 × 5 mL per mmol of aryl iodide) and saturated aqueous NH_4Cl solution (2 mL per mmol of aryl iodide). The organic portion was dried ($MgSO_4$), filtered, and concentrated on a rotary evaporator. The material thus obtained was purified by silica gel chromatography to give the product α-aryl malonate.

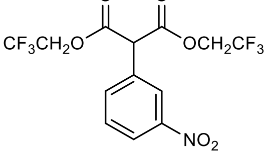

Bis(2,2,2-trifluoroethyl) 2-(3-nitrophenyl)malonate (S7). The title compound was prepared according to General Procedure B using 1-iodo-3-nitrobenzene (1.00 g, 4.02 mmol), bis(2,2,2-trifluoroethyl) malonate **S1**[2] (2.15 g, 8.03 mmol), CuI (76 mg, 0.40 mmol), 2-phenylphenol (137 mg, 0.80 mmol), Cs_2CO_3 (3.92 g, 12.05 mmol) and THF (4 mL) for 24 h, and purified by column chromatography (0% to 10% EtOAc/petroleum ether) to give a pale yellow solid (401 mg, 26%). R_f = 0.13 (10% EtOAc/petroleum ether); IR 1756 (C=O), 1527, 1308, 1291, 1213, 1160, 1043, 977, 670, 409 cm^{-1}; m.p. 50-51 °C (Et_2O/*n*-pentane); ^{1}H NMR (400 MHz, $CDCl_3$) δ 8.31-8.26 (2H, m, Ar**H**), 7.78 (1H, ddd, J = 7.8, 1.8, 1.1 Hz, Ar**H**), 7.62 (1H, t, J = 8.0 Hz, Ar**H**), 4.96 (1H, s, ArC**H**),

4.65-4.51 (4H, m, 2 × C**H**$_2$CF$_3$); ^{13}C NMR (101 MHz, CDCl$_3$) δ 165.1 (2 × C), 148.6 (C), 135.4 (CH), 132.6 (C), 130.2 (CH), 124.7 (CH), 124.3 (CH), 122.5 (q, J_{C-F} = 277.4 Hz, 2 × C), 61.8 (q, J_{C-F} = 37.0 Hz, 2 × CH$_2$), 56.2 (CH); ^{19}F NMR (376 MHz, CDCl$_3$) δ –73.8 (t, *J* = 8.1 Hz, 6 × F); HRMS (ESI) Exact mass calculated for [C$_{13}$H$_8$F$_6$NO$_6$]$^-$ [M–H]$^-$: 388.0261, found 388.0273.

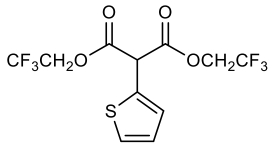

Bis(2,2,2-trifluoroethyl) 2-(2-thienyl)malonate (S8). The title compound was prepared according to General Procedure B, using 2-iodothiophene (5.00 g, 23.8 mmol), bis(2,2,2-trifluoroethyl) malonate **S1**[2] (12.77 g, 47.61 mmol), CuI (453 mg, 2.38 mmol), 2-picolinic acid (586 mg, 4.76 mmol), Cs$_2$CO$_3$ (23.26 g, 71.41 mmol) and 1,4-dioxane (25 mL) for 72 h, and purified by column chromatography (0% to 10% EtOAc/petroleum ether) to give a pale yellow oil (2.51 g, 30%). R$_f$ = 0.33 (10% EtOAc/petroleum ether); IR 1760 (C=O), 1411, 1281, 1228, 1166, 1051, 980, 859, 752, 707 cm^{-1}; ^{1}H NMR (400 MHz, CDCl$_3$) δ 7.37 (1H, dd, *J* = 5.2, 1.2 Hz, Ar**H**), 7.16 (1H, ddd, *J* = 3.6, 1.3, 0.6 Hz, Ar**H**), 7.03 (1H, dd, *J* = 5.2, 3.6 Hz, Ar**H**), 5.14 (1H, s, ArC**H**), 4.57 (4H, qd, *J* = 8.2, 3.1 Hz, 2 × C**H**$_2$CF$_3$); ^{13}C NMR (101 MHz, CDCl$_3$) δ 165.2 (2 × C), 130.9 (C), 128.9 (CH), 127.5 (CH), 127.1 (CH), 122.5 (q, J_{C-F} = 277.3 Hz, 2 × C), 61.7 (q, J_{C-F} = 37.4 Hz, 2 × CH$_2$), 52.0 (CH); ^{19}F NMR (376 MHz, CDCl$_3$) δ –73.8 (t, *J* = 8.2 Hz, 6 × F); HRMS (ESI) Exact mass calculated for [C$_{11}$H$_8$F$_6$NaO$_4$S]$^+$ [M+Na]$^+$: 372.9940, found 372.9943.

General Procedure C: Rhodium Catalyzed α-Alkoxylation of Bis(2,2,2-trifluoroethyl) 2-diazomalonate

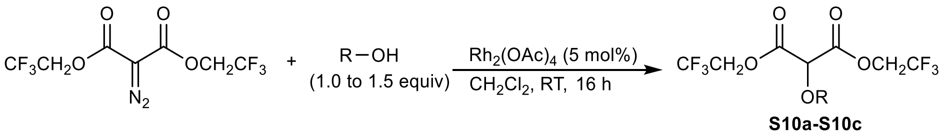

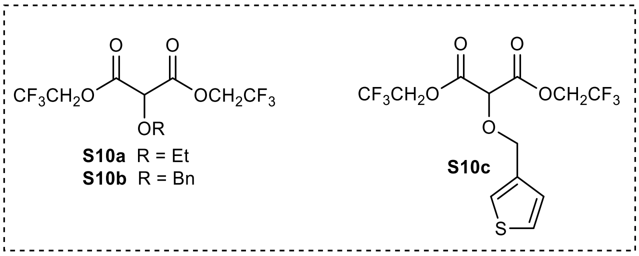

Following a modification of a reported procedure,[9] bis(2,2,2-trifluoroethyl) 2-diazomalonate[2] (1 equiv) was dissolved in CH$_2$Cl$_2$ [10 mL/g of bis(2,2,2-trifluoroethyl) 2-diazomalonate] and Rh$_2$(OAc)$_4$ (5 mol%), followed by addition of the alcohol (1.0–1.5 equiv). The mixture was stirred at room temperature for 16 h, diluted with CH$_2$Cl$_2$ [35 mL/g of bis(2,2,2-trifluoroethyl) 2-diazomalonate], and filtered through a plug of silica (3 cm in height and 7 cm wide). The silica plug

was washed with CH_2Cl_2 [15 mL/g of bis(2,2,2-trifluoroethyl) 2-diazomalonate], and the filtrate was concentrated under reduced pressure to give the title compound **S10a/S10b/S10c**.

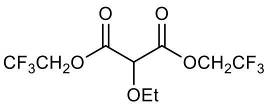

Bis(2,2,2-trifluoroethyl) 2-ethoxymalonate (S10a). The title compound was prepared according to General Procedure C, using bis(2,2,2-trifluoroethyl) 2-diazomalonate[2] (3.34 g, 11.4 mmol), $Rh_2(OAc)_4$ (251 mg, 0.57 mmol), and EtOH (994 μL, 17.0 mmol) in CH_2Cl_2 (35 mL) for 16 h to give a colorless oil (1.61 g, 45%). R_f = 0.19 (10% EtOAc/petroleum ether); IR 1766 (C=O), 1413, 1280, 1156, 1117, 1053, 978, 915, 842, 650 cm^{-1}; ^{1}H NMR (400 MHz, $CDCl_3$) δ 4.71 (1H, s, CH_2OC**H**), 4.59 (4H, q, J = 8.2 Hz, 2 × C**H**$_2CF_3$), 3.71 (2H, q, J = 7.0 Hz, C**H**$_2CH_3$), 1.31 (3H, q, J = 7.0 Hz, CH_2C**H**$_3$); ^{13}C NMR (101 MHz, $CDCl_3$) δ 164.7 (2 × C), 122.5 (q, $J_{C\text{-}F}$ = 277.3 Hz, 2 × C), 78.1 (CH), 67.8 (CH_2), 61.4 (q, $J_{C\text{-}F}$ = 37.5 Hz, 2 × CH_2), 15.0 (CH_3); ^{19}F NMR (376 MHz, $CDCl_3$) δ –73.9 (t, J = 8.1 Hz, 6 × F); HRMS (ESI) Exact mass calculated for $[C_9H_{10}F_6NaO_5]^+$ $[M+Na]^+$: 335.0325, found 335.0329.

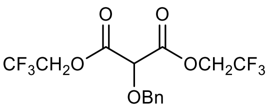

Bis(2,2,2-trifluoroethyl) 2-(benzyloxy)malonate (S10b). The title compound was prepared according to General Procedure C, using bis(2,2,2-trifluoroethyl) 2-diazomalonate[2] (2.60 g, 8.84 mmol), $Rh_2(OAc)_4$ (195 mg, 0.44 mmol), and benzyl alcohol (1.05 g, 9.72 mmol) in CH_2Cl_2 (26 mL) to give a colorless oil (2.71 g, 82%). R_f = 0.11 (5% EtOAc/petroleum ether); IR 1765 (C=O), 1413, 1280, 1209, 1158, 1054, 976, 915, 741, 698 cm^{-1}; ^{1}H NMR (400 MHz, $CDCl_3$) δ 7.41-7.32 (5H, m, Ar**H**), 4.75 (2H, s, ArC**H**$_2$O), 4.73 (1H, s, CH_2OC**H**), 4.56 (4H, q, J = 8.2 Hz, 2 × C**H**$_2CF_3$); ^{13}C NMR (101 MHz, $CDCl_3$) δ 164.5 (2 × C), 135.4 (C), 128.90 (CH), 128.88 (2 × CH), 128.7 (2 × CH), 122.5 (q, $J_{C\text{-}F}$ = 277.3 Hz, 2 × C), 76.6 (CH), 73.5 (CH_2), 61.5 (q, $J_{C\text{-}F}$ = 37.5 Hz, 2 × CH_2); ^{19}F NMR (376 MHz, $CDCl_3$) δ –73.8 (t, J = 8.1 Hz, 6 × F); HRMS (ESI) Exact mass calculated for $[C_{14}H_{12}F_6NaO_5]^+$ $[M+Na]^+$: 397.0481, found 397.0487.

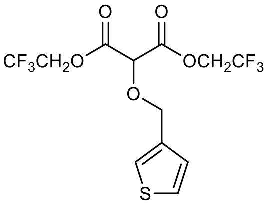

Bis(2,2,2-trifluoroethyl) 2-(thiophen-3-ylmethoxy)malonate (S10c). The title compound was prepared according to General Procedure C, using bis(2,2,2-trifluoroethyl) 2-diazomalonate[2] (1.50 g, 5.10 mmol), $Rh_2(OAc)_4$ (112.7 mg, 0.26 mmol), and 3-thiophenemethanol (582 mg, 5.10 mmol) in CH_2Cl_2 (15 mL) to give a yellow oil (1.60 g, 82%). R_f = 0.17 (10% EtOAc/petroleum ether); IR 1764 (C=O), 1412, 1280, 1156, 1054, 976, 858, 787, 693, 562 cm^{-1}; ^{1}H NMR (400 MHz, $CDCl_3$) δ 7.35 (1H, dd, J = 5.0, 3.0 Hz, Ar**H**), 7.31-7.27 (1H, m, Ar**H**), 7.11 (1H, dd, J = 4.9, 1.3 Hz, Ar**H**), 4.77 (2H, s, ArC**H**$_2$), 4.72 (1H, s, C**H**), 4.56 (4H, q, J = 8.2 Hz, 2 × C**H**$_2CF_3$); ^{13}C NMR (101 MHz, $CDCl_3$) δ 164.6 (2 × C), 136.4 (C), 127.7 (CH), 127.0 (CH), 125.3

(CH), 122.5 (q, $J_{C\text{-}F}$ = 277.4 Hz, 2 × C), 76.3 (CH), 68.3 (CH_2), 61.5 (q, $J_{C\text{-}F}$ = 37.5 Hz, 2 × CH_2); ^{19}F NMR (376 MHz, $CDCl_3$) δ –73.7 (t, J = 8.1 Hz, 6 × F); HRMS (ESI) Exact mass calculated for $[C_{12}H_{10}F_6NaO_5S]^+$ $[M+Na]^+$: 403.0045, found: 403.0042.

Bis(2,2,2-trifluoroethyl) 2-[methyl(4-methylphenyl)amino]malonate (S10d)

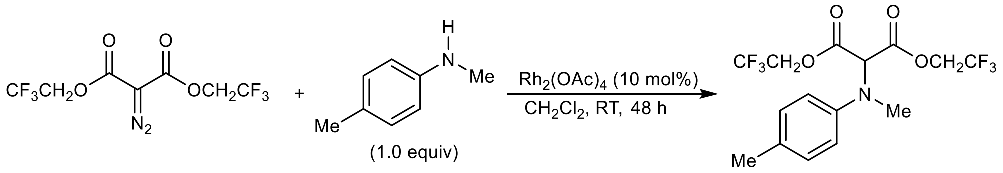

S10d

The title compound was prepared by modification of reported procedure.[9] Bis(2,2,2-trifluoroethyl) 2-diazomalonate[2] (1.00 g, 3.40 mmol) was dissolved in CH_2Cl_2 (10 mL) and $Rh_2(OAc)_4$ (150 mg, 0.34 mmol), *N*-methyl-*p*-toluidine (0.43 mL, 3.40 mmol) were added. The mixture was stirred at room temperature for 48 h, diluted with CH_2Cl_2 (35 mL), and filtered through a plug of silica (3 cm in height and 7 cm wide). The silica plug was washed with CH_2Cl_2 (15 mL), and filtrate was concentrated under reduced pressure to give the title compound **S10d** as a yellow oil (940 mg, 71%). R_f = 0.38 (15% EtOAc/petroleum ether); IR 1759 (C=O), 1519, 1411, 1274, 1161, 1112, 1052, 972, 806, 649 cm^{-1}; ^{1}H NMR (400 MHz, $CDCl_3$) δ 7.12-7.08 (2H, m, Ar**H**), 6.79-6.75 (3H, m, Ar**H**), 5.31 (1H, s, ArC**H**), 4.66-4.52 (4H, m, 2 × C**H**$_2$CF$_3$), 3.04 (3H, s, NC**H**$_3$), 2.28 (3H, s, ArC**H**$_3$); ^{13}C NMR (101 MHz, $CDCl_3$) δ 165.7 (2 × C), 146.4 (C), 130.1 (2 × CH), 126.7 (C), 122.6 (q, J = 277.3 Hz, 2 × CF_3), 114.6 (2 × CH), 66.2 (CH), 61.3 (q, J = 37.3 Hz, 2 × CH_2), 35.9 (CH_3), 20.4 (CH_3); ^{19}F NMR (376 MHz, $CDCl_3$) δ −73.7 (t, J = 8.2 Hz, 6 × F); HRMS (ESI) Exact mass calculated for $[C_{15}H_{15}F_6NNaO_4]^+$ $[M+Na]^+$: 410.0797, found: 410.0797.

6-Bromo-2-methylhex-2-en-4-yne (S17)

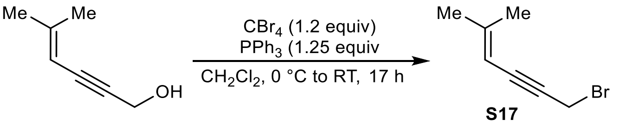

S17

To a solution of 5-methylhex-4-en-2-yn-1-ol[10] (0.90 g, 8.17 mmol) and CBr_4 (3.25 g, 9.80 mmol) in CH_2Cl_2 (15 mL) was added a solution of PPh_3 (2.68 g, 10.2 mmol) in CH_2Cl_2 (10 mL) at 0 °C dropwise. The mixture was warmed to room temperature and stirred for 17 h. The reaction was concentrated under reduced pressure and purified by column chromatography (*n*-pentane) to give *propargyl bromide* **S17** as a pale yellow oil (1.39 g, 98%). R_f = 0.39 (100% petroleum ether); IR 2910, 2207, 1633, 1444, 1378, 1334, 1220, 816, 598, 531 cm^{-1}; ^{1}H NMR (400 MHz, $CDCl_3$) δ 5.29 (1H, app tt, J = 2.4, 1.2 Hz, **H**C=C(CH$_3$)$_2$), 4.12 (2H, d, J = 2.2 Hz, C**H**$_2$), 1.90 (3H, d, J = 1.1 Hz,

=C(C**H**$_3$)$_2$), 1.81 (3H, d, *J* = 1.4 Hz, =C(C**H**$_3$)$_2$); ^{13}C NMR (101 MHz, CDCl$_3$) δ 151.1 (C), 104.6 (CH), 86.2 (C), 85.5 (C), 25.0 (CH$_3$), 21.3 (CH$_3$), 16.3 (CH$_2$).

General Procedure D: Synthesis of Substrates 1a-1d, 1f, 1g, 1i, 1j, 1m-1o, 1q, and 1r

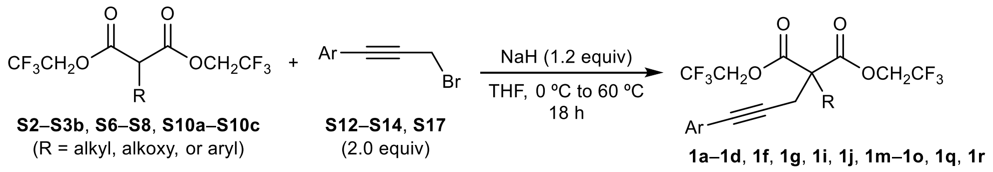

A solution of the appropriate α-substituted bis(2,2,2-trifluoroethyl) malonate **S2**–**S3b**, **S6**–**S8**, or **S10a**–**S10c** (1.0 equiv) in THF (1.0 M) was added to an ice-cooled suspension of NaH (1.2 equiv) in THF (0.35 M). The resulting solution was warmed to room temperature and stirred for *ca.* 30 min. The appropriate alkynyl bromide **S12**–**S14** or **S17** (2.0 equiv) was added dropwise and the resulting solution was stirred at 60 °C for 18 h for the specified time (except for the reactions of α-benzyl bis(2,2,2-trifluoroethyl) malonate **S3a** and **S3b**, which were carried out at room temperature for 18 h). The reaction was cooled to room temperature and quenched with a saturated aqueous NH_4Cl solution (15 mL per mmol of malonate). This mixture was extracted with EtOAc (3 × 5 mL per mmol of malonate) and the combined organic layer was dried ($MgSO_4$), filtered and concentrated under reduced pressure. The residue was purified by column chromatography (EtOAc/petroleum ether) to give the title compound.

General Procedure E: Synthesis of Substrates 1e, 1h, and 1p

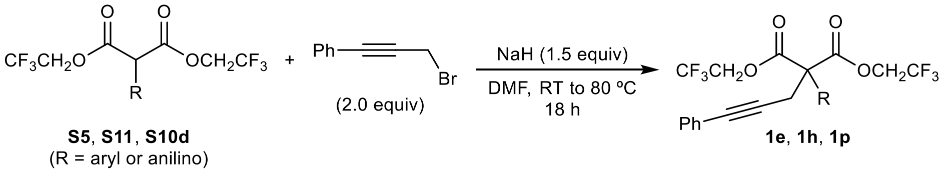

A solution of the appropriate α-substituted bis(2,2,2-trifluoroethyl) malonate **S5**, **S11**, or **S10d** (1.00 g, 1.0 equiv) in DMF (4 mL) was added to a suspension of NaH (1.5 equiv) in DMF (6 mL) at room temperature. The resulting solution was stirred at room temperature for *ca.* 30 min. The alknyl bromide **S12**[5] (80 wt. % solution in toluene, 2.0 equiv) was added dropwise and the resulting solution was stirred at 80 °C for 18 h. The mixture was cooled to room temperature and quenched with saturated aqueous NH_4Cl solution (3 mL per mmol of malonate). This mixture was extracted with EtOAc (3 × 5 mL per mmol of malonate) and the combined organic layer was washed with H_2O (3 mL per mmol of malonate), brine (3 mL per mmol of malonate), dried ($MgSO_4$), filtered and concentrated under reduced pressure. The residue was purified by column chromatography (EtOAc/petroleum ether) to give the title compound.

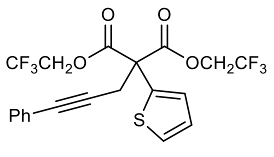

Bis(2,2,2-trifluoroethyl) 2-(3-phenylprop-2-yn-1-yl)-2-(2-thienyl)malonate (1a). The title compound was prepared according to General Procedure D, using malonate **S8** (2.00 g, 5.71 mmol), NaH (60% dispersion in mineral oil, 273 mg, 6.85 mmol), and alkynyl bromide **S12**[5] (75 wt. % in toluene, 2.97 mL, 11.4 mmol) in THF (28 mL) for 24 h, and purified by column chromatography (0% to 2% EtOAc/petroleum ether) to give a pale brown oil (1.62 g, 61%). R_f = 0.35 (7% EtOAc/petroleum ether); IR 1757 (C=O), 1409, 1282, 1211, 1156, 1059, 971, 840, 756, 691 cm^{-1}; ^{1}H NMR (400 MHz, $CDCl_3$) δ 7.29 (1H, dd, *J* = 5.1, 1.2 Hz, Ar**H**), 7.24-7.12 (6H, m, Ar**H**), 6.94 (1H, dd, *J* = 5.2, 3.7 Hz, Ar**H**), 4.59-4.42 (4H, m, 2 × C**H**$_2$CF$_3$), 3.44 (2H, s, C**H**$_2$C≡C); ^{13}C NMR (101 MHz, $CDCl_3$) δ 166.8 (2 × C), 136.0 (C), 131.8 (2 × CH), 128.5 (CH), 128.3 (2 × CH), 127.7 (CH), 127.2 (CH), 126.6 (CH), 122.8 (C), 122.5 (q, $J_{C\text{-}F}$ = 277.8 Hz, 2 × C), 85.3 (C), 82.7 (C), 61.8 (q, $J_{C\text{-}F}$ = 37.5 Hz, 2 × CH_2), 59.9 (C), 29.0 (CH_2); ^{19}F NMR (376 MHz, $CDCl_3$) δ –73.7 (t, *J* = 8.1 Hz, 6 × F); HRMS (ESI) Exact mass calculated for $[C_{20}H_{14}F_6NaO_4S]^+$ $[M+Na]^+$: 487.0409, found 487.0423.

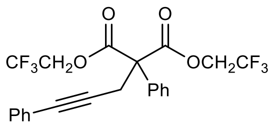

Bis(2,2,2-trifluoroethyl) 2-phenyl-2-(3-phenylprop-2-yn-1-yl)malonate (1b). The title compound was prepared according to General Procedure D, using malonate **S2** (1.00 g, 2.90 mmol), NaH (60% dispersion in mineral oil, 139 mg, 3.49 mmol), and alkynyl bromide **S12**[5] (1.13 g, 5.81 mmol) in THF (13 mL) for 18 h, and purified by column chromatography (0% to 2% EtOAc/petroleum ether) to give a yellow oil (506 mg, 38%). R_f = 0.45 (10% EtOAc/petroleum ether); IR 2976, 1754 (C=O), 1599, 1409, 1154, 1075, 973, 727, 691, 528 cm^{-1}; ^{1}H NMR (400 MHz, $CDCl_3$) δ 7.53-7.49 (2H, m, Ar**H**), 7.43-7.35 (3H, m, Ar**H**), 7.32-7.22 (5H, m, Ar**H**), 4.59 (4H, qd, *J* = 8.3, 2.5 Hz, 2 × C**H**$_2$CF$_3$), 3.50 (2H, s, C**H**$_2$C≡C); ^{13}C NMR (101 MHz, $CDCl_3$) δ 167.6 (2 × C), 134.1 (C), 131.7 (2 × CH), 128.9 (CH), 128.7 (2 × CH), 128.39 (CH), 128.35 (2 × CH), 127.9 (2 × CH), 122.9 (C), 122.6 (d, $J_{C\text{-}F}$ = 277.5 Hz, 2 × C), 84.8 (C), 83.4 (C), 62.5 (C), 61.5 (q, $J_{C\text{-}F}$ = 37.4 Hz, 2 × CH_2), 27.1 (CH_2); ^{19}F NMR (376 MHz, $CDCl_3$) δ −73.6 (t, *J* = 8.2 Hz, 6 × F); HRMS (ESI) exact mass calculated for $[C_{22}H_{16}F_6NaO_4]^+$ $[M+Na]^+$: 481.0845, found 481.0856.

Bis(2,2,2-trifluoroethyl) 2-(4-methoxyphenyl)-2-(3-phenylprop-2-yn-1-yl)malonate (1c)

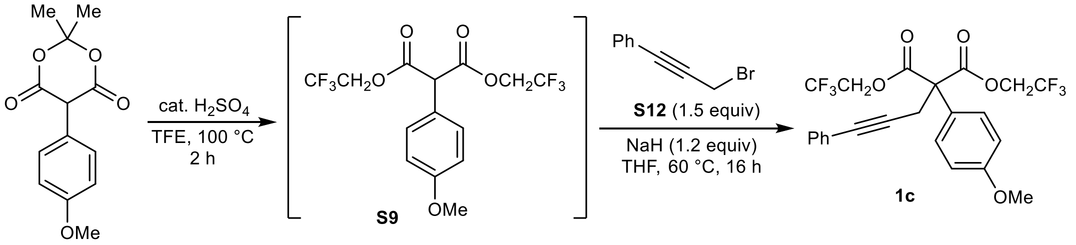

A microwave vial fitted with a stirrer bar was charged with 5-(4-methoxyphenyl)-2,2-dimethyl-1,3-dioxane-4,6-dione[11] (500 mg, 2.00 mmol), TFE (10 mL) and concentrated H_2SO_4 (43 µL, 0.80 mmol). The vial was then capped with a crimp capped PTFE seal and heated at 100 °C for 2 h. The reaction was cooled to room temperature and diluted with a mixture of Et_2O/petroleum ether (3:7, 100 mL). This solution was washed with aqueous Na_2CO_3 solution (5% w/w, 3 × 100 mL) and brine (50 mL). The organic layer was dried (Na_2SO_4) and concentrated under reduced pressure. The residual solvent was removed under high vacuum to leave the crude malonate ester **S9** (401 mg), which was used in the next step without further purification. A solution of this malonate ester **S9** in THF (5 mL) was added to an ice-cooled suspension of NaH (60% dispersion in mineral oil, 51.4 mg, 1.29 mmol) in THF (10 mL). The resulting solution was warmed to room temperature and stirred for 30 min. Alkynyl bromide **S12**[5] (75 wt. % in toluene, 0.42 mL, 1.60 mmol) was added dropwise and the resulting solution was warmed to 60 °C and stirred for 16 h. The reaction was cooled to room temperature and quenched with a saturated aqueous NH_4Cl solution (50 mL). This mixture was extracted with EtOAc (3 × 20 mL) and the combined organic layers were dried (Na_2SO_4), filtered and concentrated under reduced pressure. The residue was purified by column chromatography (0 to 10% EtOAc/petroleum ether) to give the title compound **1c** (372 mg, 38% over two steps) as a colorless oil. R_f = 0.37 (5% EtOAc/petroleum ether); IR 2972, 1754 (C=O), 1610, 1515, 1410, 1283, 1214, 1156, 1081, 528 cm^{-1}; ^1H NMR (400 MHz, $CDCl_3$) δ 7.47-7.43 (2H, m, Ar**H**), 7.31-7.23 (5H, m, Ar**H**), 6.95-6.91 (2H, m, Ar**H**), 4.59 (4H, qd, J = 8.3, 1.8 Hz, 2 × C**H**$_2$CF$_3$), 3.82 (3H, s, OC**H**$_3$), 3.49 (2H, s, C**H**$_2$C≡C); ^{13}C NMR (101 MHz, $CDCl_3$) δ 167.8 (2 × C), 159.8 (C), 131.8 (2 × CH), 129.2 (2 × CH), 128.4 (CH), 128.3 (2 × CH), 125.8 (C), 122.9 (C), 122.7 (q, $J_{C\text{-}F}$ = 277.5 Hz, 2 × C), 114.0 (2 × CH), 84.7 (C), 83.4 (C), 61.8 (C), 61.5 (q, $J_{C\text{-}F}$ = 37.4 Hz, 2 × CH_2), 55.4 (CH_3), 26.9 (CH_2); ^{19}F NMR (376 MHz, $CDCl_3$) δ –73.6 (t, J = 8.2 Hz, 6 × F); HRMS (ESI) Exact mass calculated for $[C_{23}H_{18}F_6NaO_5]^+$ $[M+Na]^+$: 511.0951, found 511.0939.

Bis(2,2,2-trifluoroethyl) 2-(3-phenylprop-2-yn-1-yl)-2-(3-methylphenyl)malonate (1d)

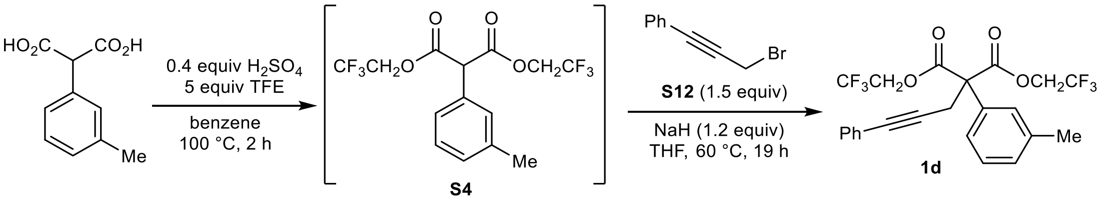

A microwave vial fitted with a stirrer bar was charged with 2-(3-methylphenyl)malonic acid[12] (375 mg, 1.93 mmol), TFE (695 µL, 9.65 mmol) and concentrated H_2SO_4 (41 µL, 0.77 mmol). The vial was then capped with a crimp capped PTFE seal and heated at 100 °C for 2 h. The reaction was cooled to room temperature and diluted with a mixture of Et_2O/petroleum ether (3:7, 75 mL). The solution was then washed with an aqueous solution of Na_2CO_3 (5% w/w, 3 × 75 mL) and brine (50 mL). The organic layer was then dried (Na_2SO_4) and concentrated under reduced pressure. The residual solvent was removed under high vacuum to leave the crude malonate ester **S4** (350 mg), which was used in the next step without further purification. A solution of this malonate ester in THF (2 mL) was added to an ice cooled suspension of NaH (60% dispersion in mineral oil, 46.6 mg, 1.17 mmol) in THF (5 mL). The resulting solution was warmed to room temperature and stirred for 30 min. Alkynyl bromide **S12**[5] (75 wt. % in toluene, 506 µL, 1.95 mmol) was added dropwise and the resulting solution was warmed to 60 °C and stirred for 19 h. The reaction was cooled to room temperature and quenched with a saturated aqueous NH_4Cl solution (40 mL). This solution was extracted with EtOAc (3 × 15 mL) and the combined organic layers were dried (Na_2SO_4), filtered and concentrated under reduced pressure. The residue was purified by column chromatography (0% to 2% EtOAc/petroleum ether) to give title compound **1d** (274 mg, 30% over two steps) as a yellow oil. R_f = 0.41 (10% EtOAc/petroleum ether); IR 2975, 1754 (C=O), 1491, 1409, 1281, 1158, 1080, 972, 757, 691 cm^{-1}; ^{1}H NMR (400 MHz, $CDCl_3$) δ 7.33-7.18 (9H, m, Ar**H**), 4.60 (4H, q, J = 8.2 Hz, 2 × C**H**$_2$CF$_3$), 3.49 (2H, s, C**H**$_2$C≡C), 2.38 (3H, s, ArC**H**$_3$); ^{13}C NMR (101 MHz, $CDCl_3$) δ 167.6 (2 × C), 138.4 (C), 134.0 (C), 131.7 (2 × CH), 129.6 (CH), 128.6 (CH), 128.5 (CH), 128.35 (CH), 128.34 (2 × CH), 124.8 (CH), 122.9 (C), 122.7 (q, J_{C-F} = 277.7 Hz, 2 × C), 84.7 (C), 83.6 (C), 62.5 (C), 61.5 (q, J_{C-F} = 37.4 Hz, 2 × CH_2), 27.1 (CH_2), 21.7 (CH_3); ^{19}F NMR (376 MHz, $CDCl_3$) δ –73.6 (t, J = 8.2 Hz, 6 × F); HRMS (ESI) Exact mass calculated for $[C_{23}H_{18}F_6NaO_4]^+$ $[M+Na]^+$: 495.1001, found 495.1010.

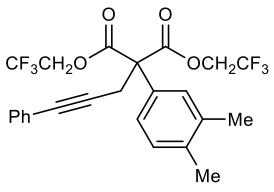

Bis(2,2,2-trifluoroethyl) 2-(3,4-dimethylphenyl)-2-(3-phenylprop-2-yn-1-yl)malonate (1e). The title compound was prepared according to General Procedure E, using malonate **S5** (1.00 g, 2.69 mmol), NaH (60% dispersion in mineral oil, 161 mg, 4.03 mmol), and alkynyl bromide **S12**[5] (80 wt. % solution in toluene, 1.31 mL, 5.37 mmol) in DMF (10 mL) for 18 h, and purified by column

chromatography (0% to 3% EtOAc/petroleum ether) to give a brown oil (1.05 g, 80%). R_f = 0.48 (6% EtOAc/petroleum ether); IR 1754 (C=O), 1443, 1409, 1281, 1067, 973, 756, 690, 650, 553 cm^{-1}; ^{1}H NMR (400 MHz, $CDCl_3$) δ 7.33-7.23 (7H, m, Ar**H**), 7.16 (1H, *J* = 8.0 Hz, Ar**H**), 4.59 (4H, q, *J* = 8.3 Hz, 2 × C**H**$_2$CF$_3$), 3.49 (2H, s, C**H**$_2$C≡C), 2.28 (3H, s, ArC**H**$_3$), 2.27 (3H, s, ArC**H**$_3$); ^{13}C NMR (101 MHz, $CDCl_3$) δ 167.7 (2 × C), 137.5 (C), 136.9 (C), 131.8 (2 × CH), 131.4 (C), 129.9 (CH), 129.1 (CH), 128.3 (3 × CH), 125.1 (CH), 123.0 (C), 122.7 (q, $J_{C\text{-}F}$ = 277.4 Hz, 2 × C), 84.6 (C), 83.7 (C), 62.2 (C), 61.5 (q, $J_{C\text{-}F}$ = 37.4 Hz, 2 × CH_2), 27.1 (CH_2), 20.1 (CH_3), 19.6 (CH_3); ^{19}F NMR (376 MHz, $CDCl_3$) δ −73.6 (t, *J* = 8.2 Hz, 6 × F); HRMS (ESI) exact mass calculated for $[C_{24}H_{20}F_6NaO_4]^+$ $[M+Na]^+$: 509.1158, found 509.1158.

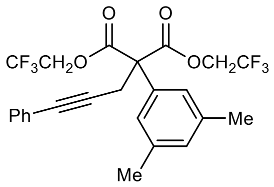

Bis(2,2,2-trifluoroethyl) 2-(3,5-dimethylphenyl)-2-(3-phenylprop-2-yn-1-yl)malonate (1f). The title compound was prepared according to General Procedure D, using malonate **S6** (464 mg, 1.25 mmol), NaH (60% dispersion in mineral oil, 60 mg, 1.50 mmol), and alkynyl bromide **S12**[5] (486 mg, 2.49 mmol, which was added neat, using 0.5 mL of THF to rinse) in THF (5 mL) for 18 h, and purified by column chromatography (0% to 2% EtOAc/petroleum ether) to give a yellow oil (413 mg, 68%). R_f = 0.45 (6% EtOAc/petroleum ether); IR 2974, 1755 (C=O), 1603, 1381, 1281, 1156, 1078, 845, 705, 650 cm^{-1}; ^{1}H NMR (400 MHz, $CDCl_3$) δ 7.34-7.24 (5H, m, Ar**H**), 7.11 (2H, m, Ar**H**), 7.02-7.01 (1H, m, Ar**H**), 4.64-4.57 (4H, m, 2 × C**H**$_2$CF$_3$), 3.48 (2H, s, C**H**$_2$C≡C), 2.34 (6H, s, 2 x ArC**H**$_3$); ^{13}C NMR (101 MHz, $CDCl_3$) δ 167.7 (2 × C), 138.2 (2 × C), 134.0 (C), 131.8 (2 × CH), 130.5 (CH), 128.3 (3 × CH), 125.6 (2 × CH), 123.0 (C), 122.7 (q, $J_{C\text{-}F}$ = 277.6 Hz, 2 × C), 84.6 (C), 83.7 (C), 62.5 (C), 61.5 (q, $J_{C\text{-}F}$ = 37.3 Hz, 2 × CH_2), 27.2 (CH_2), 21.60 (CH_3), 21.59 (CH_3); ^{19}F NMR (376 MHz, $CDCl_3$) δ −73.6 (t, *J* = 8.2 Hz, 6 × F); HRMS (ESI) exact mass calculated for $[C_{24}H_{20}F_6NaO_4]^+$ $[M+Na]^+$: 509.1158, found 509.1152.

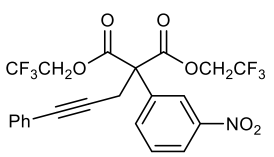

Bis(2,2,2-trifluoroethyl) 2-(3-nitrophenyl)-2-(3-phenylprop-2-yn-1-yl)malonate (1g). The title compound was prepared according to General Procedure D, using malonate **S7** (700 mg, 1.80 mmol), NaH (60% dispersion in mineral oil, 86.3 mg, 2.16 mmol), and alkynyl bromide **S12**[5] (75 wt. % in toluene, 935 μL, 3.60 mmol) in THF (8 mL) for 19 h, and purified by column chromatography (0% to 5% EtOAc/petroleum ether) to give a pale brown oil (575 mg, 63%). R_f = 0.26 (10% EtOAc/petroleum ether); IR 2360, 1757 (C=O), 1532, 1409, 1350, 1281, 1215, 1157, 1077, 651 cm^{-1}; ^{1}H NMR (400 MHz, $CDCl_3$) δ 8.57 (1H, t, *J* = 2.1 Hz, Ar**H**), 8.28 (1H, ddd, *J* = 8.3, 2.2, 0.9 Hz, Ar**H**), 7.87 (1H, ddd, *J* = 8.0, 2.0, 1.0 Hz, Ar**H**), 7.61 (1H, t, *J* = 8.1 Hz, Ar**H**), 7.32-7.24 (5H, m, Ar**H**), 4.64 (4H, q,

J = 8.1 Hz, 2 × C**H**$_2$CF$_3$), 3.57 (2H, s, C**H**$_2$C≡C); ^{13}C NMR (101 MHz, CDCl$_3$) δ 166.7 (2 × C), 148.3 (C), 135.8 (C), 134.4 (CH), 131.8 (2 × CH), 129.6 (CH), 128.7 (CH), 128.4 (2 × CH), 124.0 (CH), 123.6 (CH), 122.5 (q, J_{C-F} = 277.4 Hz, 2 × C), 122.3 (C), 122.1 (C), 85.9 (C), 82.0 (C), 62.1 (C), 61.9 (q, J_{C-F} = 37.4 Hz, 2 × CH$_2$), 27.0 (CH$_2$); ^{19}F NMR (376 MHz, CDCl$_3$) δ –73.7 (t, J = 8.1 Hz, 6 × F); HRMS (ESI) Exact mass calculated for $[C_{22}H_{15}F_6NNaO_6]^+$ $[M+Na]^+$: 526.0696, found 526.0685.

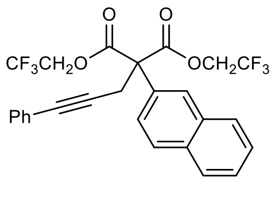

Bis(2,2,2-trifluoroethyl) 2-(naphthalen-2-yl)-2-(3-phenylprop-2-yn-1-yl)malonate (1h). The title compound was prepared according to General Procedure E, using malonate **S11** (1.00 g, 2.54 mmol), NaH (60% dispersion in mineral oil, 152 mg, 3.80 mmol), and alkynyl bromide **S12**[5] (80 wt. % solution in toluene, 1.24 mL, 5.07 mmol) in DMF (10 mL) for 18 h, and purified by column chromatography (0% to 2% EtOAc/petroleum ether) to give a brown oil (973 mg, 75%). R$_f$ = 0.40 (10 % EtOAc/petroleum ether); IR 3061, 1754 (C=O), 1410, 1281, 1214, 1158, 1081, 971, 756, 651 cm^{-1}; ^{1}H NMR (400 MHz, CDCl$_3$) δ 8.01 (1H, d, J = 2.1 Hz, Ar**H**), 7.90-7.84 (3H, m, Ar**H**), 7.62 (1H, dd, J = 8.7, 2.1 Hz, ArH), 7.56-7.49 (2H, m, Ar**H**), 7.31-7.22 (5H, m, Ar**H**), 4.66-4.60 (4H, m, 2 × C**H**$_2$CF$_3$), 3.63 (2H, s, C**H**$_2$C≡C); ^{13}C NMR (101 MHz, CDCl$_3$) δ 167.6 (2 × C), 133.1 (C), 133.0 (C), 131.8 (2 × CH), 131.4 (C), 128.6 (CH), 128.4 (CH), 128.34 (2 × CH), 128.30 (CH), 127.7 (CH), 127.4 (CH), 127.1 (CH), 126.7 (CH), 125.5 (CH), 122.8 (C), 122.7 (q, J_{C-F} = 277.8 Hz, 2 × C), 84.9 (C), 83.4 (C), 62.6 (C), 61.6 (q, J_{C-F} = 37.4 Hz, 2 × CH$_2$), 27.1 (CH$_2$); ^{19}F NMR (376 MHz, CDCl$_3$) δ −73.7 (t, J = 8.2 Hz, 6 × F); HRMS (ESI) exact mass calculated for $[C_{26}H_{18}F_6O_4]^+$ $[M+Na]^+$: 531.1001, found 531.1002.

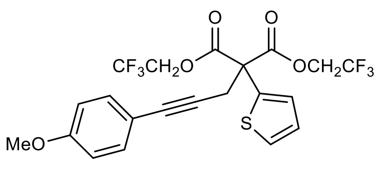

Bis(2,2,2-trifluoroethyl) 2-[3-(4-methoxyphenyl)prop-2-yn-1-yl]-2-(2-thienyl)malonate (1i). The title compound was prepared according to General Procedure D, using malonate **S8** (1.00 g, 2.85 mmol), NaH (60% dispersion in mineral oil, 137 mg, 3.42 mmol), and alkynyl bromide **S13**[6] (75 wt. % in toluene, 1.71 mL, 5.71 mmol) in THF (13 mL) for 18 h, and purified by column chromatography (0% to 2% EtOAc/petroleum ether) to give a colorless oil (850 mg, 60%). R$_f$ = 0.24 (5% EtOAc/petroleum ether); IR 2980, 1754 (C=O), 1605, 1456, 1286, 1236, 1155, 1008, 837, 709 cm^{-1}; ^{1}H NMR (400 MHz, CDCl$_3$) δ 7.38 (1H, dd, J = 5.2, 1.2 Hz, Ar**H**), 7.27-7.21 (2H, m, Ar**H**), 7.22 (1H, dd, J = 3.7, 1.2 Hz, Ar**H**), 7.03 (1H, dd, J = 5.2, 3.7 Hz, Ar**H**), 6.80-6.77 (2H, m, Ar**H**), 4.68-4.51 (4H, m, 2 × C**H**$_2$CF$_3$), 3.78 (3H, s, OC**H**$_3$), 3.51 (2H, s, C**H**$_2$C≡C); ^{13}C NMR (101 MHz, CDCl$_3$) δ 166.8 (2 × C), 159.7 (C), 136.1 (C), 133.2 (2 × CH), 127.6 (CH), 127.1 (CH), 126.5 (CH), 122.6 (q, J_{C-F} = 277.8 Hz, 2 × C), 114.9 (C), 114.0 (2 × CH),

85.1 (C), 81.2 (C), 61.7 (q, $J_{C\text{-}F}$ = 37.5 Hz, 2 × CH_2), 59.9 (C), 55.4 (CH_3), 29.0 (CH_2); ^{19}F NMR (376 MHz, $CDCl_3$) δ –73.6 (t, *J* = 8.1 Hz, 6 × F); HRMS (ESI) Exact mass calculated for $[C_{21}H_{16}F_6NaO_5S]^+$ $[M+Na]^+$: 517.0515, found 517.0515.

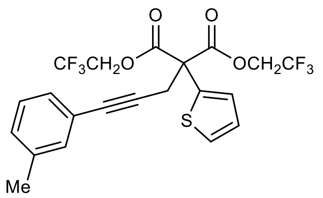

Bis(2,2,2-trifluoroethyl) 2-(2-thienyl)-2-[3-(3-methylphenyl)prop-2-yn-1-yl]malonate (1j). The title compound was prepared according to General Procedure D, using malonate **S8** (500 mg, 1.43 mmol), NaH (60% dispersion in mineral oil, 68.5 mg, 1.71 mmol), and alkynyl bromide **S14**[6] (72 wt. % in toluene, 829 μL, 2.86 mmol) in THF (6.5 mL) for 18 h, and purified by column chromatography (0% to 5% EtOAc/petroleum ether) to give a yellow oil (366 mg, 54%). R_f = 0.29 (10% EtOAc/petroleum ether); IR 2976, 1757 (C=O), 1410, 1282, 1211, 1156, 1060, 973, 784, 705 cm^{-1}; ^{1}H NMR (400 MHz, $CDCl_3$) δ 7.38 (1H, dd, *J* = 5.2, 1.2 Hz, Ar**H**), 7.22 (1H, dd, *J* = 3.7, 1.2 Hz, Ar**H**), 7.17-7.08 (4H, m, Ar**H**), 7.04 (1H, dd, *J* = 5.2, 3.7 Hz, Ar**H**), 4.68-4.52 (4H, m, 2 × C**H**$_2$CF$_3$), 3.53 (2H, s, C**H**$_2$C≡C), 2.29 (3H, s, C**H**$_3$); ^{13}C NMR (101 MHz, $CDCl_3$) δ 166.8 (2 × C), 138.0 (C), 136.0 (C), 132.3 (CH), 129.4 (CH), 128.9 (CH), 128.2 (CH), 127.7 (CH), 127.2 (CH), 126.6 (CH), 122.6 (t, $J_{C\text{-}F}$ = 277.5 Hz, 2 × C), 122.55 (C), 85.4 (C), 82.3 (C), 61.8 (q, $J_{C\text{-}F}$ = 37.7 Hz, 2 × CH_2), 59.9 (C), 29.0 (CH_2), 21.3 (CH_3); ^{19}F NMR (376 MHz, $CDCl_3$) δ –73.7 (td, *J* = 8.1, 2.8 Hz, 6 × F); HRMS (ESI) Exact mass calculated for $[C_{21}H_{16}F_6NaO_4S]^+$ $[M+Na]^+$: 501.0566, found: 501.0565.

1,3-Bis(2,2,2-trifluoroethyl)-2-[3-(4-chlorophenyl)prop-2-yn-1-yl]-2-(4-methoxyphenyl)propanedioate (1k)

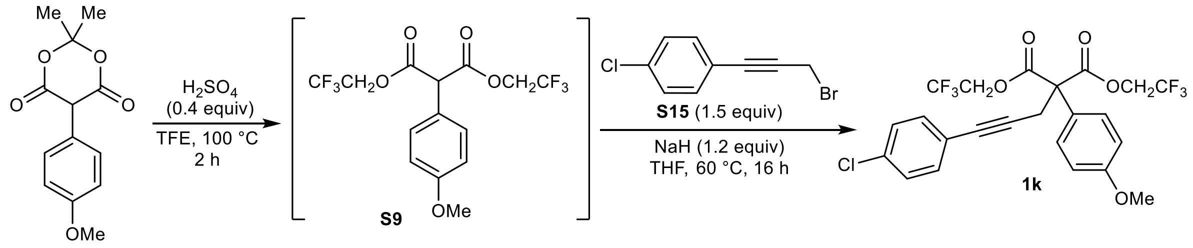

A microwave vial fitted with a stirrer bar was charged with 5-(4-methoxyphenyl)-2,2-dimethyl-1,3-dioxane-4,6-dione[11] (500 mg, 2.00 mmol), TFE (10 mL) and concentrated H_2SO_4 (43 µL, 0.80 mmol). The vial was then capped with a crimp capped PTFE seal and heated at 100 °C for 2 h. The reaction was cooled to room temperature and diluted with a mixture of Et_2O/petroleum ether (3:7, 100 mL). This solution was washed with aqueous Na_2CO_3 solution (5% w/w, 3 × 100 mL) and brine (50 mL). The organic layer was dried (Na_2SO_4) and concentrated under reduced pressure. The residual solvent was removed under high vacuum to leave the crude malonate ester **S9** (440 mg), which was used in the next step without further purification. A solution of this malonate ester **S9** in

THF (5 mL) was added to an ice-cooled suspension of NaH (60% dispersion in mineral oil, 56.7 mg, 1.42 mmol) in THF (10 mL). The resulting solution was warmed to room temperature and stirred for 30 min. Alkynyl bromide **S15**[5] (75 wt. % in toluene, 539 µL, 1.76 mmol) was added dropwise and the resulting solution was warmed to 60 °C and stirred for 16 h. The reaction was cooled to room temperature and quenched with a saturated aqueous NH_4Cl solution (50 mL). This mixture was extracted with EtOAc (3 × 20 mL) and the combined organic layers were dried (Na_2SO_4), filtered and concentrated under reduced pressure. The residue was purified by column chromatography (0% to 10% EtOAc/petroleum ether) to give title compound **1h** as a yellow oil (310 mg, 30% over two steps). R_f = 0.52 (10% EtOAc/petroleum ether); IR 2974, 1753 (C=O), 1515, 1490, 1282, 1157, 1087, 974, 827, 526 cm^{-1}; ^1H NMR (400 MHz, $CDCl_3$) δ 7.45-7.41 (2H, m, Ar**H**), 7.25-7.20 (4H, m, Ar**H**), 6.95-6.91 (2H, m, Ar**H**), 4.65-4.51 (4H, m, 2 × C**H**$_2$CF$_3$), 3.82 (3H, s, OC**H**$_3$), 3.48 (2H, s, C**H**$_2$C≡C); ^{13}C NMR (101 MHz, $CDCl_3$) δ 167.7 (2 × C), 159.9 (C), 134.4 (C), 133.0 (2 × CH), 129.2 (2 × CH), 128.7 (2 × CH), 125.7 (C), 122.6 (q, $J_{C\text{-}F}$ = 277.5 Hz, 2 × C), 121.4 (C), 114.0 (2 × CH), 84.5 (C), 83.6 (C), 61.7 (C), 61.5 (q, $J_{C\text{-}F}$ = 37.3 Hz, 2 × CH_2), 55.4 (CH_3), 26.9 (CH_2); ^{19}F NMR (376 MHz, $CDCl_3$) δ −73.6 (t, J = 8.2 Hz, 6 × F); HRMS (ESI) exact mass calculated for $[C_{23}H_{17}ClF_6NaO_5]^+$ $[M+Na]^+$: 545.0561, found: 545.0565.

1,3-Bis(2,2,2-trifluoroethyl)-2-(4-methoxyphenyl)-2-[3-(2-thienyl)prop-2-yn-1-yl]propanedioate (1l)

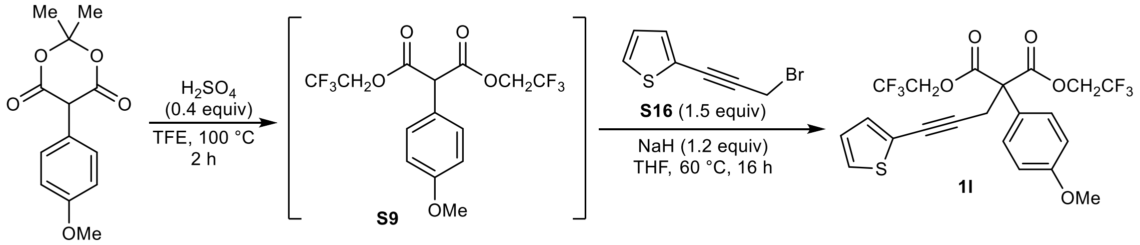

A microwave vial fitted with a stirrer bar was charged with 5-(4-methoxyphenyl)-2,2-dimethyl-1,3-dioxane-4,6-dione[11] (500 mg, 2.00 mmol), TFE (10 mL) and concentrated H_2SO_4 (43 µL, 0.80 mmol). The vial was then capped with a crimp capped PTFE seal and heated at 100 °C for 2 h. The reaction was cooled to room temperature and diluted with a mixture of Et_2O/petroleum ether (3:7, 100 mL). This solution was washed with aqueous Na_2CO_3 solution (5% w/w, 3 × 100 mL) and brine (50 mL). The organic layer was dried (Na_2SO_4) and concentrated under reduced pressure. The residual solvent was removed under high vacuum to leave the crude malonate ester **S9** (440 mg), which was used in the next step without further purification. A solution of this malonate ester **S9** in THF (5 mL) was added to an ice-cooled suspension of NaH (60% dispersion in mineral oil, 56.7 mg, 1.42 mmol) in THF (10 mL). The resulting solution was warmed to room temperature and stirred for 30 min. Alkynyl bromide **S16**[7] (75 wt. % in toluene, 0.473 mL, 1.76 mmol) was added

dropwise and the resulting solution was warmed to 60 °C and stirred for 16 h. The reaction was cooled to room temperature and quenched with a saturated aqueous NH_4Cl solution (50 mL). This mixture was extracted with EtOAc (3 × 20 mL) and the combined organic layers were dried (Na_2SO_4), filtered and concentrated under reduced pressure. The residue was purified by column chromatography (0% to 10% EtOAc/petroleum ether) to give title compound **1l** as a yellow oil (467 mg, 47% over two steps). R_f = 0.41 (10% EtOAc/petroleum ether); IR 2968, 1753 (C=O), 1611, 1514, 1282, 1155, 1079, 976, 828, 703 cm^{-1}; ^{1}H NMR (400 MHz, $CDCl_3$) δ 7.44-7.40 (2H, m, Ar**H**), 7.19 (1H, dd, *J* = 5.2, 1.2 Hz, Ar**H**), 7.08 (1H, dd, *J* = 3.6, 1.2 Hz, Ar**H**), 6.95-6.90 (3H, m, Ar**H**), 4.59 (4H, q, *J* = 8.1 Hz, 2 × C**H**$_2$CF$_3$), 3.82 (3H, s, OC**H**$_3$), 3.50 (2H, s, C**H**$_2$C≡C); ^{13}C NMR (101 MHz, $CDCl_3$) δ 167.7 (2 × C), 159.9 (C), 132.1 (CH), 129.2 (2 × CH), 127.01 (CH), 126.95 (CH), 125.8 (C), 122.8 (C), 122.6 (q, J_{C-F} = 277.4 Hz, 2 × C), 114.1 (2 × CH), 87.5 (C), 77.9 (C), 61.7 (C), 61.5 (q, J_{C-F} = 37.6 Hz, 2 × CH_2), 55.4 (CH_3), 27.3 (CH_2); ^{19}F NMR (376 MHz, $CDCl_3$) δ −73.6 (t, *J* = 8.2 Hz, 6 × F); HRMS (ESI) exact mass calculated for $[C_{21}H_{16}F_6NaO_5S]^+$ $[M+Na]^+$: 517.0515, found 517.0507.

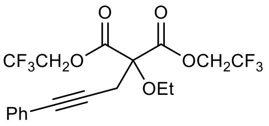

Bis(2,2,2-trifluoroethyl) 2-ethoxy-2-(3-phenylprop-2-yn-1-yl)malonate (1m). The title compound was prepared according to a slight modification of General Procedure D, in that the reaction temperature was 45 °C (rather than 60 °C), using malonate **S10b** (1.00 g, 3.20 mmol), NaH (60% dispersion in mineral oil, 154 mg, 3.84 mmol), and alkynyl bromide **S12**[5] (80 wt. % in toluene, 1.56 mL, 6.41 mmol) in THF (9 mL) at 45 ˚C for 18 h, and purified by column chromatography (0% to 5% EtOAc/petroleum ether) as a yellow oil (727 mg, 53%). R_f = 0.20 (10% EtOAc in petroleum ether); IR 1762 (C=O), 1411, 1282, 1220, 1160, 1116, 1082, 973, 757, 691 cm^{-1}; ^{1}H NMR (400 MHz, $CDCl_3$) δ 7.39-7.25 (5H, m, Ar**H**), 4.68-4.54 (4H, m, 2 × C**H**$_2$CF$_3$), 3.72 (2H, q, *J* = 7.0 Hz, OC**H**$_2$CH$_3$), 3.26 (2H, s, C**H**$_2$C≡C), 1.31 (3H, t, *J* = 7.0 Hz, OCH$_2$C**H**$_3$); ^{13}C NMR (101 MHz, $CDCl_3$) δ 166.0 (2 × C), 131.9 (2 × CH), 128.4 (CH), 128.3 (2 × CH), 122.9 (C), 122.6 (q, J_{C-F} = 277.4 Hz, 2 × C), 84.5 (C), 83.8 (C), 81.5 (C), 63.1 (CH_2), 61.4 (q, J_{C-F} = 37.4 Hz, 2 × CH_2), 26.2 (CH_2), 15.5 (CH_3); ^{19}F NMR (376 MHz, $CDCl_3$) δ −73.7 (t, *J* = 8.1 Hz, 6 × F); HRMS (ESI) Exact mass calculated for $[C_{18}H_{16}F_6NaO_5]^+$ $[M+Na]^+$: 449.0794, found: 449.0801.

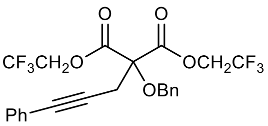

Bis(2,2,2-trifluoroethyl) 2-(benzyloxy)-2-(3-phenylprop-2-yn-1-yl)malonate (1n). The title compound was prepared according to a slight modification of General Procedure D, in that the reaction temperature was 45 °C (rather than 60 °C), using malonate **S10b** (1.00 g, 2.67 mmol), NaH (60% dispersion in

mineral oil, 128 mg, 3.20 mmol), and alkynyl bromide **S12**[5] (75 wt. % in toluene, 1.39 mL, 5.34 mmol) in THF (12 mL) for 18 h, and purified by column chromatography (0 to 2% EtOAc/petroleum ether) to give a colorless oil (511 mg, 39%). R_f = 0.26 (5% EtOAc/petroleum ether); IR 3034, 1762 (C=O), 1411, 1282, 1219, 1162, 1092, 1028, 973, 756 cm^{-1}; ^{1}H NMR (400 MHz, $CDCl_3$) δ 7.44-7.27 (10H, m, Ar**H**), 4.82 (2H, s, ArC**H**$_2$), 4.58 (4H, qd, J = 8.2, 1.0 Hz, 2 × C**H**$_2$CF$_3$), 3.35 (2H, s, C**H**$_2$C≡C); ^{13}C NMR (101 MHz, $CDCl_3$) δ 165.8 (2 × C), 136.8 (C), 131.9 (2 × CH), 128.5 (2 × CH), 128.44 (CH), 128.36 (2 × CH), 128.2 (CH), 128.0 (2 × CH), 122.9 (C), 122.5 (q, J_{C-F} = 277.4 Hz, 2 × C), 84.7 (C), 83.6 (C), 81.5 (C), 69.4 (CH_2), 61.5 (q, J_{C-F} = 37.5 Hz, 2 × CH_2), 26.6 (CH_2); ^{19}F NMR (376 MHz, $CDCl_3$) δ –73.6 (t, J = 8.1 Hz, 6 × F); HRMS (ESI) Exact mass calculated for $[C_{23}H_{18}F_6NaO_5]^+$ $[M+Na]^+$: 511.0951, found 511.0945.

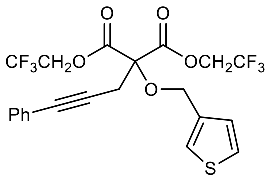

Bis(2,2,2-trifluoroethyl) 2-(3-phenylprop-2-yn-1-yl)-2-(thiophen-3-ylmethoxy)malonate (1o). The title compound was prepared according to a slight modification of General Procedure D, in that the reaction temperature was 45 °C (rather than 60 °C), using malonate **S10c** (1.00 g, 2.63 mmol), NaH (60% dispersion in mineral oil, 126 mg, 3.16 mmol), and alkynyl bromide **S12**[5] (80 wt. % in toluene, 1.28 mL, 5.26 mmol) in THF (9 mL) at 45 ˚C for 18 h, and purified by column chromatography (0% to 5% EtOAc/petroleum ether) as a yellow oil (546 mg, 42%). R_f = 0.19 (10% EtOAc/petroleum ether); IR 1762 (C=O), 1412, 1282, 1220, 1161, 1091, 1065, 973, 777, 691 cm^{-1}; ^{1}H NMR (400 MHz, $CDCl_3$) δ 7.38-7.27 (7H, m, Ar**H**), 7.15-7.14 (1H, m, Ar**H**), 4.83 (2H, s, ArC**H**$_2$O), 4.68-4.54 (4H, m, C**H**$_2$CF$_3$), 3.33 (2H, s, C**H**$_2$C≡C); ^{13}C NMR (101 MHz, $CDCl_3$) δ 165.8 (2 × C), 137.8 (C), 131.9 (2 × CH), 128.5 (CH), 128.4 (2 × CH), 127.5 (CH), 126.1 (CH), 123.6 (CH), 122.8 (C), 122.5 (q, J_{C-F} = 277.4 Hz, 2 × CF_3), 84.7 (C), 83.5 (C), 81.5 (C), 65.0 (CH_2), 61.5 (q, J_{C-F} = 37.5 Hz, 2 × CH_2), 26.6 (CH_2); ^{19}F NMR (376 MHz, $CDCl_3$) δ −73.6 (t, J = 8.1 Hz, 6 × F); HRMS (ESI) Exact mass calculated for $[C_{21}H_{16}F_6NaO_5S]$ $[M+Na]^+$: 517.0515, found: 517.0509.

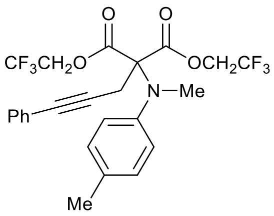

Bis(2,2,2-trifluoroethyl) 2-[methyl(4-methylphenyl)amino]-2-(3-phenylprop-2-yn-1-yl)malonate (1p). The title compound was prepared according to a slight modification of General Procedure E, in that the reaction temperature was 90 °C (rather than 80 °C), using malonate **S10d** (920 mg, 2.38 mmol), NaH (60% dispersion in mineral oil, 143 mg, 3.56 mmol), and alkynyl bromide **S12**[5] (80 wt. % in toluene, 1.16 mL, 5.4 mmol) in DMF (9 mL), for 3.5 h and purified by column chromatography (0% to 2% EtOAc/petroleum ether) to give a yellow oil (517 mg, 43%). R_f = 0.43 (15% EtOAc/petroleum ether); IR 1751 (C=O), 1411, 1280, 1159,

1071, 971, 837, 756, 691, 528 cm^{-1}; ^{1}H NMR (400 MHz, $CDCl_3$) δ 7.36-7.33 (2H, m, Ar**H**), 7.31-7.27 (3H, m, Ar**H**), 7.15-7.10 (4H, m, Ar**H**), .71-4.51 (4H, m, 2 × C**H**$_2$CF$_3$), 3.04 (3H, s, NC**H**$_3$), 3.02 (2H, s, C**H**$_2$C≡C), 2.32 (3H, s, ArC**H**$_3$); ^{13}C NMR (126 MHz, $CDCl_3$) δ 167.2 (2 × C), 144.9 (C), 136.0 (C), 131.8 (2 × CH), 129.8 (2 × CH), 128.33 (2 × CH), 128.26 (CH), 126.9 (2 × CH), 123.2 (C), 122.8 (q, $J_{C\text{-}F}$ = 277.4 Hz, 2 × C), 84.5 (C), 83.2 (C), 74.7 (C), 61.3 (q, $J_{C\text{-}F}$ = 37.4 Hz, 2 × CH_2), 41.1 (CH_3), 27.7 (CH_2), 21.1 (CH_3); ^{19}F NMR (376 MHz, $CDCl_3$) δ −73.5 (t, J = 8.3 Hz, 6 × F); HRMS (ESI) Exact mass calculated for $[C_{24}H_{21}F_6NNaO_4]^+$ $[M+Na]^+$: 524.1267, found: 524.1266.

Bis(2,2,2-trifluoroethyl) 2-(4-methoxyphenyl)-2-(5-methylhex-4-en-2-yn-1-yl)malonate (6)

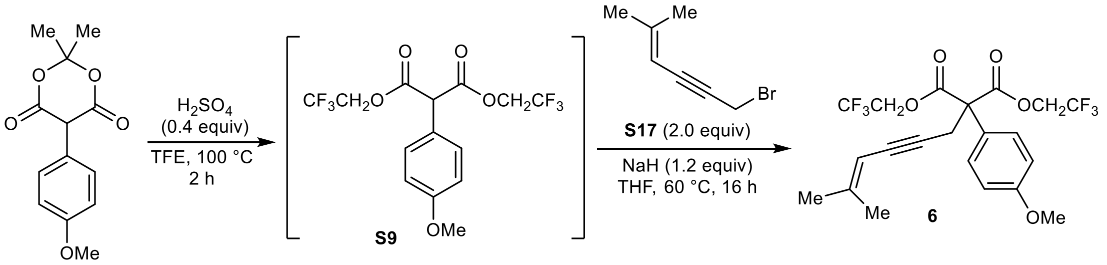

A microwave vial fitted with a stirrer bar was charged with 5-(4-methoxyphenyl)-2,2-dimethyl-1,3-dioxane-4,6-dione[11] (1.82 g, 7.27 mmol), TFE (35 mL) and concentrated H_2SO_4 (155 µL, 2.90 mmol). The vial was then capped with a crimp capped PTFE seal and heated at 100 °C for 2 h. The reaction was cooled to room temperature and diluted with a mixture of Et_2O/petroleum ether (3:7, 300 mL). This solution was washed with aqueous Na_2CO_3 solution (5% w/w, 3 × 300 mL) and brine (150 mL). The organic layer was dried (Na_2SO_4) and concentrated under reduced pressure. The residual solvent was removed under high vacuum to leave the crude malonate ester **S9** (1.60 g), which was used in the next step without further purification. A solution of this malonate ester **S9** in THF (18 mL) was added to an ice-cooled suspension of NaH (60% dispersion in mineral oil, 205 mg, 5.13 mmol) in THF (36 mL). The resulting solution was warmed to room temperature and stirred for 30 min. Alkynyl bromide **S17** (75 wt. % in toluene, 1.97 mL, 8.55 mmol) was added dropwise and the resulting solution was warmed to 60 °C and stirred for 16 h. The reaction was cooled to room temperature and quenched with saturated aqueous NH_4Cl solution (150 mL). This mixture was extracted with EtOAc (3 × 75 mL) and the combined organic layers were dried (Na_2SO_4), filtered and concentrated under reduced pressure. The residue was purified by column chromatography (0 to 5% EtOAc/petroleum ether) to give title compound **6** as a pale yellow oil (706 mg, 24% over two steps). R_f = 0.25 (5% EtOAc/petroleum ether); IR 2974, 1753 (C=O), 1611, 1515, 1410, 1256, 1156, 1031, 897, 545 cm^{-1}; ^{1}H NMR (400 MHz, $CDCl_3$) δ 7.44-7.40 (2H, m, Ar**H**), 6.92-6.88 (2H, m, Ar**H**), 5.14 (1H, dq, J = 2.5, 1.3 Hz, (CH$_3$)$_2$C=C**H**), 4.55 (4H, q, J = 8.0

Hz, 2 × C**H**$_2$CF$_3$), 3.81 (3H, s, OC**H**$_3$), 3.43 (2H, d, *J* = 2.2 Hz, C**H**$_2$C≡C), 1.74 (3H, s, =C(C**H**$_3$)$_2$), 1.73 (3H, s, =C(C**H**$_3$)$_2$); ^{13}C NMR (101 MHz, CDCl$_3$) δ 167.9 (2 × C), 159.8 (C), 149.0 (C), 129.3 (2 × CH), 125.8 (C), 122.7 (q, $J_{C\text{-}F}$ = 277.4 Hz, 2 × C), 113.9 (2 × CH), 104.8 (CH), 84.9 (C), 82.7 (C), 61.7 (C), 61.5 (q, $J_{C\text{-}F}$ = 37.4 Hz, 2 × CH$_2$), 55.4 (CH$_3$), 26.8 (CH$_2$), 24.8 (CH$_3$), 20.8 (CH$_3$); ^{19}F NMR (376 MHz, CDCl$_3$) δ –73.7 (t, *J* = 8.2 Hz, 6 × F); HRMS (ESI) Exact mass calculated for [C$_{21}$H$_{20}$F$_6$NaO$_5$]$^+$ [M+Na]$^+$: 489.1107, found 489.1101.

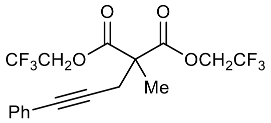

Bis(2,2,2-trifluoroethyl) 2-methyl-2-(3-phenylprop-2-yn-1-yl)malonate (1q). The title compound was prepared according to General Procedure D, using malonate **S3a** (1.00 g, 3.54 mmol), NaH (60% dispersion in mineral oil, 170 mg, 4.25 mmol), and alkynyl bromide **S12**[5] (1.38 g, 7.09 mmol) in THF (25 mL) for 18 h at room temperature, and purified by column chromatography (0% to 2% EtOAc/petroleum ether) to give a colorless oil (708 mg, 50%). R$_f$ = 0.44 (10% EtOAc/petroleum ether); IR 2979, 1755 (C=O), 1411, 1279, 1158, 1102, 975, 757, 691, 651 cm^{-1}; ^{1}H NMR (400 MHz, CDCl$_3$) δ 7.38-7.35 (2H, m, Ar**H**), 7.31-7.27 (3H, m, Ar**H**), 4.56 (4H, q, *J* = 8.2 Hz, 2 × C**H**$_2$CF$_3$), 3.09 (2H, s, C**H**$_2$C≡C), 1.71 (3H, s, C**H**$_3$); ^{13}C NMR (101 MHz, CDCl$_3$) δ 168.9 (2 × C), 131.8 (2 × CH), 128.4 (3 × CH), 131.8 (C), 122.9 (C), 122.7 (q, $J_{C\text{-}F}$ = 277.4 Hz, 2 × C), 84.4 (C), 82.9 (C), 61.3 (q, $J_{C\text{-}F}$ = 37.4 Hz, 2 × CH$_2$), 53.7 (C), 26.8 (CH$_2$), 20.0 (CH$_3$); ^{19}F NMR (376 MHz, CDCl$_3$) δ −73.9 (t, *J* = 8.2 Hz, 6 × F); HRMS (ESI) exact mass calculated for [C$_{17}$H$_{14}$F$_6$NaO$_4$]$^+$ [M+Na]$^+$: 419.0688, found 419.0698.

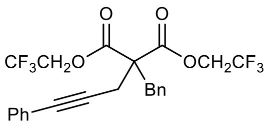

Bis(2,2,2-trifluoroethyl) 2-benzyl-2-(3-phenylprop-2-yn-1-yl)malonate (1r). The title compound was prepared according to General Procedure D, using malonate **S3b** (1.00 g, 2.79 mmol), NaH (60% dispersion in mineral oil, 134 mg, 3.35 mmol), and alkynyl bromide **S12**[5] (1.09 g, 5.59 mmol) in THF (12.5 mL) at room temperature for 18 h, and purified by column chromatography (0% to 2% EtOAc/petroleum ether) to give a colorless oil (630 mg, 48%). R$_f$ = 0.22 (2% EtOAc/petroleum ether); IR 3033, 1757 (C=O), 1411, 1281, 1237, 1155, 1086, 973, 756, 665 cm^{-1}; ^{1}H NMR (400 MHz, CDCl$_3$) δ 7.47-7.42 (2H, m, Ar**H**), 7.35-7.28 (6H, m, Ar**H**), 7.23-7.18 (2H, m, Ar**H**), 4.54 (4H, qd, *J* = 8.2, 1.7 Hz, 2 × C**H**$_2$CF$_3$), 3.53 (2H, s, ArC**H**$_2$), 2.99 (2H, s, C**H**$_2$C≡C); ^{13}C NMR (101 MHz, CDCl$_3$) δ 167.7 (2 × C), 134.4 (C), 131.9 (2 × CH), 129.9 (2 × CH), 128.9 (2 × CH), 128.54 (CH), 128.48 (2 × CH), 127.8 (CH), 122.9 (C), 122.7 (q, $J_{C\text{-}F}$ = 277.3 Hz, 2 × C), 85.3 (C), 83.0 (CH), 61.4 (q, $J_{C\text{-}F}$ = 37.4 Hz, 2 × CH$_2$), 58.8 (C), 37.6 (CH$_2$), 23.4 (CH$_2$); ^{19}F NMR (376 MHz, CDCl$_3$) δ –73.7 (t, *J* = 8.2 Hz, 6 × F); HRMS (ESI) Exact mass calculated for [C$_{23}$H$_{18}$F$_6$NaO$_4$]$^+$ [M+Na]$^+$: 495.1001, found 495.0983.

Phenyl *N*-(3-phenylprop-2-yn-1-yl)-*N*-tosylglycinate (10)

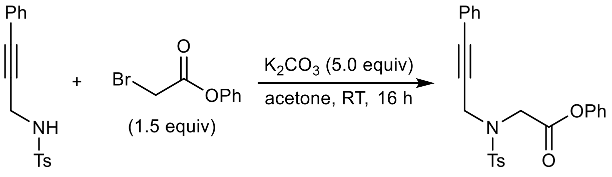

10

A mixture of 4-methyl-*N*-(3-phenylprop-2-yn-1-yl)benzenesulfonamide[13] (650 mg, 2.28 mmol), K_2CO_3 (1.57 g, 11.4 mmol), and phenyl bromoacetate (735 mg, 3.42 mmol) in acetone (10 mL) was stirred at room temperature for 16 h. The reaction mixture was filtered through a small bed of celite using acetone (20 mL) as eluent and the filtrate was concentrated under reduced pressure. The residue was purified by column chromatography (0% to 20% EtOAc/petroleum ether) to give title compound **10** as a white solid (859 mg, 90%). R_f = 0.32 (20% EtOAc/petroleum ether); IR 2970, 1769 (C=O), 1747, 1490, 1325, 1243, 1158, 920, 746, 524 cm^{-1}; m.p. 96-97 °C (CH_2Cl_2/ petroleum ether); ^{1}H NMR (400 MHz, $CDCl_3$) δ 7.84-7.82 (2H, m, Ar**H**), 7.39-7.19 (10H, Ar**H**), 7.10-7.07 (2H, Ar**H**), 4.56 (2H, s, C**H**$_2$C=O), 4.43 (2H, s, C**H**$_2$C≡C), 2.38 (3H, s, C**H**$_3$); ^{13}C NMR (101 MHz, $CDCl_3$) δ 167.2 (C), 150.3 (C), 144.1 (C), 136.0 (C), 131.8 (2 × CH), 129.9 (2 × CH), 129.6 (2 × CH), 128.8 (CH), 128.4 (2 × CH), 127.8 (2 × CH), 126.3 (CH), 122.0 (C), 121.4 (2 × CH), 86.5 (C), 81.4 (C), 47.5 (CH_2), 38.7 (CH_2), 21.6 (CH_3); HRMS (ESI) exact mass calculated for $[C_{24}H_{21}NNaO_4S]^+$ $[M+Na]^+$: 442.1083, found 442.1072.

3. General Procedure F: Enantioselective Nickel-Catalyzed Desymmetrization of Malonate Esters

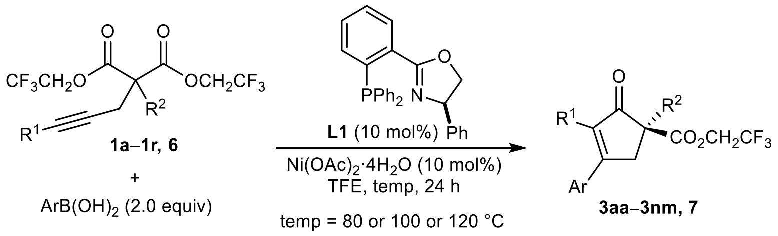

An oven-dried microwave vial fitted with a stirrer bar was charged with the appropriate substrate **1** (0.30 mmol), the arylboronic acid (0.60 mmol), $Ni(OAc)_2·4H_2O$ (7.5 mg, 0.03 mmol) and (*R*)-PhPhox (**L1**, 12.2 mg, 0.03 mmol). The vial was capped with a crimp cap PTFE seal and evacuated and back filled with argon (5 cycles). TFE (3 mL) which had been freshly degassed (using 5 freeze-pump-thaw cycles) was added under argon flow, the septum was resealed with a layer of vacuum grease, and the contents were stirred at room temperature for 10 min and then at the specified temperature (80 or 100 or 120 °C) for 24 h. The reaction was cooled to room temperature, diluted with EtOAc (5 mL) and washed with brine (10 mL). The aqueous layer was extracted with EtOAc (5 mL). The combined organic layers were dried (Na_2SO_4), filtered and concentrated under reduced

pressure. The residue was purified by column chromatography using EtOAc/*n*-pentane to give the cyclopent-2-enone **3** or **7**.

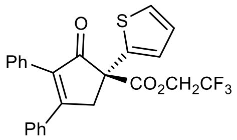

2,2,2-Trifluoroethyl (*R*)-2-oxo-3,4-diphenyl-1-(2-thienyl)cyclopent-3-ene-1-carboxylate (3aa). The title compound was prepared according to General Procedure F, using malonate ester **1a** (139.3 mg, 0.30 mmol) and phenylboronic acid (73.0 mg, 0.60 mmol) at 80 °C, and purified by column chromatography (10% EtOAc/*n*-pentane) to give a pale yellow oil (108.0 mg, 82%). R_f = 0.15 (7% EtOAc/petroleum ether); IR 3057, 1764 (C=O), 1702 (C=O), 1622, 1407, 1156, 965, 841, 693, 640 cm^{-1}; $[\alpha]_D^{23}$ –36.0 (*c* 1.00, $CHCl_3$); ^1H NMR (400 MHz, $CDCl_3$) δ 7.41-7.30 (10H, m, Ar**H**), 7.26-7.23 (2H, m, Ar**H**), 7.04 (1H, dd, *J* = 5.2, 3.7 Hz, Ar**H**), 4.66-4.47 (2H, m, C$\mathbf{H}_2$$CF_3$), 4.09 (1H, d, *J* = 18.1 Hz, =CC$\mathbf{H}_a$$H_b$), 3.68 (1H, d, *J* = 18.1 Hz, =CC$H_a$$\mathbf{H}_b$); ^{13}C NMR (101 MHz, $CDCl_3$) δ 198.8 (C), 168.6 (C), 166.2 (C), 139.1 (C), 136.6 (C), 134.5 (C), 131.4 (C), 130.8 (CH), 129.7 (2 × CH), 128.8 (2 × CH), 128.7 (2 × CH), 128.5 (CH), 128.4 (2 × CH), 126.93 (CH), 126.85 (CH), 125.9 (CH), 122.7 (q, $J_{C\text{-}F}$ = 277.9 Hz, C), 61.6 (q, $J_{C\text{-}F}$ = 37.0 Hz, CH_2), 59.8 (C), 44.4 (CH_2); ^{19}F NMR (376 MHz, $CDCl_3$) δ –73.7 (t, *J* = 8.3 Hz, 3 × F); HRMS (ESI) Exact mass calculated for $[C_{24}H_{17}F_3NaO_3S]^+$ $[M+Na]^+$: 465.0743, found 465.0746. Enantiomeric excess was determined by HPLC using a Chiralpak AD-H column (93:7 *iso*-hexane:*i*-PrOH, 1.0 mL/min, 230 nm, 25 °C); t_r (minor) = 15.7 min, t_r (major) = 17.8 min, 94% ee.

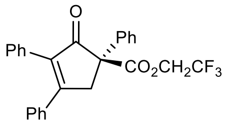

2,2,2-Trifluoroethyl (*R*)-2-oxo-1,3,4-triphenylcyclopent-3-ene-1-carboxylate (3ba). The title compound was prepared according to General Procedure F, using malonate ester **1b** (137.5 mg, 0.30 mmol) and phenylboronic acid (73.1 mg, 0.60 mmol) at 80 °C, and purified by column chromatography (0% to 10% EtOAc/petroleum ether) to give a pale yellow oil (127.2 mg, 97%). R_f = 0.3 (10% EtOAc/petroleum ether); IR 3058, 1763 (C=O), 1700 (C=O), 1445, 1407, 1281, 1153, 908, 731, 693 cm^{-1}; $[\alpha]_D^{20}$ −92.3 (*c* 0.52, $CHCl_3$); ^1H NMR (400 MHz, $CDCl_3$) δ 7.50-7.47 (2H, m, Ar**H**), 7.42-7.26 (13H, m, Ar**H**), 4.58 (2H, qd, *J* = 8.3, 1.8 Hz, C$\mathbf{H}_2$$CF_3$), 4.15 (1H, d, *J* = 18.1 Hz, =CC$\mathbf{H}_a$$H_b$), 3.53 (1H, d, *J* = 18.1 Hz, =CC$H_a$$\mathbf{H}_b$); ^{13}C NMR (101 MHz, $CDCl_3$) δ 199.9 (C), 169.4 (C), 166.0 (C), 138.1 (C), 137.5 (C), 134.6 (C), 131.7 (C), 130.7 (CH), 129.7 (2 × CH), 128.9 (2 × CH), 128.7 (4 × CH), 128.5 (CH), 128.4 (2 × CH), 128.1 (CH), 127.6 (2 × CH), 122.8 (q, $J_{C\text{-}F}$ = 277.7 Hz, C), 63.2 (C), 61.3 (q, $J_{C\text{-}F}$ = 36.8 Hz, CH_2), 43.9 (CH_2); ^{19}F NMR (376 MHz, $CDCl_3$) δ −73.6 (t, *J* = 8.3 Hz, 3 × F); HRMS (ESI) exact mass calculated for $[C_{26}H_{19}F_3NaO_3]^+$ $[M+Na]^+$: 459.1178, found: 459.1171.

Enantiomeric excess was determined by HPLC with Chiralpak AD-H column (95:5 *iso*-hexane:*i*-PrOH, 1.0 mL/min, 254 nm, 25 °C); t_r (major) = 22.4 min, t_r (minor) = 26.5 min, 82% ee.

Gram-Scale Reaction:

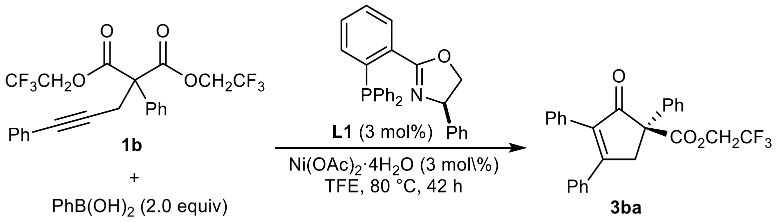

An oven-dried microwave vial fitted with a stirrer bar was charged with malonate ester **1a** (1.375 g, 3.00 mmol), the phenylboronic acid (731 mg, 6.0 mmol), $Ni(OAc)_2·4H_2O$ (22.4 mg, 0.09 mmol) and (*R*)-PhPhox (**L1**, 36.4 mg, 0.09 mmol). The vial was capped with a crimp cap PTFE seal and evacuated and back filled with argon (5 cycles). TFE (7.5 mL) which had been freshly degassed (using 5 freeze-pump-thaw cycles) was added under argon flow, the septum resealed with a layer of vacuum grease, and the contents were stirred at room temperature for 10 min and then at 80 °C for 42 h. The reaction was cooled to room temperature, diluted with EtOAc (50 mL) and washed with brine (100 mL). The aqueous layer was extracted with EtOAc (50 mL). The combined organic layers were dried (Na_2SO_4), filtered and concentrated under reduced pressure. The residue was purified by column chromatography (0% to 10% EtOAc/*n*-pentane) to give the title compound **3ba** as a pale yellow oil (1.097 g, 84%). Enantiomeric excess was determined by HPLC with Chiralpak AD-H column (95:5 *iso*-hexane:*i*-PrOH, 1.0 mL/min, 254 nm, 25 °C); t_r (major) = 22.3 min, t_r (minor) = 27.0 min, 80% ee.

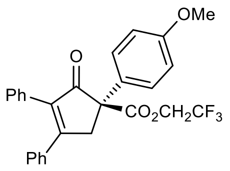

2,2,2-Trifluoroethyl (*R*)-1-(4-methoxyphenyl)-2-oxo-3,4-diphenylcyclopent-3-ene-1-carboxylate (3ca). The title compound was prepared according to General Procedure F, using malonate ester **1c** (146.5 mg, 0.30 mmol) and phenylboronic acid (73.2 mg, 0.60 mmol) at 100 °C, and purified by column chromatography (10% EtOAc/petroleum ether) to give a yellow oil (137.7 mg, 98%). R_f = 0.51 (20% EtOAc/petroleum ether); IR 2933, 1762 (C=O), 1699 (C=O), 1611, 1512, 1282, 1252, 1152, 1031, 694 cm^{-1}; $[\alpha]_D^{23}$ −71.0 (*c* 0.62, $CHCl_3$); ^1H NMR (400 MHz, $CDCl_3$) δ 7.46-7.42 (2H, m, Ar**H**), 7.39-7.25 (10H, m, Ar**H**), 6.95-6.91 (2H, m, Ar**H**), 4.57 (2H, qd, *J* = 8.3, 1.7 Hz, C**H**$_2$CF$_3$), 4.10 (1H, d, *J* = 18.1 Hz, =CC**H**$_a$H$_b$), 3.81 (3H, s, OC**H**$_3$), 3.53 (1H, d, *J* = 18.1 Hz, =CCH$_a$**H**$_b$); ^{13}C NMR (101 MHz, $CDCl_3$) δ 200.2 (C), 169.6 (C), 165.9 (C), 159.3 (C), 137.5 (C), 134.7 (C), 131.7 (C), 130.6 (CH), 129.8 (C), 129.7 (2 × CH), 128.8 (2 × CH), 128.70 (2 × CH), 128.68 (2 × CH), 128.42 (CH), 128.38 (2 × CH), 122.8 (q, J_{C-F} = 277.9 Hz, C), 114.3 (2 × CH), 62.4 (C), 61.3 (q, J_{C-F} = 36.7 Hz, CH_2), 55.4 (CH_3), 43.8 (CH_2); ^{19}F NMR (376 MHz, $CDCl_3$) δ −73.6 (td, *J* = 8.3, 2.3 Hz,

3 × F); HRMS (ESI) exact mass calculated for $[C_{27}H_{21}F_3NaO_4]^+$ $[M+Na]^+$: 489.1284, found: 489.1282. Enantiomeric excess was determined by HPLC with Chiralpak AD-H column (90:10 *iso*-hexane:*i*-PrOH, 1.0 mL/min, 254 nm, 25 °C); t_r (minor) = 28.8 min, t_r (major) = 30.7 min, 88% ee.

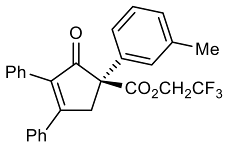

2,2,2-Trifluoroethyl (*R*)-2-oxo-3,4-diphenyl-1-(3-methylphenyl)cyclopent-3-ene-1-carboxylate (3da). The title compound was prepared according to General Procedure F, using malonate ester **1d** (141.7 mg, 0.30 mmol) and phenylboronic acid (73.2 mg, 0.60 mmol) at 80 °C, and purified by column chromatography (10% EtOAc/petroleum ether) to give a white solid (129.7 mg, 96%). R_f = 0.32 (10% EtOAc/petroleum ether); m.p. 93-94°C (Et_2O/*n*-pentane); IR 3057, 1762 (C=O), 1700 (C=O), 1623, 1351, 1282, 1157, 887, 729, 693 cm^{-1}; $[\alpha]_D^{23}$ −90.5 (*c* 0.84, $CHCl_3$); ^1H NMR (400 MHz, $CDCl_3$) δ 7.39-7.24 (13H, m, Ar**H**), 7.17-7.14 (1H, m, Ar**H**), 4.59 (2H, qd, *J* = 8.3, 3.8 Hz, C**H**$_2$CF$_3$), 4.14 (1H, d, *J* = 18.2 Hz, =CC**H**$_a$H$_b$), 3.52 (1H, d, *J* = 18.2 Hz, =CCH$_a$**H**$_b$), 2.37 (3H, s, ArC**H**$_3$); ^{13}C NMR (101 MHz, $CDCl_3$) δ 199.9 (C), 169.4 (C), 166.0 (C), 138.7 (C), 138.1 (C), 137.5 (C), 134.7 (C), 131.7 (C), 130.7 (CH), 129.7 (2 × CH), 128.8 (2 × CH), 128.7 (4 × CH), 128.45 (CH), 128.42 (2 × CH), 128.3 (CH), 124.5 (CH), 122.8 (q, J_{C-F} = 278.0 Hz, C), 63.3 (C), 61.3 (q, J_{C-F} = 36.8 Hz, CH_2), 44.0 (CH_2), 21.7 (CH_3); ^{19}F NMR (376 MHz, $CDCl_3$) δ −73.5 (t, *J* = 8.3 Hz, 3 × F); HRMS (ESI) exact mass calculated for $[C_{27}H_{21}F_3NaO_3]^+$ $[M+Na]^+$: 473.1335, found: 473.1348. Enantiomeric excess was determined by HPLC with Chiralpak AD-H column (98:2 *iso*-hexane:EtOH, 1.0 mL/min, 254 nm, 25 °C); t_r (minor) = 31.8 min, t_r (major) = 36.0 min, 87% ee.

Recrystallization of **3da** from Et_2O/pentane gave crystals that were suitable for X-ray crystallography:

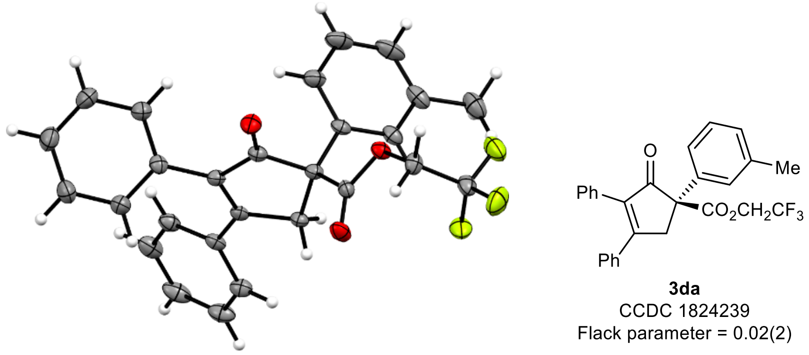

3da
CCDC 1824239
Flack parameter = 0.02(2)

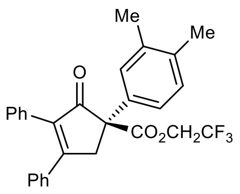

2,2,2-Trifluoroethyl (*R*)-1-(3,4-dimethylphenyl)-2-oxo-3,4-diphenylcyclopent-3-ene-1-carboxylate (3ea). The title compound was prepared according to General Procedure F, using malonate ester **1e** (145.9 mg, 0.30 mmol) and phenylboronic acid (73.1 mg, 0.60 mmol) at 80 °C, and purified by column chromatography (5% to 10% EtOAc/*n*-pentane) to give a yellow oil (104.2 mg,

75%). R_f = 0.17 (10% EtOAc/petroleum ether); $[\alpha]_D^{25}$ −92.0 (*c* 1.00, $CHCl_3$); IR 1762 (C=O), 1700 (C=O), 1444, 1351, 1281, 1156, 972, 730, 693, 476 cm^{-1}; ^{1}H NMR (400 MHz, $CDCl_3$) δ 7.39-7.26 (11H, m, Ar**H**), 7.23-7.15 (2H, m, Ar**H**), 4.59 (2H, q, *J* = 8.3 Hz, C**H**$_2$CF$_3$), 4.12 (1H, d, *J* = 18.2 Hz, =CC**H**$_a$CH$_b$), 3.54 (1H, d, *J* = 18.2 Hz, =CC**H**$_a$CH$_b$), 2.29 (3H, s, ArC**H**$_3$), 2.27 (3H, s, ArC**H**$_3$); ^{13}C NMR (101 MHz, $CDCl_3$) δ 200.1 (C), 169.6 (C), 165.9 (C), 137.5 (C), 137.2 (C), 136.6 (C), 135.5 (C), 134.7 (C), 131.7 (C), 130.6 (CH), 130.1 (CH), 129.7 (2 × CH), 128.8 (CH), 128.7 (4 × CH), 128.4 (2 × CH), 124.9 (CH), 122.8 (q, $J_{C\text{-}F}$ = 277.9 Hz, C), 62.9 (C), 61.3 (q, $J_{C\text{-}F}$ = 36.8 Hz, CH$_2$), 43.9 (CH$_2$), 20.1 (CH$_3$), 19.5 (CH$_3$); ^{19}F NMR (376 MHz, $CDCl_3$) δ −73.5 (t, *J* = 8.3 Hz, 3 × F); HRMS (ESI) Exact mass calculated for $[C_{28}H_{23}F_3NaO_3]^+$ $[M+Na]^+$: 487.1491, found: 487.1493. Enantiomeric excess was determined by HPLC with Chiralpak AD-H column (95:5 *iso*-hexane:EtOH, 1.0 mL/min, 254 nm, 25 °C); t_r (major) = 28.2 min, t_r (minor) = 39.3 min, 92% ee.

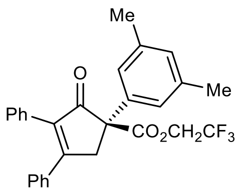

2,2,2-Trifluoroethyl (*R*)-1-(3,5-dimethylphenyl)-2-oxo-3,4-diphenylcyclopent-3-ene-1-carboxylate (3fa). The title compound was prepared according to General Procedure F, using malonate ester **1f** (145.9 mg, 0.30 mmol) and phenylboronic acid (73.1 mg, 0.60 mmol) at 80 °C, and purified by column chromatography (5% EtOAc/*n*-pentane) to give a yellow oil (115.1 mg, 83%). R_f = 0.11 (5% EtOAc/petroleum ether); IR 2920, 1763 (C=O), 1700 (C=O), 1599, 1351, 1282, 1154, 730, 693, 642 cm^{-1}; $[\alpha]_D^{23}$ −118.1 (*c* 1.00, $CHCl_3$); ^{1}H NMR (400 MHz, $CDCl_3$) δ 7.39-7.33 (6H, m, Ar**H**), 7.32-7.27 (3H, m, Ar**H**), 7.26-7.24 (1H, m, Ar**H**), 7.05-7.04 (2H, m, Ar**H**), 6.97-6.96 (1H, m, Ar**H**), 4.65-4.51 (2H, m, C**H**$_2$CF$_3$), 4.11 (1H, d, *J* = 18.2 Hz, =C**H**$_a$H$_b$), 3.50 (1H, d, *J* = 18.2 Hz, =CH$_a$**H**$_b$), 2.32 (6H, s, 2 × ArC**H**$_3$); ^{13}C NMR (101 MHz, $CDCl_3$) δ 200.0 (C), 169.5 (C), 166.0 (C), 138.5 (2 × C), 138.1 (C), 137.6 (C), 134.7 (C), 131.7 (C), 130.6 (CH), 129.73 (2 × CH), 129.69 (CH), 128.68 (2 × CH), 128.67 (2 × CH), 128.4 (3 × CH), 125.3 (2 × CH), 122.8 (q, $J_{C\text{-}F}$ = 277.9 Hz, CF$_3$), 63.3 (C), 61.2 (q, $J_{C\text{-}F}$ = 36.8 Hz, CH$_2$), 44.1 (CH$_2$), 21.5 (2 × CH$_3$); ^{19}F NMR (376 MHz, $CDCl_3$) δ −73.5 (t, *J* = 8.3 Hz, 3 × F); HRMS (ESI) exact mass calculated for $[C_{28}H_{23}F_3NaO_3]^+$ $[M+Na]^+$: 487.1491, found: 487.1504. Enantiomeric excess was determined by HPLC with Chiralpak AD-H column (98:2 *iso*-hexane:EtOH, 1.0 mL/min, 230 nm, 25 °C); t_r (major) = 11.1 min, t_r (minor) = 18.8 min, 92% ee.

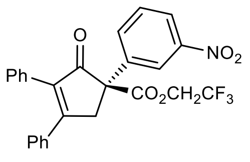

2,2,2-Trifluoroethyl (*R*)-1-(3-nitrophenyl)-2-oxo-3,4-diphenylcyclopent-3-ene-1-carboxylate (3ga). The title compound was prepared according to General Procedure F, using malonate ester **1g** (151.0 mg, 0.30 mmol) and phenylboronic acid (73.1 mg, 0.60 mmol) at 80 °C, and purified by column chromatography (10%

EtOAc/*n*-pentane) to give a colorless oil (138.0 mg, 96%). R_f = 0.22 (15% EtOAc/petroleum ether); IR 3085, 1757 (C=O), 1698 (C=O), 1622, 1528, 1347, 1280, 1154, 968, 692 cm^{-1}; $[\alpha]_D^{20}$ –80.0 (*c* 1.00, $CHCl_3$); ^1H NMR (400 MHz, $CDCl_3$) δ 8.41 (1H, t, *J* = 2.1 Hz, Ar**H**), 8.22 (1H, ddd, *J* = 8.2, 2.4, 1.0 Hz, Ar**H**), 7.97-7.94 (1H, m, Ar**H**), 7.60 (1H, t, *J* = 8.1 Hz, Ar**H**), 7.43-7.31 (8H, m, Ar**H**), 7.29-7.24 (2H, m, Ar**H**), 4.58 (2H, q, *J* = 8.2 Hz, C**H**$_2$CF$_3$), 4.15 (1H, d, *J* = 18.1 Hz, =CC**H**$_a$H$_b$), 3.57 (1H, d, *J* = 18.1 Hz, =CCH$_a$**H**$_b$); ^{13}C NMR (101 MHz, $CDCl_3$) δ 198.9 (C), 168.5 (C), 166.4 (C), 148.5 (C), 139.4 (C), 137.6 (C), 134.3 (CH), 134.2 (C), 131.2 (C)**,** 131.1 (CH), 129.8 (CH), 129.6 (2 × CH), 128.9 (2 × CH), 128.84 (2 × CH), 128.75 (CH), 128.4 (2 × CH), 123.2 (CH), 123.0 (CH), 122.6 (q, J_{C-F} = 277.4 Hz, C), 62.5 (C), 61.7 (q, J_{C-F} = 37.1 Hz, CH_2), 43.2 (CH_2); ^{19}F NMR (376 MHz, $CDCl_3$) δ –73.7 (t, *J* = 8.2 Hz, 3 × F); HRMS (ESI) Exact mass calculated for $[C_{26}H_{18}F_3NNaO_5]^+$ $[M+Na]^+$: 504.1029, found 504.1023. Enantiomeric excess was determined by HPLC with Chiralpak AD-H column (90:10 *iso*-hexane:*i*-PrOH, 1.0 mL/min, 230 nm, 25 °C); t_r (minor) = 16.9 min, t_r (major) = 20.7 min, 77% ee.

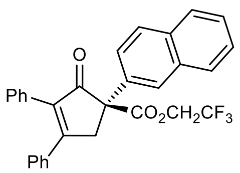

2,2,2-Trifluoroethyl (*R*)-1-(naphthalen-2-yl)-2-oxo-3,4-diphenylcyclopent-3-ene-1-carboxylate (3ha). The title compound was prepared according to General Procedure F, using malonate ester **1h** (152.5 mg, 0.30 mmol) and phenylboronic acid (73.1 mg, 0.60 mmol) at 80 °C, and purified by column chromatography (0% to 10% EtOAc/*n*-pentane) to give a yellow oil (127.2 mg, 87%). R_f = 0.17 (10% EtOAc/petroleum ether); $[\alpha]_D^{25}$ −76.0 (*c* 1.00 $CHCl_3$); IR 1761 (C=O), 1698 (C=O), 1407, 1351, 1280, 1154, 971, 816, 693, 475 cm^{-1}; ^1H NMR (400 MHz, $CDCl_3$) δ 7.98 (1H, d, *J* = 2.0 Hz, Ar**H**), 7.91 (1H, d, *J* = 8.7 Hz, Ar**H**), 7.88-7.83 (2H, m, Ar**H**), 7.58 (1H, dd, *J* = 8.7, 2.0 Hz, Ar**H**), 7.53-7.49 (2H, m, Ar**H**), 7.43-7.36 (6H, m, Ar**H**), 7.34-7.30 (4H, m, Ar**H**), 4.67-4.58 (2H, m, C**H**$_2$CF$_3$), 4.24 (1H, d, *J* = 18.2 Hz, =CC**H**$_a$H$_b$), 3.64 (1H, d, *J* = 18.2 Hz, =CCH$_a$**H**$_b$); ^{13}C NMR (101 MHz, $CDCl_3$) δ 199.9 (C), 169.4 (C), 166.1 (C), 137.6 (C), 135.3 (C), 134.6 (C), 133.2 (C), 132.8 (C), 131.6 (C), 130.7 (CH), 129.7 (2 × CH), 128.8 (CH), 128.73 (2 × CH), 128.71 (2 × CH), 128.50 (CH), 128.47 (CH), 128.4 (2 × CH), 127.6 (CH), 126.64 (CH), 126.55 (CH), 126.3 (CH), 125.7 (CH), 122.8 (q, J_{C-F} = 277.6 Hz, C), 63.4 (C), 61.4 (q, J_{C-F} = 36.9 Hz, CH_2), 43.9 (CH_2); ^{19}F NMR (376 MHz, $CDCl_3$) δ −73.5 (t, *J* = 8.3 Hz, 3 × F); HRMS (ESI) Exact mass calculated for $[C_{30}H_{21}F_3NaO_3]^+$ $[M+Na]^+$: 509.1335, found: 509.1338. Enantiomeric excess was determined by HPLC with Chiralpak AD-H column (95:5 *iso*-hexane: *i*-PrOH, 1.0 mL/min, 254 nm, 25 °C); t_r (major) = 47.1 min, t_r (minor) = 50.8 min, 94% ee.

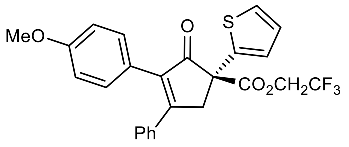

2,2,2-Trifluoroethyl (*R*)-3-(4-methoxyphenyl)-2-oxo-4-phenyl-1-(2-thienyl)cyclopent-3-ene-1-carboxylate (3ia). The title compound was prepared according to General Procedure F, using malonate ester **1i** (148.3 mg, 0.30 mmol) and phenylboronic acid (73.1 mg, 0.60 mmol) at 80 °C, and purified by column chromatography (10% EtOAc/*n*-pentane) to give a pale yellow oil (136.0 mg, 96%). R_f = 0.26 (9% EtOAc/petroleum ether); IR 2934, 1764 (C=O), 1703 (C=O), 1602, 1510, 1283, 1159, 1031, 837, 695 cm^{-1}; $[\alpha]_D^{22}$ –36.0 (*c* 1.00, $CHCl_3$); ^1H NMR (400 MHz, $CDCl_3$) δ 7.41-7.29 (7H, m, Ar**H**), 7.21-7.17 (2H, m, Ar**H**), 7.03 (1H, dd, *J* = 5.1, 3.7 Hz, Ar**H**), 6.89-6.85 (2H, m, Ar**H**), 4.65-4.46 (2H, m, C$\mathbf{H}_2$$CF_3$), 4.05 (1H, d, *J* = 18.0 Hz, =CC$\mathbf{H}_a$$H_b$), 3.81 (3H, s, OC$\mathbf{H}_3$), 3.65 (1H, d, *J* = 18.0 Hz, =CC$H_a$$\mathbf{H}_b$); ^{13}C NMR (101 MHz, $CDCl_3$) δ 199.1 (C), 168.6 (C), 165.3 (C), 159.8 (C), 139.2 (C), 136.1 (C), 134.8 (C), 131.0 (2 × CH), 130.6 (CH), 128.8 (2 × CH), 128.3 (2 × CH), 126.9 (CH), 126.8 (CH), 125.9 (CH), 123.5 (C), 122.7 (q, $J_{C\text{-}F}$ = 277.3 Hz, C), 114.2 (2 × CH), 61.6 (q, $J_{C\text{-}F}$ = 37.0 Hz, CH_2), 59.8 (C), 55.4 (CH_3), 44.4 (CH_2); ^{19}F NMR (376 MHz, $CDCl_3$) δ –73.7 (t, *J* = 8.2 Hz, 3 × F); HRMS (ESI) Exact mass calculated for $[C_{25}H_{19}F_3NaO_4S]^+$ $[M+Na]^+$: 495.0848, found 495.0850. Enantiomeric excess was determined by HPLC using a Chiralpak AD-H column (95:5 *iso*-hexane:*i*-PrOH, 1.0 mL/min, 230 nm, 25 °C); t_r (minor) = 31.7 min, t_r (major) = 40.5 min, 94% ee.

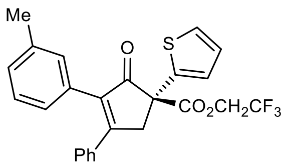

2,2,2-Trifluoroethyl (*R*)-2-oxo-4-phenyl-1-(2-thienyl)-3-(3-methylphenyl)cyclopent-3-ene-1-carboxylate (3ja). The title compound was prepared according to General Procedure F, using malonate ester **1j** (143.5 mg, 0.30 mmol) and phenylboronic acid (73.1 mg, 0.60 mmol) at 80 °C, and purified by column chromatography (5% EtOAc/*n*-pentane) to give a yellow oil (79.2 mg, 58%). R_f = 0.25 (10% EtOAc/petroleum ether); IR 2923, 1765 (C=O), 1704 (C=O), 1622, 1483, 1281, 1234, 1155, 841, 693 cm^{-1}; $[\alpha]_D^{24}$ –28.0 (*c* 1.00, $CHCl_3$); ^1H NMR (500 MHz, $CDCl_3$) δ 7.41-7.37 (3H, m, Ar**H**), 7.34-7.30 (4H, m, Ar**H**), 7.22 (1H, t, *J* = 7.6 Hz, Ar**H**), 7.16-7.13 (1H, m, Ar**H**), 7.09-7.08 (1H, m, Ar**H**), 7.04 (1H, dd, *J* = 5.1, 3.7 Hz, Ar**H**), 6.99 (1H, dd, *J* = 7.6, 1.5 Hz, Ar**H**), 4.61 (1H, dq, *J* = 12.7, 8.3 Hz, C$\mathbf{H}_a$$H_b$$CF_3$), 4.51 (1H, dq, *J* = 12.7, 8.3 Hz, C$H_a$$\mathbf{H}_b$$CF_3$), 4.08 (1H, d, *J* = 18.0 Hz, =CC$\mathbf{H}_c$$H_d$), 3.67 (1H, d, *J* = 17.9 Hz, =CC$H_c$$\mathbf{H}_d$), 2.31 (3H, s, C$\mathbf{H}_3$); ^{13}C NMR (126 MHz, $CDCl_3$) δ 198.9 (C), 168.6 (C), 165.9 (C), 139.1 (C), 138.4 (C), 136.8 (C), 134.5 (C), 131.4 (C), 130.8 (CH), 130.1 (CH), 129.3 (CH), 128.7 (2 × CH), 128.6 (CH), 128.4 (2 × CH), 126.9 (CH), 126.8 (CH), 126.7 (CH), 125.9 (CH), 122.7 (q, $J_{C\text{-}F}$ = 277.8 Hz, C), 61.6 (q, $J_{C\text{-}F}$ = 36.9 Hz, CH_2), 59.8 (C), 44.3 (CH_2), 21.5 (CH_3); ^{19}F NMR (376 MHz, $CDCl_3$) δ –73.6 (td, *J* = 8.2, 2.6 Hz, 3 × F); HRMS (ESI) Exact mass calculated for $[C_{25}H_{19}F_3NaO_3S]^+$ $[M+Na]^+$: 479.0899, found: 479.0902.

Enantiomeric excess was determined by HPLC using a Chiralpak AD-H column (95:5 *iso*-hexane:*i*-PrOH, 1.0 mL/min, 230 nm, 25 °C); t_r (minor) = 12.1 min, t_r (major) = 15.7 min, 91% ee.

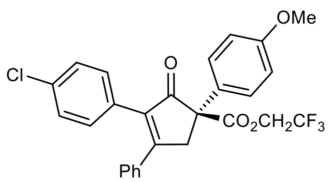

2,2,2-Trifluoroethyl (*R*)-3-(4-chlorophenyl)-1-(4-methoxyphenyl)-2-oxo-4-phenylcyclopent-3-ene-1-carboxylate (3ka). The title compound was prepared according to General Procedure F, using malonate ester **1k** (156.8 mg, 0.30 mmol) and phenylboronic acid (73.2 mg, 0.60 mmol) at 100 °C, and purified by column chromatography (10% EtOAc/*n*-pentane) to give a yellow oil (130.7 mg, 87%). R_f = 0.41 (20% EtOAc/petroleum ether); IR 2934, 1761 (C=O), 1702 (C=O), 1512, 1282, 1253, 1155, 1089, 1032, 730 cm^{-1}; $[\alpha]_D^{21}$ −66.7 (*c* 0.48, $CHCl_3$); ^{1}H NMR (400 MHz, $CDCl_3$) δ 7.44-7.41 (2H, m, Ar**H**), 7.40-7.31 (7H, m, Ar**H**), 7.23-7.19 (2H, m, Ar**H**), 6.94-6.90 (2H, m, Ar**H**), 4.56 (2H, qd, *J* = 8.3, 1.5 Hz, C$\mathbf{H}_2$$CF_3$), 4.08 (1H, d, *J* = 18.2 Hz, =CC$\mathbf{H}_a$$H_b$), 3.81 (3H, s, OC$\mathbf{H}_3$), 3.53 (1H, d, *J* = 18.2 Hz, =CC$H_a$$\mathbf{H}_b$); ^{13}C NMR (101 MHz, $CDCl_3$) δ 199.9 (C), 169.5 (C), 166.5 (C), 159.4 (C), 136.2 (C), 134.46 (C), 134.42 (C), 131.1 (2 × CH), 130.9 (CH), 130.1 (C), 129.5 (C), 129.0 (2 × CH), 128.9 (2 × CH), 128.7 (2 × CH), 128.3 (2 × CH), 122.8 (q, $J_{C\text{-}F}$ = 277.8 Hz, C), 114.3 (2 × CH), 62.4 (C), 61.3 (q, $J_{C\text{-}F}$ = 36.7 Hz, CH_2), 55.4 (CH_3), 43.8 (CH_2); ^{19}F NMR (376 MHz, $CDCl_3$) δ −73.6 (t, *J* = 8.3 Hz, 3 × F); HRMS (ESI) exact mass calculated for $[C_{27}H_{20}ClF_3NaO_4]^+$ $[M+Na]^+$: 523.0894, found: 523.0898. Enantiomeric excess was determined by HPLC with Chiralpak AD-H column (90:10 *iso*-hexane:*i*-PrOH, 1.0 mL/min, 254 nm, 25 °C); t_r (minor) = 34.1 min, t_r (major) = 54.4 min, 87% ee.

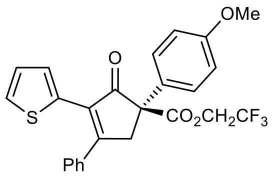

2,2,2-Trifluoroethyl (*R*)-1-(4-methoxyphenyl)-2-oxo-4-phenyl-3-(2-thienyl)cyclopent-3-ene-1-carboxylate (3la). The title compound was prepared according to General Procedure F, using malonate ester **1l** (148.3 mg, 0.30 mmol) and phenylboronic acid (73.2 mg, 0.60 mmol) at 100 °C, and purified by column chromatography (10% EtOAc/*n*-pentane) to give a yellow oil (118.5 mg, 84%). R_f = 0.42 (20% EtOAc/petroleum ether); IR 2934, 1762 (C=O), 1706 (C=O), 1610, 1512, 1282, 1252, 1153, 1032, 755 cm^{-1}; $[\alpha]_D^{20}$ −78.0 (*c* 0.82, $CHCl_3$); ^{1}H NMR (400 MHz, $CDCl_3$) δ 7.48-7.40 (7H, m, Ar**H**), 7.33-7.29 (2H, m, Ar**H**), 6.99 (1H, dd, *J* = 5.1, 3.7 Hz, Ar**H**), 6.94-6.90 (2H, m, Ar**H**), 4.57 (2H, qd, *J* = 8.3, 4.8 Hz, C$\mathbf{H}_2$$CF_3$), 4.01 (1H, d, *J* = 18.7 Hz, =CC$\mathbf{H}_a$$H_b$), 3.81 (3H, s, OC$\mathbf{H}_3$), 3.47 (1H, d, *J* = 18.7 Hz, =CC$H_a$$\mathbf{H}_b$); ^{13}C NMR (101 MHz, $CDCl_3$) δ 199.2 (C), 169.4 (C), 165.8 (C), 159.3 (C), 135.5 (C), 131.7 (C), 130.7 (C), 130.5 (CH), 129.5 (C), 129.0 (2 × CH), 128.7 (2 × CH), 128.4 (CH), 127.7 (2 × CH), 127.2 (CH), 126.9 (CH), 122.8 (q, $J_{C\text{-}F}$ = 277.6 Hz, C), 114.3 (2 × CH), 62.3 (C), 61.3 (q, $J_{C\text{-}F}$ = 36.7 Hz, CH_2), 55.4 (CH_3), 45.0 (CH_2); ^{19}F NMR

(376 MHz, $CDCl_3$) δ −73.6 (t, *J* = 8.3 Hz, 3 × F); HRMS (ESI) exact mass calculated for $[C_{25}H_{19}F_3NaO_4S]^+$ $[M+Na]^+$: 495.0848, found: 495.0849. Enantiomeric excess was determined by HPLC with Chiralpak AD-H column (90:10 *iso*-hexane:*i*-PrOH, 1.0 mL/min, 254 nm, 25 °C); t_r (minor) = 24.4 min, t_r (major) = 29.1 min, 86% ee.

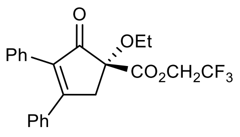

2,2,2-Trifluoroethyl (*R*)-1-ethoxy-2-oxo-3,4-diphenylcyclopent-3-ene-1-carboxylate (3ma). The title compound was prepared according to a slight modification of General Procedure F (in that a 20 mol% catalyst loading was used), using malonate ester **1m** (127.9 mg, 0.30 mmol), phenylboronic acid (73.1 mg, 0.60 mmol), $Ni(OAc)_2{\cdot}4H_2O$ (15.0 mg, 0.06 mmol) and (*R*)-PhPhox (**L1**, 24.4 mg, 0.06 mmol) at 80 °C, and purified by column chromatography (5% to 10% EtOAc/*n*-pentane) to give a pale yellow oil (56.1 mg, 46%). R_f = 0.15 (10% EtOAc/petroleum ether); $[\alpha]_D^{22}$ +72.0 (*c* 1.00, $CHCl_3$); IR 2854, 1747 (C=O), 1718 (C=O), 1437, 1358, 1281, 1232, 1171, 970, 669 cm^{-1}; m.p. 128-130 °C ($CHCl_3$); ^1H NMR (500 MHz, $CDCl_3$) δ 7.40-7.23 (10H, m, Ar**H**), 4.65 (1H, dq, *J* = 12.7, 8.3 Hz, C**H**$_a$H$_b$CF$_3$), 4.55 (1H, dq, *J* = 12.6, 8.3 Hz, CH$_a$**H**$_b$CF$_3$), 3.91-3.82 (2H, m, OC**H**$_2$CH$_3$), 3.51 (1H, d, *J* = 17.8 Hz, =CC**H**$_a$CH$_b$), 3.29 (1H, d, *J* = 17.8 Hz, =CCH$_a$C**H**$_b$), 1.30 (3H, t, *J* = 7.0 Hz, C**H**$_3$); ^{13}C NMR (126 MHz, $CDCl_3$) δ 198.6 (C), 168.9 (C), 165.9 (C), 137.1 (C), 134.5 (C), 131.3 (C), 130.9 (CH), 129.5 (2 × CH), 128.7 (4 × CH), 128.5 (CH), 128.4 (2 × CH), 122.8 (q, $J_{C\text{-}F}$ = 277.5 Hz, C), 83.5 (C), 63.0 (CH_2), 61.0 (q, $J_{C\text{-}F}$ = 37.1 Hz, CH_2), 42.0 (CH_2), 15.8 (CH_3); ^{19}F NMR (376 MHz, $CDCl_3$) δ −73.7 (t, *J* = 8.3 Hz, 3 × F); HRMS (ESI) Exact mass calculated for $[C_{22}H_{19}F_3NaO_4]^+$ $[M+H]^+$: 405.1308, found: 405.1308. Enantiomeric excess was determined by HPLC with Chiralpak OD-H column (95:5 *iso*-hexane: *i*-PrOH, 1.0 mL/min, 210 nm, 25 °C); t_r (major) = 10.6 min, t_r (minor) = 12.3 min, 92% ee.

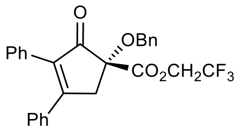

2,2,2-Trifluoroethyl (*R*)-1-(benzyloxy)-2-oxo-3,4-diphenylcyclopent-3-ene-1-carboxylate (3na). The title compound was prepared according to General Procedure F, using malonate ester **1n** (146.5 mg, 0.30 mmol) and phenylboronic acid (73.1 mg, 0.60 mmol) at 80 °C, and purified by column chromatography (10% EtOAc/*n*-pentane) to give a pale yellow oil (110.0 mg, 78%). R_f = 0.18 (7% EtOAc/petroleum ether); IR 3061, 1771 (C=O), 1703 (C=O), 1619, 1354, 1283, 1157, 1092, 911, 693 cm^{-1}; $[\alpha]_D^{20}$ +24.0 (*c* 1.00, $CHCl_3$); ^1H NMR (400 MHz, $CDCl_3$) δ 7.45-7.43 (2H, m, Ar**H**), 7.40-7.28 (11H, m, Ar**H**), 7.24-7.19 (2H, m, Ar**H**), 4.98-4.92 (2H, m, OC**H**$_2$Ar), 4.73 (1H, dq, *J* = 12.6, 8.3 Hz, C**H**$_a$H$_b$CF$_3$), 4.56 (1H, dq, *J* = 12.6, 8.2 Hz, CH$_a$**H**$_b$CF$_3$), 3.54 (1H, d, *J* = 18.0 Hz, =CC**H**$_a$H$_b$), 3.36 (1H, d, *J* = 17.9 Hz, =CCH$_a$**H**$_b$); ^{13}C NMR (101 MHz, $CDCl_3$) δ 198.7 (C), 168.9 (C), 165.9 (C), 137.7 (C), 137.1 (C), 134.4 (C), 131.3 (C), 130.9 (CH), 129.6 (2 × CH), 128.8 (4 × CH), 128.59

(CH), 128.57 (2 × CH), 128.5 (2 × CH), 128.3 (2 × CH), 128.1 (CH), 122.8 (q, $J_{C\text{-}F}$ = 277.7 Hz, C), 83.0 (C), 69.4 (CH_2), 61.1 (q, $J_{C\text{-}F}$ = 37.2 Hz, CH_2), 42.9 (CH_2); ^{19}F NMR (376 MHz, $CDCl_3$) δ –73.6 (t, *J* = 8.3 Hz, 3 × F); HRMS (ESI) Exact mass calculated for $[C_{27}H_{21}F_3NaO_4]^+$ $[M+Na]^+$: 489.1284, found 489.1301. Enantiomeric excess was determined by HPLC using a Chiralpak AD-H column (95:5 *iso*-hexane:*i*-PrOH, 1.0 mL/min, 254 nm, 25 °C); t_r (major) = 19.0 min, t_r (minor) = 20.7 min, 93% ee.

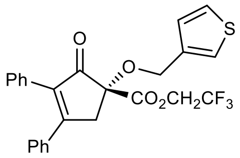

2,2,2-Trifluoroethyl (*R*)-2-oxo-3,4-diphenyl-1-(thiophen-3-ylmethoxy)cyclopent-3-ene-1-carboxylate (3oa). The title compound was prepared according to slightly modified General Procedure F, (20 mol% catalyst loading was used), using malonate ester **1o** (148.3 mg, 0.30 mmol), phenylboronic acid (73.1 mg, 0.60 mmol), $Ni(OAc)_2{\cdot}4H_2O$ (15.0 mg, 0.06 mmol) and (*R*)-PhPhox (**L1**, 24.4 mg, 0.06 mmol) at 80 °C, and purified by column chromatography (5%-10% EtOAc/*n*-pentane) to give an orange oil (111.0 mg, 78%). R_f = 0.16 (10% EtOAc/petroleum ether); $[\alpha]_D^{23}$ +20.0 (*c* 1.00, $CHCl_3$); IR 1770 (C=O), 1702 (C=O), 1620, 1412, 1354, 1282, 1156, 1077, 766, 515 cm^{-1}; ^{1}H NMR (400 MHz, $CDCl_3$) δ 7.41-7.18 (13H, m, Ar**H**), 5.00-4.95 (2H, m, ArC**H**$_2$O), 4.72 (1H, dq, *J* = 12.6, 8.3 Hz, C**H**$_a$H$_b$CF$_3$), 4.56 (1H, dq, *J* = 12.6, 8.3 Hz, CH$_a$**H**$_b$CF$_3$), 3.53 (1H, d, *J* = 18.0 Hz, =CC**H**$_a$CH$_b$), 3.35 (1H, d, *J* = 18.0 Hz, =CCH$_a$C**H**$_b$); ^{13}C NMR (126 MHz, $CDCl_3$) δ 198.7 (C), 168.9 (C), 165.9 (C), 138.7 (C), 137.1 (C), 134.4 (C), 131.2 (C), 131.0 (CH), 129.6 (2 × CH), 128.8 (4 × CH), 128.6 (CH), 128.4 (2 × CH), 127.8 (CH), 126.1 (CH), 123.8 (CH), 122.8 (q, $J_{C\text{-}F}$ = 277.5 Hz, C), 82.9 (C), 64.7 (CH_2), 61.1 (q, $J_{C\text{-}F}$ = 37.1 Hz, CH_2), 42.9 (CH_2); ^{19}F NMR (376 MHz, $CDCl_3$) δ −73.6 (t, *J* = 8.3 Hz, 3 × F); HRMS (ESI) Exact mass calculated for $[C_{25}H_{19}F_3NaO_4S]^+$ $[M+Na]^+$: 495.0848, found: 495.0845. Enantiomeric excess was determined by HPLC with Chiralpak OD-H column (95:5 *iso*-hexane: *i*-PrOH, 1.0 mL/min, 210 nm, 25 °C); t_r (minor) = 19.7 min, t_r (major) = 29.5 min, 93% ee.

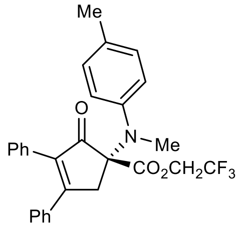

2,2,2-Trifluoroethyl (*R*)-1-[methyl(4-methylphenyl)amino]-2-oxo-3,4-diphenylcyclopent-3-ene-1-carboxylate (3pa). The title compound was prepared according to General Procedure F, using malonate ester **1p** (150.4 mg, 0.30 mmol) and phenylboronic acid (73.1 mg, 0.60 mmol) at 80 °C, and purified by column chromatography (0% to 10% EtOAc/*n*-pentane) to give an orange oil (141.0 mg, 98%). R_f = 0.30 (10% EtOAc/petroleum ether); $[\alpha]_D^{21}$ −156.0 (*c* 1.00, $CHCl_3$); IR 1769 (C=O), 1703 (C=O), 1514, 1445, 1353, 1290, 1156, 1072, 866, 665 cm^{-1}; ^{1}H NMR (400 MHz, $CDCl_3$) δ 7.38-7.21 (10H, m, Ar**H**), 7.06 (2H, d, *J* = 8.4 Hz, Ar**H**), 6.87-6.83 (2H, m,

ArH), 4.63-4.45 (2H, m, C**H**$_2$CF$_3$), 3.89 (1H, d, *J* = 18.0 Hz, =CC**H**$_a$CH$_b$), 3.33 (1H, d, *J* = 18.0 Hz, =CCH$_a$C**H**$_b$), 3.10 (3H, s, NC**H**$_3$), 2.28 (3H, s, ArC**H**$_3$); ^{13}C NMR (126 MHz, CDCl$_3$) δ 198.7 (C), 169.5 (C), 167.4 (C), 147.2 (C), 136.8 (C), 134.6 (C), 131.5 (C), 131.1 (C), 130.8 (CH), 129.65 (2 × CH), 129.63 (2 × CH), 128.73 (2 × CH), 128.67 (2 × CH), 128.51 (CH), 128.45 (2 × CH), 122.8 (q, $J_{C\text{-}F}$ = 277.9 Hz, C), 120.3 (2 × CH), 74.1 (C), 61.3 (q, $J_{C\text{-}F}$ = 36.8 Hz, CH$_2$), 41.0 (CH$_3$), 39.2 (CH$_2$), 20.7 (CH$_3$); ^{19}F NMR (376 MHz, CDCl$_3$) δ −73.5 (t, *J* = 8.3 Hz, 3 × F); HRMS (ESI) Exact mass calculated for $[C_{28}H_{24}F_3NNaO_3]^+$ $[M+Na]^+$: 502.1600, found: 502.1604. Enantiomeric excess was determined by HPLC with Chiralpak AD-H column (95:5 *iso*-hexane:*i*-PrOH, 1.0 mL/min, 254 nm, 25 °C); t$_r$ (major) = 9.4 min, t$_r$ (minor) = 15.7 min, 90% ee.

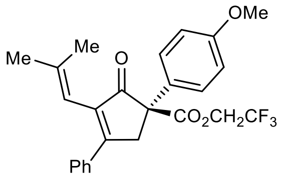

2,2,2-Trifluoroethyl (*R*)-1-(4-methoxyphenyl)-3-(2-methylprop-1-en-1-yl)-2-oxo-4-phenylcyclopent-3-ene-1-carboxylate (7). Following General Procedure F, using malonate ester **6** (139.9 mg, 0.30 mmol) and phenylboronic acid (73.2 mg, 0.60 mmol) at 100 °C, and purified by column chromatography (0% to 10% EtOAc/*n*-pentane) to give a yellow oil (101.5 mg, 76%). R$_f$ = 0.20 (10% EtOAc/petroleum ether); IR 2970, 1760 (C=O), 1698 (C=O), 1609, 1513, 1252, 1153, 972, 731, 648 cm^{-1}; $[\alpha]_D^{21}$ −190.0 (*c* 0.40, CHCl$_3$); ^{1}H NMR (500 MHz, CDCl$_3$) δ 7.72-7.69 (2H, m, Ar**H**), 7.46-7.42 (3H, m, Ar**H**), 7.39-7.36 (2H, m, Ar**H**), 6.91-6.88 (2H, m, Ar**H**), 5.82 (1H, dt, *J* = 3.2, 1.8 Hz, **H**C=C(CH$_3$)$_2$), 4.61-4.47 (2H, m, C**H**$_2$CF$_3$), 4.01 (1H, dd, *J* = 17.9, 2.1 Hz, =CC**H**$_a$H$_b$), 3.80 (3H, s, OC**H**$_3$), 3.45 (1H, dd, *J* = 17.9, 2.1 Hz, =CCH$_a$**H**$_b$), 1.90 (3H, d, *J* = 1.6 Hz, HC=C(C**H**$_3$)$_2$), 1.46 (3H, d, *J* = 1.2 Hz, HC=C(C**H**$_3$)$_2$); ^{13}C NMR (126 MHz, CDCl$_3$) δ 200.6 (C), 169.9 (C), 164.0 (C), 159.2 (C), 141.8 (C), 135.4 (C), 130.6 (CH), 129.8 (C), 128.78 (2 × CH), 128.77 (2 × CH), 128.2 (2 × CH), 122.8 (q, $J_{C\text{-}F}$ = 277.3 Hz, C), 115.2 (CH), 114.2 (2 × CH), 62.0 (C), 61.2 (q, $J_{C\text{-}F}$ = 36.5 Hz, CH$_2$) 55.4 (CH$_3$), 43.0 (CH$_2$), 26.1 (CH$_3$), 20.7 (CH$_3$); ^{19}F NMR (376 MHz, CDCl$_3$) δ −73.6 (td, *J* = 8.3, 2.2 Hz, 3 × F); HRMS (ESI) Exact mass calculated for $[C_{25}H_{23}F_3NaO_4]^+$ $[M+Na]^+$: 467.1441, found: 467.1437. Enantiomeric excess was determined by HPLC using a Chiralpak AD-H column (90:10 *iso*-hexane:*i*-PrOH, 1.0 mL/min, 230 nm, 25 °C); t$_r$ (major) = 16.0 min, t$_r$ (minor) = 25.6 min, 80% ee.

2,2,2-Trifluoroethyl (*R*)-1-methyl-2-oxo-3,4-diphenylcyclopent-3-ene-1-carboxylate (*ent*-3qa)

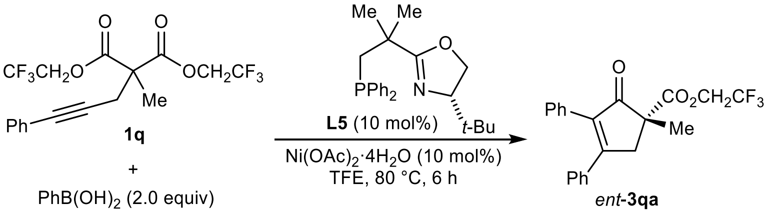

An oven-dried microwave vial fitted with a stirrer bar was charged with malonate ester **1q** (110.8 mg, 0.30 mmol), phenylboronic acid (73.1 mg, 0.60 mmol), $Ni(OAc)_2{\cdot}4H_2O$ (7.5 mg, 0.03 mmol) and (*S*)-*t*-Bu-NeoPHOX (**L5**, 11.0 mg, 0.03 mmol). The vial was capped with a crimp cap PTFE seal and evacuated and back filled with argon (5 cycles). TFE (3 mL) which had been freshly degassed (using 5 freeze-pump-thaw cycles) was added under argon flow, the septum was resealed with a layer of vacuum grease, and the contents were stirred at room temperature for 10 min followed by stirring at 80 °C for 6 h. The reaction was cooled to room temperature, diluted with EtOAc (5 mL) and washed with brine (10 mL). The aqueous layer was extracted with EtOAc (5 mL). The combined organic layers were dried (Na_2SO_4), filtered and concentrated under reduced pressure. The residue was purified by column chromatography (0% to 5% EtOAc/petroleum ether) to give an off-white oil (52.0 mg, 46%). R_f = 0.17 (10% EtOAc/petroleum ether); IR 2934, 1760, 1699, 1623, 1352, 1154, 1097, 1073, 974, 694 cm^{-1}; $[\alpha]_D^{25}$–24.3 (*c* 1.00, $CHCl_3$); ^{1}H NMR (400 MHz, $CDCl_3$) δ 7.40-7.28 (8H, m, Ar**H**), 7.25-7.21 (2H, m, Ar**H**), 4.60 (1H, dq, *J* = 12.7, 8.3 Hz, C$\mathbf{H}_a$H$_b$CF$_3$), 4.47 (1H, dq, *J* = 12.7, 8.3 Hz, CH$_a\mathbf{H}_b$CF$_3$), 3.62 (1H, d, *J* = 18.1 Hz, =C$\mathbf{H}_a$H$_b$), 2.96 (1H, d, *J* = 18.1 Hz, =CH$_a\mathbf{H}_b$), 1.63 (3H, s, C**H**$_3$); ^{13}C NMR (101 MHz, $CDCl_3$) δ 202.9 (C), 170.5 (C), 166.3 (C), 137.1 (C), 134.9 (C), 131.7 (C), 130.6 (CH), 129.6 (CH), 128.73 (CH), 128.68 (CH), 128.4 (CH), 128.3 (CH), 122.9 (q, *J* = 277.5 Hz, C), 61.1 (q, *J* = 36.7 Hz, CH_2), 54.3 (C), 43.2 (CH_2), 21.1 (CH_3); ^{19}F NMR (376 MHz, $CDCl_3$) δ –73.8 (t, *J* = 8.4 Hz, 3 × F); HRMS (ESI) Exact mass calculated for $[C_{21}H_{17}F_3NaO_3]^+$ $[M+Na]^+$: 397.1022, found: 397.1028. Enantiomeric excess was determined by HPLC using a Chiralpak AD-H column (97:3 *iso*-hexane:*i*-PrOH, 0.8 mL/min, 254 nm, 25 °C); t_r (major) = 12.5 min, t_r (minor) = 13.5 min, 59% ee.

2,2,2-Trifluoroethyl (*R*)-1-benzyl-2-oxo-3,4-diphenylcyclopent-3-ene-1-carboxylate (*ent*-3ra)

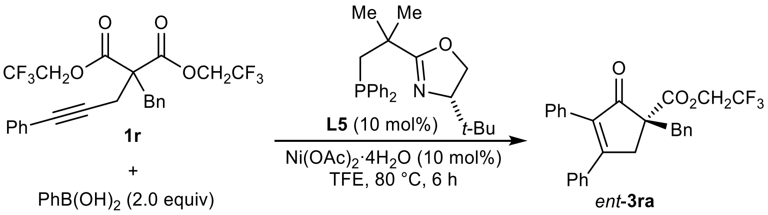

An oven-dried microwave vial fitted with a stirrer bar was charged with malonate ester **1r** (141.6 mg, 0.30 mmol), phenylboronic acid (73.1 mg, 0.60 mmol), $Ni(OAc)_2{\cdot}4H_2O$ (7.5 mg,

0.03 mmol) and (*S*)-*t*-Bu-NeoPHOX (**L5**, 11.0 mg, 0.03 mmol). The vial was capped with a crimp cap PTFE seal and evacuated and back filled with argon (5 cycles). TFE (3 mL) which had been freshly degassed (using 5 freeze-pump-thaw cycles) was added under argon flow, the septum was resealed with a layer of vacuum grease, and the contents were stirred at room temperature for 10 min followed by stirring at 80 °C for 6 h. The reaction was cooled to room temperature, diluted with EtOAc (5 mL) and washed with brine (10 mL). The aqueous layer was extracted with EtOAc (5 mL). The combined organic layers were dried (Na_2SO_4), filtered and concentrated under reduced pressure. The residue was purified by column chromatography (0% to 10% EtOAc/petroleum ether) to give a colorless oil (60.3 mg, 45%). R_f = 0.22 (7% EtOAc/petroleum ether); IR 3084, 1759 (C=O), 1699 (C=O), 1624, 1596, 1283, 1246, 1162, 1074, 642 cm^{-1}; $[\alpha]_D^{20}$ –72.0 (*c* 1.00, $CHCl_3$); ^{1}H NMR (400 MHz, $CDCl_3$) δ 7.33-7.17 (11H, m, Ar**H**), 7.10-7.07 (2H, m, Ar**H**), 7.06-7.00 (2H, m, Ar**H**), 4.65 (1H, dq, *J* = 12.6, 8.3 Hz, C$\mathbf{H}_a$$H_b$$CF_3$), 4.52 (1H, dq, *J* = 12.7, 8.3 Hz, $CH_a$$\mathbf{H}_b$$CF_3$), 3.56-3.48 (2H, m, ArC$\mathbf{H}_a$$H_b$ and =CC$\mathbf{H}_a$$H_b$), 3.38 (1H, d, *J* = 13.7 Hz, Ar$CH_a$$\mathbf{H}_b$), 3.13 (1H, d, *J* = 18.4 Hz, =C$CH_a$$\mathbf{H}_b$); ^{13}C NMR (101 MHz, $CDCl_3$) δ 202.2 (C), 169.8 (C), 167.9 (C), 138.3 (C), 135.4 (C), 134.8 (C), 131.6 (C), 130.3 (CH), 130.2 (2 × CH), 129.5 (2 × CH), 128.55 (4 × CH), 128.51 (2 × CH), 128.2 (CH), 128.0 (2 × CH), 127.3 (2 × CH), 122.9 (q, J_{C-F} = 277.8 Hz, C), 61.2 (q, J_{C-F} = 36.7 Hz, CH_2), 59.2 (C), 39.8 (CH_2), 39.0 (CH_2); ^{19}F NMR (376 MHz, $CDCl_3$) δ –73.6 (t, *J* = 8.3 Hz, 3 × F); HRMS (ESI) Exact mass calculated for $[C_{27}H_{21}F_3NaO_3]^+$ $[M+Na]^+$: 473.1335, found 473.1344. Enantiomeric excess was determined by HPLC using a Chiralpak AD-H column (95:5 *iso*-hexane:*i*-PrOH, 1.0 mL/min, 230 nm, 25 °C); t_r (major) = 11.2 min, t_r (minor) = 12.5 min, 54% ee.

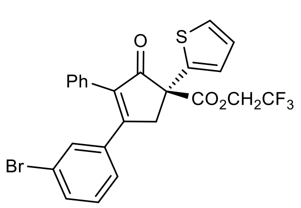

2,2,2-Trifluoroethyl (*R*)-4-(3-bromophenyl)-2-oxo-3-phenyl-1-(2-thienyl)cyclopent-3-ene-1-carboxylate (3ab). The title compound was prepared according to General Procedure F, using malonate ester **1a** (139.3 mg, 0.30 mmol) and 3-bromophenylboronic acid (120.5 mg, 0.60 mmol) at 80 °C, and purified by column chromatography (10% EtOAc/*n*-pentane) to give a pale yellow oil (133.0 mg, 85%). R_f = 0.19 (0% to 10% EtOAc/petroleum ether); IR 3060, 1765 (C=O), 1705 (C=O), 1406, 1347, 1281, 1234, 1158, 788, 698 cm^{-1}; $[\alpha]_D^{23}$ –16.0 (*c* 1.00, $CHCl_3$); ^{1}H NMR (400 MHz, $CDCl_3$) δ 7.55-7.52 (2H, m, Ar**H**), 7.39-7.17 (9H, m, Ar**H**), 7.06 (1H, dd, *J* = 5.2, 3.7 Hz, Ar**H**), 4.67-4.49 (2H, m, C$\mathbf{H}_2$$CF_3$), 4.07 (1H, d, *J* = 18.0 Hz, =CC$\mathbf{H}_a$$H_b$), 3.65 (1H, d, *J* = 18.0 Hz, =C$CH_a$$\mathbf{H}_b$); ^{13}C NMR (101 MHz, $CDCl_3$) δ 198.5 (C), 168.3 (C), 164.1 (C), 138.7 (C), 137.6 (C), 136.6 (C), 133.6 (CH), 131.0 (CH), 130.8 (C), 130.3 (CH), 129.5 (2 × CH), 128.88 (CH), 128.83 (2 × CH), 127.1 (CH), 126.99 (CH), 126.93 (CH), 126.0 (CH), 122.9 (C), 122.7 (q, J_{C-F} = 277.4 Hz,

C), 61.7 (q, J_{C-F} = 37.0 Hz, CH_2), 59.9 (C), 44.3 (CH_2); ^{19}F NMR (376 MHz, $CDCl_3$) δ –73.6 (t, *J* = 8.1 Hz, 3 × F); HRMS (ESI) Exact mass calculated for $[C_{24}H_{16}BrF_3NaO_3S]^+$ $[M+Na]^+$: 542.9848, found 542.9854; Enantiomeric excess was determined by HPLC using a Chiralpak AD-H column (97:3 *iso*-hexane:*i*-PrOH, 1.0 mL/min, 230 nm, 25 °C); t_r (minor) = 25.3 min, t_r (major) = 31.0 min, 93% ee.

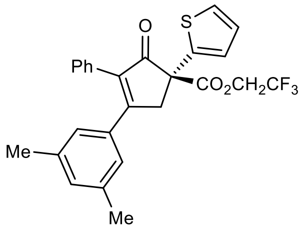

2,2,2-Trifluoroethyl (*R*)-4-(3,5-dimethylphenyl)-2-oxo-3-phenyl-1-(2-thienyl)cyclopent-3-ene-1-carboxylate (3ac). The title compound was prepared according to General Procedure F, using malonate ester **1a** (139.3 mg, 0.30 mmol) and 3,5-dimethylphenylboronic acid (89.8 mg, 0.60 mmol) at 80 °C, and purified by column chromatography (0% to 10% EtOAc/*n*-pentane) to give a pale yellow oil (106.0 mg, 75%). R_f = 0.30 (10% EtOAc/petroleum ether); IR 2921, 1765 (C=O), 1704 (C=O), 1623, 1280, 1156, 1069, 850, 788, 694 cm^{-1}; $[\alpha]_D^{23}$ –44.0 (*c* 1.00, $CHCl_3$); ^{1}H NMR (400 MHz, $CDCl_3$) δ 7.36-7.29 (5H, m, Ar**H**), 7.26-7.23 (2H, m, Ar**H**), 7.05-7.02 (2H, m, Ar**H**), 6.97-6.96 (2H, m, Ar**H**), 4.65-4.47 (2H, m, C**H**$_2$CF$_3$), 4.06 (1H, d, *J* = 18.0 Hz, =CC**H**$_a$H$_b$), 3.65 (1H, d, *J* = 18.0 Hz, =CCH$_a$**H**$_b$), 2.23 (6H, s, 2 × C**H**$_3$); ^{13}C NMR (101 MHz, $CDCl_3$) δ 198.9 (C), 168.7 (C), 166.8 (C), 139.2 (C), 138.3 (CH), 136.3 (C), 134.5 (C), 132.5 (CH), 131.6 (C), 129.6 (2 × CH), 128.6 (2 × CH), 128.4 (CH), 126.9 (CH), 126.8 (CH), 126.1 (2 × CH), 125.8 (CH), 122.8 (q, J_{C-F} = 277.9 Hz, C), 61.6 (q, J_{C-F} = 36.8 Hz, CH_2), 59.8 (C), 44.5 (CH_2), 21.4 (2 × CH_3); ^{19}F NMR (376 MHz, $CDCl_3$) δ –73.6 (t, *J* = 8.3 Hz, 3 × F); HRMS (ESI) Exact mass calculated for $[C_{26}H_{21}F_3NaO_3S]^+$ $[M+Na]^+$: 493.1056, found 493.1071. Enantiomeric excess was determined by HPLC using a Chiralpak AD-H column (93:7 *iso*-hexane:*i*-PrOH, 1.0 mL/min, 230 nm, 25 °C); t_r (minor) = 9.1 min, t_r (major) = 10.3 min, 92% ee.

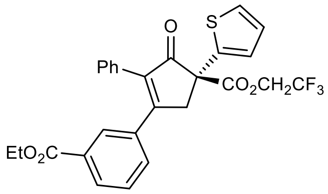

Ethyl (*R*)-3-{3-oxo-2-phenyl-4-(2-thienyl)-4-[(2,2,2-trifluoroethoxy)carbonyl]cyclopent-1-en-1-yl}benzoate (3ad). The title compound was prepared according to General Procedure F, using malonate ester **1a** (139.3 mg, 0.30 mmol) and 3-(ethoxycarbonyl)phenylboronic acid (116.4 mg, 0.60 mmol) at 80 °C, and purified by column chromatography (0% to 10% EtOAc/*n*-pentane) to give a yellow oil (142.4 mg, 92%). R_f = 0.05 (10% EtOAc/petroleum ether); IR 2980, 1766 (C=O), 1709 (C=O), 1431, 1271, 1160, 909, 753, 733, 699 cm^{-1}; $[\alpha]_D^{23}$ –20.0 (*c* 1.00, $CHCl_3$); ^{1}H NMR (500 MHz, $CDCl_3$) δ 8.10 (1H, td, *J* = 1.8, 0.6 Hz, Ar**H**), 8.06 (1H, dt, *J* = 7.7, 1.4 Hz, Ar**H**), 7.49 (1H, ddd, *J* = 7.9, 1.9, 1.3 Hz, Ar**H**), 7.38-7.30

(6H, m, Ar**H**), 7.24-7.20 (2H, m, Ar**H**), 7.05 (1H, dd, *J* = 5.2, 3.7 Hz, Ar**H**), 4.65-4.48 (2H, m, C**H**$_2$CF$_3$), 4.35 (2H, q, *J* = 7.1 Hz, OC**H**$_2$CH$_3$), 4.13 (1H, d, *J* = 18.1 Hz, =CC**H**$_a$H$_b$), 3.71 (1H, d, *J* = 18.1 Hz, =CCH$_a$**H**$_b$), 1.35 (3H, t, *J* = 7.1 Hz, OCH$_2$C**H**$_3$); ^{13}C NMR (126 MHz, CDCl$_3$) δ 198.7 (C), 168.4 (C), 165.9 (C), 164.8 (C), 138.9 (C), 137.5 (C), 134.8 (C), 132.7 (CH), 131.6 (CH), 131.3 (C), 131.0 (C), 129.6 (2 × CH), 129.2 (CH), 128.85 (3 × CH), 128.77 (CH), 126.97 (CH), 126.93 (CH), 126.0 (CH), 122.7 (q, J_{C-F} = 277.6 Hz, C), 61.6 (q, J_{C-F} = 37.7 Hz, CH$_2$), 61.5 (CH$_2$) 59.9 (C), 44.3 (CH$_2$), 14.4 (CH$_3$); ^{19}F NMR (376 MHz, CDCl$_3$) δ –73.6 (t, *J* = 8.2 Hz, 3 × F); HRMS (ESI) Exact mass calculated for $[C_{27}H_{21}F_3NaO_5S]^+$ $[M+Na]^+$: 537.0954, found: 537.0953. Enantiomeric excess was determined by HPLC using a Chiralpak ADH column (90:10 *iso*-hexane:*i*-PrOH, 1.0 mL/min, 230 nm, 25 °C); t$_r$ (minor) = 14.9 min, t$_r$ (major) = 17.6 min, 92% ee.

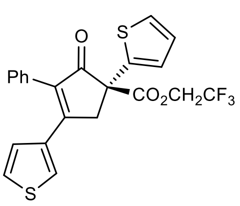

2,2,2-Trifluoroethyl (*R*)-2-oxo-3-phenyl-1-(2-thienyl)-4-(3-thienyl)cyclopent-3-ene-1-carboxylate (3ae). The title compound was prepared according to General Procedure F, using malonate ester **1a** (139.3 mg, 0.30 mmol) and 3-thienylboronic acid (76.8 mg, 0.60 mmol) at 80 °C, and purified by column chromatography (0% to 10% EtOAc/*n*-pentane) to give a pale yellow oil (99.1 mg, 73%). R$_f$ = 0.18 (7% EtOAc/petroleum ether); IR 3101, 1764 (C=O), 1700 (C=O), 1620, 1492, 1332, 1283, 1160, 792, 701 cm^{-1}; $[\alpha]_D^{20}$ –20.0 (*c* 1.00, CHCl$_3$); ^{1}H NMR (400 MHz, CDCl$_3$) δ 7.59 (1H, dd, *J* = 2.9, 1.3 Hz, Ar**H**), 7.43-7.35 (3H, m, Ar**H**), 7.31-7.21 (5H, m, Ar**H**), 7.01 (1H, dd, *J* = 5.2, 3.7 Hz, Ar**H**), 6.93 (1H, dd, *J* = 5.2, 1.3 Hz, Ar**H**), 4.65-4.47 (2H, m, C**H**$_2$CF$_3$), 4.09 (1H, d, *J* = 17.7 Hz, =CC**H**$_a$H$_b$), 3.69 (1H, d, *J* = 17.7 Hz, =CCH$_a$**H**$_b$); ^{13}C NMR (101 MHz, CDCl$_3$) δ 198.7 (C), 168.7 (C), 159.5 (C), 139.3 (C), 136.1 (C), 135.4 (C), 132.1 (C), 129.6 (2 × CH), 128.9 (3 × CH), 128.8 (CH), 127.1 (CH), 126.9 (CH), 126.8 (CH), 126.4 (CH), 125.8 (CH), 122.7 (q, J_{C-F} = 277.4 Hz, C), 61.6 (q, J_{C-F} = 36.8 Hz, CH$_2$), 59.5 (C), 43.9 (CH$_2$); ^{19}F NMR (376 MHz, CDCl$_3$) δ –73.6 (t, *J* = 8.3 Hz, 3 × F); HRMS (ESI) Exact mass calculated for $[C_{22}H_{15}F_3NaO_3S_2]^+$ $[M+Na]^+$: 471.0307, found 471.0320. Enantiomeric excess was determined by HPLC using a Chiralpak ASH column (98:2 *iso*-hexane:*i*-PrOH, 0.5 mL/min, 254 nm, 25 °C); t$_r$ (minor) = 53.8 min, t$_r$ (major) = 57.9 min, 80% ee.

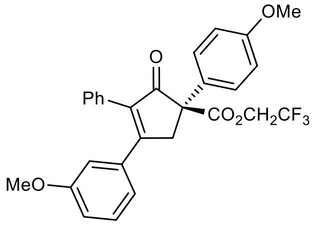

2,2,2-Trifluoroethyl (*R*)-4-(3-methoxyphenyl)-1-(4-methoxyphenyl)-2-oxo-3-phenylcyclopent-3-ene-1-carboxylate (3cf). The title compound was prepared according to General Procedure F, using malonate ester **1c** (146.5 mg, 0.30 mmol) and 3-methoxyphenylboronic acid (91.2 mg, 0.60 mmol) at 100 °C, and purified by column chromatography (0% to 10% EtOAc/*n*-pentane) to give a yellow oil (136.1 mg, 91%). R$_f$ = 0.12

(10% EtOAc/petroleum ether); IR 2936, 1762 (C=O), 1702 (C=O), 1597, 1513, 1284, 1253, 1153, 1033, 731 cm^{-1}; $[\alpha]_D^{20}$ −104.3 (*c* 0.46, $CHCl_3$); ^{1}H NMR (400 MHz, $CDCl_3$) δ 7.47-7.43 (2H, m, Ar**H**), 7.41-7.33 (3H, m, Ar**H**), 7.29-7.23 (3H, m, Ar**H**), 7.01 (1H, ddd, *J* = 7.7, 1.7, 1.0 Hz, Ar**H**), 6.96-6.91 (3H, m, Ar**H**), 6.86 (1H, dd, *J* = 2.6, 1.6 Hz, Ar**H**), 4.63-4.54 (2H, m, C**H**$_2$CF$_3$), 4.10 (1H, d, *J* = 18.1 Hz, =CC**H**$_a$H$_b$), 3.83 (3H, s, OC**H**$_3$), 3.59 (3H, s, OC**H**$_3$), 3.54 (d, *J* = 18.1 Hz, =CCH$_a$**H**$_b$); ^{13}C NMR (101 MHz, $CDCl_3$) δ 200.2 (C), 169.6 (C), 165.7 (C), 159.5 (C), 159.3 (C), 137.7 (C), 135.8 (C), 131.9 (C), 129.79 (C), 129.77 (CH), 129.7 (2 × CH), 128.8 (2 × CH), 128.7 (2 × CH), 128.4 (CH), 122.8 (q, J_{C-F} = 277.8 Hz, C), 120.7 (CH), 117.0 (CH), 114.3 (2 × CH), 113.5 (CH), 62.4 (C), 61.3 (q, J_{C-F} = 36.7 Hz, CH_2), 55.4 (CH_3), 55.1 (CH_3), 43.8 (CH_2); ^{19}F NMR (376 MHz, $CDCl_3$) δ −73.6 (t, *J* = 8.3 Hz, 3 × F); HRMS (ESI) exact mass calculated for $[C_{28}H_{23}F_3NaO_5]^+$ $[M+Na]^+$: 519.1390, found: 519.1399. Enantiomeric excess was determined by HPLC with Chiralpak AD-H column (90:10 *iso*-hexane:*i*-PrOH, 1.0 mL/min, 254 nm, 25 °C); t_r (minor) = 33.9 min, t_r (major) = 37.4 min, 86% ee.

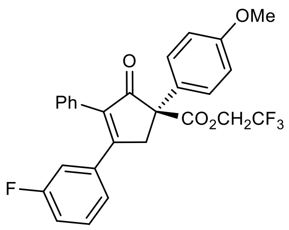

2,2,2-Trifluoroethyl (*R*)-4-(3-fluorophenyl)-1-(4-methoxyphenyl)-2-oxo-3-phenylcyclopent-3-ene-1-carboxylate (3cg). The title compound was prepared according to General Procedure F, using malonate ester **1c** (146.5 mg, 0.30 mmol) and 3-fluorophenylboronic acid (83.9 mg, 0.60 mmol) at 120 °C, and purified by column chromatography (0% to 10% EtOAc/*n*-pentane) to give a yellow oil (118.2 mg, 81%). R_f = 0.14 (10% EtOAc/petroleum ether); IR 2839, 1763 (C=O), 1705 (C=O), 1513, 1483, 1282, 1252, 1154, 1031, 732 cm^{-1}; $[\alpha]_D^{21}$ −80.0 (*c* 1.00, $CHCl_3$); ^{1}H NMR (500 MHz, $CDCl_3$) δ 7.45-7.43 (2H, m, Ar**H**), 7.41-7.34 (3H, m, Ar**H**), 7.30-7.24 (3H, m, Ar**H**), 7.16 (1H, dq, *J* = 8.0, 1.6 Hz, Ar**H**), 7.09-7.05 (2H, m, Ar**H**), 6.96-6.92 (2H, m, Ar**H**), 4.60-4.55 (2H, m, C**H**$_2$CF$_3$), 4.09 (1H, dd, *J* = 18.0, 2.2 Hz, =CC**H**$_a$H$_b$), 3.81 (3H, s, OC**H**$_3$), 3.51 (dd, *J* = 18.1, 2.2 Hz, =CCH$_a$**H**$_b$); ^{13}C NMR (126 MHz, $CDCl_3$) δ 200.0 (C), 169.4 (C), 164.0 (C), 162.6 (d, J_{C-F} = 247.7 Hz, C), 159.3 (C), 138.3 (C), 136.7 (d, J_{C-F} = 7.6 Hz, C), 131.2 (C), 130.3 (d, J_{C-F} = 8.3 Hz, C), 129.6 (2 × CH), 128.78 (2 × CH), 128.76 (2 × CH), 128.71 (CH), 128.66 (C), 124.1 (d, J_{C-F} = 8.3 Hz, C), 122.8 (q, J_{C-F} = 277.8 Hz, C), 117.5 (d, J_{C-F} = 21.3 Hz, C), 115.2 (d, J_{C-F} = 22.7 Hz, C), 114.3 (2 × CH), 62.4 (C), 61.3 (q, J_{C-F} = 37.1 Hz, CH_2), 55.4 (CH_3), 43.7 (CH_2); ^{19}F NMR (376 MHz, $CDCl_3$) δ −73.6 (m, 3 × F), –111.7 (q, *J* = 8.1 Hz, F); HRMS (ESI) exact mass calculated for $[C_{27}H_{20}F_4NaO_4]^+$ $[M+Na]^+$: 507.1190, found: 507.1187. Enantiomeric excess was determined by HPLC with Chiralpak AD-H column (90:10 *iso*-hexane:*i*-PrOH, 1.0 mL/min, 230 nm, 25 °C); t_r (minor) = 27.5 min, t_r (major) = 30.4 min, 81% ee.

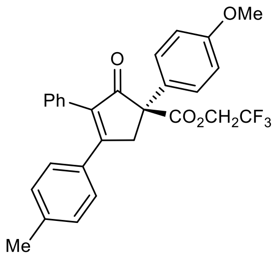

2,2,2-Trifluoroethyl (*R*)-1-(4-methoxyphenyl)-2-oxo-3-phenyl-4-(4-methylphenyl)cyclopent-3-ene-1-carboxylate (3ch). The title compound was prepared according to General Procedure F, using malonate ester **1c** (146.5 mg, 0.30 mmol) and 4-methylphenylboronic acid (81.6 mg, 0.60 mmol) at 100 °C, and purified by column chromatography (10% EtOAc/*n*-pentane) to give a yellow oil (110.4 mg, 76%). R_f = 0.11 (10% EtOAc/petroleum ether); IR 2931, 1763 (C=O), 1698 (C=O), 1512, 1350, 1252, 1153, 1033, 818, 732 cm^{-1}; $[\alpha]_D^{21}$ –80.0 (*c* 1.00, $CHCl_3$); ^1H NMR (400 MHz, $CDCl_3$) δ 7.44-7.41 (2H, m, Ar**H**), 7.38-7.32 (3H, m, Ar**H**), 7.28-7.25 (4H, m, Ar**H**), 7.10 (2H, d, *J* = 7.9 Hz, Ar**H**), 6.93-6.90 (2H, m, Ar**H**), 4.61-6.51 (2H, m, C$\mathbf{H}_2CF_3$), 4.08 (1H, d, *J* = 18.0 Hz, =CC$\mathbf{H}_a H_b$), 3.81 (3H, s, OC$\mathbf{H}_3$), 3.51 (1H, d, *J* = 18.0 Hz, =CC$H_a\mathbf{H}_b$), 2.34 (3H, s, ArC$\mathbf{H}_3$); ^{13}C NMR (101 MHz, $CDCl_3$) δ 200.1 (C), 169.7 (C), 165.8 (C), 159.2 (C), 141.3 (C), 136.8 (C), 132.0 (C), 131.7 (C), 129.9 (C), 129.7 (2 × CH), 129.4 (2 × CH), 128.8 (2 × CH), 128.7 (2 × CH), 128.4 (2 × CH), 128.3 (CH), 122.8 (q, J_{C-F} = 277.9 Hz, C), 114.2 (2 × CH), 62.4 (C), 61.2 (q, J_{C-F} = 36.7 Hz, CH_2), 55.4 (CH_3), 43.6 (CH_2), 21.5 (CH_3); ^{19}F NMR (376 MHz, $CDCl_3$) δ –73.5 (t, *J* = 8.2 Hz, 3 × F); HRMS (ESI) Exact mass calculated for $[C_{28}H_{23}F_3NaO_4]^+$ $[M+Na]^+$: 503.1441, found: 503.1438. Enantiomeric excess was determined by HPLC with Chiralpak AD-H column (90:10 *iso*-hexane:*i*-PrOH, 1.0 mL/min, 230 nm, 25 °C); t_r (major) = 26.4 min, t_r (minor) = 29.8 min, 82% ee.

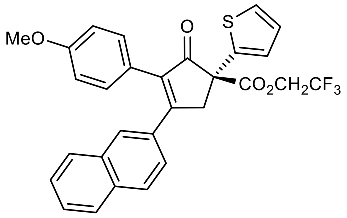

2,2,2-Trifluoroethyl (*R*)-3-(4-methoxyphenyl)-4-(naphthalen-2-yl)-2-oxo-1-(2-thienyl)cyclopent-3-ene-1-carboxylate (3ii). The title compound was prepared according to General Procedure F, using malonate ester **1i** (148.3 mg, 0.30 mmol) and 2-napthyleneboronic acid (103.2 mg, 0.60 mmol) at 80 °C, and purified by column chromatography (0% to 10% EtOAc/*n*-pentane) to give a yellow oil (136.8 mg, 87%). R_f = 0.40 (20% EtOAc/petroleum ether); IR 2963, 1764 (C=O), 1702 (C=O), 1602, 1509, 1283, 1248, 1159, 820, 730 cm^{-1}; $[\alpha]_D^{21}$ –7.4 (*c* 0.54, $CHCl_3$); ^1H NMR (400 MHz, $CDCl_3$) δ 8.01 (1H, d, *J* = 1.8 Hz, Ar**H**), 7.84-7.80 (2H, m, Ar**H**), 7.71 (1H, d, *J* = 8.6 Hz, Ar**H**), 7.57-7.50 (2H, m, Ar**H**), 7.38-7.33 (3H, m, Ar**H**), 7.27-7.22 (2H, m, Ar**H**), 7.07-7.04 (1H, m, Ar**H**), 6.90-6.86 (2H, m, Ar**H**), 4.67-4.49 (2H, m, C$\mathbf{H}_2CF_3$), 4.20 (1H, d, *J* = 17.9 Hz, =CC$\mathbf{H}_a H_b$), 3.82 (3H, s, OC$\mathbf{H}_3$), 3.78 (1H, d, *J* = 17.9 Hz, =CC$H_a\mathbf{H}_b$); ^{13}C NMR (101 MHz, $CDCl_3$) δ 199.1 (C), 168.7 (C), 164.9 (C), 159.9 (C), 139.2 (C), 136.2 (C), 134.2 (C), 133.1 (C), 132.4 (C), 131.1 (2 × CH), 128.9 (CH), 128.3 (CH), 128.2 (CH), 127.9 (CH), 127.8 (C), 126.92 (CH), 126.89 (CH), 126.84 (CH), 125.9 (CH), 125.5 (CH), 123.5 (C), 122.7 (q, J_{C-F} = 277.9 Hz, C), 114.2 (2 × CH), 61.6 (q, J_{C-F} = 36.9 Hz, CH_2), 59.9 (C), 55.4 (CH_3), 44.4 (CH_2); ^{19}F NMR (376 MHz, $CDCl_3$)

δ −73.6 (t, J = 8.2 Hz, 3 × F); HRMS (ESI) exact mass calculated for $[C_{29}H_{21}F_3NaO_4S]^+$ $[M+Na]^+$: 545.1005, found: 545.1009. Enantiomeric excess was determined by HPLC with Chiralpak AD-H column (90:10 *iso*-hexane:*i*-PrOH, 1.0 mL/min, 254 nm, 25 °C); t_r (minor) = 25.6 min, t_r (major) = 31.9 min, 92% ee.

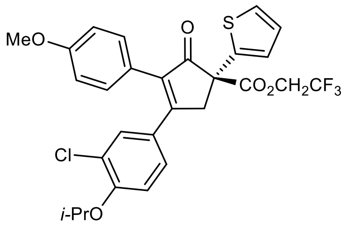

2,2,2-Trifluoroethyl (*R*)-4-(3-chloro-4-isopropoxyphenyl)-3-(4-methoxyphenyl)-2-oxo-1-(2-thienyl)cyclopent-3-ene-1-carboxylate (3ij). The title compound was prepared according to General Procedure F, using malonate ester **1i** (148.3 mg, 0.30 mmol) and 3-chloro-4-isopropoxyphenylboronic acid (128.7 mg, 0.60 mmol) at 80 °C, and purified by column chromatography (10% EtOAc/*n*-pentane) to give a yellow oil (149.3 mg, 88%). R_f = 0.12 (10% EtOAc/petroleum ether); IR 2978, 1764 (C=O), 1703 (C=O), 1594, 1511, 1494, 1278, 1248, 1158, 730 cm^{-1}; $[\alpha]_D^{21}$ −45.5 (c 0.44, $CHCl_3$); ^{1}H NMR (400 MHz, $CDCl_3$) δ 7.49 (1H, d, J = 2.3 Hz, Ar**H**), 7.32 (1H, dd, J = 5.1, 1.2 Hz, Ar**H**), 7.29-7.24 (2H, m, Ar**H**), 7.22-7.18 (2H, m, Ar**H**), 7.03 (1H, dd, J = 5.2, 3.7 Hz, Ar**H**), 6.93-6.89 (2H, m, Ar**H**), 6.83 (1H, d, J = 8.8 Hz, Ar**H**), 4.64-4.47 (3H, m, C**H**$(CH_3)_2$ and C**H**$_2CF_3$), 4.02 (1H, d, J = 17.7 Hz, =CC**H**$_a$H$_b$), 3.83 (3H, s, OC**H**$_3$), 3.60 (1H, d, J = 17.7 Hz, =CCH$_a$**H**$_b$), 1.39 (6H, d, J = 6.1 Hz, CH(C**H**$_3$)$_2$); ^{13}C NMR (101 MHz, $CDCl_3$) δ 198.8 (C), 168.6 (C), 163.1 (C), 159.9 (C), 155.6 (C), 139.2 (C), 135.3 (C), 130.9 (2 × CH), 130.4 (CH), 128.4 (CH), 127.4 (C), 126.9 (CH), 126.8 (CH), 125.8 (CH), 124.1 (C), 123.6 (C), 122.7 (q, J_{C-F} = 277.5 Hz, C), 114.4 (2 × CH), 114.2 (CH), 72.0 (CH), 61.5 (q, J_{C-F} = 37.0 Hz, CH_2), 59.7 (C), 55.4 (CH_3), 43.9 (CH_2), 22.1 (2 × CH_3); ^{19}F NMR (376 MHz, $CDCl_3$) δ −73.6 (t, J = 8.3 Hz, 3 × F); HRMS (ESI) exact mass calculated for $[C_{28}H_{24}ClF_3NaO_5S]^+$ $[M+Na]^+$: 587.0877, found 587.0880. Enantiomeric excess was determined by HPLC with Chiralpak AD-H column (90:10 *iso*-hexane:*i*-PrOH, 1.0 mL/min, 254 nm, 25 °C); t_r (minor) = 19.7 min, t_r (major) = 22.9 min, 89% ee.

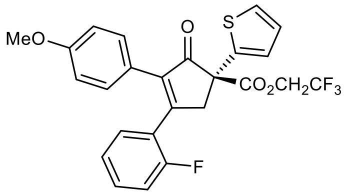

2,2,2-Trifluoroethyl (*R*)-4-(2-fluorophenyl)-3-(4-methoxyphenyl)-2-oxo-1-(2-thienyl)cyclopent-3-ene-1-carboxylate (3ik). The title compound was prepared according to General Procedure F, using malonate ester **1i** (148.3 mg, 0.30 mmol) and 2-fluorophenylboronic acid (84.0 mg, 0.60 mmol) at 80 °C, and purified by column chromatography (10% EtOAc/*n*-pentane) to give a yellow oil (125.5 mg, 85%). R_f = 0.43 (20% EtOAc/petroleum ether); IR 2960, 1765 (C=O), 1708 (C=O), 1603, 1575, 1512, 1283, 1249, 1158, 759 cm^{-1}; $[\alpha]_D^{22}$ −19.0 (c 0.42, $CHCl_3$); ^{1}H NMR (400 MHz, $CDCl_3$) δ 7.40-7.36 (1H, m, Ar**H**), 7.33 (1H, dd, J = 5.2, 1.2 Hz,

ArH), 7.30 (1H, dd, J = 3.7, 1.2 Hz, ArH), 7.20-7.03 (6H, m, ArH), 6.83-6.80 (2H, m, ArH), 4.67-4.58 (1H, m, $CH_aH_bCF_3$), 4.55-4.45 (1H, m, $CH_aH_bCF_3$), 4.03 (1H, dd, J = 18.5, 0.8 Hz, $=CCH_aH_b$), 3.78 (3H, s, OCH_3), 3.70 (1H, dd, J = 18.5, 1.0 Hz, $=CCH_aH_b$); ^{13}C NMR (101 MHz, $CDCl_3$) δ 198.9 (C), 168.5 (C), 161.7 (C), 159.9 (d, J_{C-F} = 251.7 Hz, C), 159.8 (C), 138.7 (C), 137.8 (C), 131.8 (d, J_{C-F} = 8.2 Hz, CH), 130.6 (2 × CH), 130.1 (d, J_{C-F} = 3.5 Hz, CH), 126.9 (CH), 126.8 (CH), 126.0 (CH), 124.5 (d, J_{C-F} = 3.1 Hz, CH), 123.5 (d, J_{C-F} = 14.5 Hz, C), 122.9 (C), 122.7 (q, J_{C-F} = 277.3 Hz, C), 116.7 (d, J_{C-F} = 21.8 Hz, CH), 114.0 (2 × CH), 61.6 (q, J_{C-F} = 36.8 Hz, CH_2), 60.0 (C), 55.3 (CH_3), 44.9 (d, J_{C-F} = 4.9 Hz, CH_2); ^{19}F NMR (376 MHz, $CDCl_3$) δ −73.7 (t, J = 8.2 Hz, 3 × F), −110.7 (dt, J = 11.5, 6.1 Hz, F); HRMS (ESI) exact mass calculated for $[C_{25}H_{18}F_4NaO_4S]^+$ $[M+Na]^+$: 513.0754, found: 513.0750. Enantiomeric excess was determined by HPLC with Chiralpak AD-H column (90:10 *iso*-hexane:*i*-PrOH, 1.0 mL/min, 254 nm, 25 °C); t_r (minor) = 17.8 min, t_r (major) = 28.2 min, 94% ee.

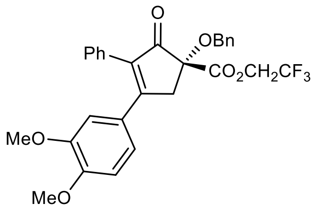

2,2,2-Trifluoroethyl (*R*)-1-(benzyloxy)-4-(3,4-dimethoxyphenyl)-2-oxo-3-phenylcyclopent-3-ene-1-carboxylate (3nl). The title compound was prepared according to General Procedure F, using malonate ester **1n** (146.5 mg, 0.30 mmol) and 3,4-dimethoxyphenylboronic acid (109.2 mg, 0.60 mmol) at 80 °C, and purified by column chromatography (0% to 10% EtOAc/*n*-pentane) to give a pale yellow oil (120.6 mg, 76%). R_f = 0.23 (30% EtOAc/petroleum ether); IR 3024, 1771 (C=O), 1699 (C=O), 1596, 1517, 1267, 1155, 1022, 749, 697 cm^{-1}; $[\alpha]_D^{22}$ +16.0 (*c* 1.00, $CHCl_3$); ^{1}H NMR (500 MHz, $CDCl_3$) δ 7.45-7.39 (4H, m, ArH), 7.37-7.34 (3H, m, ArH), 7.32-7.28 (1H, m, ArH), 7.26-7.24 (2H, m, ArH), 7.07 (1H, dd, J = 8.5, 2.2 Hz, ArH), 6.83 (1H, d, J = 8.5 Hz, ArH), 6.78 (1H, d, J = 2.1 Hz, ArH), 4.96 (1H, d, J = 11.2 Hz, OCH_aH_bPh), 4.92 (1H, d, J = 11.2 Hz, OCH_aH_bPh), 4.73 (1H, dq, J = 12.6, 8.3 Hz, $CH_aH_bCF_3$), 4.56 (1H, dq, J = 12.6, 8.2 Hz, $CH_aH_bCF_3$), 3.89 (3H, s, OCH_3), 3.54 (1H, d, J = 17.7 Hz, $=CCH_aH_b$), 3.43 (3H, s, OCH_3), 3.36 (1H, d, J = 17.7 Hz, $=CCH_aH_b$); ^{13}C NMR (126 MHz, $CDCl_3$) δ 198.4 (C), 169.1 (C), 165.0 (C), 151.7 (C), 148.5 (C), 137.8 (C), 135.8 (C), 132.3 (C), 129.7 (2 × CH), 129.0 (2 × CH), 128.6 (2 × CH), 128.5 (CH), 128.3 (2 × CH), 128.0 (CH), 126.6 (C), 122.7 (q, J_{C-F} = 277.2 Hz, C), 122.1 (CH), 112.0 (CH), 110.8 (CH), 82.9 (C), 69.4 (CH_2), 61.1 (q, J_{C-F} = 37.1 Hz, CH_2), 56.1 (CH_3), 55.4 (CH_3), 42.4 (CH_2); ^{19}F NMR (376 MHz, $CDCl_3$) δ –73.6 (t, J = 8.3 Hz, 3 × F); HRMS (ESI) Exact mass calculated for $[C_{29}H_{25}F_3NaO_6]^+$ $[M+Na]^+$: 549.1495, found 549.1479. Enantiomeric excess was determined by HPLC using a Chiralpak AD-H column (90:10 *iso*-hexane:*i*-PrOH, 1.0 mL/min, 210 nm, 25 °C); t_r (major) = 20.5 min, t_r (minor) = 36.7 min, 90% ee.

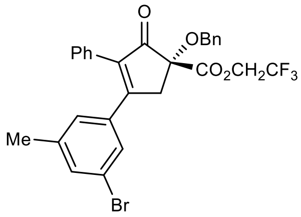

2,2,2-Trifluoroethyl (*R*)-1-(benzyloxy)-4-(3-bromo-5-methylphenyl)-2-oxo-3-phenylcyclopent-3-ene-1-carboxylate (3nm). The title compound was prepared according to General Procedure F, using malonate ester **1n** (146.5 mg, 0.30 mmol) and 3-bromo-5-methylphenylboronic acid (128.9 mg, 0.60 mmol) at 80 °C, and purified by column chromatography (0% to 10% EtOAc/*n*-pentane) to give a pale yellow oil (122.1 mg, 73%). R_f = 0.10 (10% EtOAc/petroleum ether); IR 3033, 1772 (C=O), 1706 (C=O), 1347, 1283, 1160, 907, 854, 729, 700 cm^{-1}; $[\alpha]_D^{21}$ +24.0 (*c* 1.00, $CHCl_3$); ^{1}H NMR (400 MHz, $CDCl_3$) δ 7.44-7.41 (2H, m, Ar**H**), 7.38-7.28 (7H, m, Ar**H**), 7.24-7.23 (1H, m, Ar**H**), 7.22-7.16 (2H, m, Ar**H**), 7.01 (1H, td, *J* = 1.5, 0.8 Hz, Ar**H**), 4.95 (1H, d, *J* = 11.2 Hz, OC$\mathbf{H}_a$H$_b$Ph), 4.90 (1H, d, *J* = 11.2 Hz, OCH$_a\mathbf{H}_b$Ph), 4.72 (1H, dq, *J* = 12.6, 8.3 Hz, C$\mathbf{H}_a$H$_b$CF$_3$), 4.57 (1H, dq, *J* = 12.6, 8.2 Hz, CH$_a\mathbf{H}_b$CF$_3$), 3.48 (1H, d, *J* = 17.9 Hz, =CC$\mathbf{H}_a$H$_b$), 3.30 (1H, d, *J* = 18.0 Hz, =CCH$_a\mathbf{H}_b$), 2.22 (3H, s, C$\mathbf{H}_3$); ^{13}C NMR (101 MHz, $CDCl_3$) δ 198.4 (C), 168.7 (C), 164.2 (C), 140.5 (C), 137.8 (C), 137.6 (C), 136.2 (C), 134.3 (CH), 130.7 (C), 129.4 (2 × CH), 128.84 (CH), 128.77 (2 × CH), 128.6 (2 × CH), 128.3 (CH), 128.2 (2 × CH), 128.1 (CH), 127.7 (CH), 122.7 (q, $J_{C\text{-}F}$ = 277.8 Hz, C), 122.6 (C), 83.0 (C), 69.4 (CH_2), 61.1 (q, $J_{C\text{-}F}$ = 37.2 Hz, CH_2), 42.8 (CH_2), 21.2 (CH_3); ^{19}F NMR (376 MHz, $CDCl_3$) δ –73.6 (t, *J* = 8.2 Hz, 3 × F); HRMS (ESI) Exact mass calculated for $[C_{28}H_{22}BrF_3NaO_4]^+$ $[M+Na]^+$: 581.0546, found 581.0547. Enantiomeric excess was determined by HPLC using a Chiralpak AD-H column (90:10 *iso*-hexane:*i*-PrOH, 1.0 mL/min, 254 nm, 25 °C); t_r (major) = 8.6 min, t_r (minor) = 9.8 min, 94% ee.

4. Further Transformations of Cyclopent-2-enone 3ik

(*R*)-*N*-Benzyl-4-(2-fluorophenyl)-3-(4-methoxyphenyl)-2-oxo-1-(2-thienyl)cyclopent-3-ene-1-carboxamide (8)

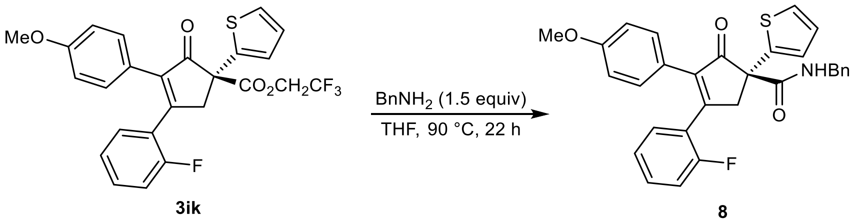

An oven-dried microwave vial fitted with a stirrer bar was charged with ester **3ik** (95.0 mg, 0.19 mmol) and benzylamine (31.1 mg, 0.29 mmol). The vial was capped with a crimp cap PTFE seal and evacuated and back filled with argon gas (3 × cycles). Anhydrous THF (2.5 mL) was added and the mixture was stirred 90 °C for 22 h. The reaction was cooled to room temperature and diluted with saturated aqueous NH_4Cl solution (2 mL) and H_2O (2 mL). The mixture was extracted with EtOAc (3 × 4 mL) and the combined organic layers were washed with brine (2 mL), dried

($MgSO_4$), filtered and concentrated under reduced pressure. The residue was purified by column chromatography (0% to 10% EtOAc/*n*-pentane) to give the title compound **8** as a colorless oil (81.4 mg, 84%). R_f = 0.11 (9% EtOAc/petroleum ether); IR 3352, 2929, 1693 (C=O), 1670 (C=O), 1510, 1451, 1354, 1178, 759, 699 cm^{-1}; $[\alpha]_D^{21}$ −76.0 (*c* 1.00, $CHCl_3$); ^1H NMR (400 MHz, $CDCl_3$) δ 7.38-7.15 (12H, m, Ar**H**), 7.10-7.06 (2H, m, Ar**H**), 6.98 (1H, dd, *J* = 5.1, 3.6 Hz, Ar**H**), 6.85-6.81 (2H, m, Ar**H**), 4.56-4.40 (3H, m, C**H**$_2$Ph and =CC**H**$_a$H$_b$), 3.79 (3H, s, OC**H**$_3$), 3.47 (1H, d, *J* = 18.8 Hz, =CCH$_a$**H**$_b$); ^{13}C NMR (101 MHz, $CDCl_3$) δ 203.9 (C), 168.1 (C), 163.8 (C), 159.8 (d, J_{C-F} = 253.5 Hz, C), 159.7 (C), 143.2 (C), 137.9 (C), 137.8 (C), 131.8 (d, J_{C-F} = 8.4 Hz, CH), 130.5 (2 × CH), 130.2 (d, J_{C-F} = 3.3 Hz, CH), 128.8 (2 × CH), 127.5 (2 × CH), 127.0 (CH), 125.6 (CH), 125.3 (CH), 124.4 (d, J_{C-F} = 3.5 Hz, CH), 123.7 (d, J_{C-F} = 14.0 Hz, C), 123.3 (C), 116.6 (d, J_{C-F} = 21.8 Hz, CH), 114.0 (2 × CH), 60.7 (C), 55.3 (CH_3), 45.1 (d, J_{C-F} = 4.2 Hz, CH_2), 44.2 (CH_2); ^{19}F NMR (376 MHz, $CDCl_3$) δ –109.5 (app dt, *J* = 11.4, 6.1 Hz, F); HRMS (ESI) exact mass calculated for $[C_{30}H_{24}FNNaO_3S]^+$ $[M+Na]^+$: 520.1353, found: 520.1340.

(1*R*,2*S*)-*N*-Benzyl-4-(2-fluorophenyl)-2-hydroxy-3-(4-methoxyphenyl)-1-(2-thienyl)cyclopent-3-ene-1-carboxamide (9)

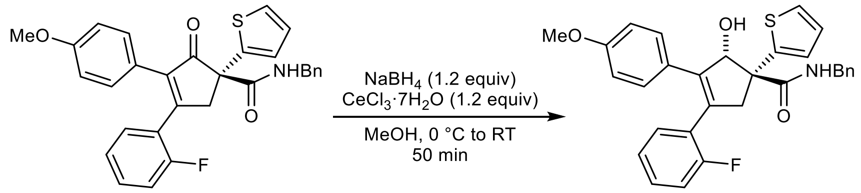

8 **9**

To a solution of amide **8** (68.0 mg, 0.14 mmol) in anhydrous MeOH (1.2 mL) 0 °C was $CeCl_3·7H_2O$ (50.9 mg, 0.16 mmol). $NaBH_4$ (6.2 mg, 0.16 mmol) was then added in portions over 2 min, and the mixture was stirred 0 °C for 20 min. The mixture was warmed to room temperature and stirred for 30 min, and quenched carefully with H_2O (2 mL). The mixture was diluted with brine (1 mL) and saturated aqueous NH_4Cl solution (1 mL), and extracted with EtOAc (5 × 3 mL). The combined organic layers were dried ($MgSO_4$), filtered and concentrated under reduced pressure to give the title compound **9** as a white solid (57.1 mg, 83%). R_f = 0.26 (40% EtOAc/petroleum ether); m.p. 67-68 °C (CH_2Cl_2/*n*-pentane); IR 3361, 2928, 1651 (C=O), 1510, 1451, 1245, 1177, 1031, 758, 698 cm^{-1}; $[\alpha]_D^{25}$ +208.0 (*c* 0.50, $CHCl_3$); ^1H NMR (400 MHz, $CDCl_3$) δ 7.35-7.15 (10H, m, Ar**H**), 7.07 (1H, dd, *J* = 3.6, 1.2 Hz, Ar**H**), 7.02 (2H, td, *J* = 8.2, 1.4 Hz, Ar**H**), 6.96 (1H, dd, *J* = 5.2, 3.6 Hz, Ar**H**), 6.78-6.74 (2H, m, Ar**H**), 6.68 (1H, t, *J* = 5.8 Hz, N**H**), 5.69-5.67 (1H, m, C**H**OH), 4.61-4.51 (2H, m, PhC**H**$_2$), 3.75 (3H, s, OC**H**$_3$), 3.45 (2H, s, =CC**H**$_2$), 2.06-2.03 (1H, br m, O**H**); ^{13}C NMR (101 MHz, $CDCl_3$) δ 173.4 (C), 160.3 (d, J_{C-F} = 248.6 Hz, C), 159.1 (C), 142.1 (C),

140.5 (C), 138.2 (C), 131.1 (d, $J_{C\text{-}F}$ = 4.1 Hz, CH), 130.8 (C), 129.9 (2 × CH), 129.5 (d, $J_{C\text{-}F}$ = 8.1 Hz, CH), 128.8 (2 × CH), 127.9 (2 × CH), 127.6 (CH), 127.4 (CH), 126.9 (CH), 125.3 (CH), 124.8 (d, $J_{C\text{-}F}$ = 15.0 Hz, C), 124.2 (d, $J_{C\text{-}F}$ = 3.4 Hz, CH), 116.1 (d, $J_{C\text{-}F}$ = 22.1 Hz, CH), 113.8 (2 × CH), 82.3 (CH), 60.6 (C), 55.3 (CH_3), 46.1 (d, $J_{C\text{-}F}$ = 2.9 Hz, CH_2), 43.9 (CH_2); ^{19}F NMR (376 MHz, $CDCl_3$) δ –112.2 (app dt, J = 11.1, 6.3 Hz, F); HRMS (ESI) exact mass calculated for $[C_{30}H_{26}FNNaO_3S]^+$ $[M+Na]^+$: 522.1510, found: 522.1506.

The configuration at the newly formed stereocenter of **9** was assigned using NOESY NMR spectroscopy (see next page).

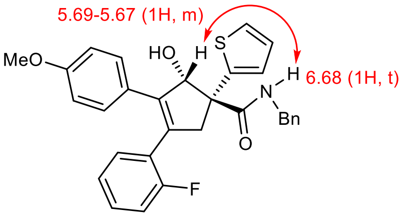

9

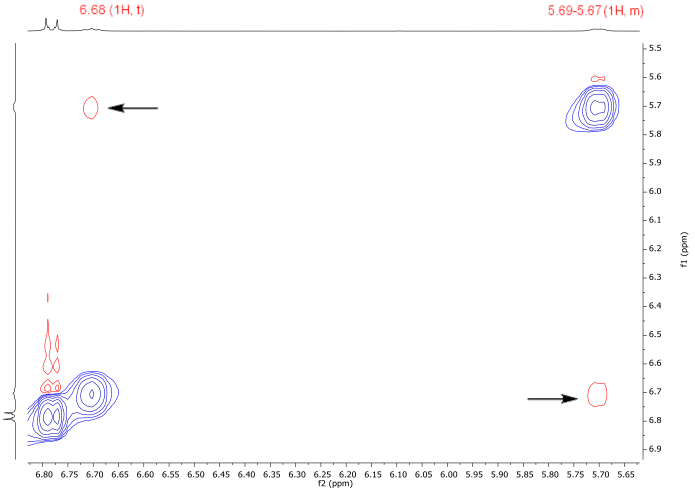

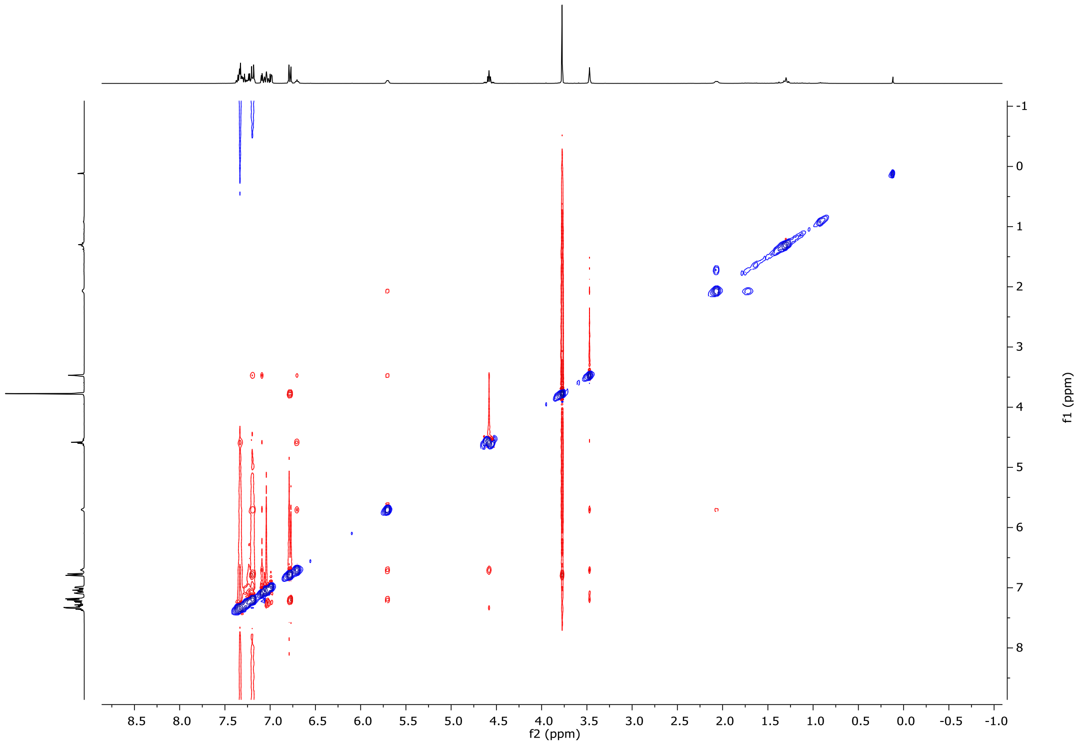

5. Nickel-Catalyzed Arylative Cyclization of Substrate 10

4,5-Diphenyl-1-tosyl-1,6-dihydropyridin-3(2*H*)-one (11)

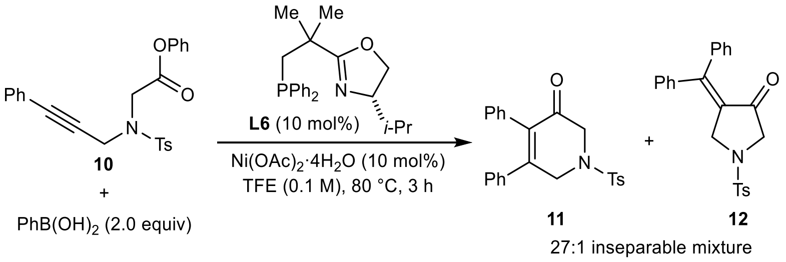

An oven-dried microwave vial fitted with a stirrer bar was charged with phenoxy ester **10** (125.8 mg, 0.30 mmol), phenylboronic acid (73.1 mg, 0.60 mmol), $Ni(OAc)_2·4H_2O$ (7.5 mg, 0.03 mmol) and (*S*)-*i*-Pr-NeoPHOX (**L6**, 10.6 mg, 0.03 mmol). The vial was capped with a crimp cap PTFE seal and evacuated and back filled with argon (3 cycles). TFE (3 mL) which had been freshly degassed (using 5 freeze-pump-thaw cycles) was added under argon flow, the septum was resealed with a layer of vacuum grease, and the contents were stirred at room temperature for 10 min and then at 80 °C for 3 h. The reaction was cooled to room temperature, diluted with EtOAc (5 mL) and washed with brine (10 mL). The aqueous layer was extracted with EtOAc (5 mL). The combined organic layers were dried (Na_2SO_4), filtered and concentrated under reduced pressure. ^{1}H NMR analysis of the crude material showed a mixture of **11** and **12** (27:1). The residue was purified by column chromatography (0% to 22% EtOAc/*n*-pentane) to give a white solid (81.9 mg, 68%). R_f = 0.42 (30% EtOAc/petroleum ether); IR 2924, 1679 (C=O), 1443, 1349, 1327, 1159, 1091, 964, 758, 700 cm^{-1}; m.p. 157-158 °C (CH_2Cl_2/petroleum ether); HRMS (ESI) Exact mass calculated for $[C_{24}H_{21}NNaO_3S]^+$ $[M+Na]^+$: 426.1134, found 426.1142.

Data for major isomer **11**: ^{1}H NMR (400 MHz, $CDCl_3$) δ 7.74 (2H, *J* = 8.3 Hz, Ar**H**), 7.35 (2H, *J* = 8.0 Hz, Ar**H**), 7.23-7.17 (3H, m, Ar**H**), 7.14-7.09 (3H, m, Ar**H**), 7.00-6.98 (2H, m, Ar**H**), 6.71-6.68 (2H, m, Ar**H**), 4.43 (2H, s, =CC**H**$_2$), 4.11 (2H, s, C**H**$_2$C=O), 2.43 (3H, s, ArC**H**$_3$); ^{13}C NMR (101 MHz, $CDCl_3$) δ 190.7 (C), 152.7 (C), 144.6 (C), 136.5 (C), 136.3 (C), 133.6 (C), 132.9 (C), 130.7 (2 × CH), 130.3 (2 × CH), 129.2 (CH), 128.52 (2 × CH), 128.49 (2 × CH), 127.8 (4 × CH), 127.6 (CH), 53.2 (CH_2), 49.8 (CH_2), 21.7 (CH_3).

Characteristic signals for minor isomer **12**: 7.69-7.67 (2H, m, Ar**H**), 6.89-6.87 (2H, m, Ar**H**), 4.35 (2H, s, =CC**H**$_2$), 3.76 (2H, s, C**H**$_2$C=O), 2.47 (3H, s, ArC**H**$_3$).

6. NMR Spectra

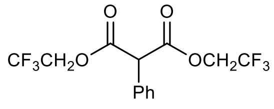

S2

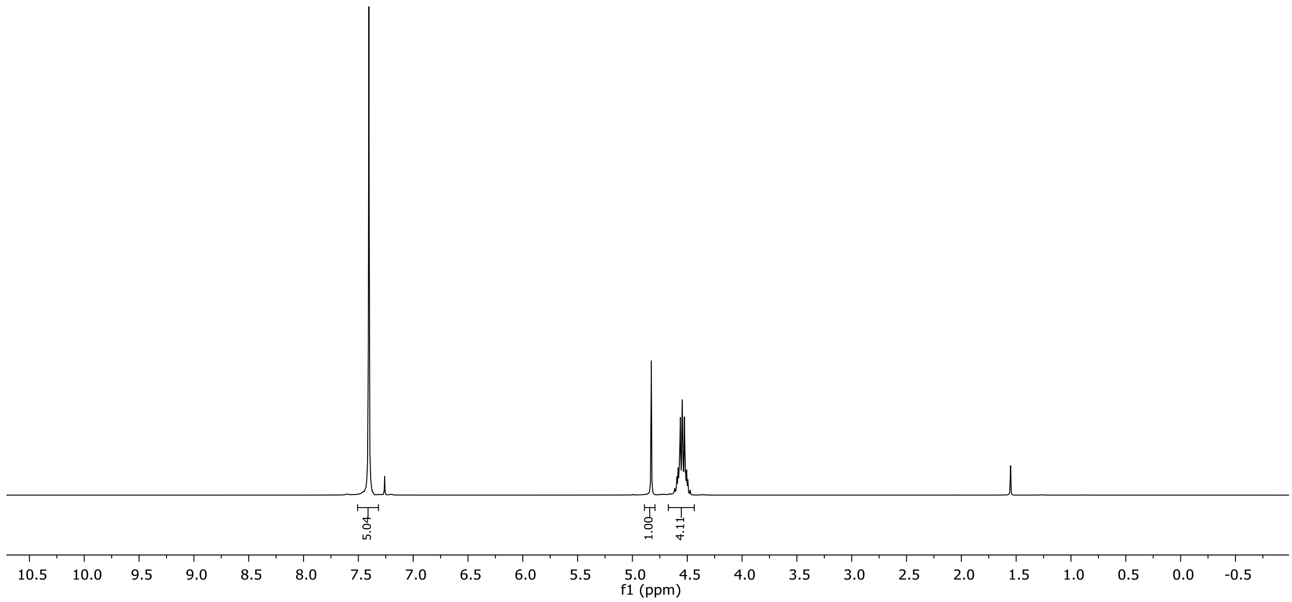

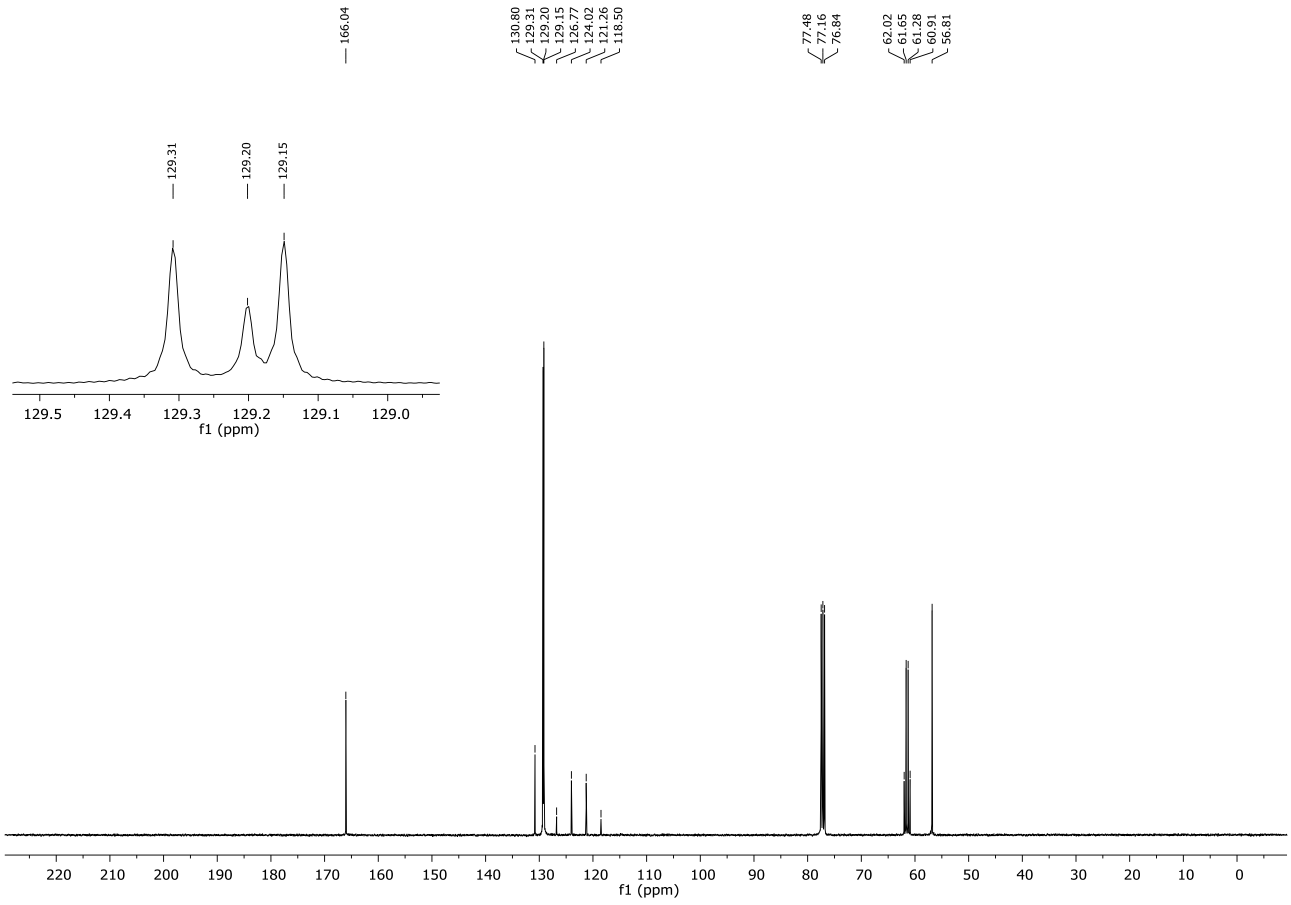

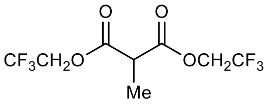
CF3CH2O
OCH2CF3
Me

S3a

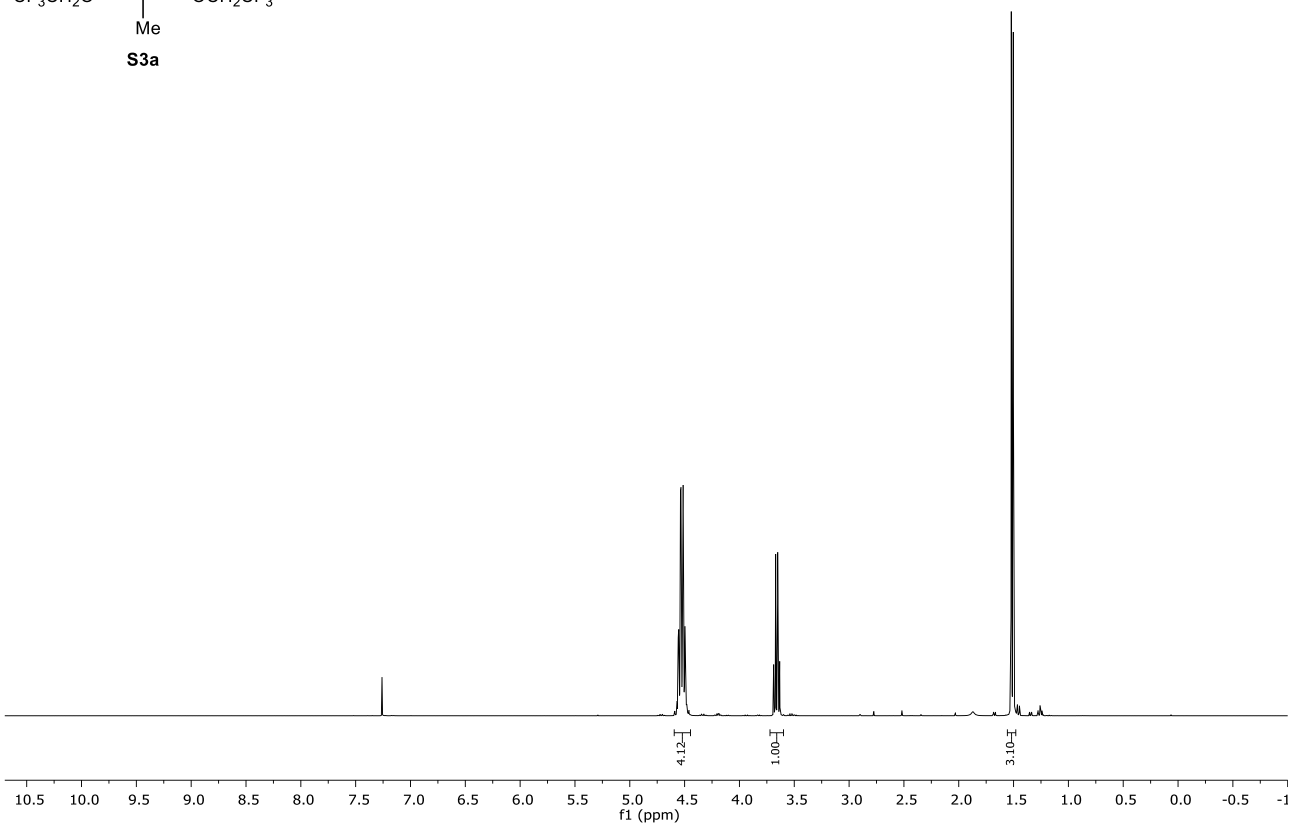
4.12
1.00
3.10
10.5 10.0 9.5 9.0 8.5 8.0 7.5 7.0 6.5 6.0 5.5 5.0 4.5 4.0 3.5 3.0 2.5 2.0 1.5 1.0 0.5 0.0 -0.5
f1 (ppm)

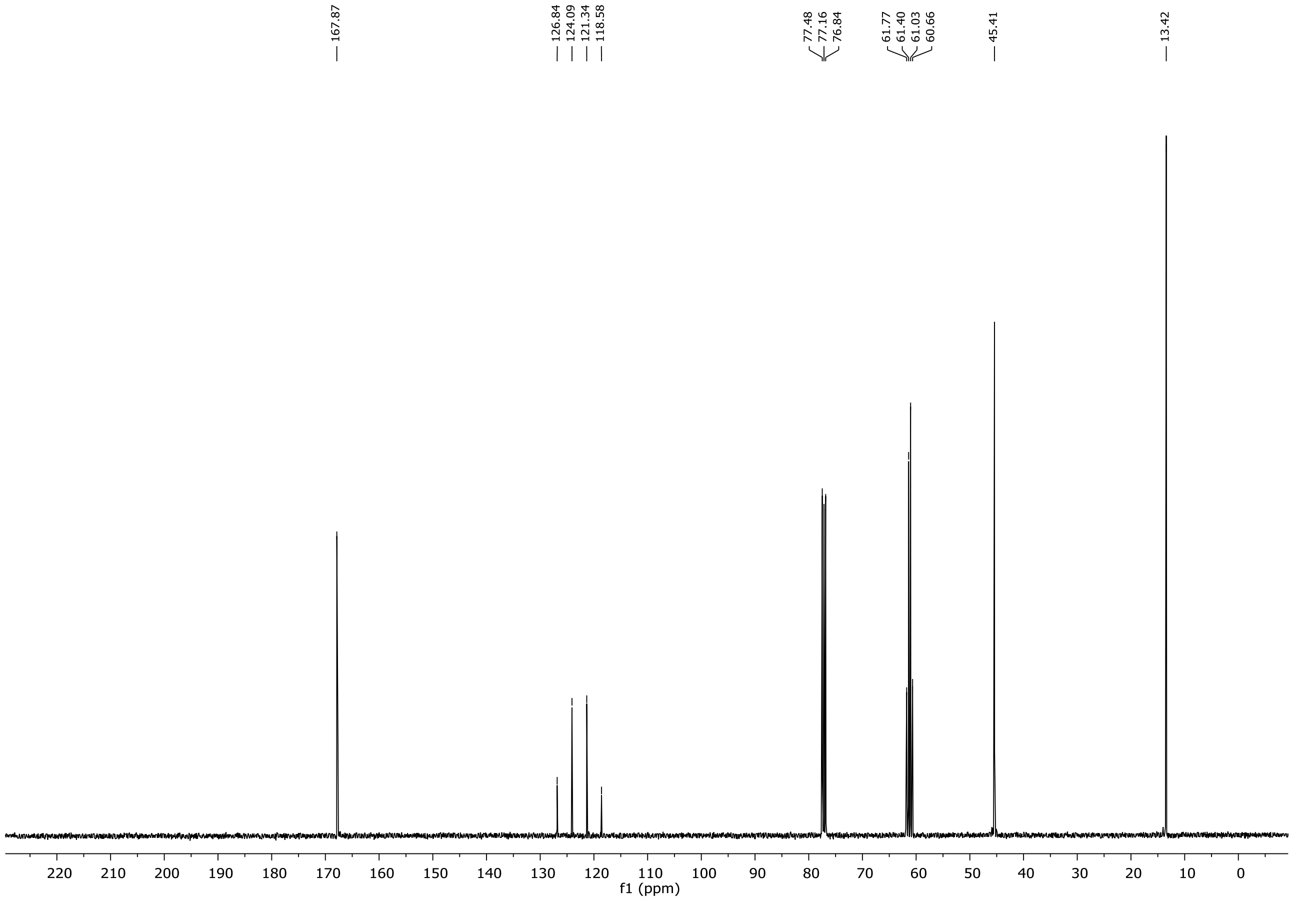
167.87
126.84
124.09
121.34
118.58
77.48
77.16
76.84
61.77
61.40
61.03
60.66
45.41
13.42
220 210 200 190 180 170 160 150 140 130 120 110 100 90 80 70 60 50 40 30 20 10 0
f1 (ppm)

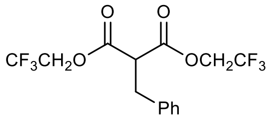
O O
CF3CH2O OCH2CF3
Ph

S3b

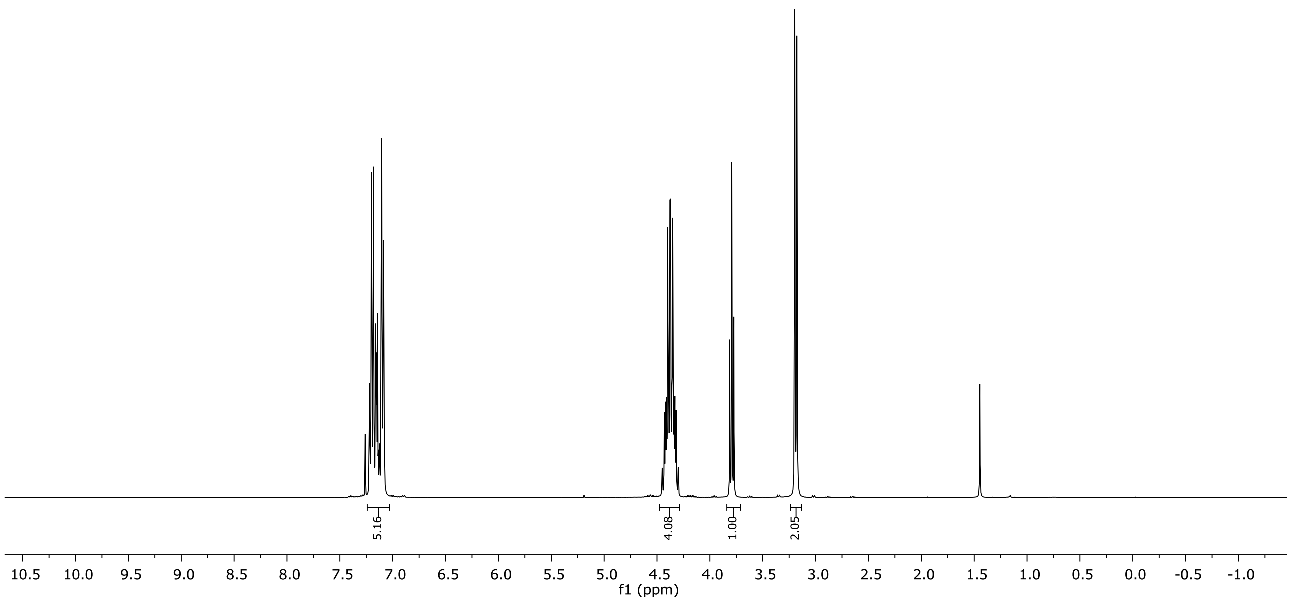
5.16
4.08
1.00
2.05
10.5 10.0 9.5 9.0 8.5 8.0 7.5 7.0 6.5 6.0 5.5 5.0 4.5 4.0 3.5 3.0 2.5 2.0 1.5 1.0 0.5 0.0 -0.5 -1.0
f1 (ppm)

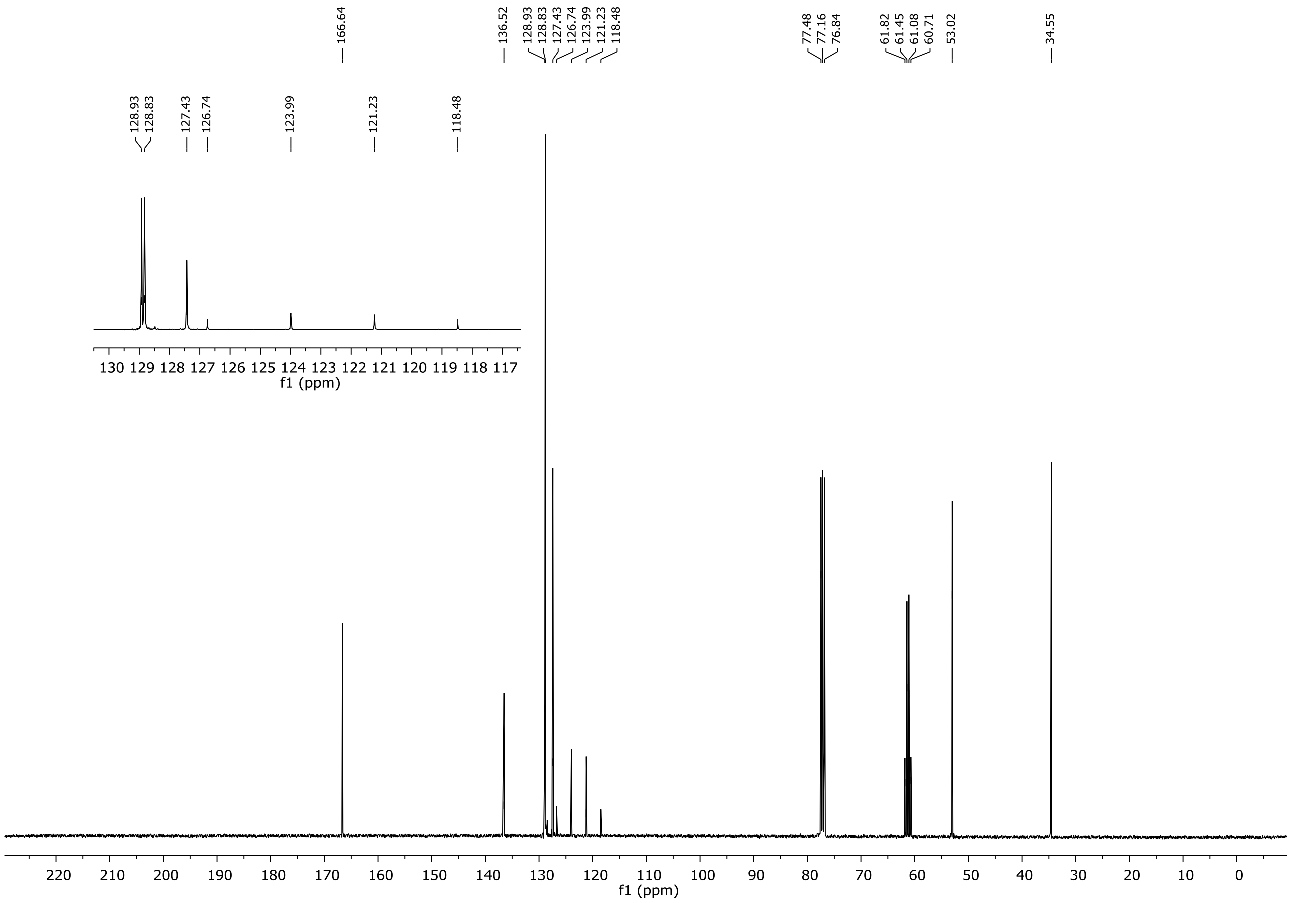
166.64
136.52
128.93
128.83
127.43
126.74
123.99
121.23
118.48
77.48
77.16
76.84
61.82
61.45
61.08
60.71
53.02
34.55
130 129 128 127 126 125 124 123 122 121 120 119 118 117
f1 (ppm)
220 210 200 190 180 170 160 150 140 130 120 110 100 90 80 70 60 50 40 30 20 10 0
f1 (ppm)

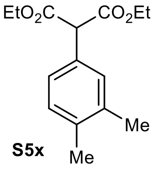
EtO2C
CO2Et
Me
Me

S5x

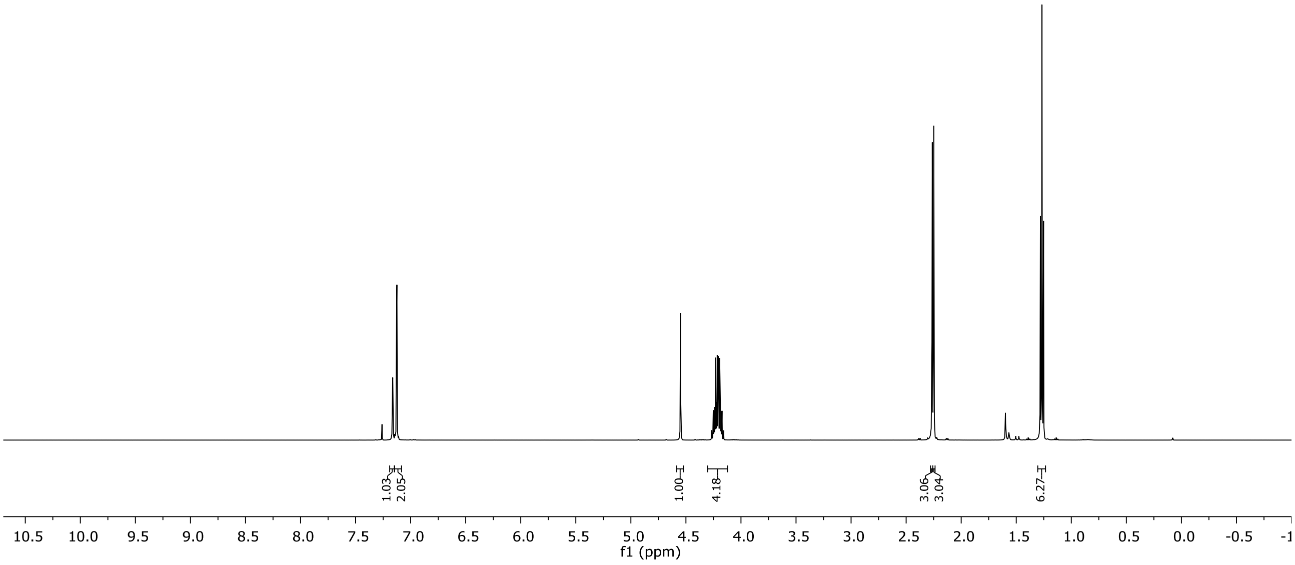
1.03
2.05
1.00
4.18
3.06
3.04
6.27
10.5 10.0 9.5 9.0 8.5 8.0 7.5 7.0 6.5 6.0 5.5 5.0 4.5 4.0 3.5 3.0 2.5 2.0 1.5 1.0 0.5 0.0 -0.5
f1 (ppm)

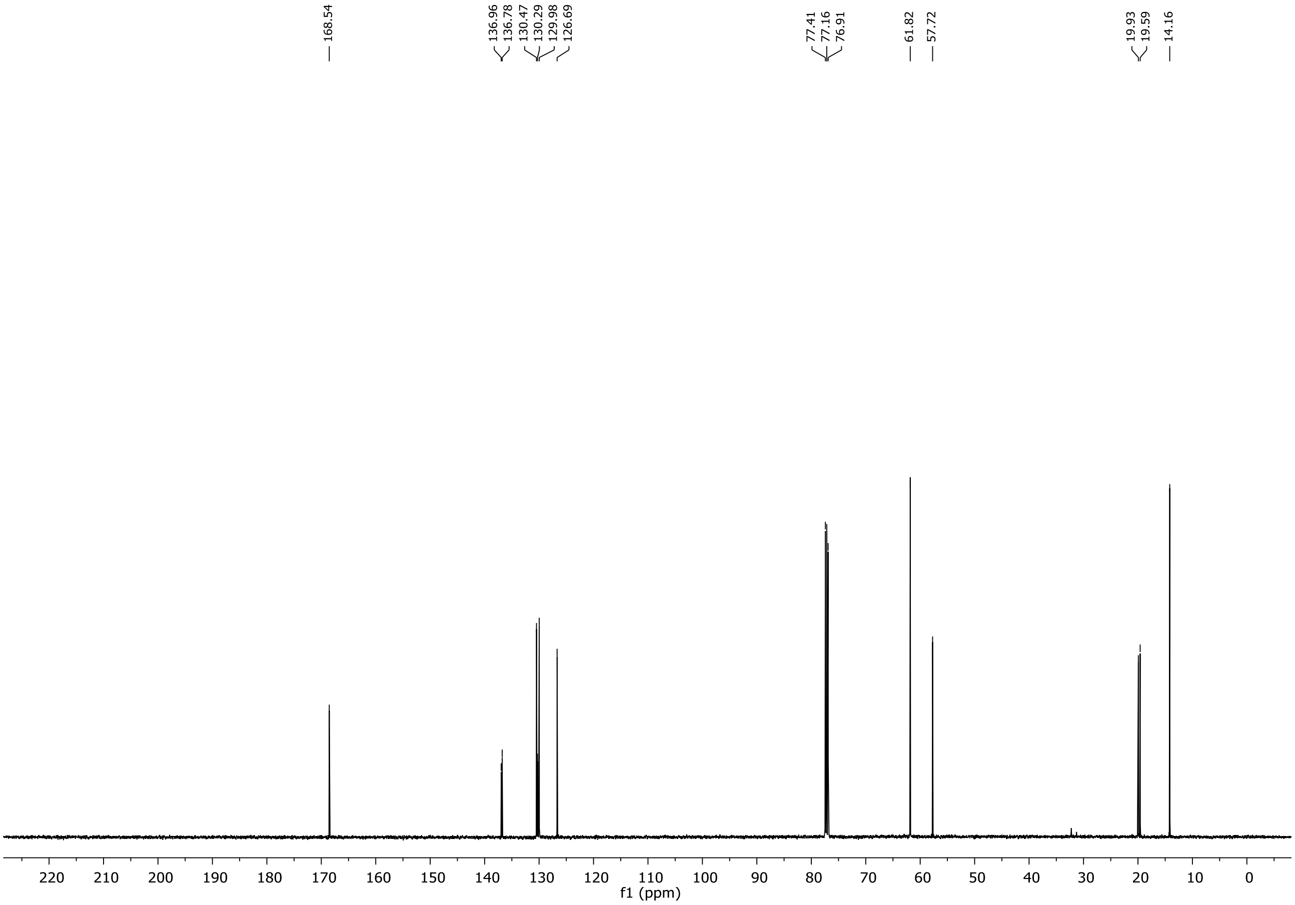
168.54
136.96
136.78
130.47
130.29
129.98
126.69
77.41
77.16
76.91
61.82
57.72
19.93
19.59
14.16
220 210 200 190 180 170 160 150 140 130 120 110 100 90 80 70 60 50 40 30 20 10 0
f1 (ppm)

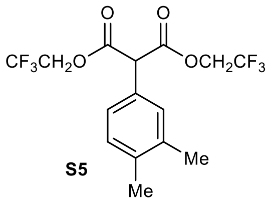
CF3CH2O
OCH2CF3
Me
Me

S5

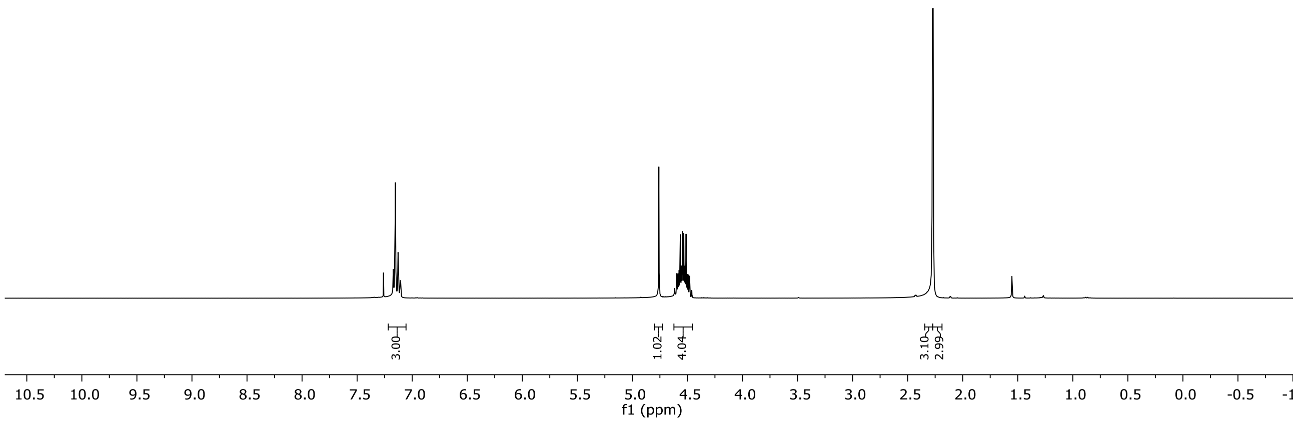
3.00
1.02
4.04
3.10
2.99
10.5 10.0 9.5 9.0 8.5 8.0 7.5 7.0 6.5 6.0 5.5 5.0 4.5 4.0 3.5 3.0 2.5 2.0 1.5 1.0 0.5 0.0 -0.5
f1 (ppm)

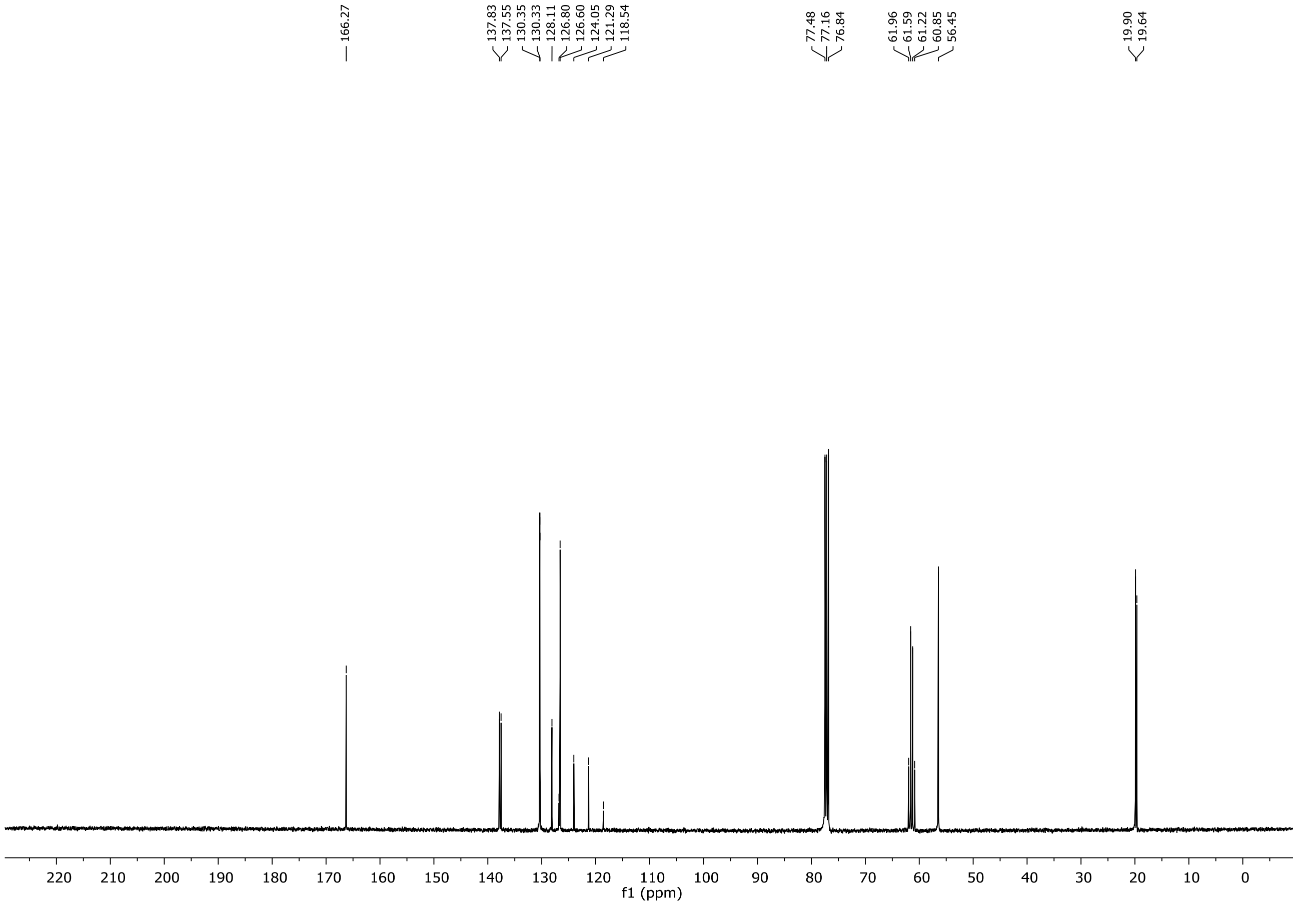
166.27
137.83
137.55
130.35
130.33
128.11
126.80
126.60
124.05
121.29
118.54
77.48
77.16
76.84
61.96
61.59
61.22
60.85
56.45
19.90
19.64
220 210 200 190 180 170 160 150 140 130 120 110 100 90 80 70 60 50 40 30 20 10 0
f1 (ppm)

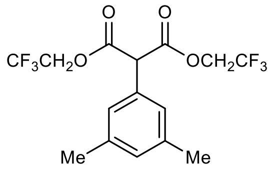
O
O
CF3CH2O
OCH2CF3
Me
Me

S6

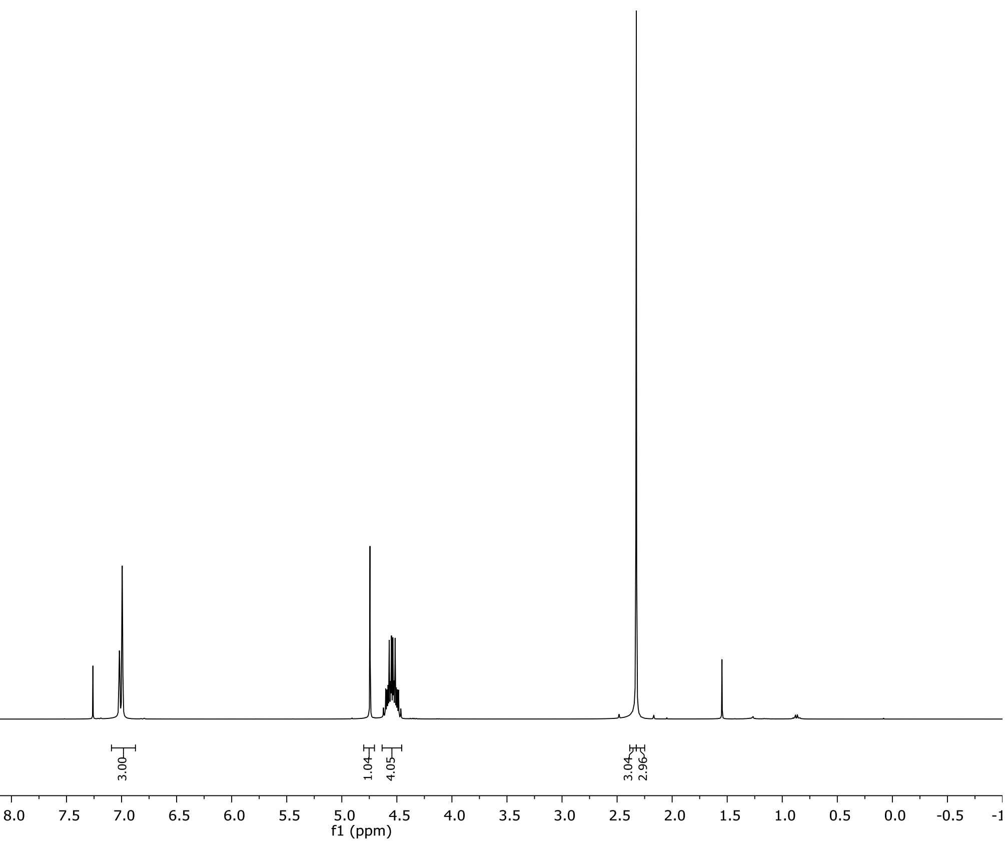
3.00
1.04
4.05
3.04
2.96
10.5 10.0 9.5 9.0 8.5 8.0 7.5 7.0 6.5 6.0 5.5 5.0 4.5 4.0 3.5 3.0 2.5 2.0 1.5 1.0 0.5 0.0 -0.5 -1
f1 (ppm)

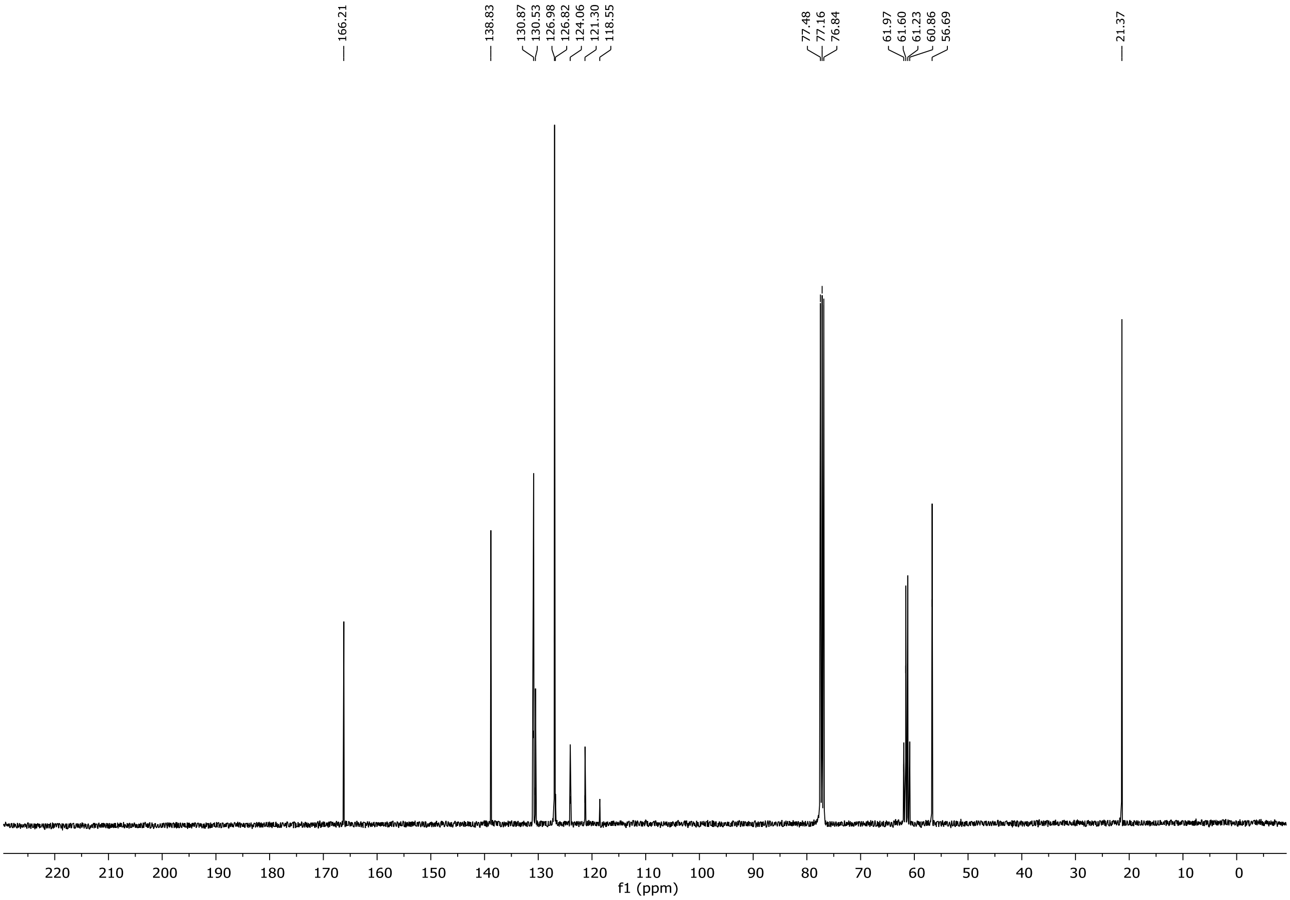
166.21
138.83
130.87
130.53
126.98
126.82
124.06
121.30
118.55
77.48
77.16
76.84
61.97
61.60
61.23
60.86
56.69
21.37
220 210 200 190 180 170 160 150 140 130 120 110 100 90 80 70 60 50 40 30 20 10 0
f1 (ppm)

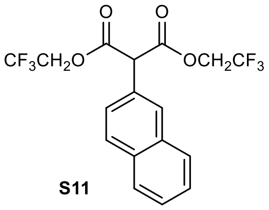

O
O
CF3CH2O
OCH2CF3

S11

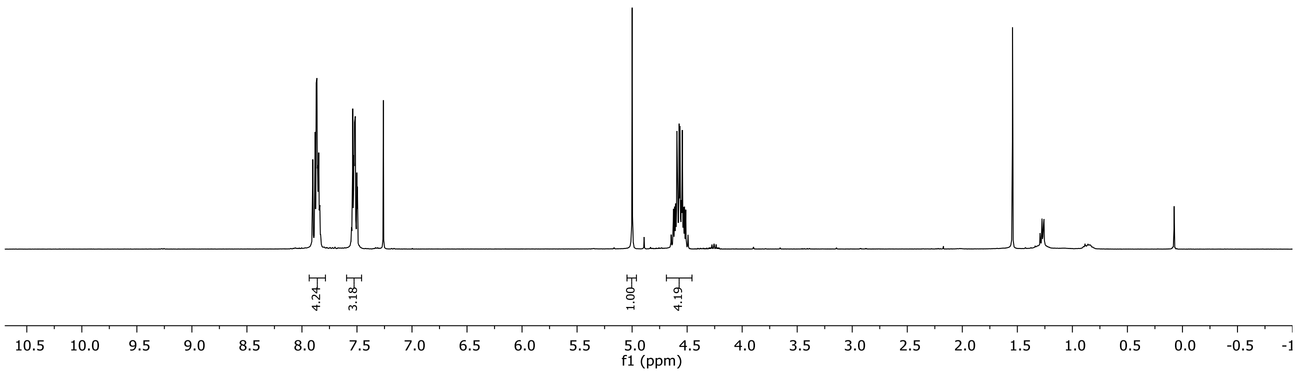

4.24
3.18
1.00
4.19
10.5 10.0 9.5 9.0 8.5 8.0 7.5 7.0 6.5 6.0 5.5 5.0 4.5 4.0 3.5 3.0 2.5 2.0 1.5 1.0 0.5 0.0 -0.5
f1 (ppm)

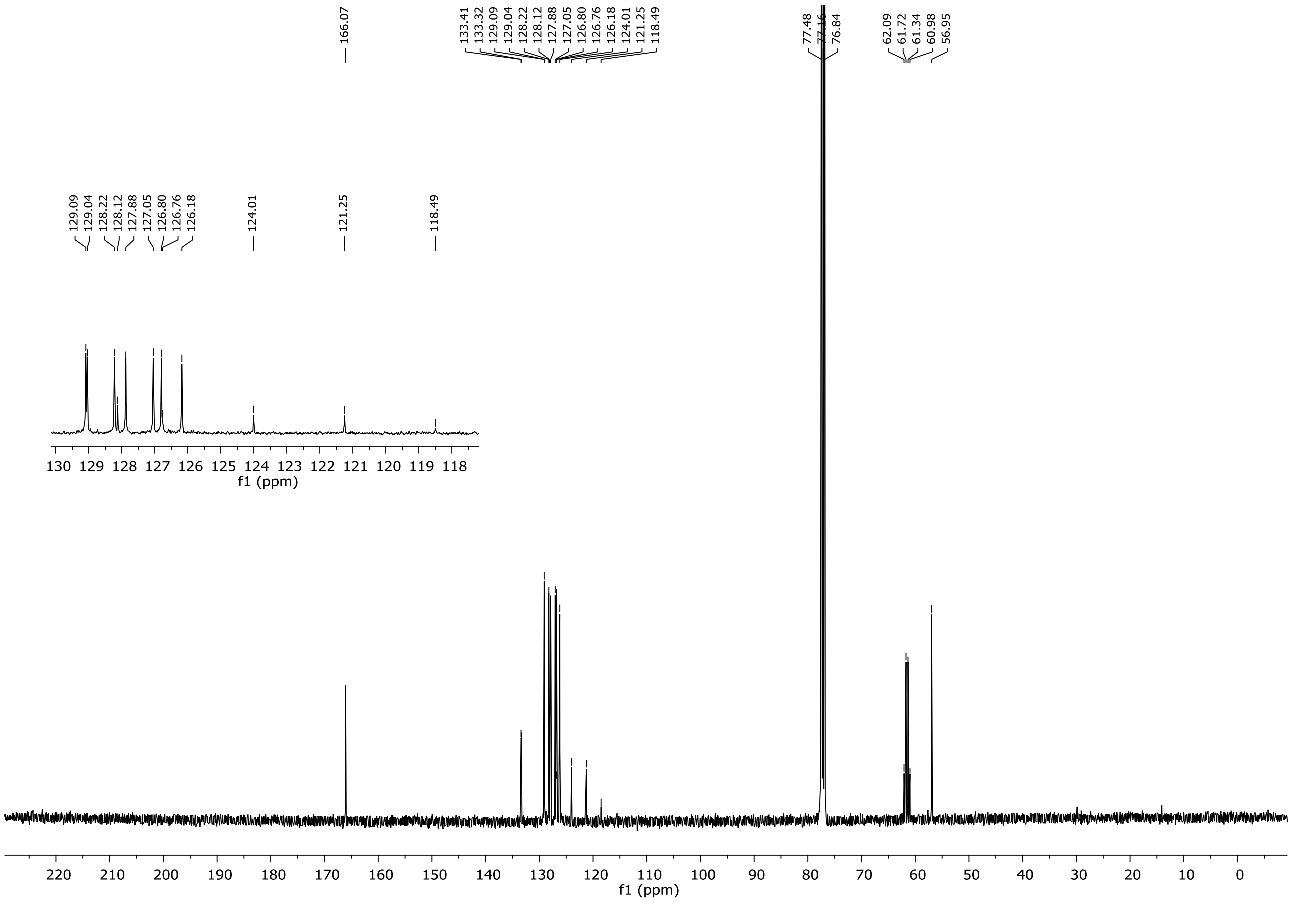

166.07
133.41 133.32 129.09 129.04 128.22 128.12 127.88 127.05 126.80 126.76 126.18 124.01 121.25 118.49
77.48 76.84
62.09 61.72 61.34 60.98 56.95
129.09 129.04 128.22 128.12 127.88 127.05 126.80 126.76 126.18
124.01
121.25
118.49
130 129 128 127 126 125 124 123 122 121 120 119 118
f1 (ppm)
220 210 200 190 180 170 160 150 140 130 120 110 100 90 80 70 60 50 40 30 20 10 0
f1 (ppm)

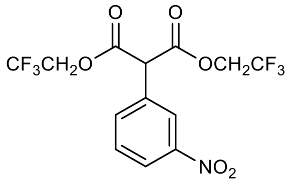
O
O
CF_3CH_2O
OCH_2CF_3
NO_2

S7

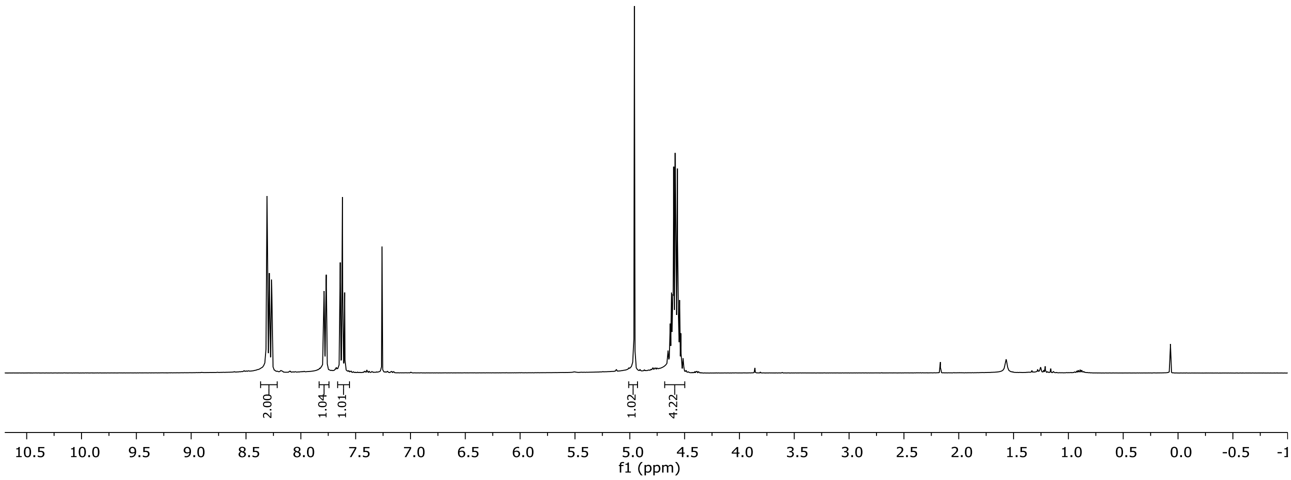
2.00
1.04
1.01
1.02
4.22
10.5 10.0 9.5 9.0 8.5 8.0 7.5 7.0 6.5 6.0 5.5 5.0 4.5 4.0 3.5 3.0 2.5 2.0 1.5 1.0 0.5 0.0 -0.5
f1 (ppm)

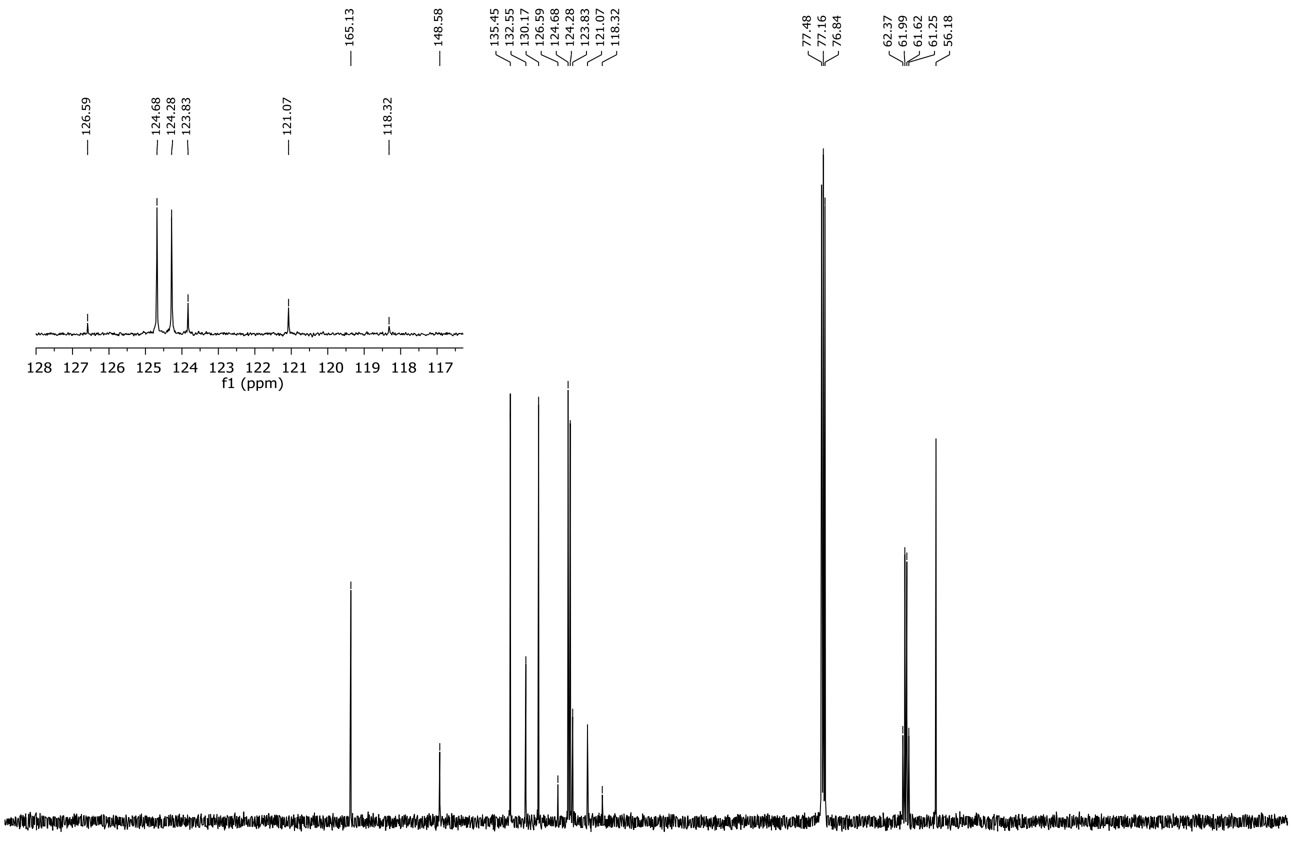
165.13
148.58
135.45
132.55
130.17
126.59
124.68
124.28
123.83
121.07
118.32
77.48
77.16
76.84
62.37
61.99
61.62
61.25
56.18
126.59
124.68
124.28
123.83
121.07
118.32
128 127 126 125 124 123 122 121 120 119 118 117
f1 (ppm)
220 210 200 190 180 170 160 150 140 130 120 110 100 90 80 70 60 50 40 30 20 10 0
f1 (ppm)

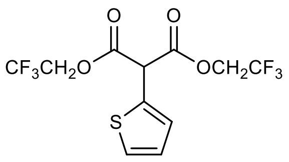
O
O
CF3CH2O
OCH2CF3
S

S8

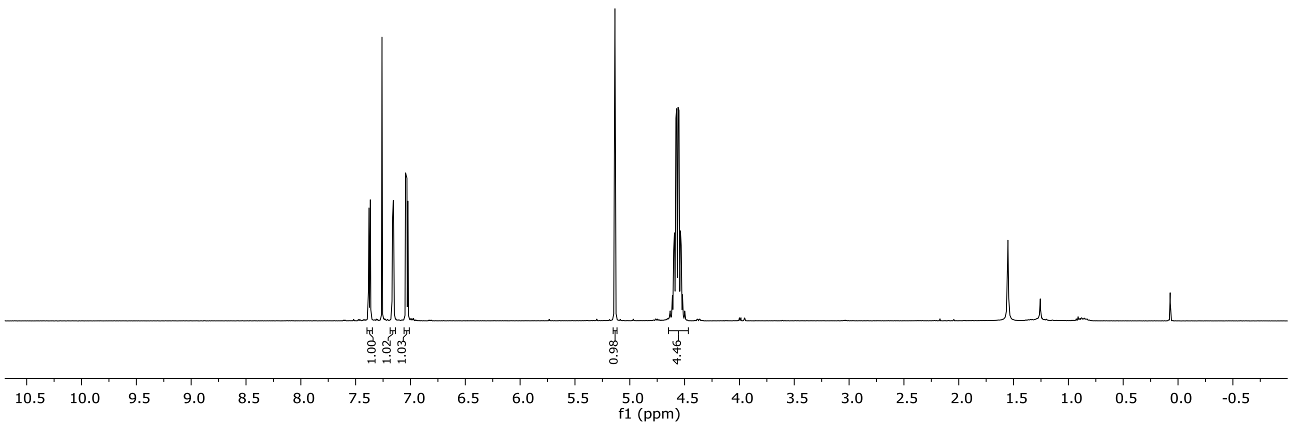
1.00
1.02
1.03
0.98
4.46
f1 (ppm)

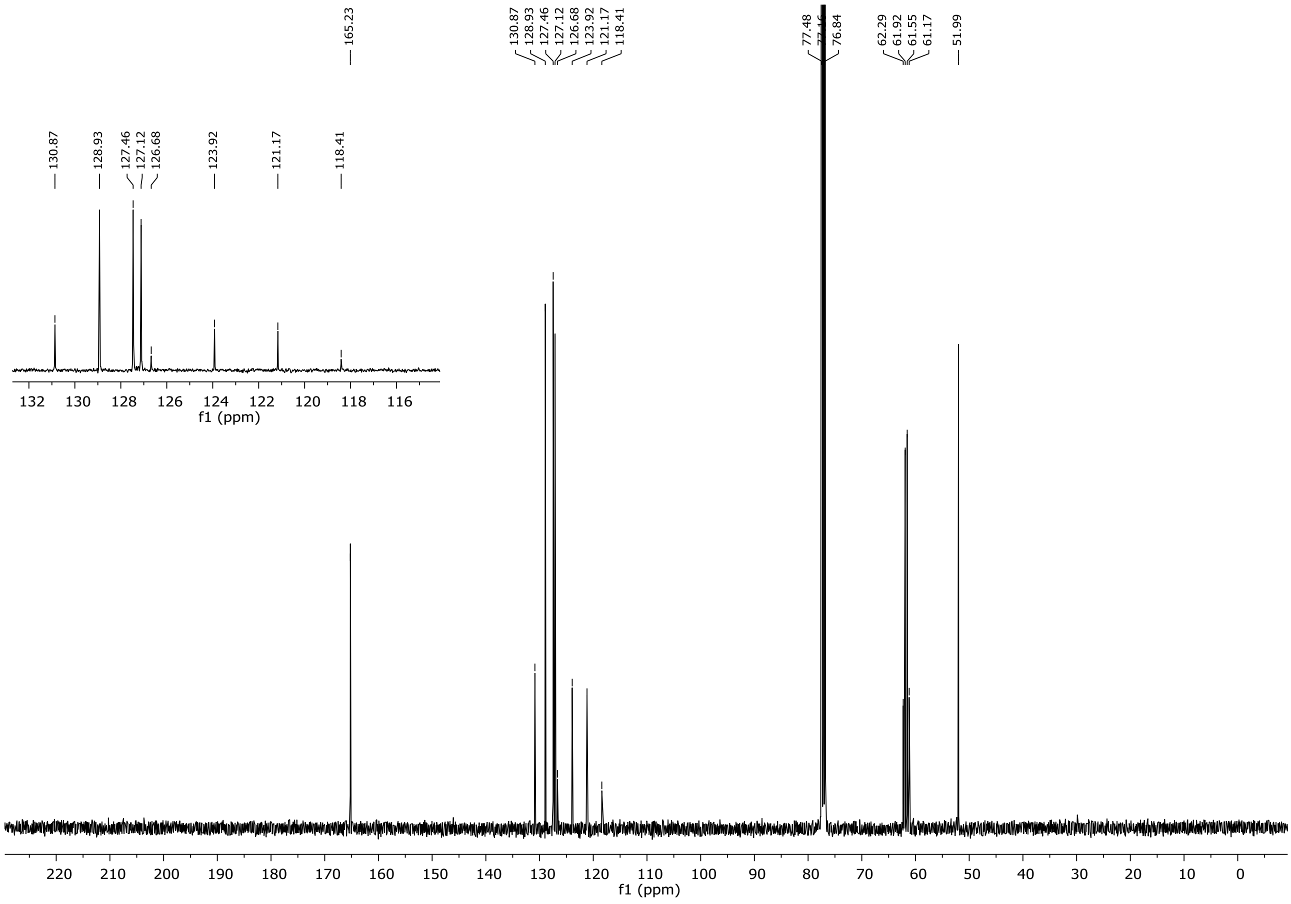
165.23
130.87
128.93
127.46
127.12
126.68
123.92
121.17
118.41
77.48
76.84
62.29
61.92
61.55
61.17
51.99
f1 (ppm)

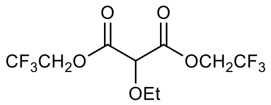
O
O
CF3CH2O
OCH2CF3
OEt

S10a

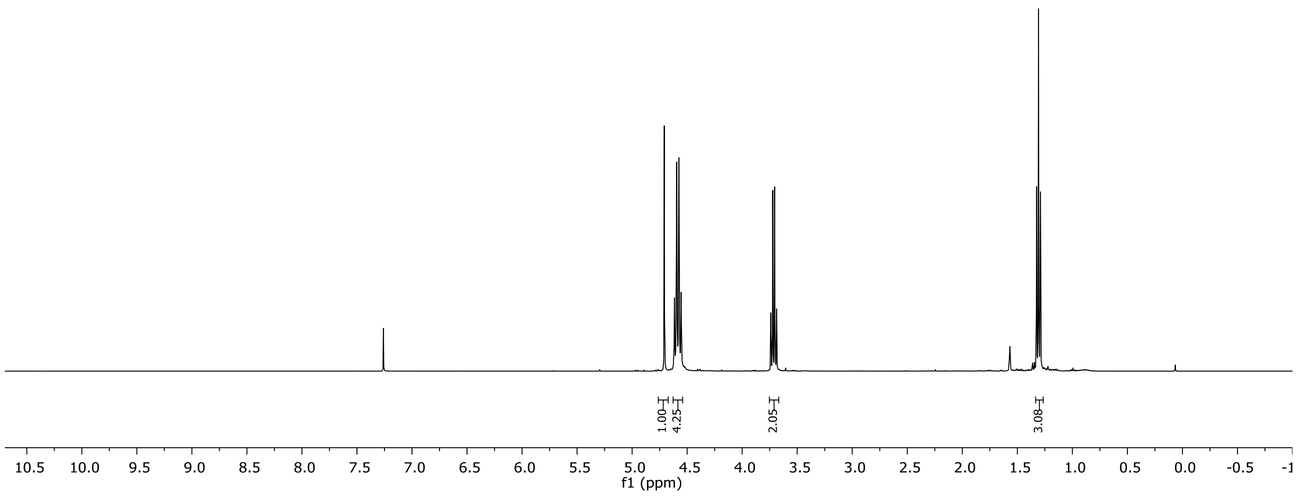
1.00
4.25
2.05
3.08
10.5 10.0 9.5 9.0 8.5 8.0 7.5 7.0 6.5 6.0 5.5 5.0 4.5 4.0 3.5 3.0 2.5 2.0 1.5 1.0 0.5 0.0 -0.5
f1 (ppm)

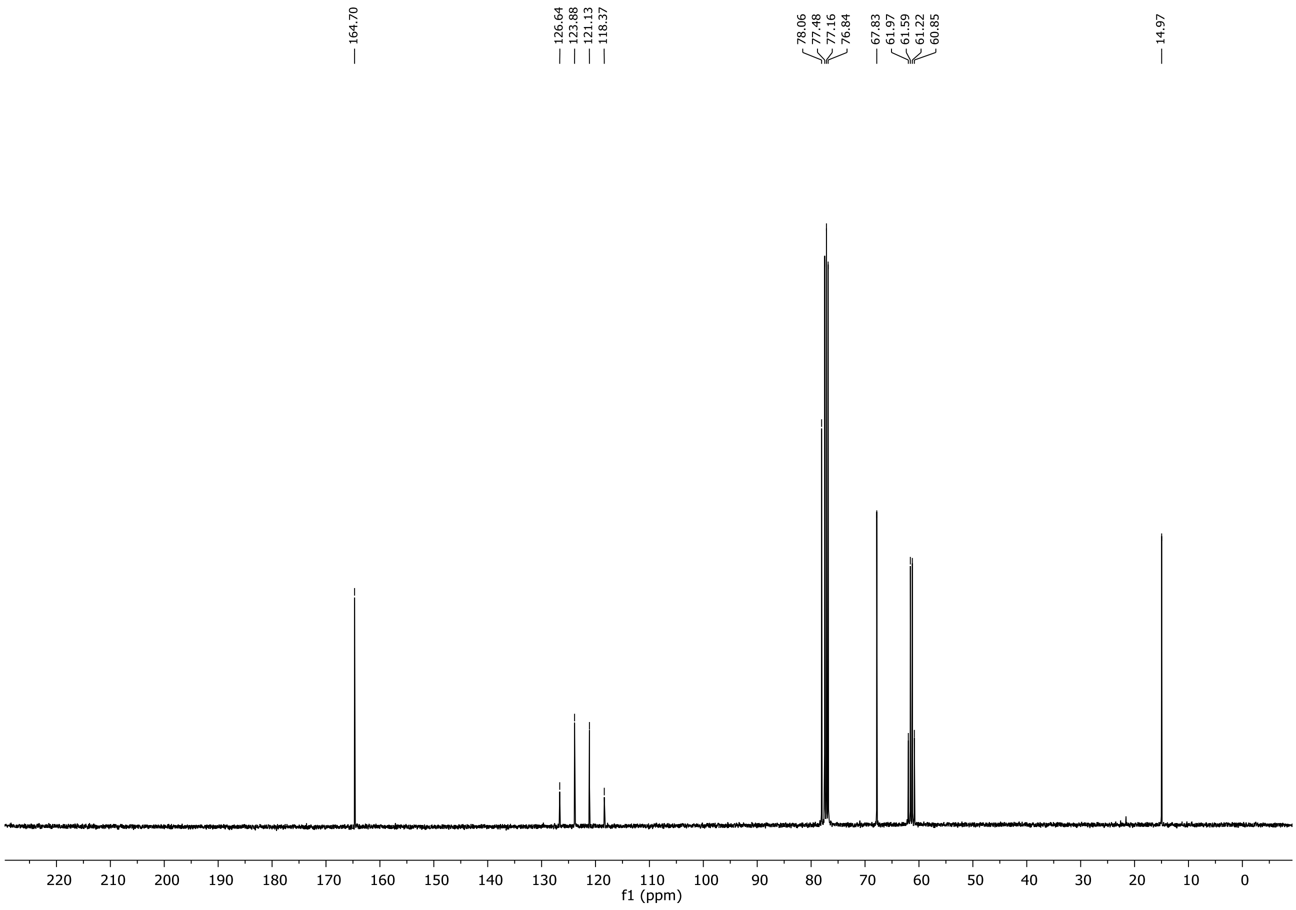
164.70
126.64
123.88
121.13
118.37
78.06
77.48
77.16
76.84
67.83
61.97
61.59
61.22
60.85
14.97
220 210 200 190 180 170 160 150 140 130 120 110 100 90 80 70 60 50 40 30 20 10 0
f1 (ppm)

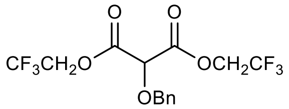
CF3CH2O
OCH2CF3
OBn

S10b

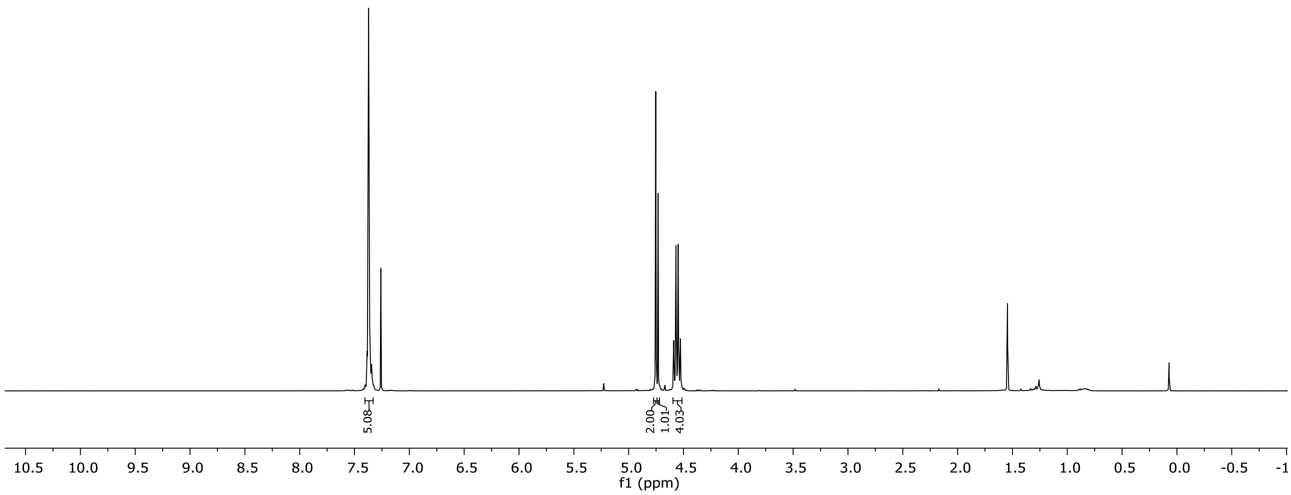
5.08
2.00
1.01
4.03
f1 (ppm)

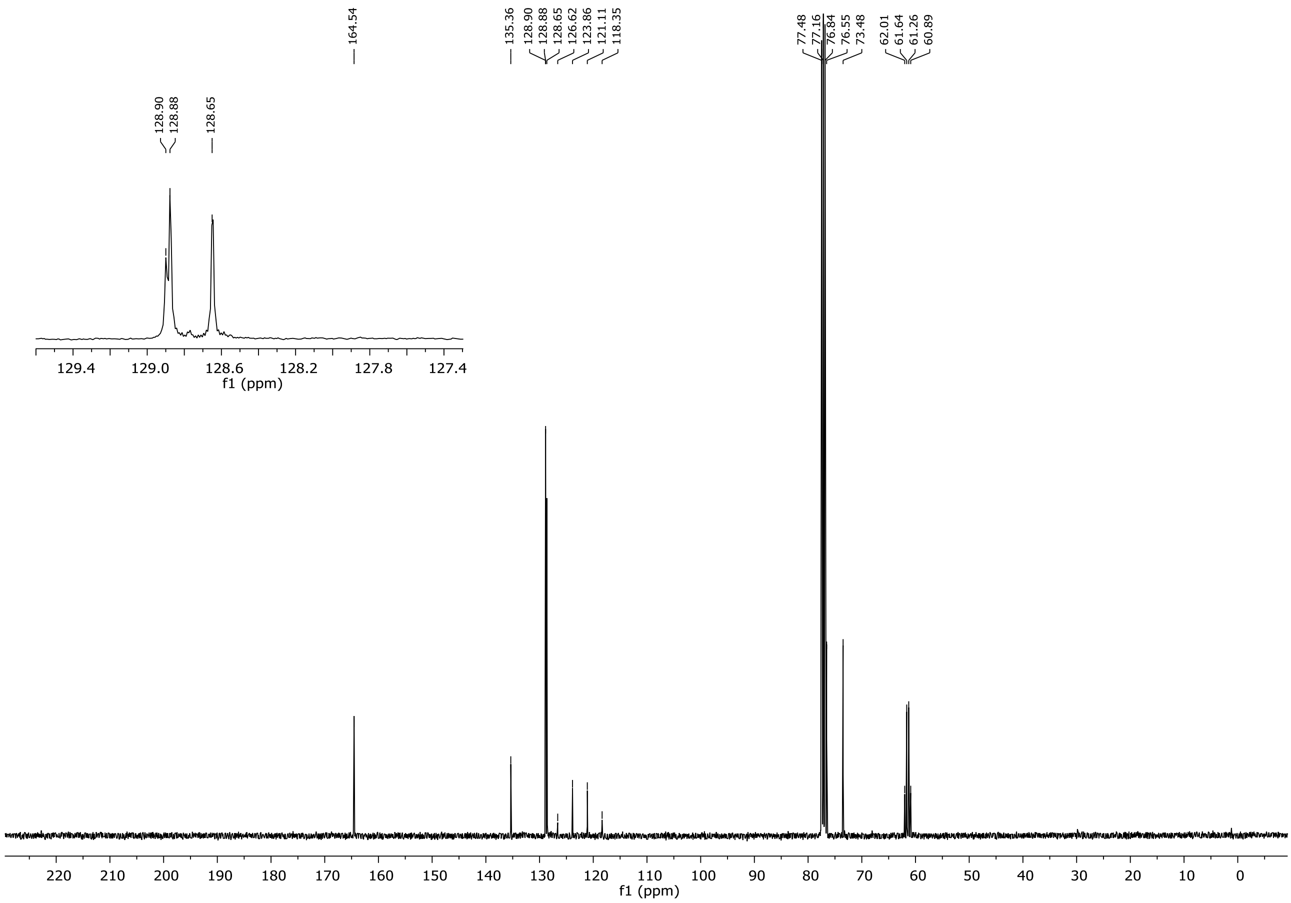
164.54
135.36
128.90
128.88
128.65
126.62
123.86
121.11
118.35
77.48
77.16
76.84
76.55
73.48
62.01
61.64
61.26
60.89
f1 (ppm)

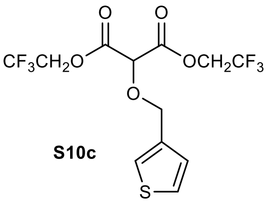

S10c

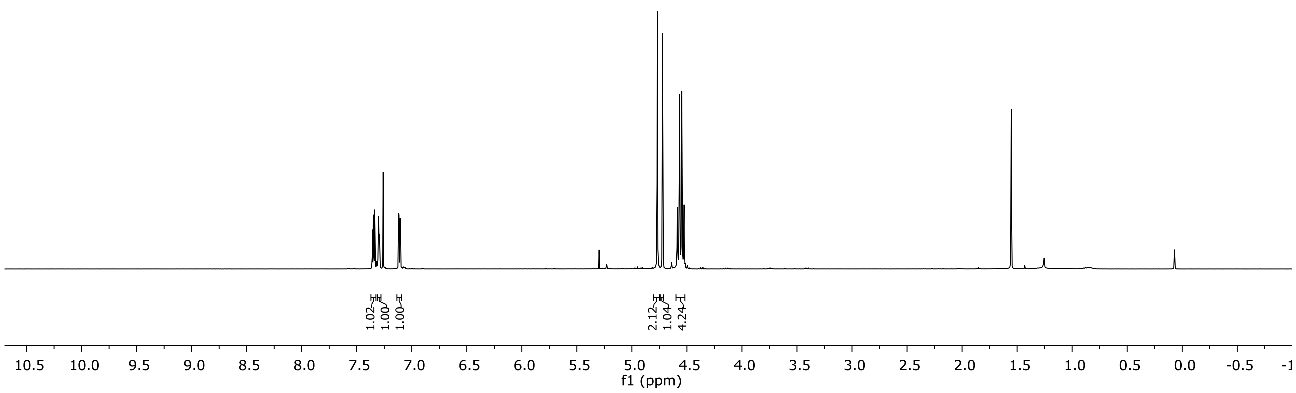

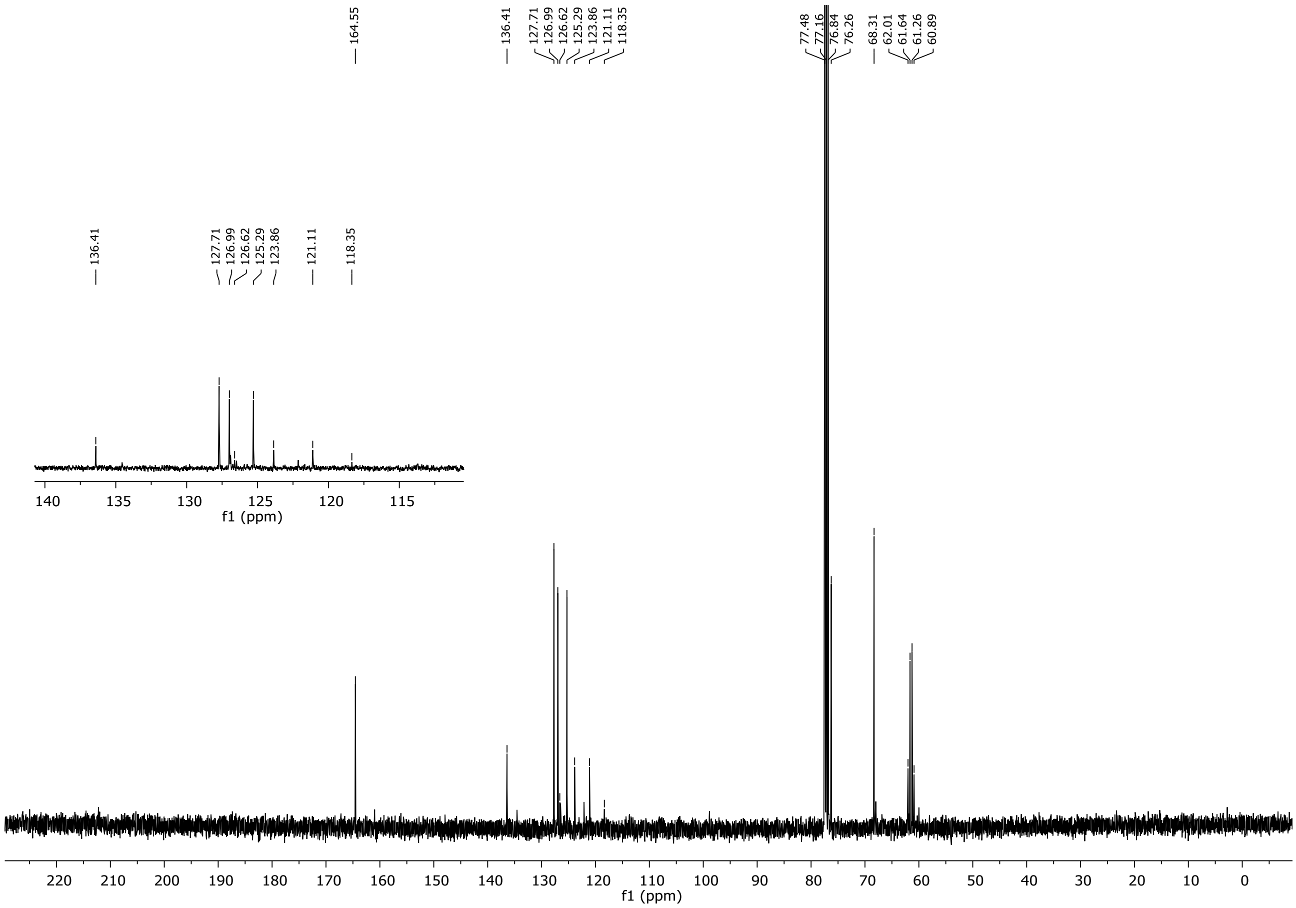

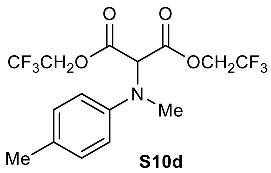
CF3CH2O
OCH2CF3
N
Me
Me

S10d

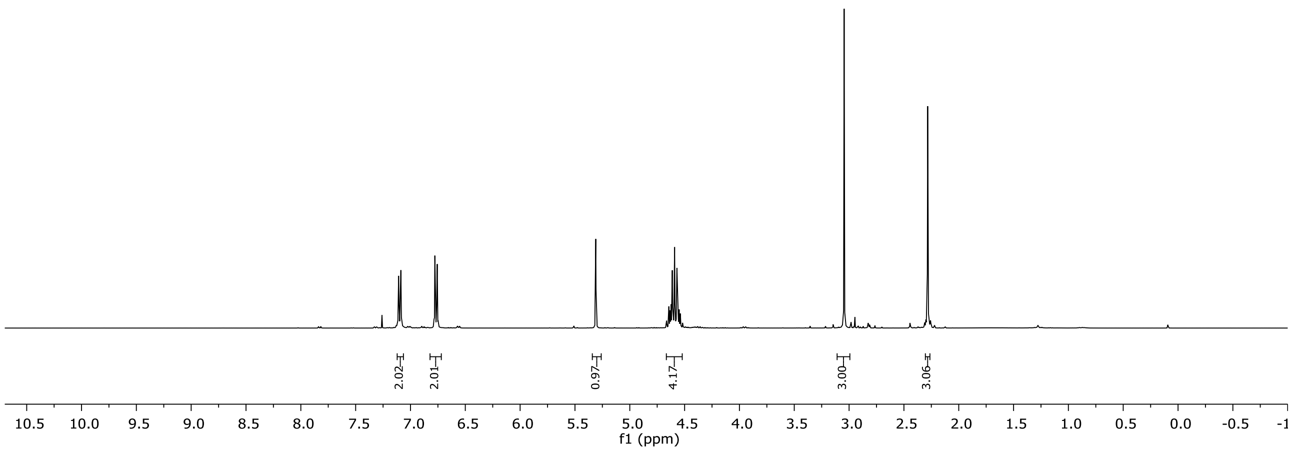
2.02
2.01
0.97
4.17
3.00
3.06
10.5 10.0 9.5 9.0 8.5 8.0 7.5 7.0 6.5 6.0 5.5 5.0 4.5 4.0 3.5 3.0 2.5 2.0 1.5 1.0 0.5 0.0 -0.5 -1
f1 (ppm)

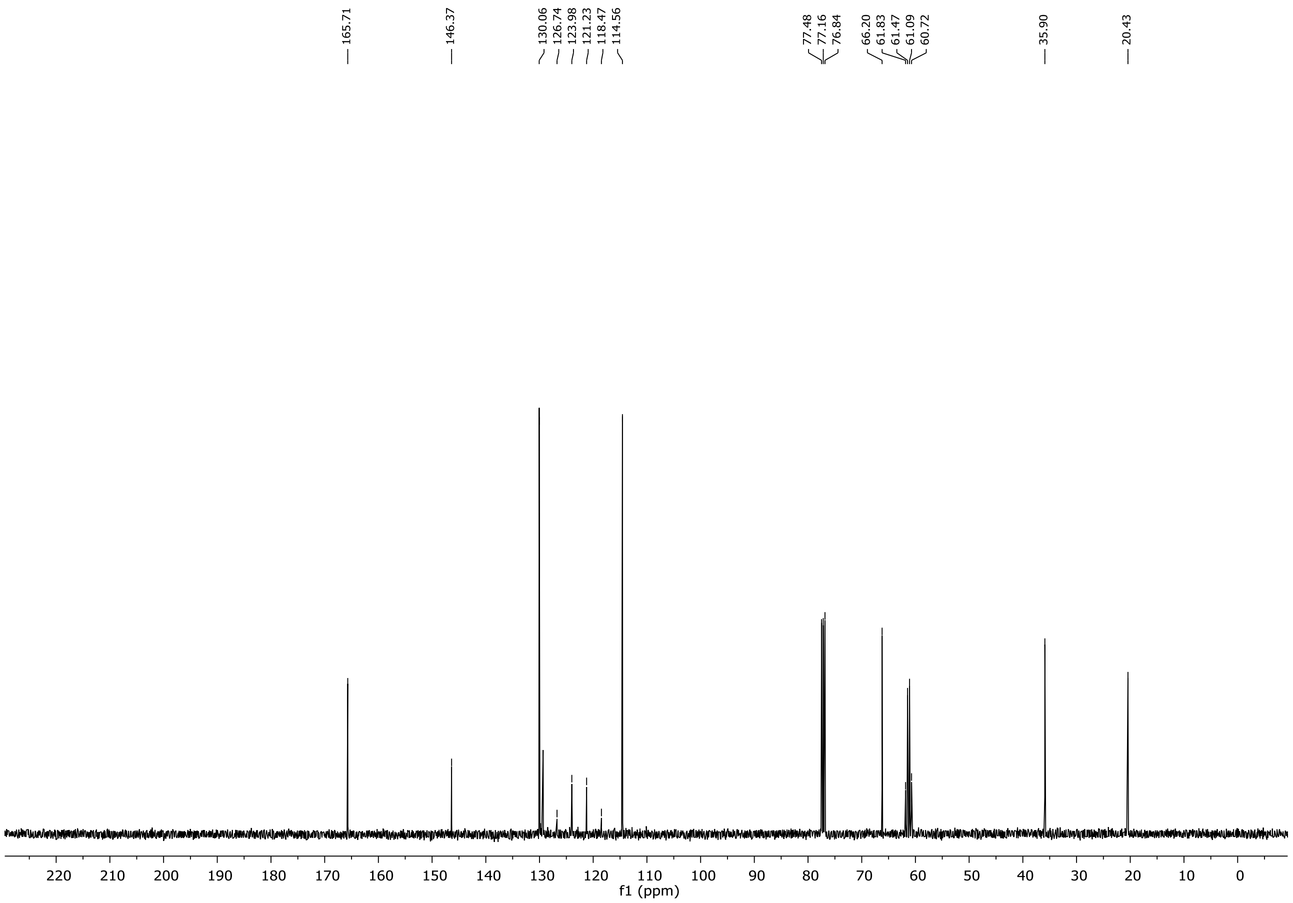
165.71
146.37
130.06
126.74
123.98
121.23
118.47
114.56
77.48
77.16
76.84
66.20
61.83
61.47
61.09
60.72
35.90
20.43
220 210 200 190 180 170 160 150 140 130 120 110 100 90 80 70 60 50 40 30 20 10 0
f1 (ppm)

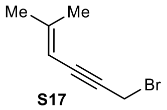
Me
Me
Br

S17

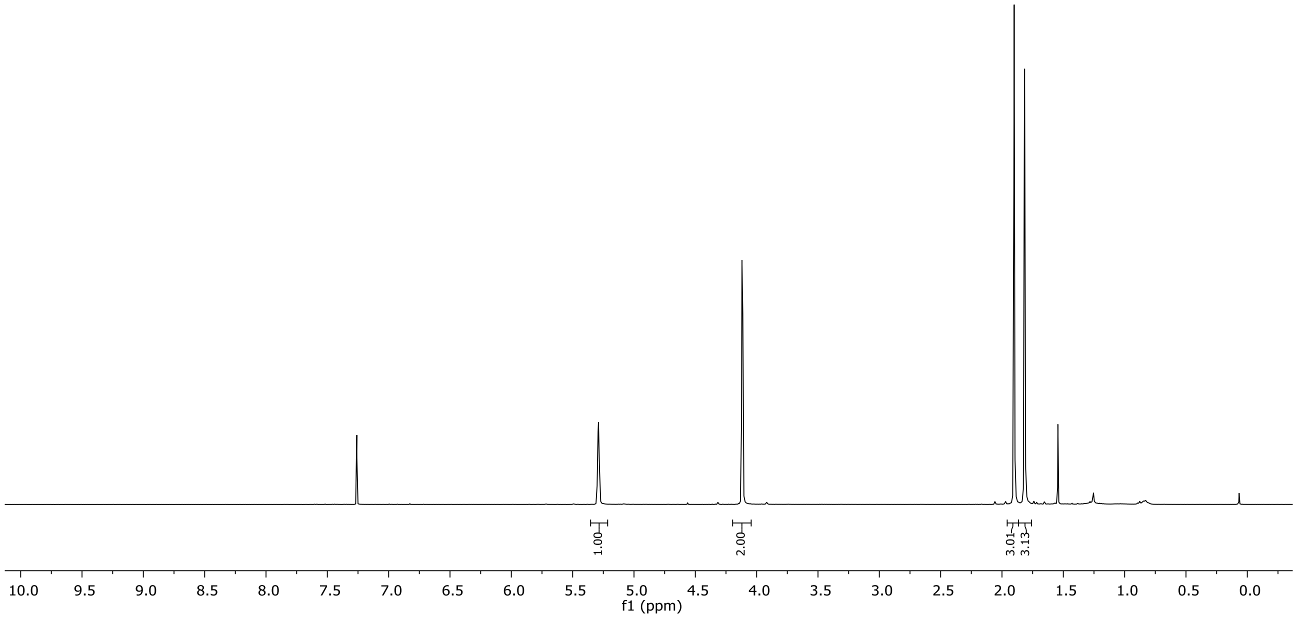
1.00
2.00
3.01
3.13
10.0 9.5 9.0 8.5 8.0 7.5 7.0 6.5 6.0 5.5 5.0 4.5 4.0 3.5 3.0 2.5 2.0 1.5 1.0 0.5 0.0
f1 (ppm)

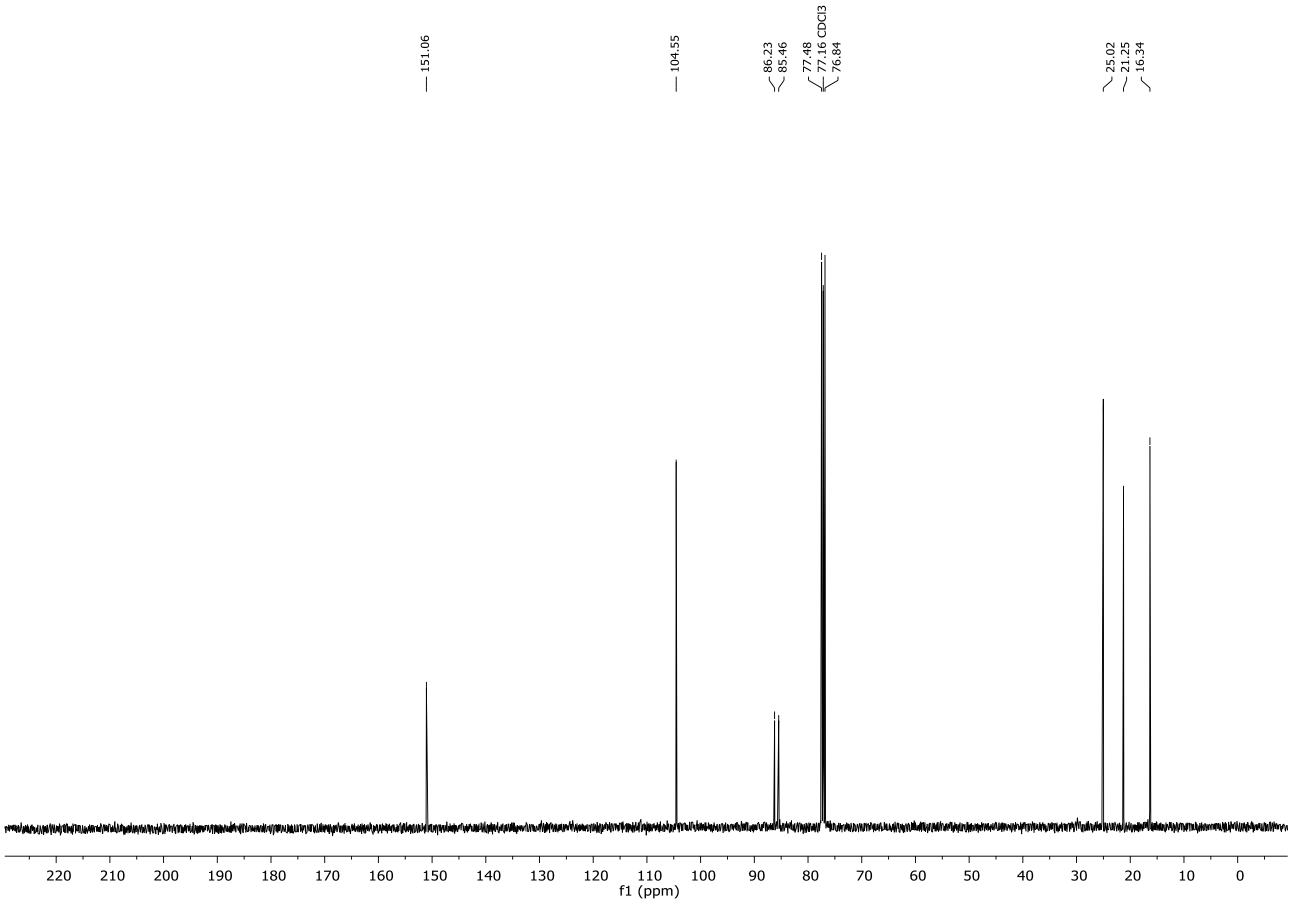
151.06
104.55
86.23
85.46
77.48
77.16 CDCl3
76.84
25.02
21.25
16.34
220 210 200 190 180 170 160 150 140 130 120 110 100 90 80 70 60 50 40 30 20 10 0
f1 (ppm)

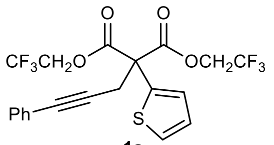
CF3CH2O
OCH2CF3
Ph
S

1a

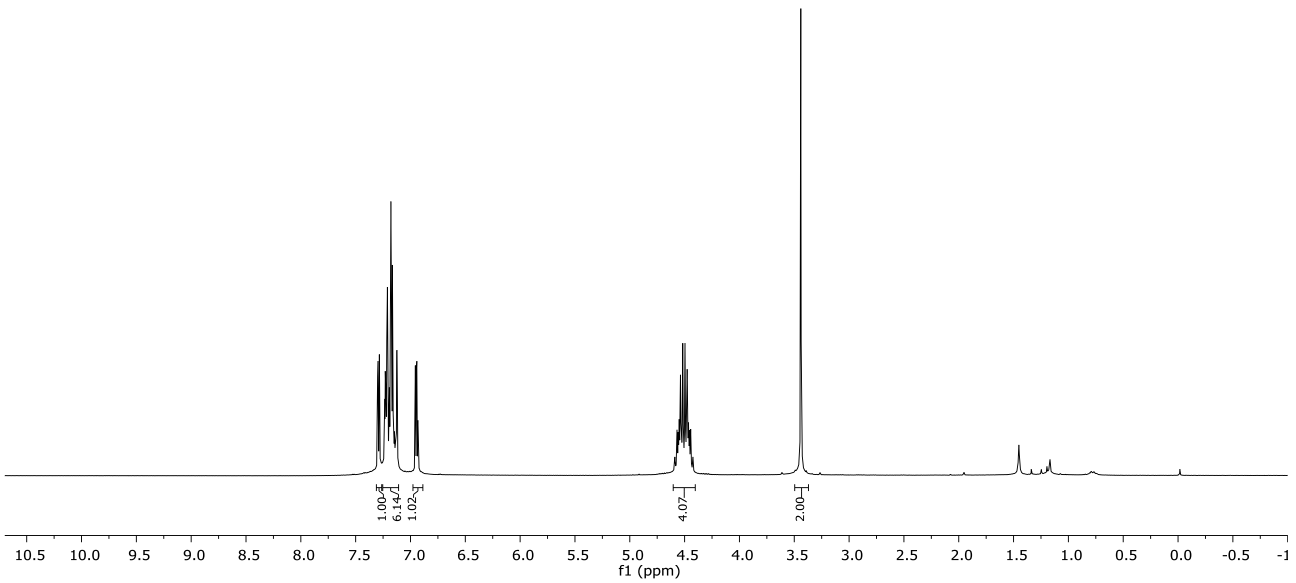
1.00
6.14
1.02
4.07
2.00
f1 (ppm)

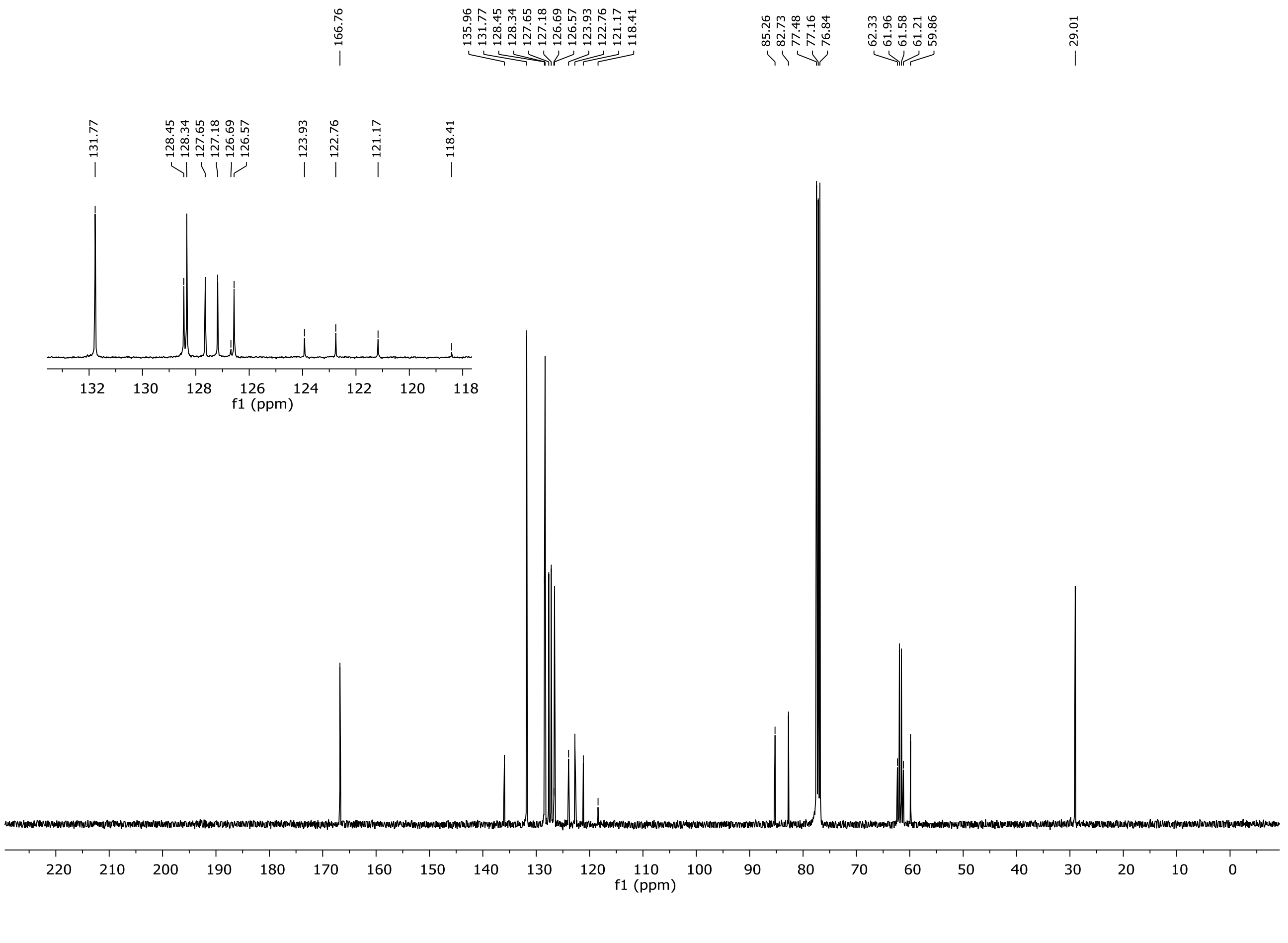
166.76
135.96
131.77
128.45
128.34
127.65
127.18
126.69
126.57
123.93
122.76
121.17
118.41
85.26
82.73
77.48
77.16
76.84
62.33
61.96
61.58
61.21
59.86
29.01
f1 (ppm)

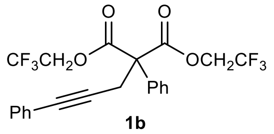

1b

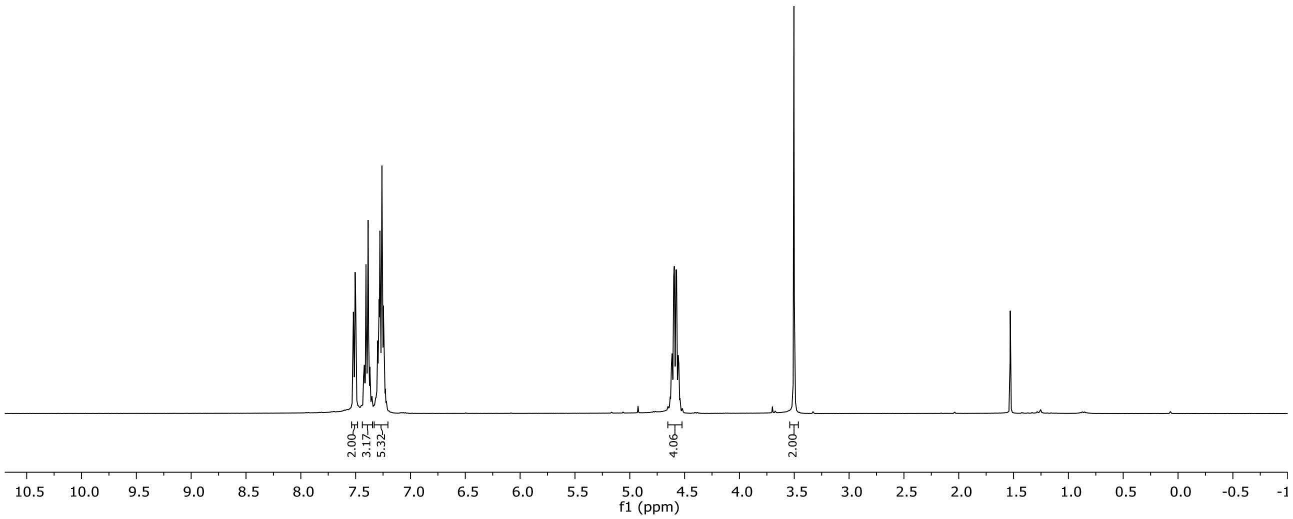

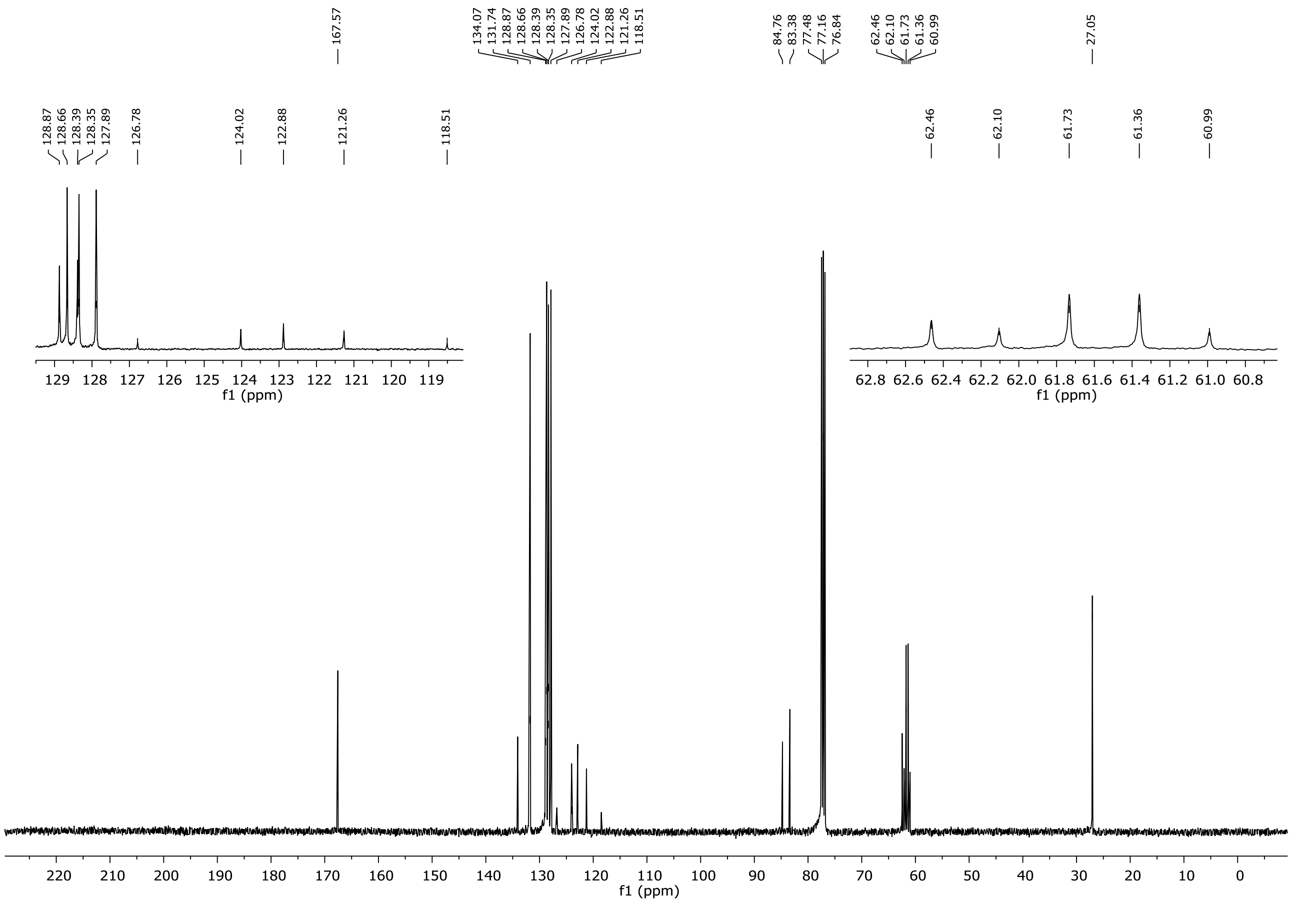

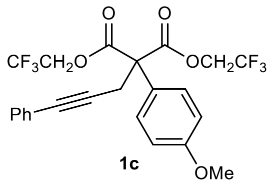
CF3CH2O
OCH2CF3
Ph
OMe

1c

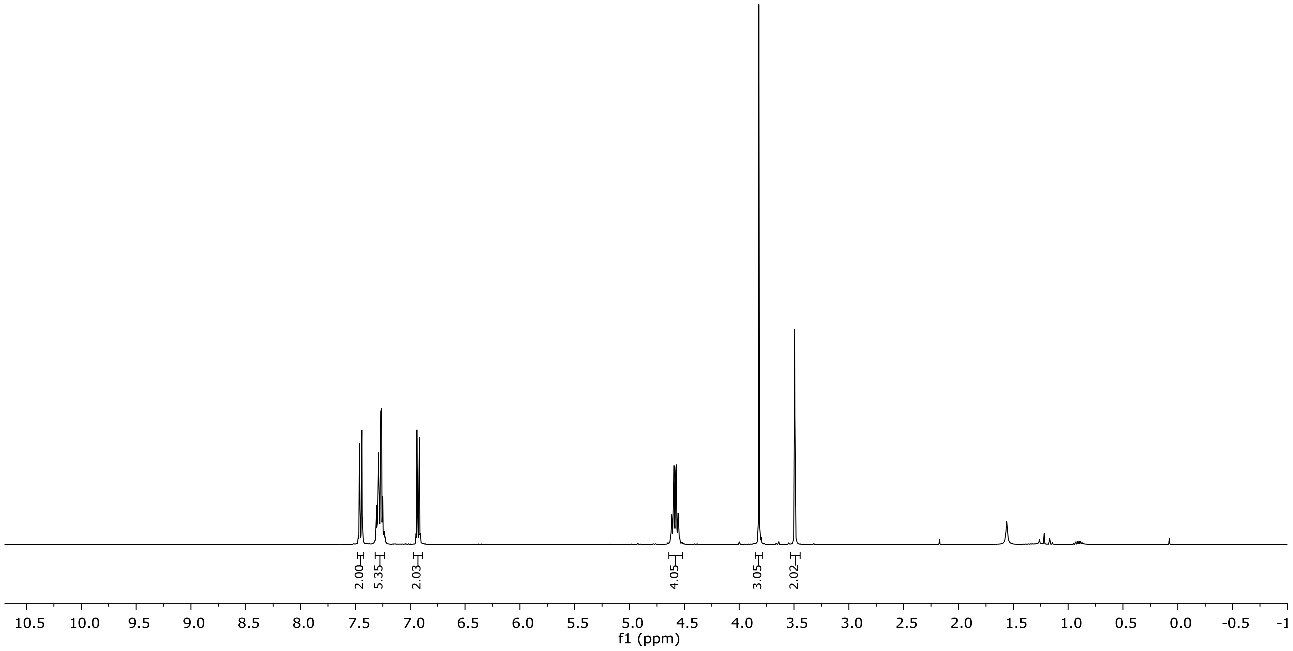
2.00
5.35
2.03
4.05
3.05
2.02
f1 (ppm)

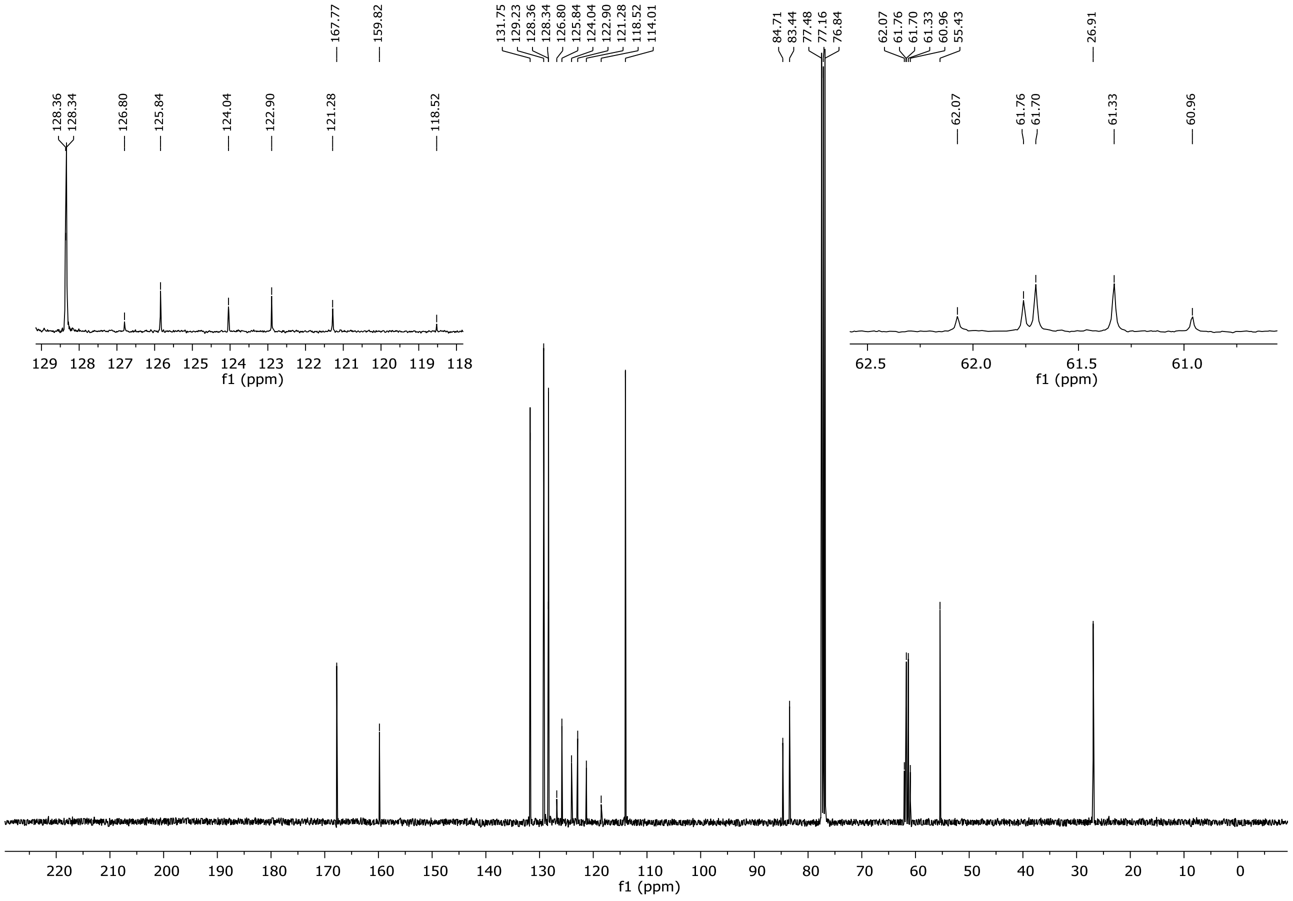
167.77
159.82
131.75
129.23
128.36
128.34
126.80
125.84
124.04
122.90
121.28
118.52
114.01
84.71
83.44
77.48
77.16
76.84
62.07
61.76
61.70
61.33
60.96
55.43
26.91
f1 (ppm)

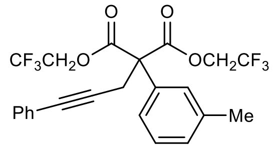
CF3CH2O
OCH2CF3
Ph
Me

1d

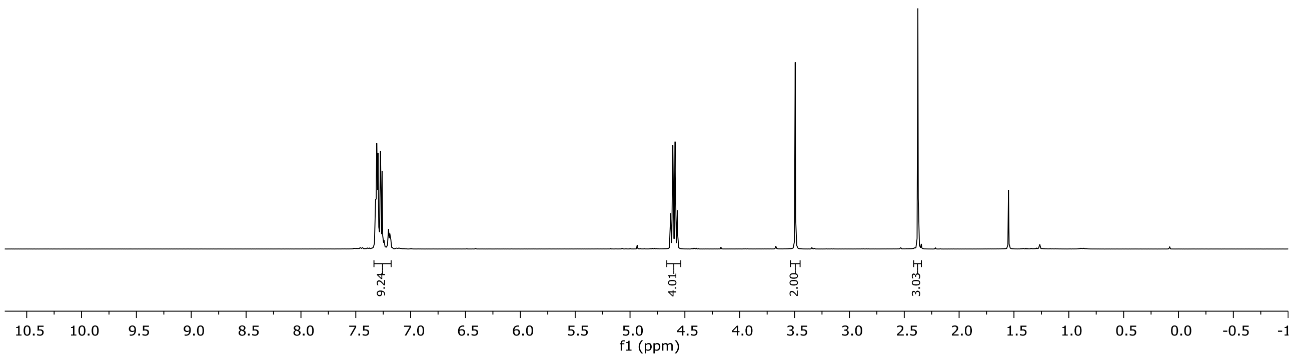
9.24
4.01
2.00
3.03
f1 (ppm)

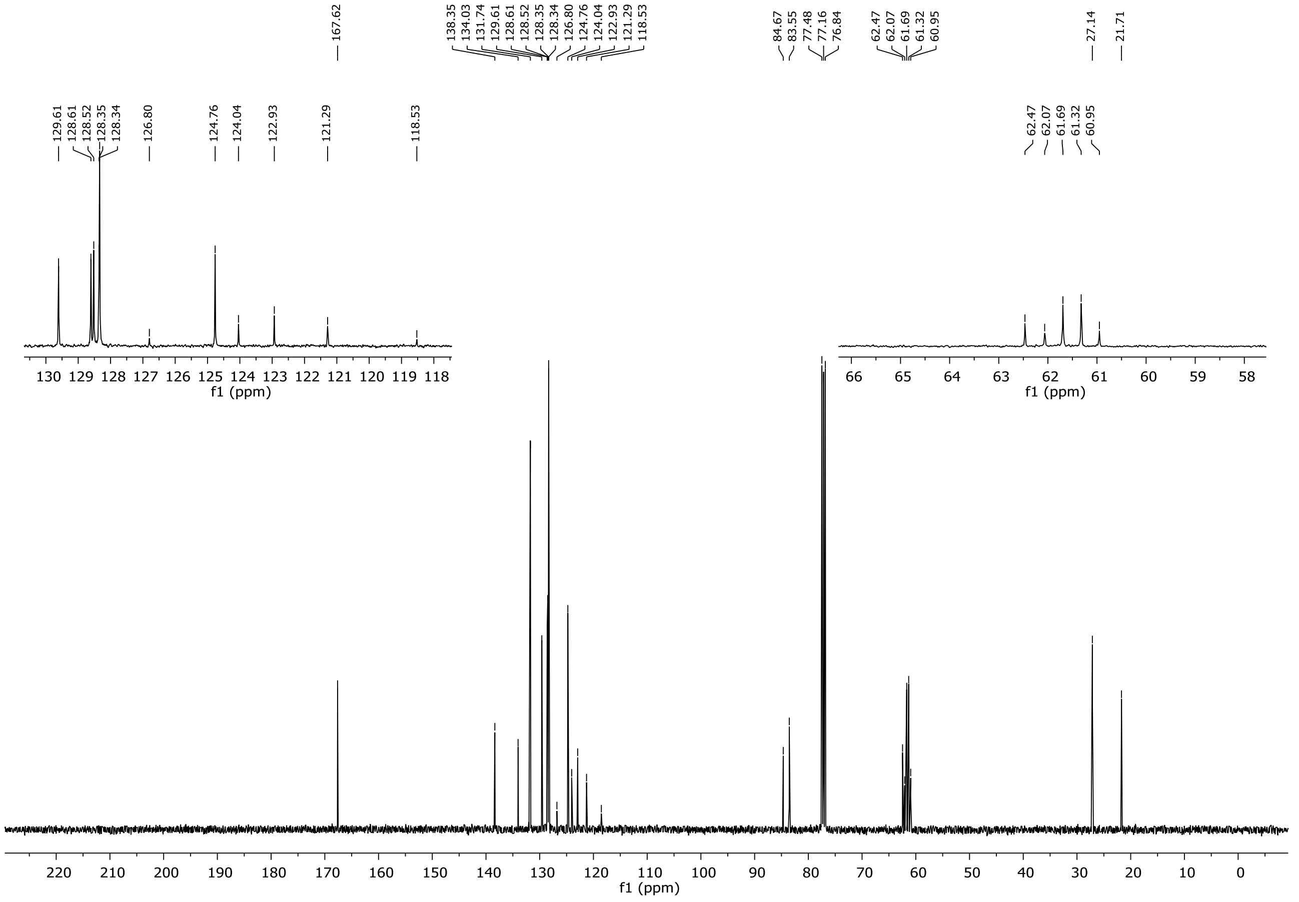
167.62
138.35
134.03
131.74
129.61
128.61
128.52
128.35
128.34
126.80
124.76
124.04
122.93
121.29
118.53
84.67
83.55
77.48
77.16
76.84
62.47
62.07
61.69
61.32
60.95
27.14
21.71
f1 (ppm)

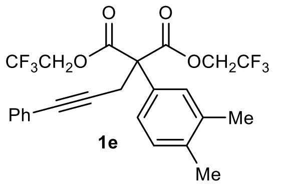
CF3CH2O
OCH2CF3
Ph
Me
Me

1e

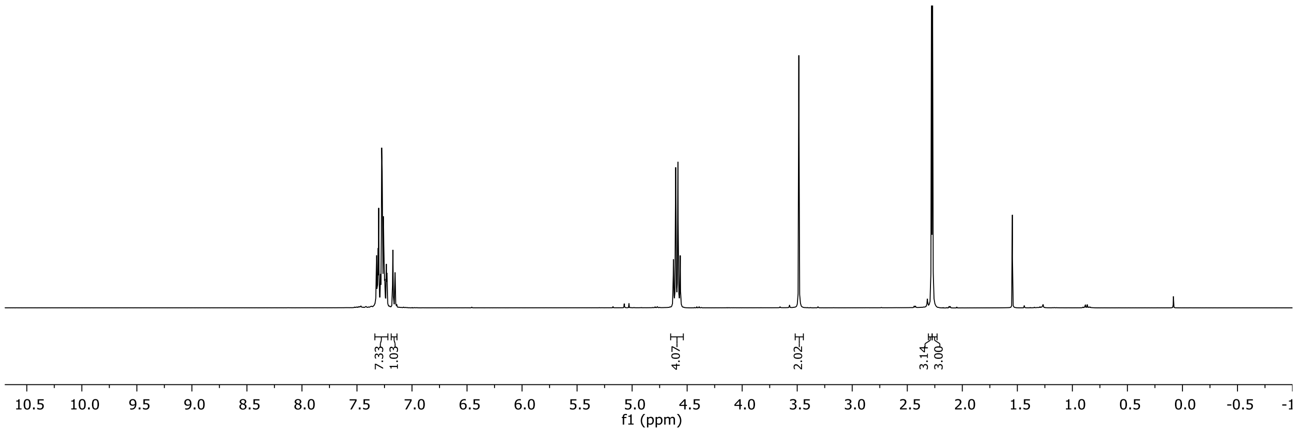
7.33
1.03
4.07
2.02
3.14
3.00
10.5 10.0 9.5 9.0 8.5 8.0 7.5 7.0 6.5 6.0 5.5 5.0 4.5 4.0 3.5 3.0 2.5 2.0 1.5 1.0 0.5 0.0 -0.5 -1
f1 (ppm)

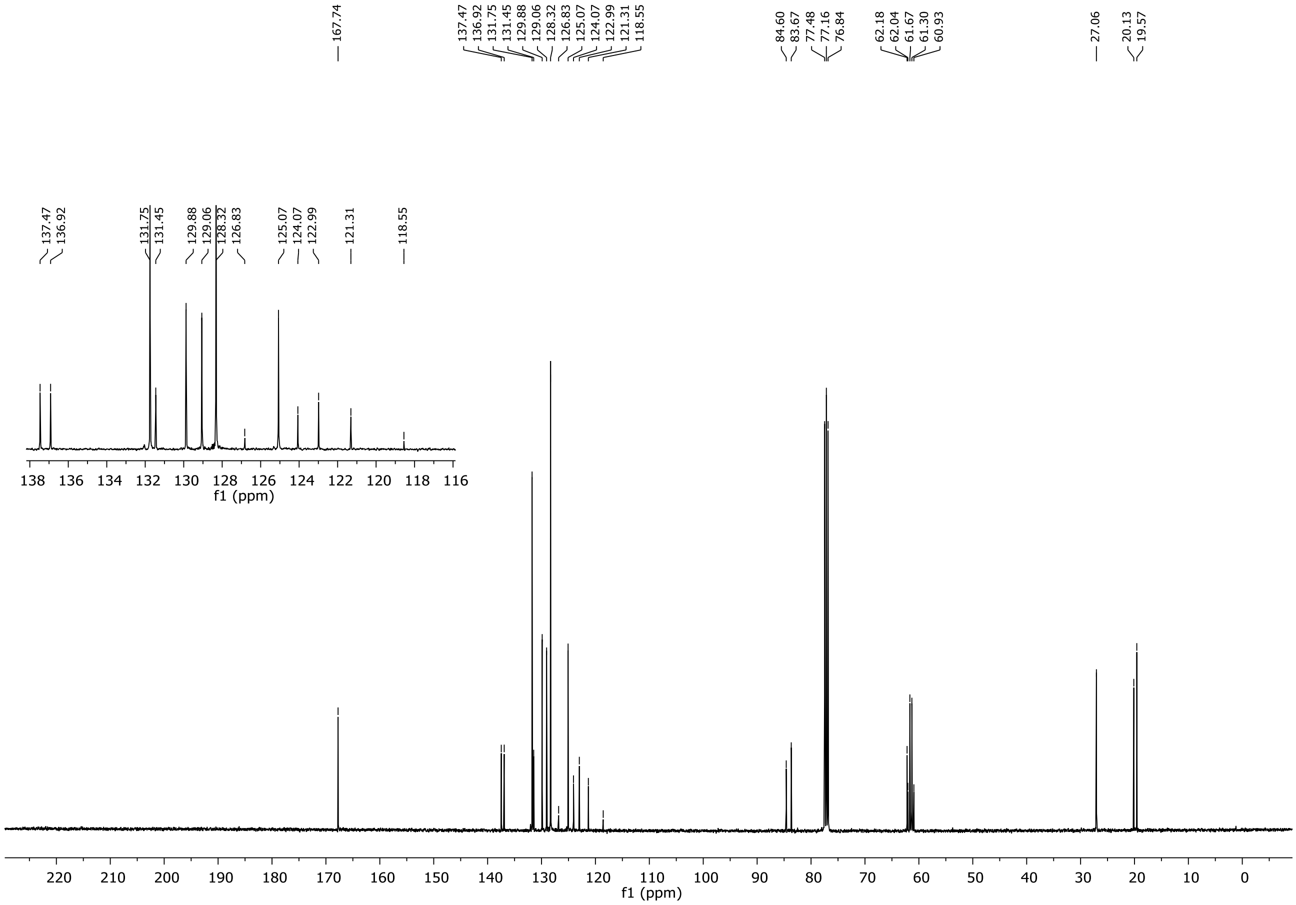
167.74
137.47 136.92 131.75 131.45 129.88 129.06 128.32 126.83 125.07 124.07 122.99 121.31 118.55
84.60 83.67 77.48 77.16 76.84
62.18 62.04 61.67 61.30 60.93
27.06
20.13 19.57
138 136 134 132 130 128 126 124 122 120 118 116
f1 (ppm)
220 210 200 190 180 170 160 150 140 130 120 110 100 90 80 70 60 50 40 30 20 10 0
f1 (ppm)

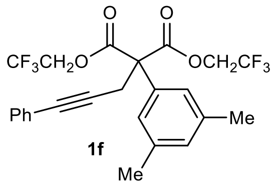
O
O
CF3CH2O
OCH2CF3
Ph
Me
Me

1f

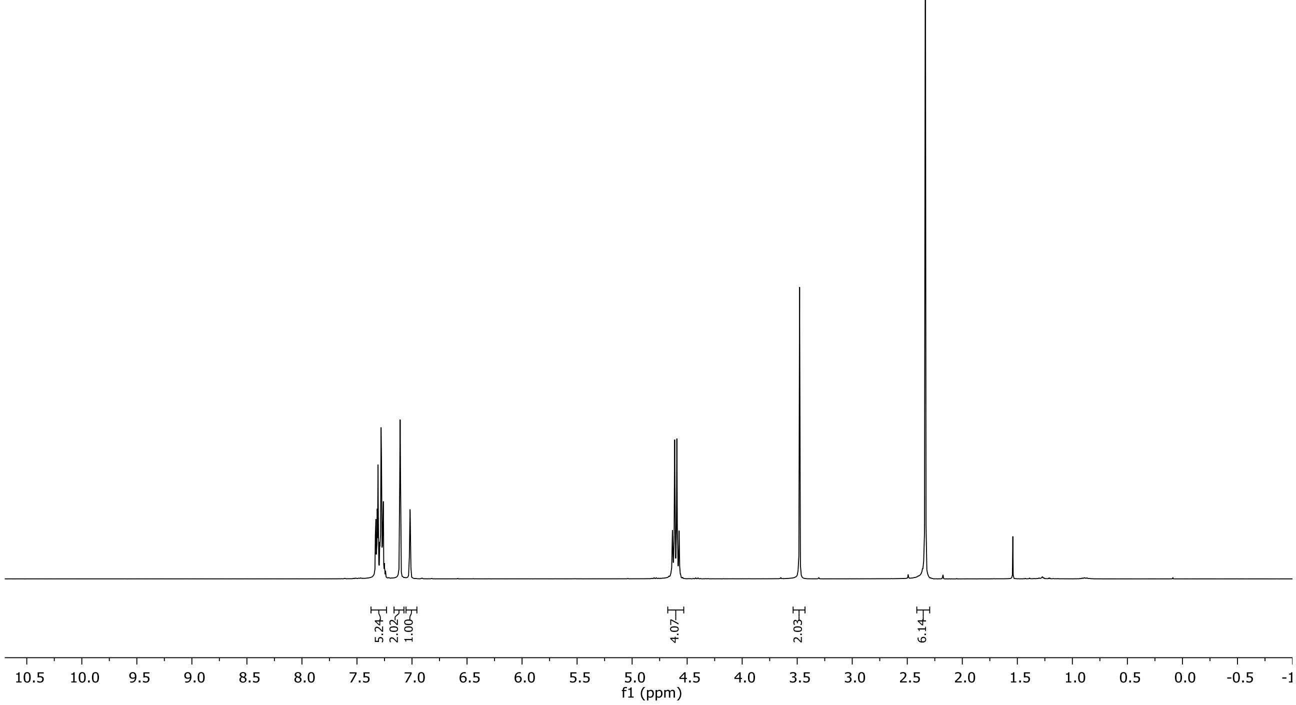
5.24
2.02
1.00
4.07
2.03
6.14
10.5 10.0 9.5 9.0 8.5 8.0 7.5 7.0 6.5 6.0 5.5 5.0 4.5 4.0 3.5 3.0 2.5 2.0 1.5 1.0 0.5 0.0 -0.5 -1
f1 (ppm)

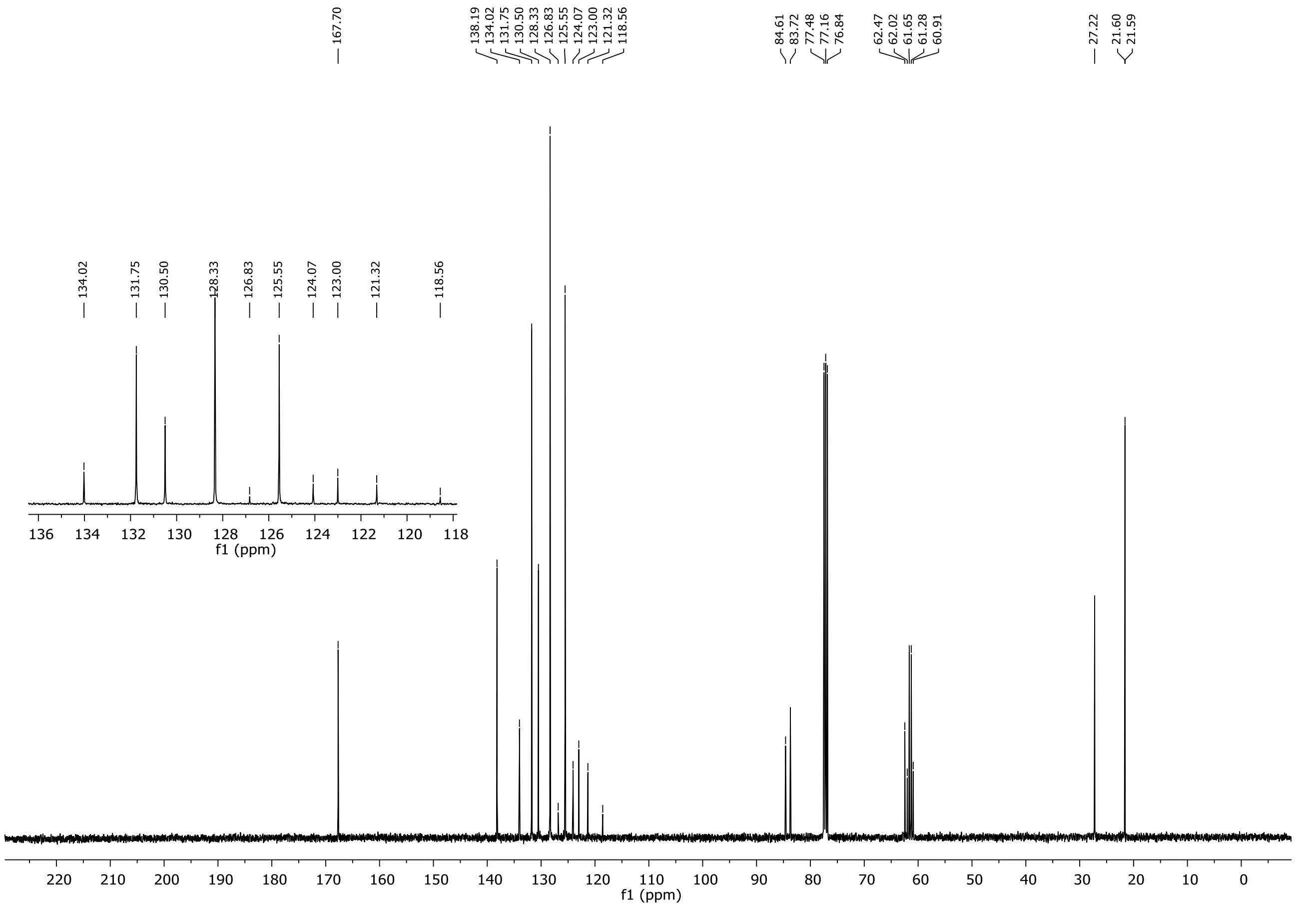
167.70
138.19
134.02
131.75
130.50
128.33
126.83
125.55
124.07
123.00
121.32
118.56
84.61
83.72
77.48
77.16
76.84
62.47
62.02
61.65
61.28
60.91
27.22
21.60
21.59
136 134 132 130 128 126 124 122 120 118
f1 (ppm)
220 210 200 190 180 170 160 150 140 130 120 110 100 90 80 70 60 50 40 30 20 10 0
f1 (ppm)

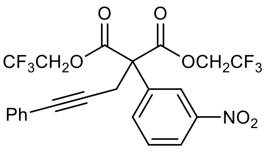

O
O
CF3CH2O
OCH2CF3
Ph
NO2

1g

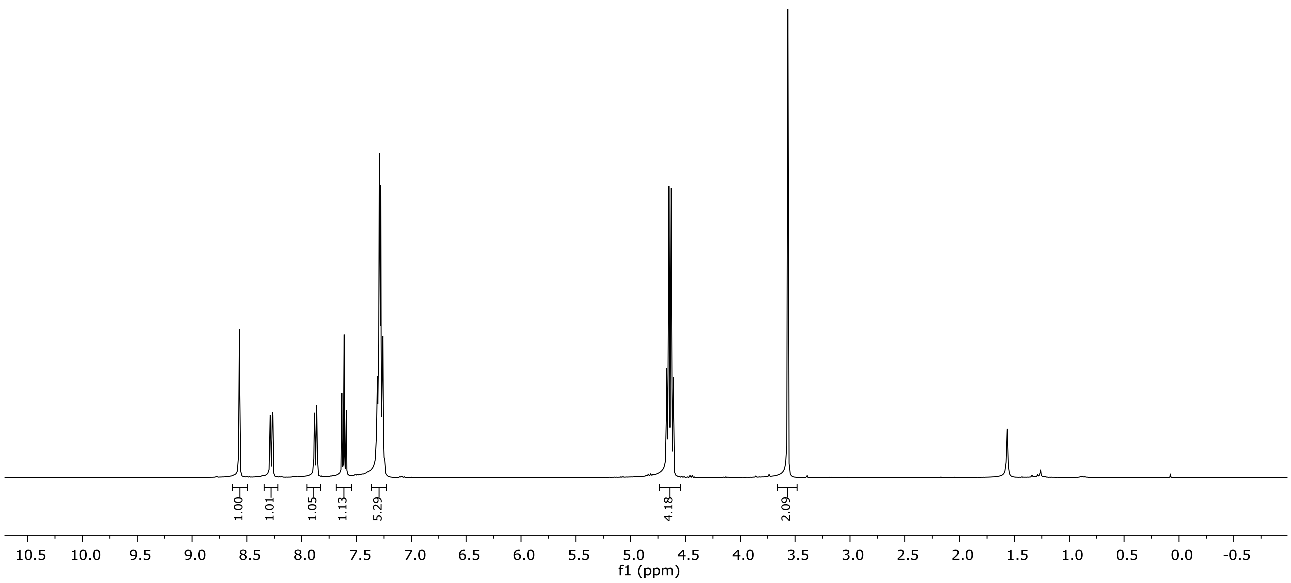

1.00
1.01
1.05
1.13
5.29
4.18
2.09
f1 (ppm)

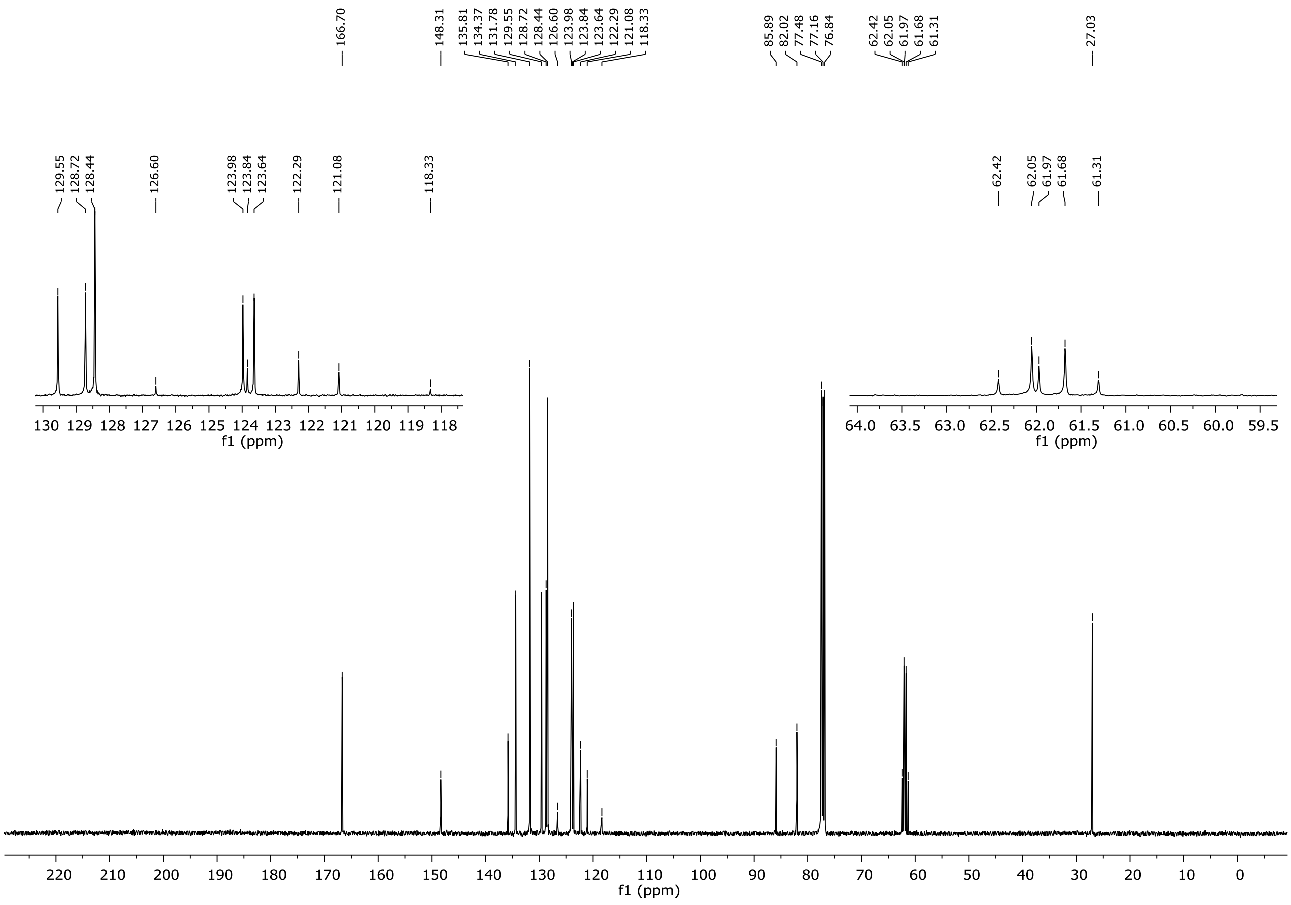

166.70
148.31
135.81
134.37
131.78
129.55
128.72
128.44
126.60
123.98
123.84
123.64
122.29
121.08
118.33
85.89
82.02
77.48
77.16
76.84
62.42
62.05
61.97
61.68
61.31
27.03
f1 (ppm)

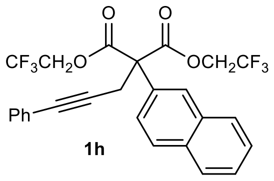
CF3CH2O
OCH2CF3
Ph

1h

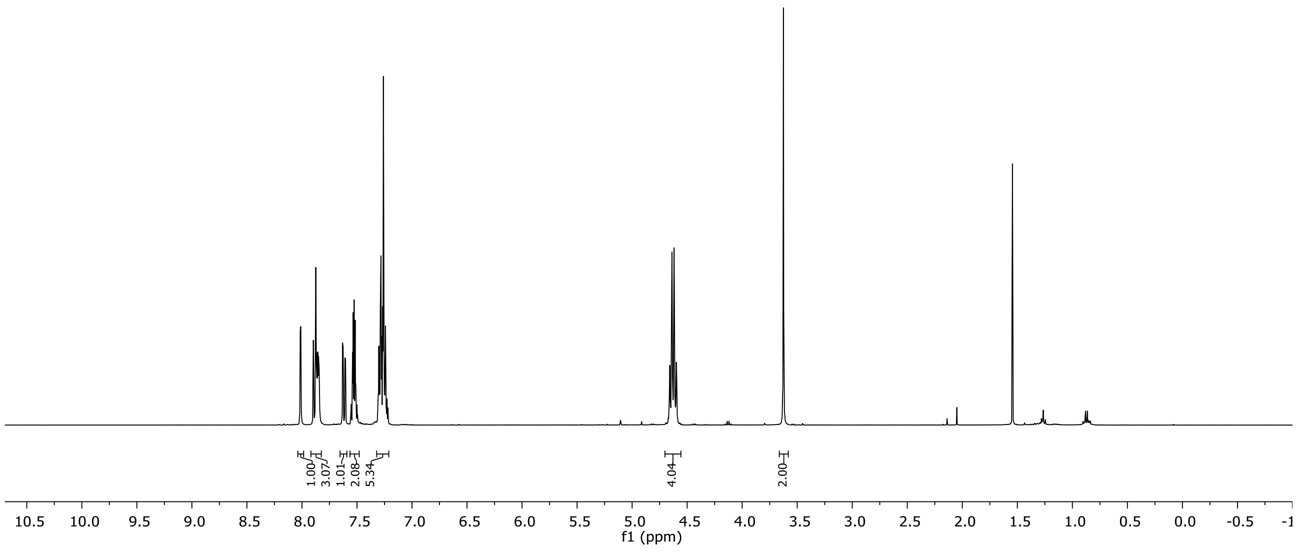
1.00
3.07
1.01
2.08
5.34
4.04
2.00
f1 (ppm)

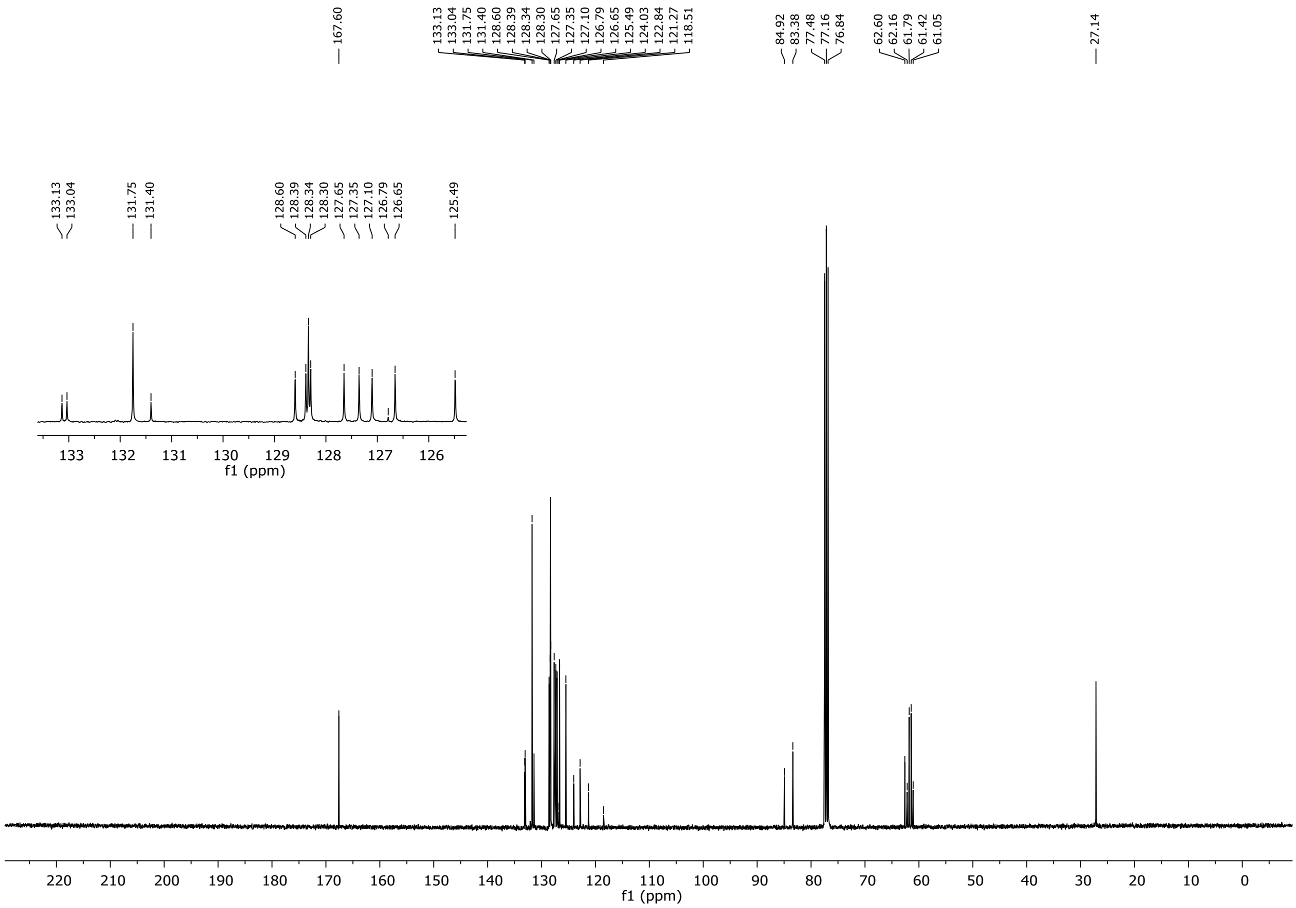
167.60
133.13
133.04
131.75
131.40
128.60
128.39
128.34
128.30
127.65
127.35
127.10
126.79
126.65
125.49
124.03
122.84
121.27
118.51
84.92
83.38
77.48
77.16
76.84
62.60
62.16
61.79
61.42
61.05
27.14
f1 (ppm)

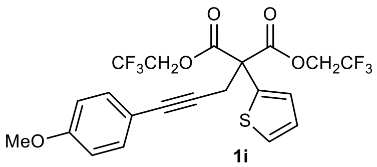
CF3CH2O
OCH2CF3
MeO
S

1i

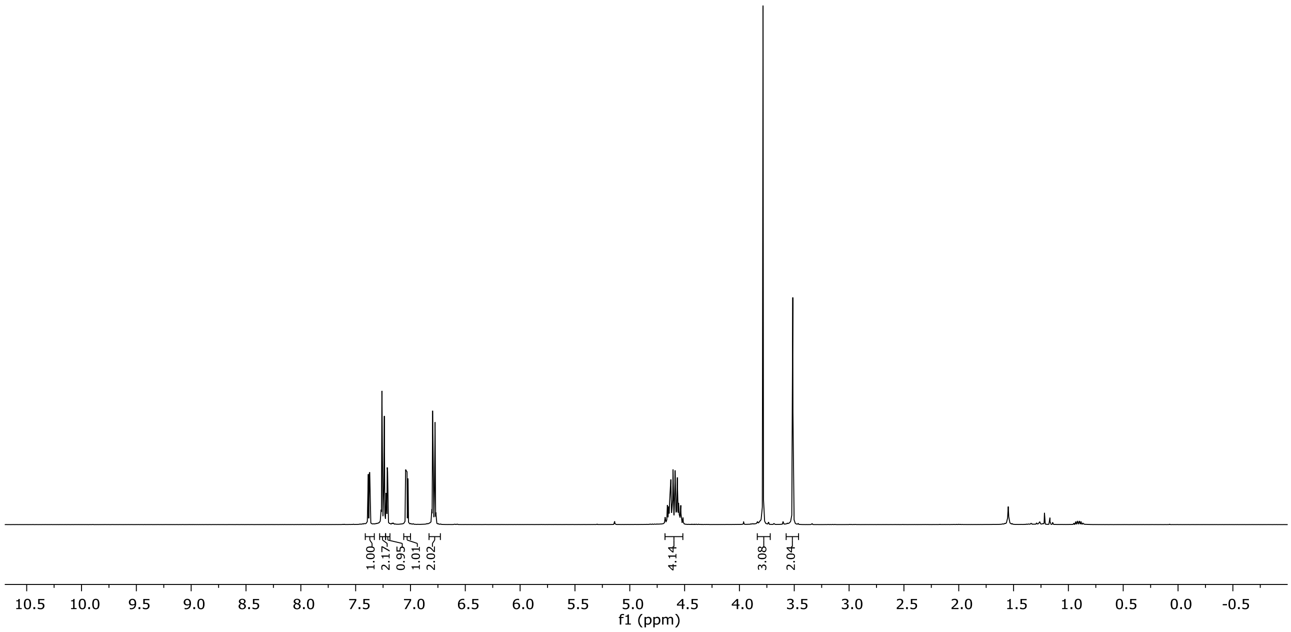
1.00
2.17
0.95
1.01
2.02
4.14
3.08
2.04
10.5 10.0 9.5 9.0 8.5 8.0 7.5 7.0 6.5 6.0 5.5 5.0 4.5 4.0 3.5 3.0 2.5 2.0 1.5 1.0 0.5 0.0 -0.5
f1 (ppm)

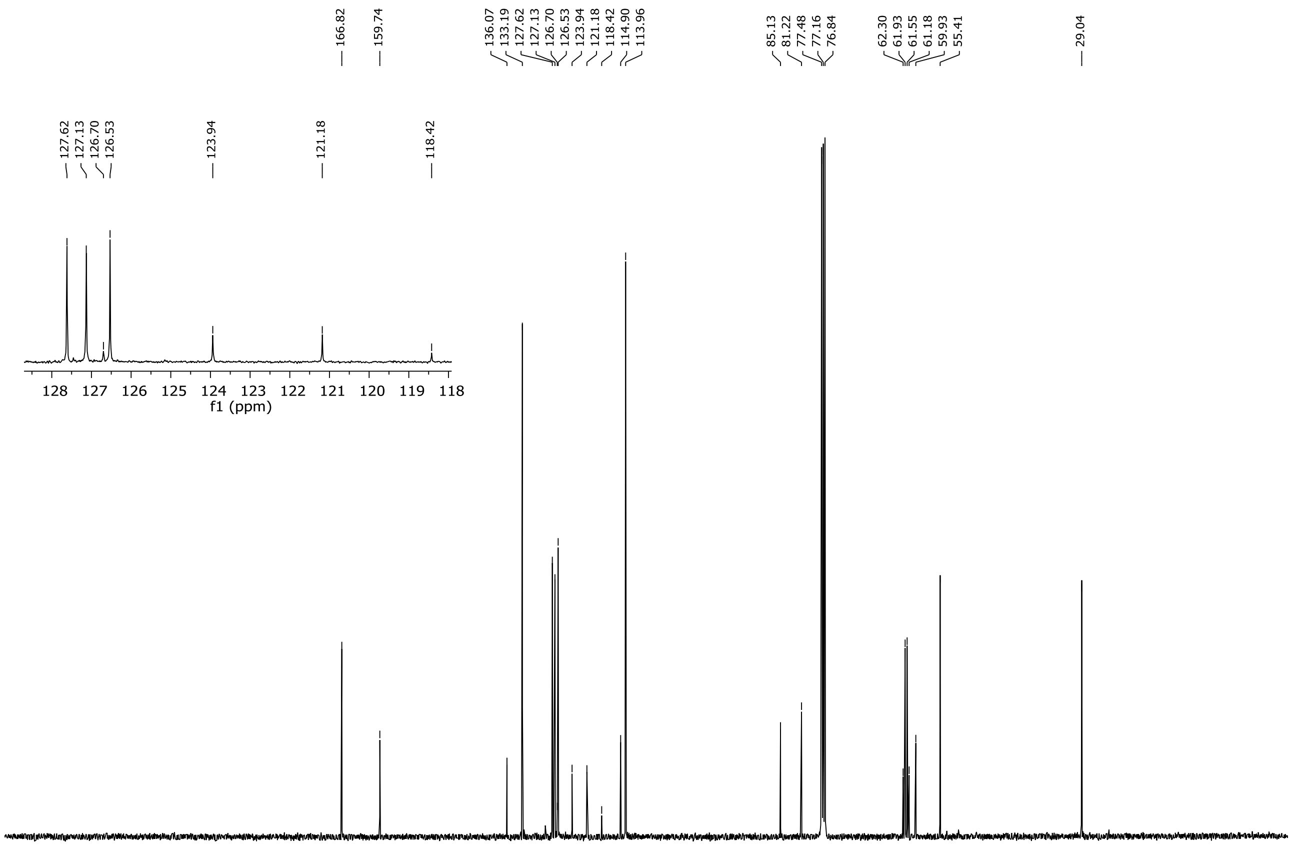
166.82
159.74
136.07
133.19
127.62
127.13
126.70
126.53
123.94
121.18
118.42
114.90
113.96
85.13
81.22
77.48
77.16
76.84
62.30
61.93
61.55
61.18
59.93
55.41
29.04
127.62
127.13
126.70
126.53
123.94
121.18
118.42
128 127 126 125 124 123 122 121 120 119 118
f1 (ppm)
220 210 200 190 180 170 160 150 140 130 120 110 100 90 80 70 60 50 40 30 20 10 0
f1 (ppm)

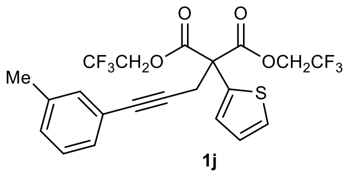

1j

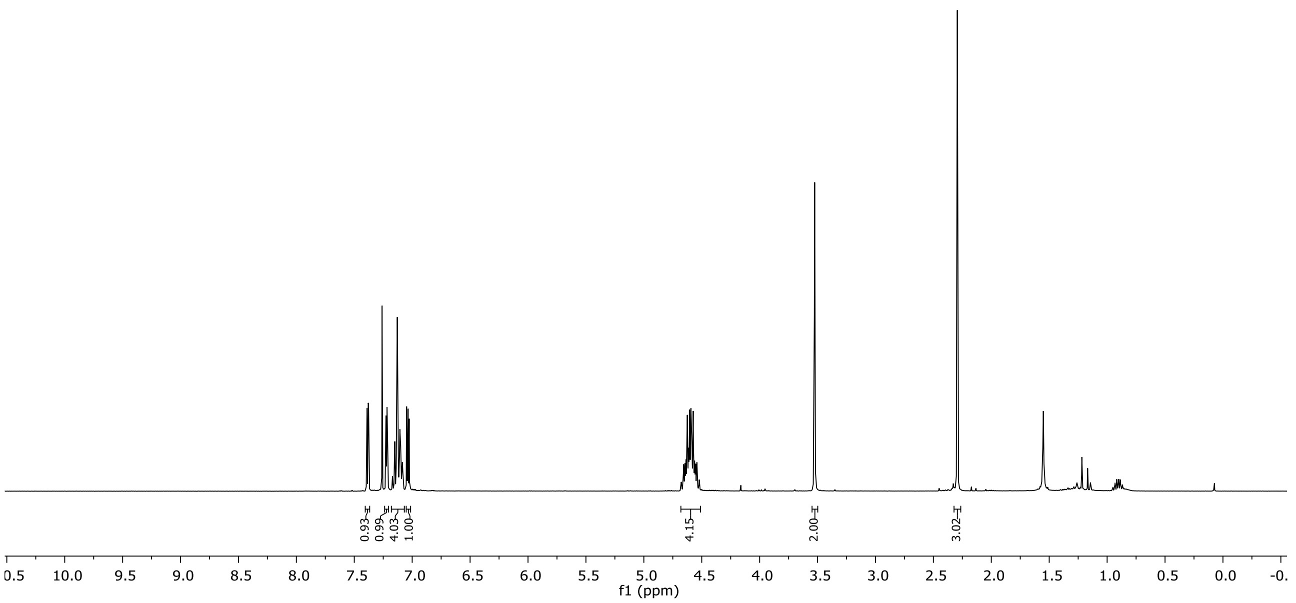
0.93
0.99
4.03
1.00
4.15
2.00
3.02
f1 (ppm)

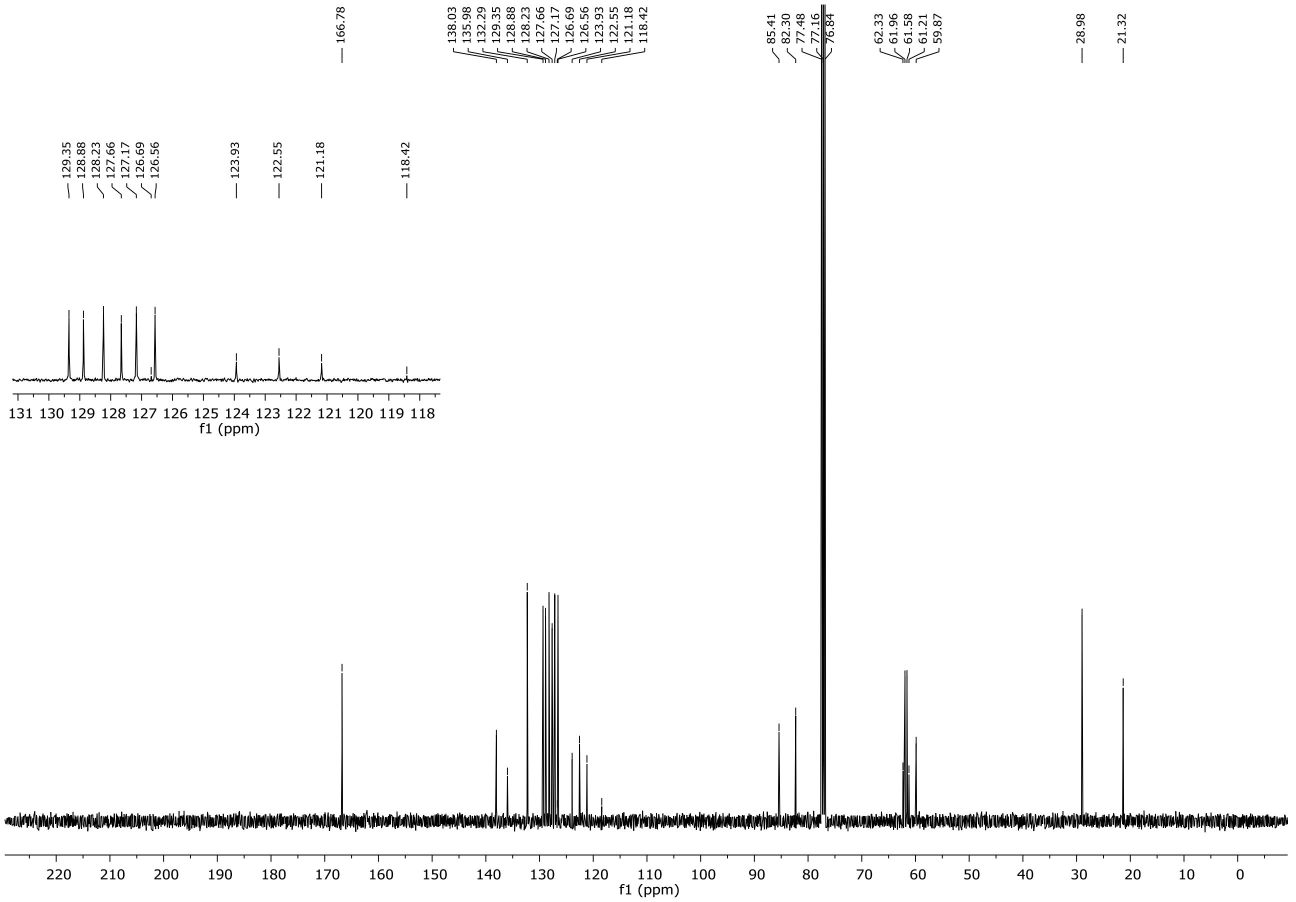
166.78
138.03
135.98
132.29
129.35
128.88
128.23
127.66
127.17
126.69
126.56
123.93
122.55
121.18
118.42
85.41
82.30
77.48
77.16
76.84
62.33
61.96
61.58
61.21
59.87
28.98
21.32
f1 (ppm)

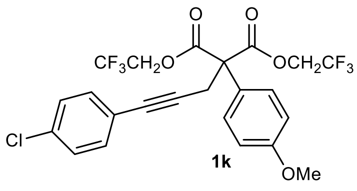
CF3CH2O
OCH2CF3
Cl
OMe

1k

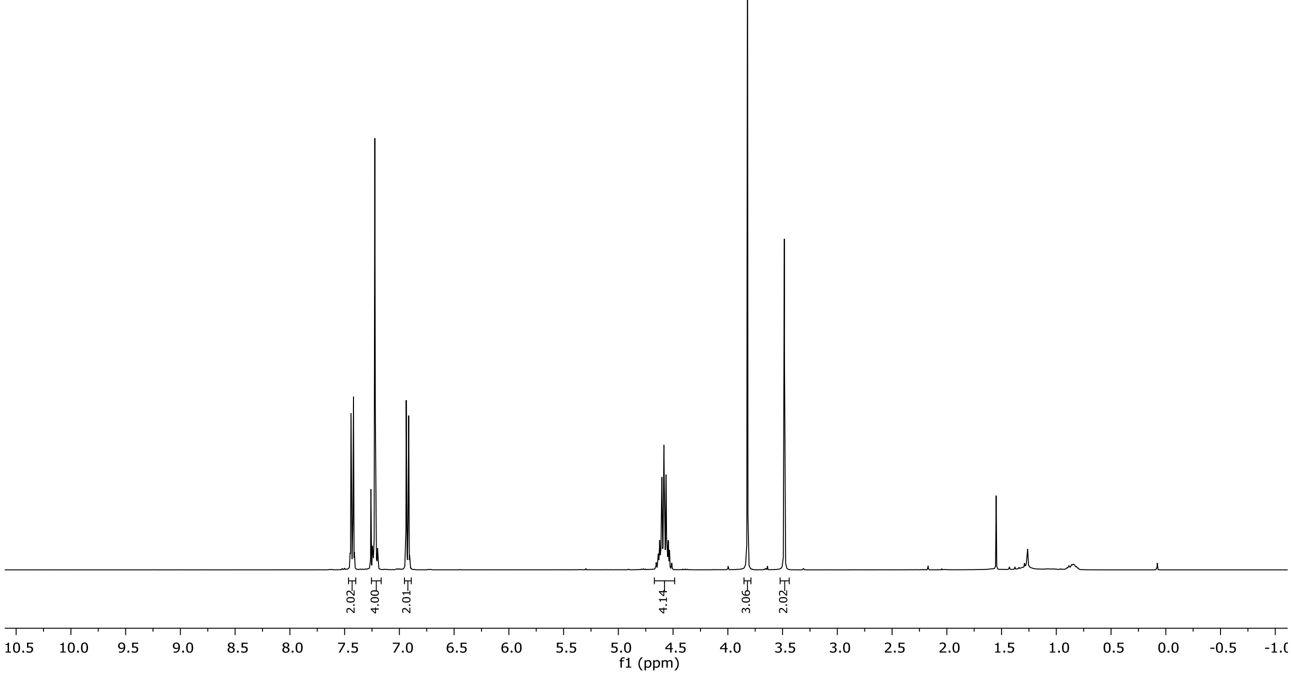
2.02
4.00
2.01
4.14
3.06
2.02
f1 (ppm)

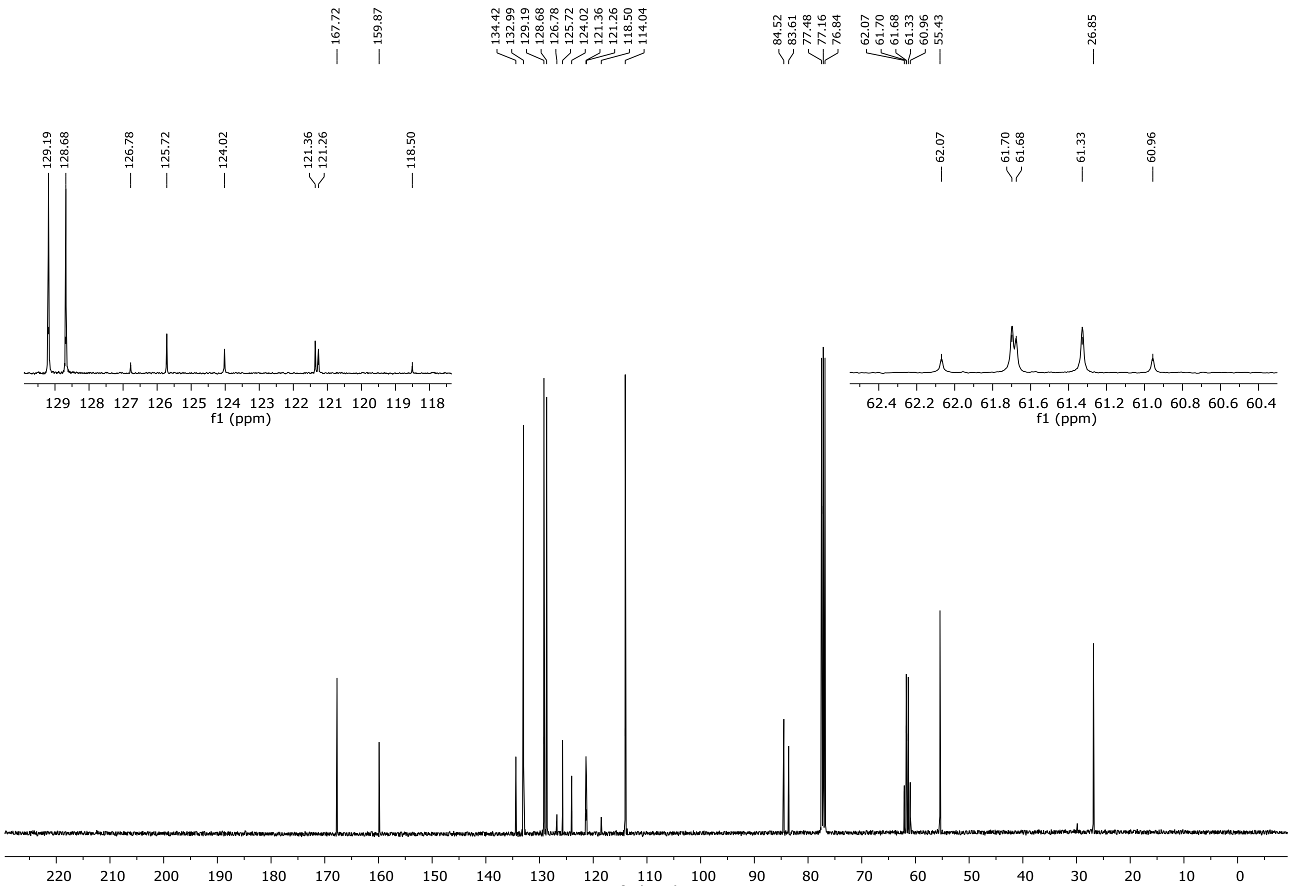
167.72
159.87
134.42
132.99
129.19
128.68
126.78
125.72
124.02
121.36
121.26
118.50
114.04
84.52
83.61
77.48
77.16
76.84
62.07
61.70
61.68
61.33
60.96
55.43
26.85
f1 (ppm)

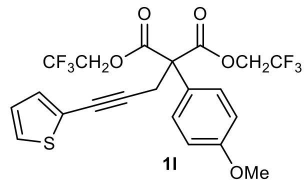
CF3CH2O
OCH2CF3
S
OMe

1l

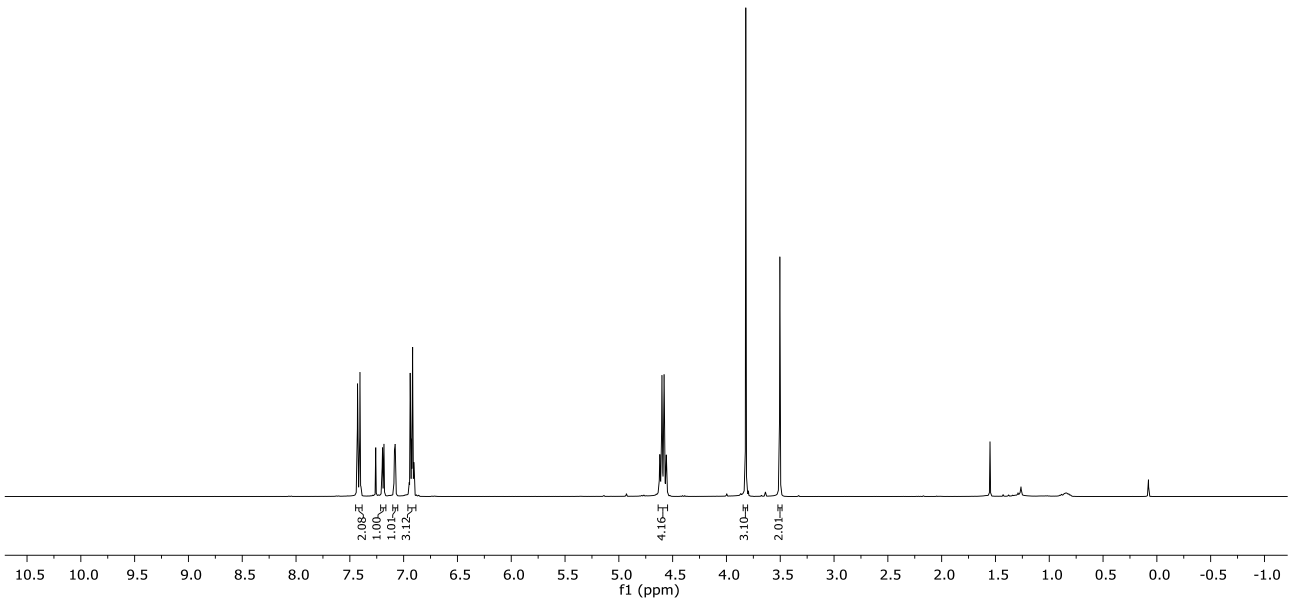
2.08
1.00
1.01
3.12
4.16
3.10
2.01
f1 (ppm)

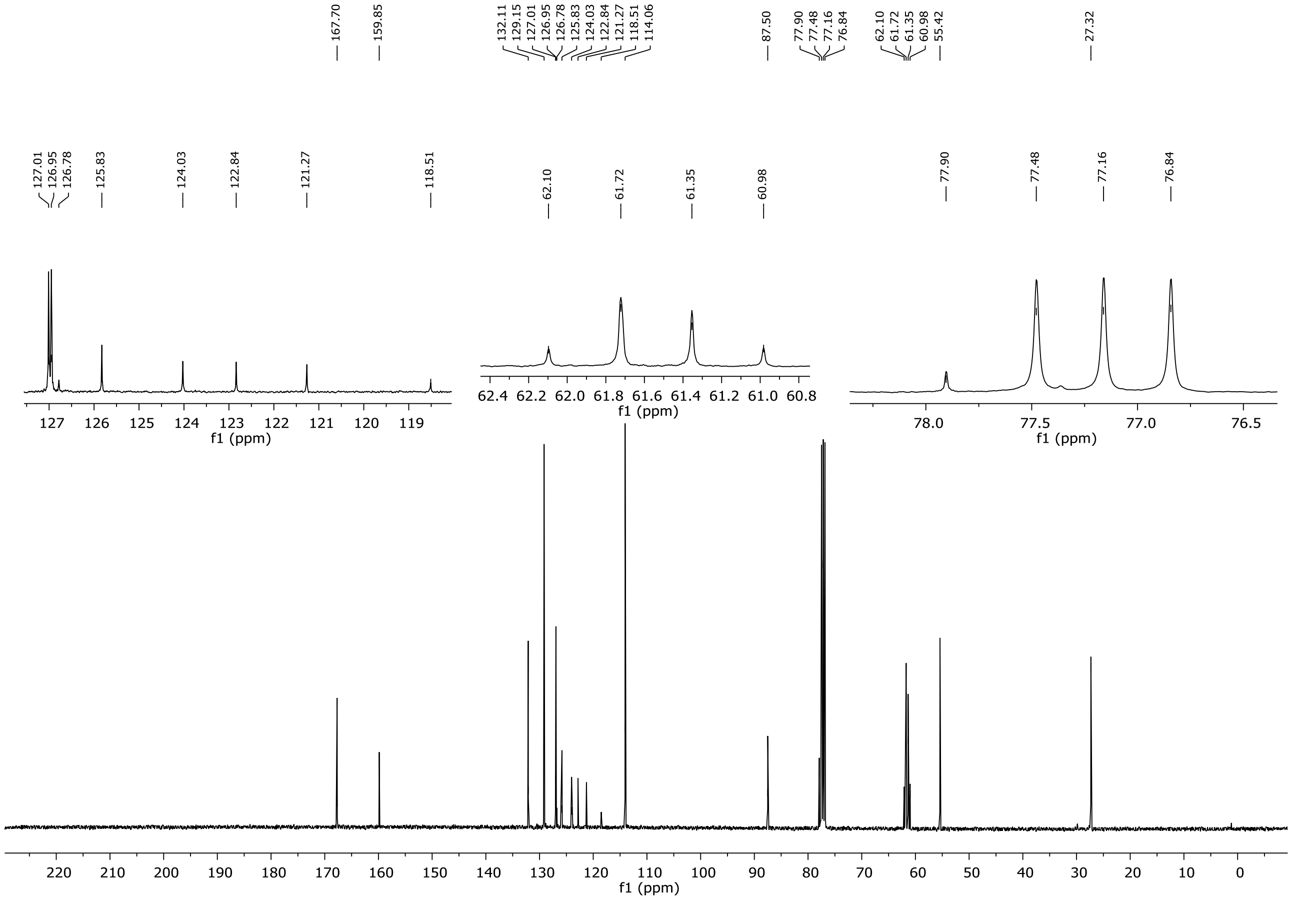
167.70
159.85
132.11
129.15
127.01
126.95
126.78
125.83
124.03
122.84
121.27
118.51
114.06
87.50
77.90
77.48
77.16
76.84
62.10
61.72
61.35
60.98
55.42
27.32
f1 (ppm)

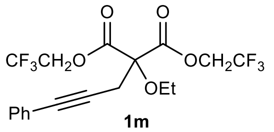

1m

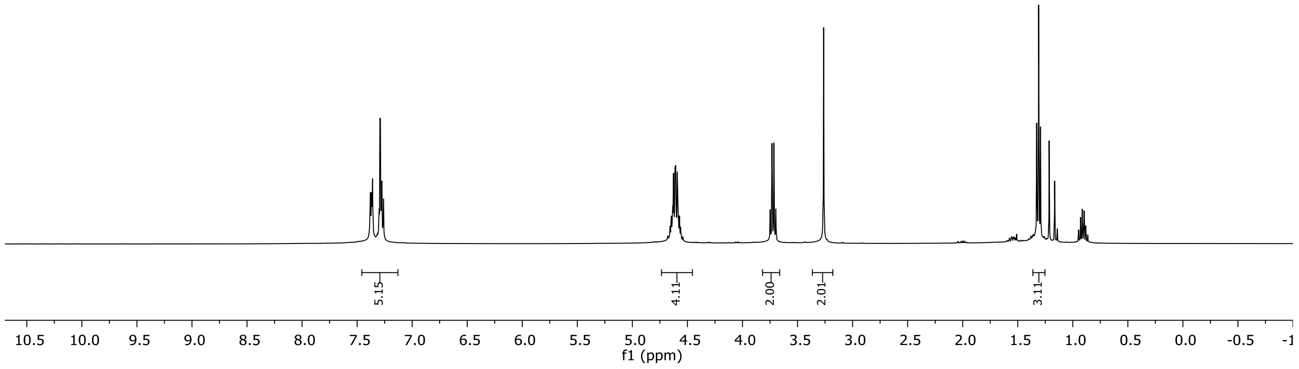

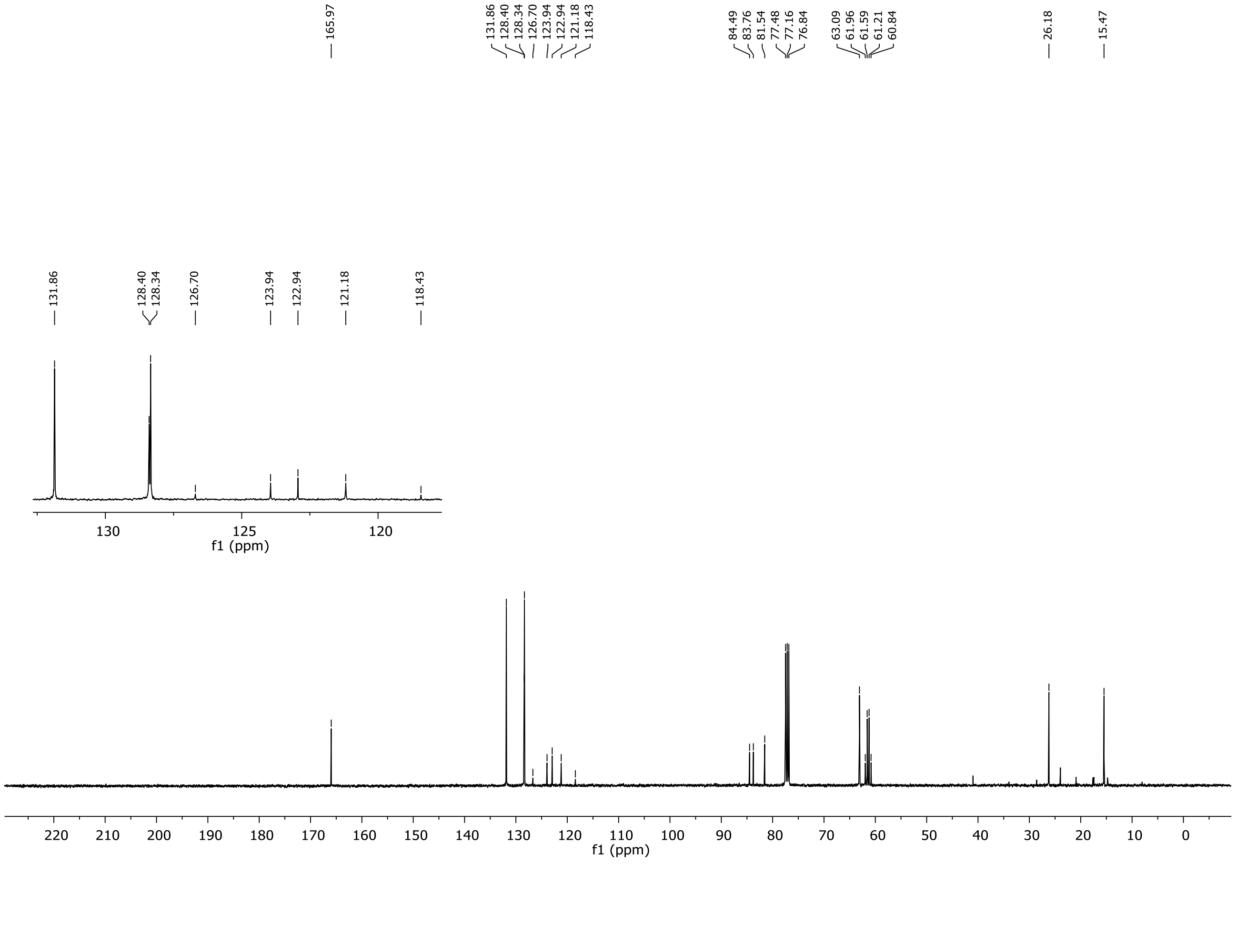

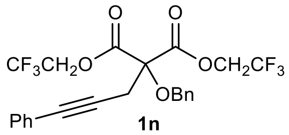
CF3CH2O
OCH2CF3
OBn
Ph

1n

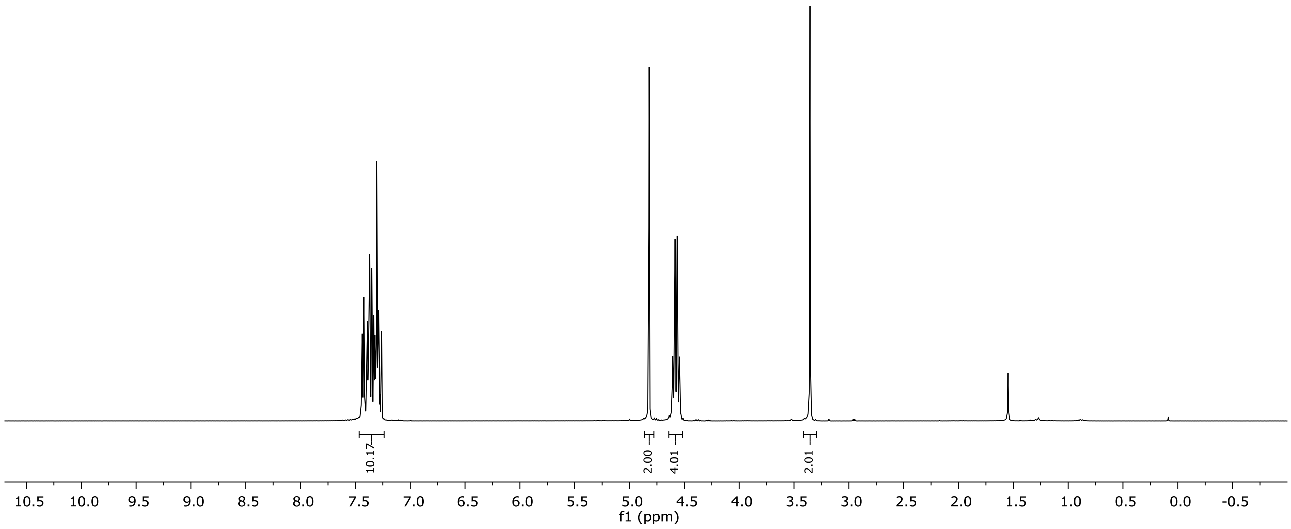
10.17
2.00
4.01
2.01
f1 (ppm)

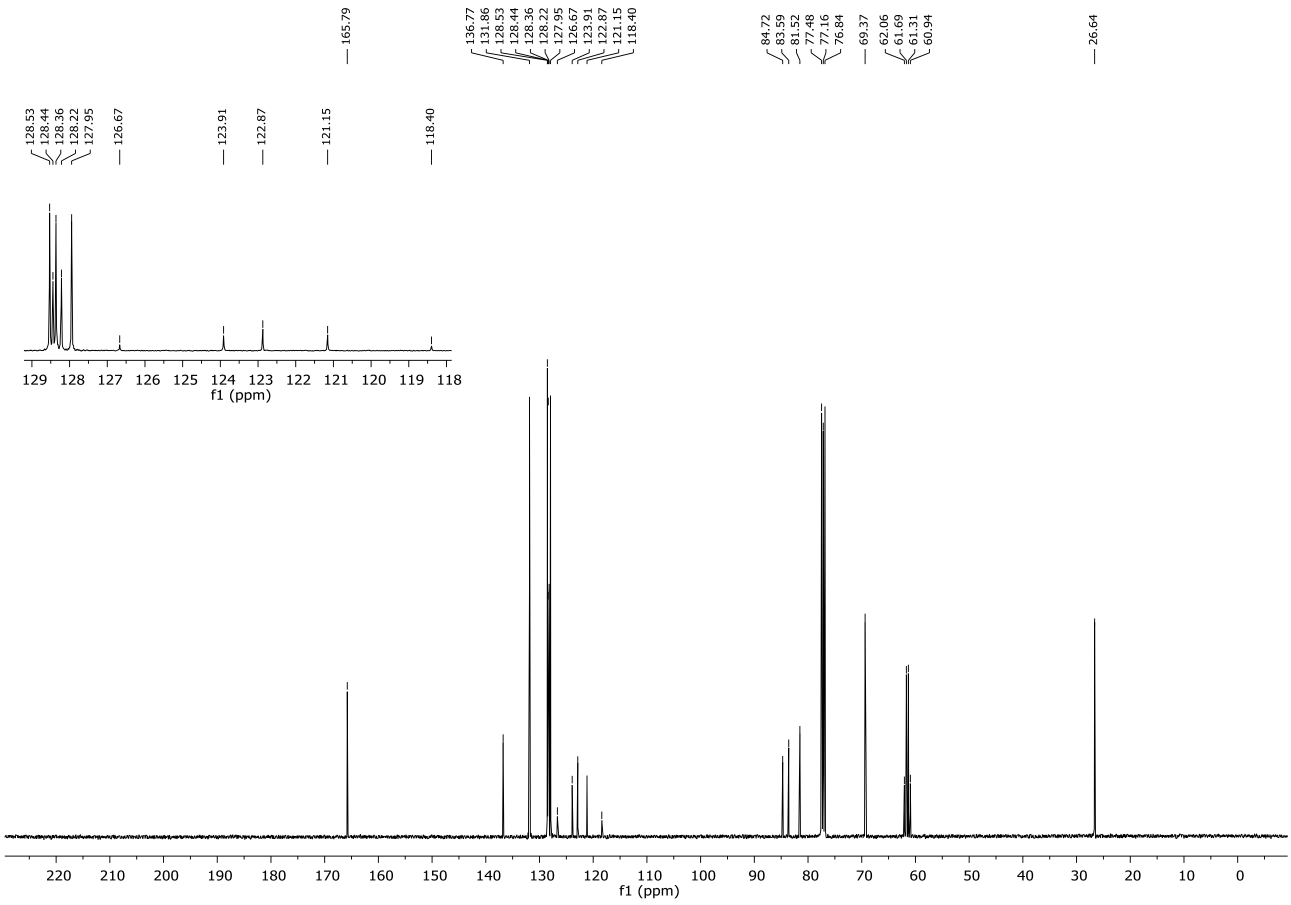
165.79
136.77
131.86
128.53
128.44
128.36
128.22
127.95
126.67
123.91
122.87
121.15
118.40
84.72
83.59
81.52
77.48
77.16
76.84
69.37
62.06
61.69
61.31
60.94
26.64
f1 (ppm)

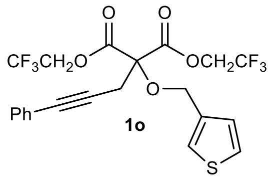

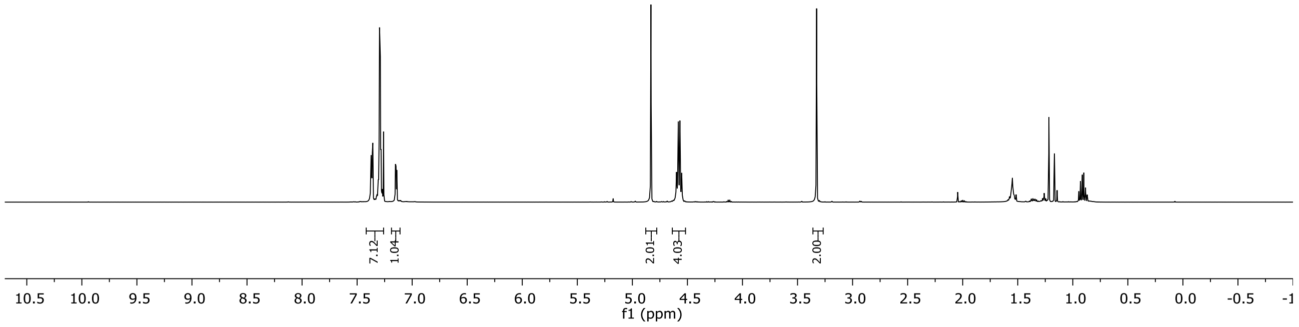

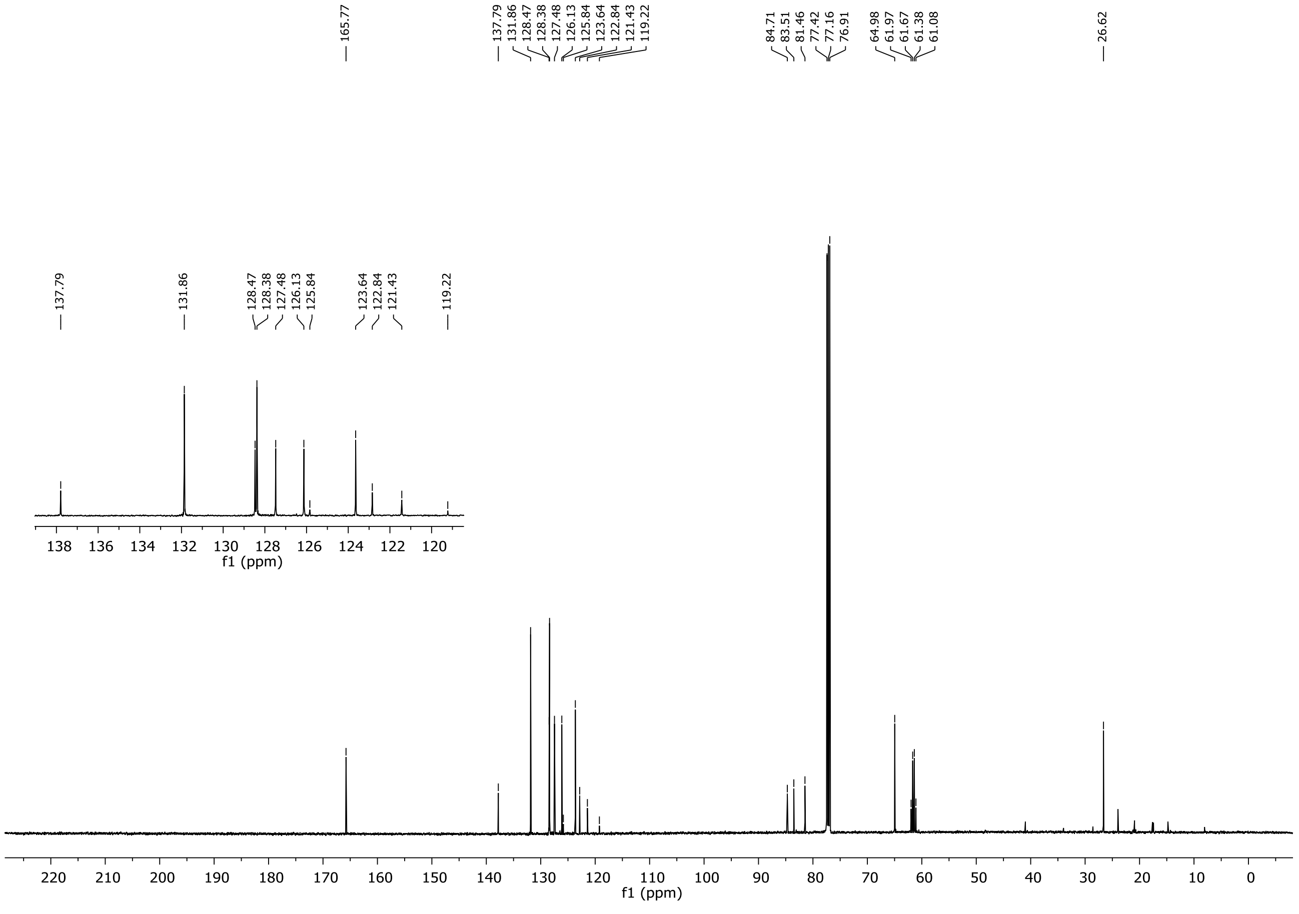

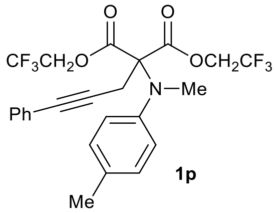
O
O
CF3CH2O
OCH2CF3
N—Me
Ph
Me

1p

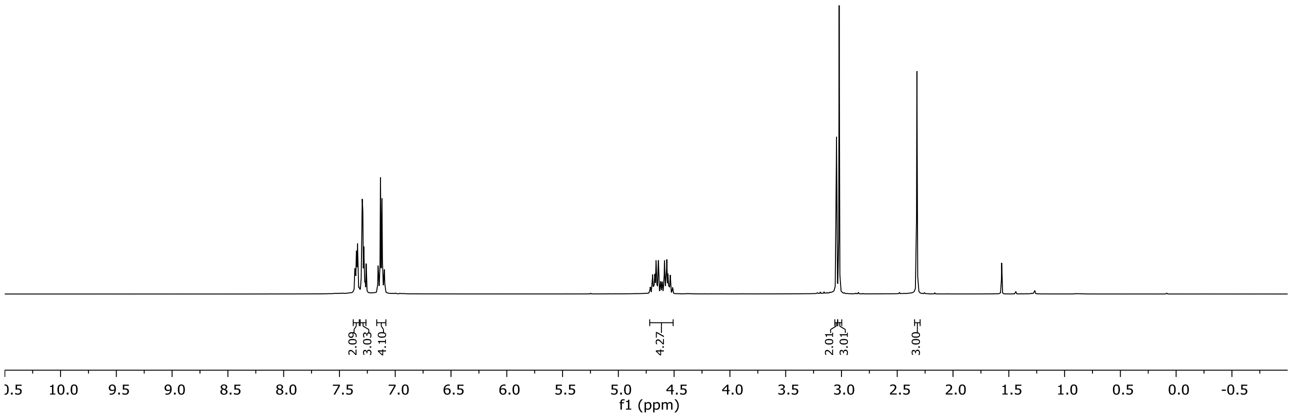
2.09
3.03
4.10
4.27
2.01
3.01
3.00
f1 (ppm)

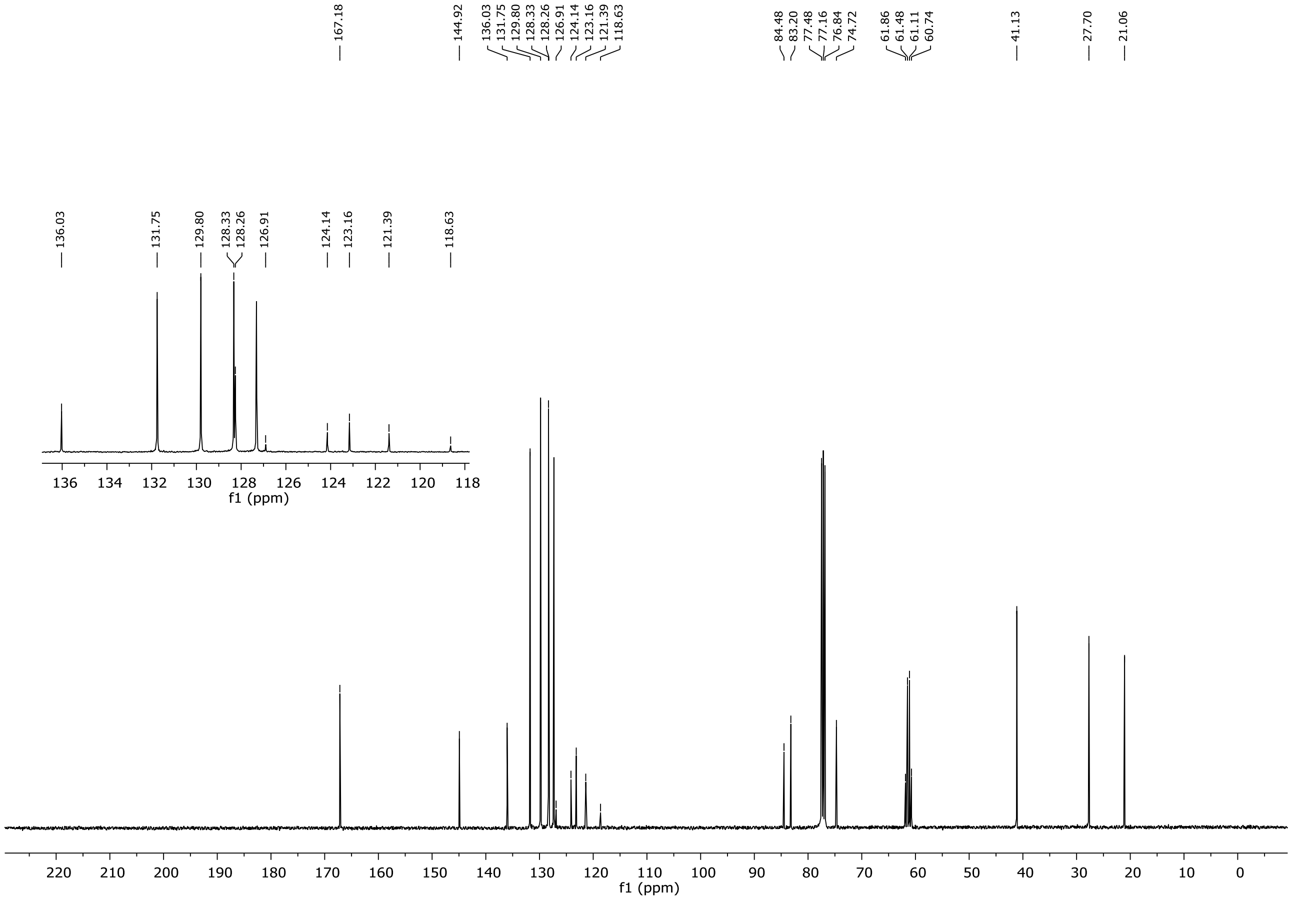
167.18
144.92
136.03
131.75
129.80
128.33
128.26
126.91
124.14
123.16
121.39
118.63
84.48
83.20
77.48
77.16
76.84
74.72
61.86
61.48
61.11
60.74
41.13
27.70
21.06
f1 (ppm)

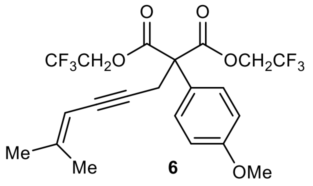

CF3CH2O
OCH2CF3
Me
Me
OMe

6

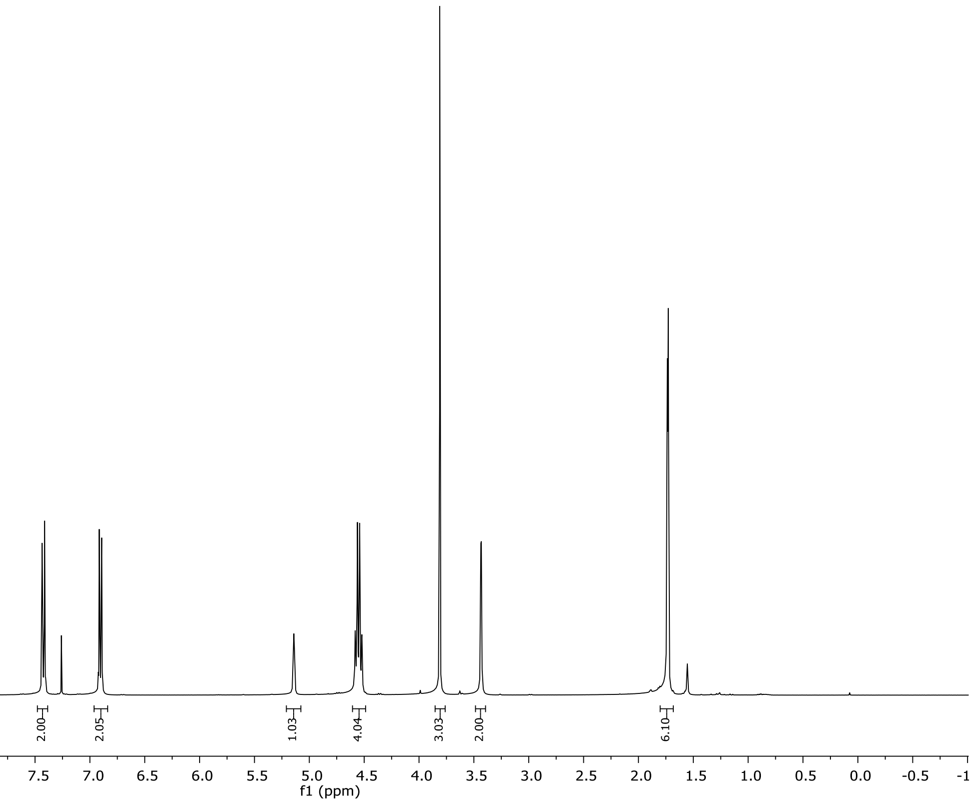

2.00
2.05
1.03
4.04
3.03
2.00
6.10
f1 (ppm)

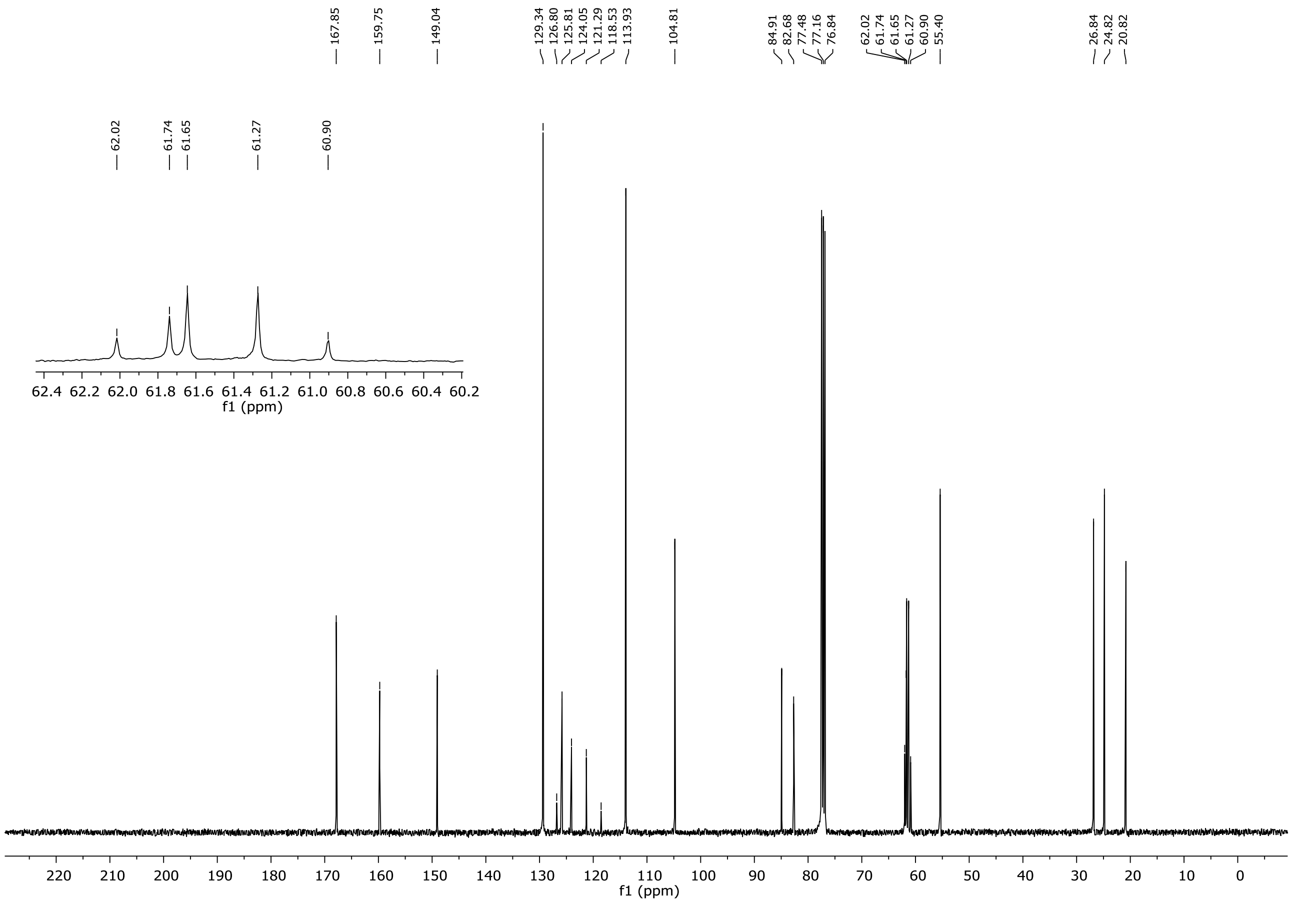

167.85
159.75
149.04
129.34
126.80
125.81
124.05
121.29
118.53
113.93
104.81
84.91
82.68
77.48
77.16
76.84
62.02
61.74
61.65
61.27
60.90
55.40
26.84
24.82
20.82
f1 (ppm)

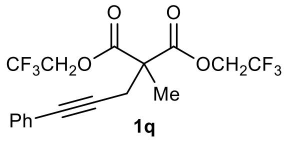

1q

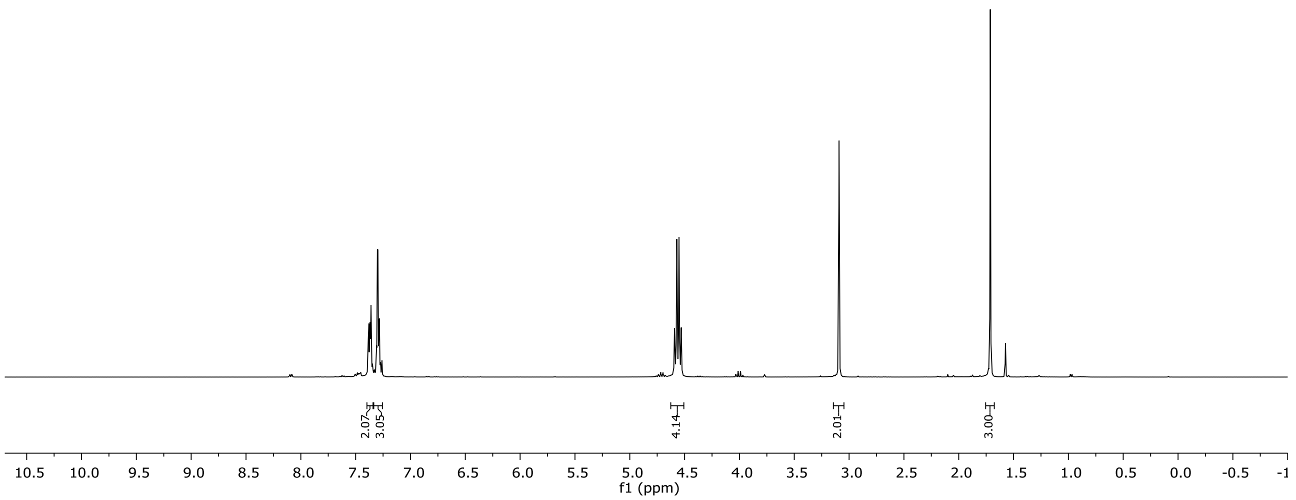

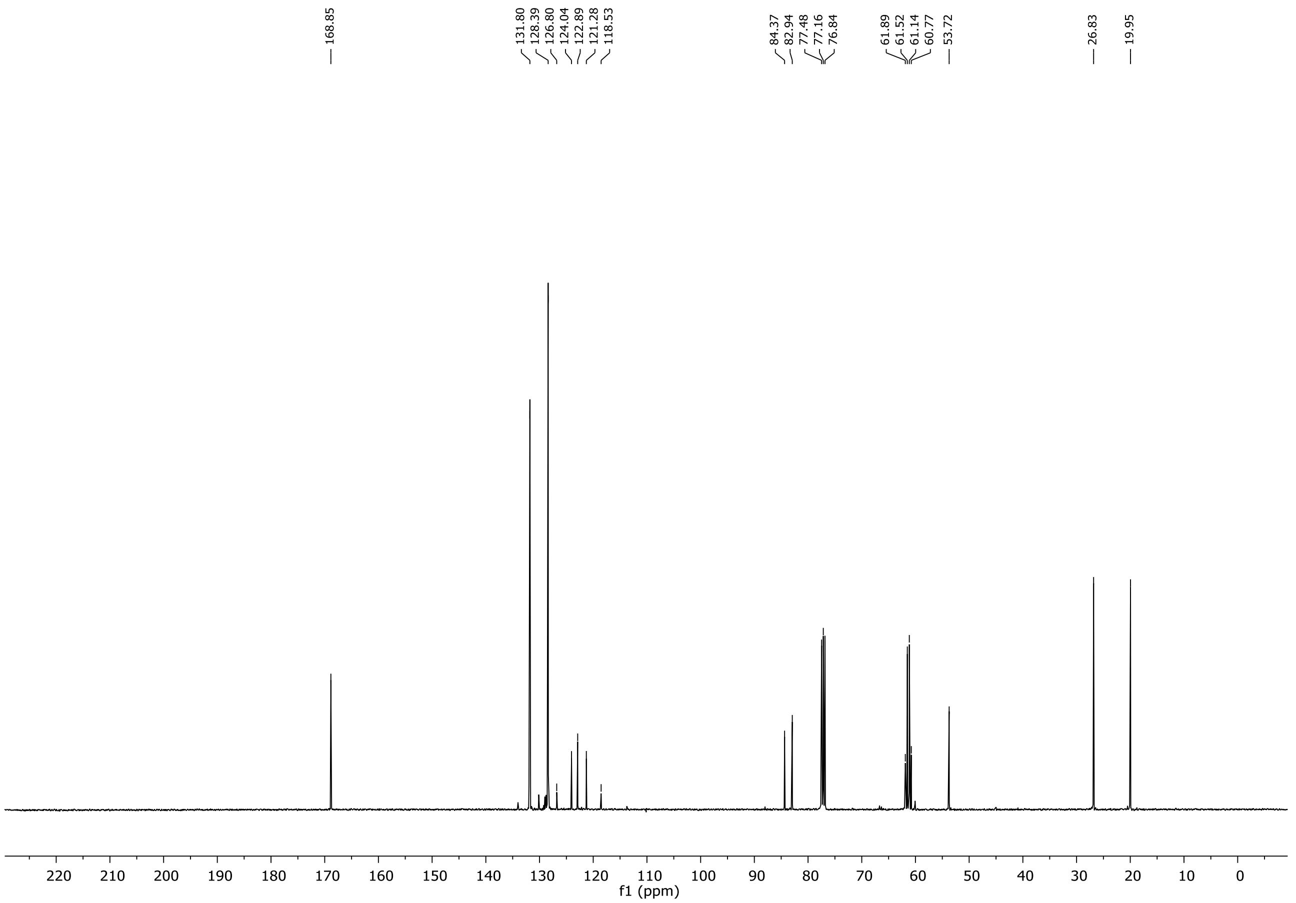

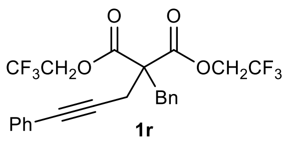
O
O
CF3CH2O
OCH2CF3
Bn
Ph

1r

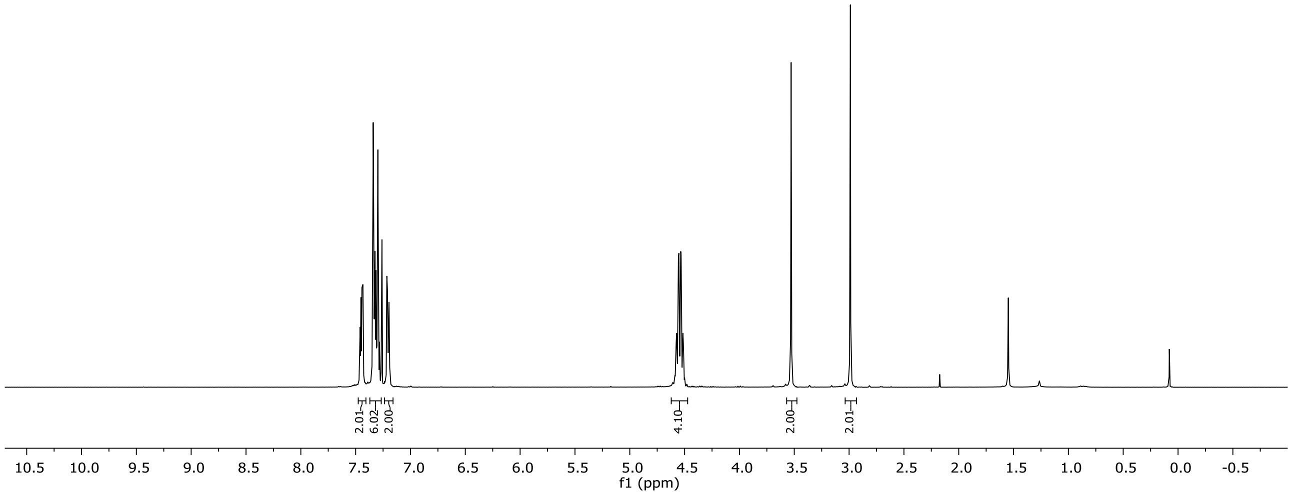
2.01
6.02
2.00
4.10
2.00
2.01
10.5 10.0 9.5 9.0 8.5 8.0 7.5 7.0 6.5 6.0 5.5 5.0 4.5 4.0 3.5 3.0 2.5 2.0 1.5 1.0 0.5 0.0 -0.5
f1 (ppm)

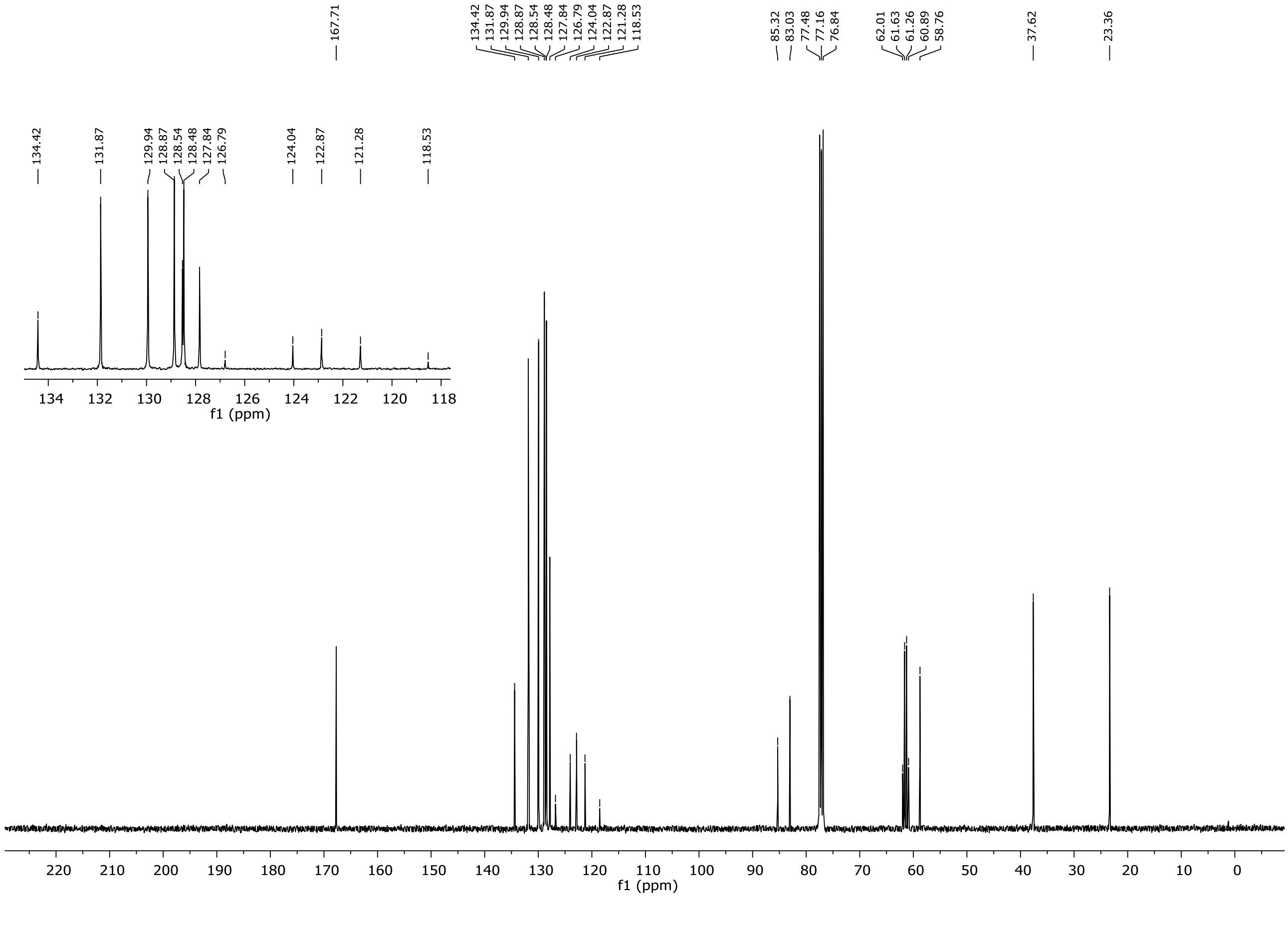
167.71
134.42 131.87 129.94 128.87 128.54 128.48 127.84 126.79 124.04 122.87 121.28 118.53
85.32 83.03 77.48 77.16 76.84
62.01 61.63 61.26 60.89 58.76
37.62
23.36
134 132 130 128 126 124 122 120 118
f1 (ppm)
220 210 200 190 180 170 160 150 140 130 120 110 100 90 80 70 60 50 40 30 20 10 0
f1 (ppm)

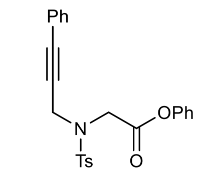
Ph
OPh
N
Ts
O

10

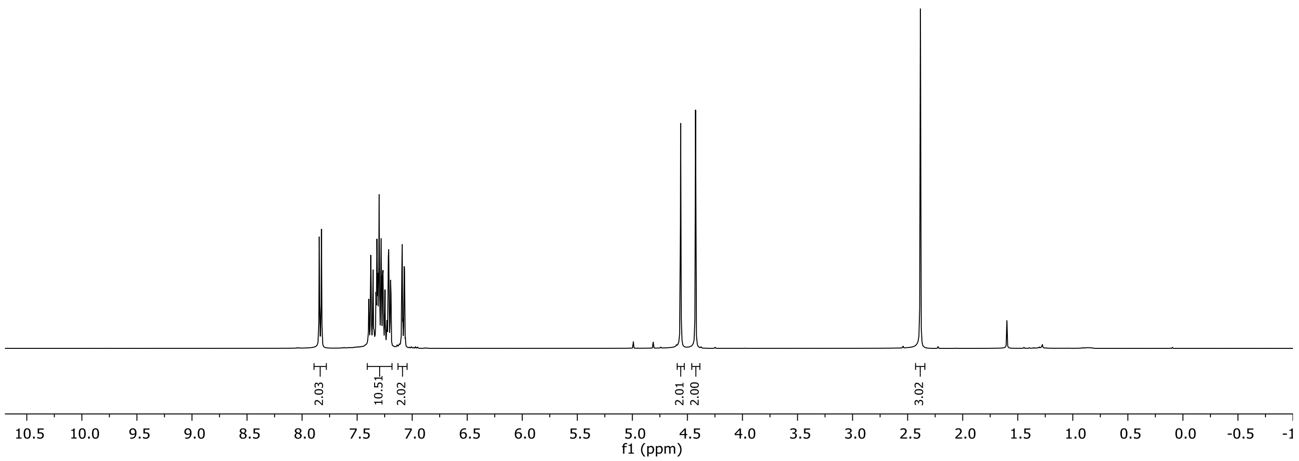
2.03
10.51
2.02
2.01
2.00
3.02
f1 (ppm)

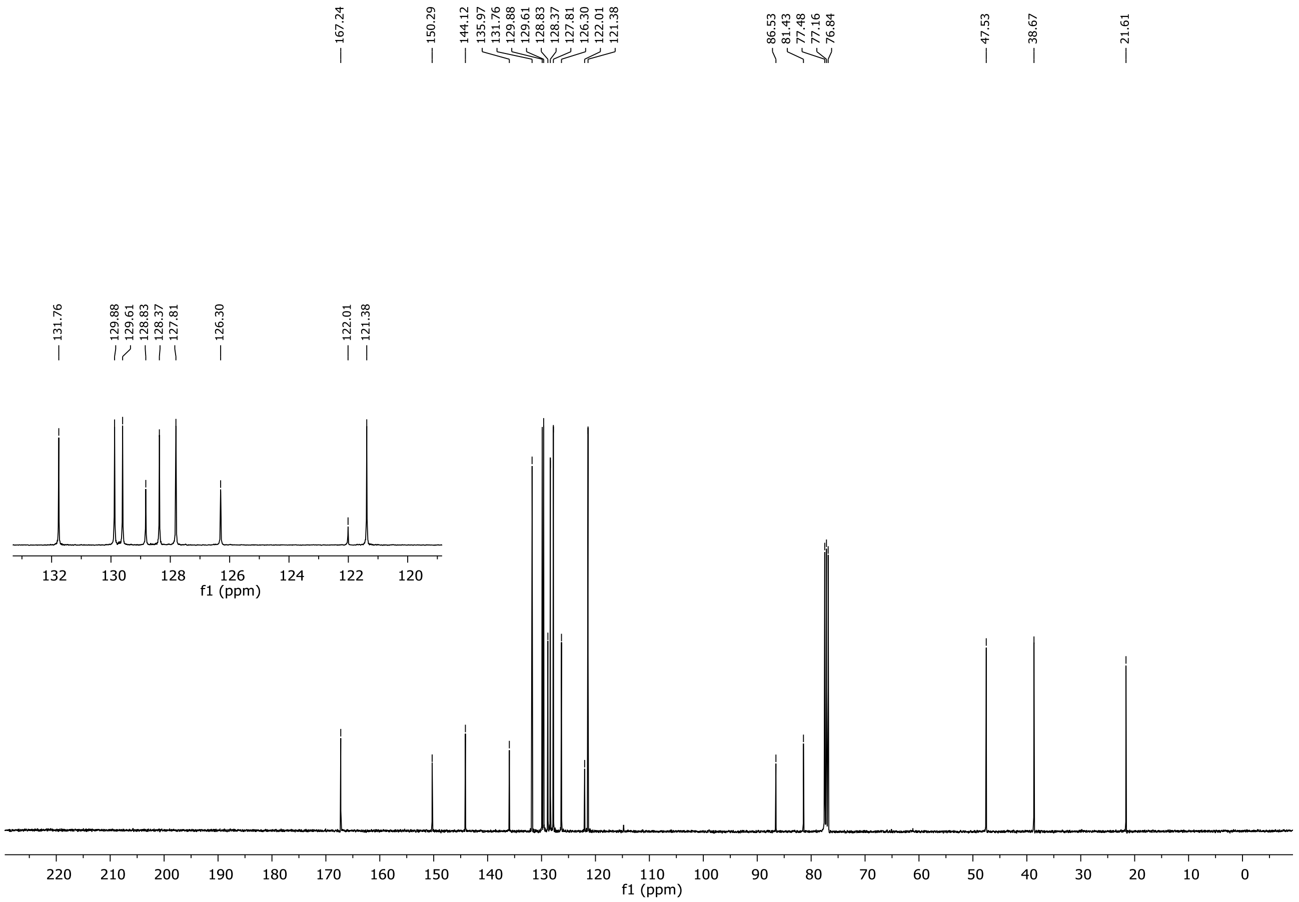
167.24
150.29
144.12
135.97
131.76
129.88
129.61
128.83
128.37
127.81
126.30
122.01
121.38
86.53
81.43
77.48
77.16
76.84
47.53
38.67
21.61
f1 (ppm)

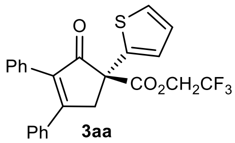
O
S
Ph
$CO_2CH_2CF_3$
Ph

3aa

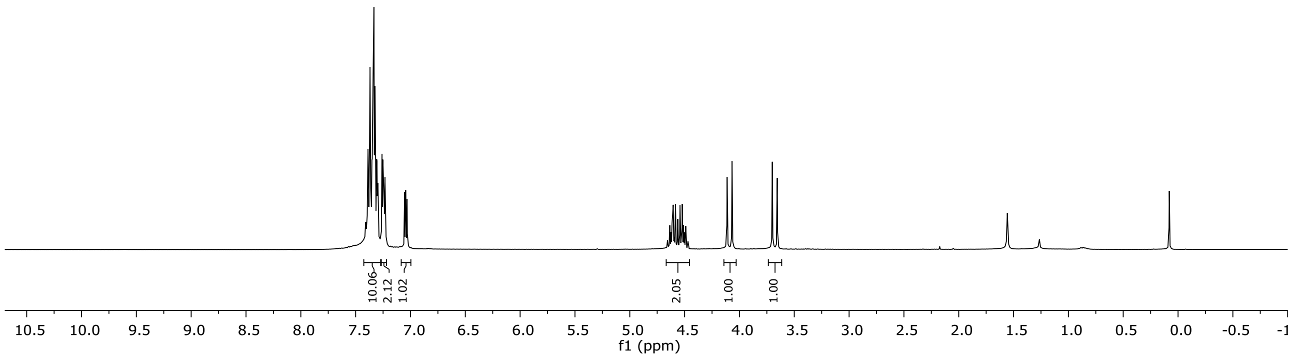
10.06
2.12
1.02
2.05
1.00
1.00
f1 (ppm)

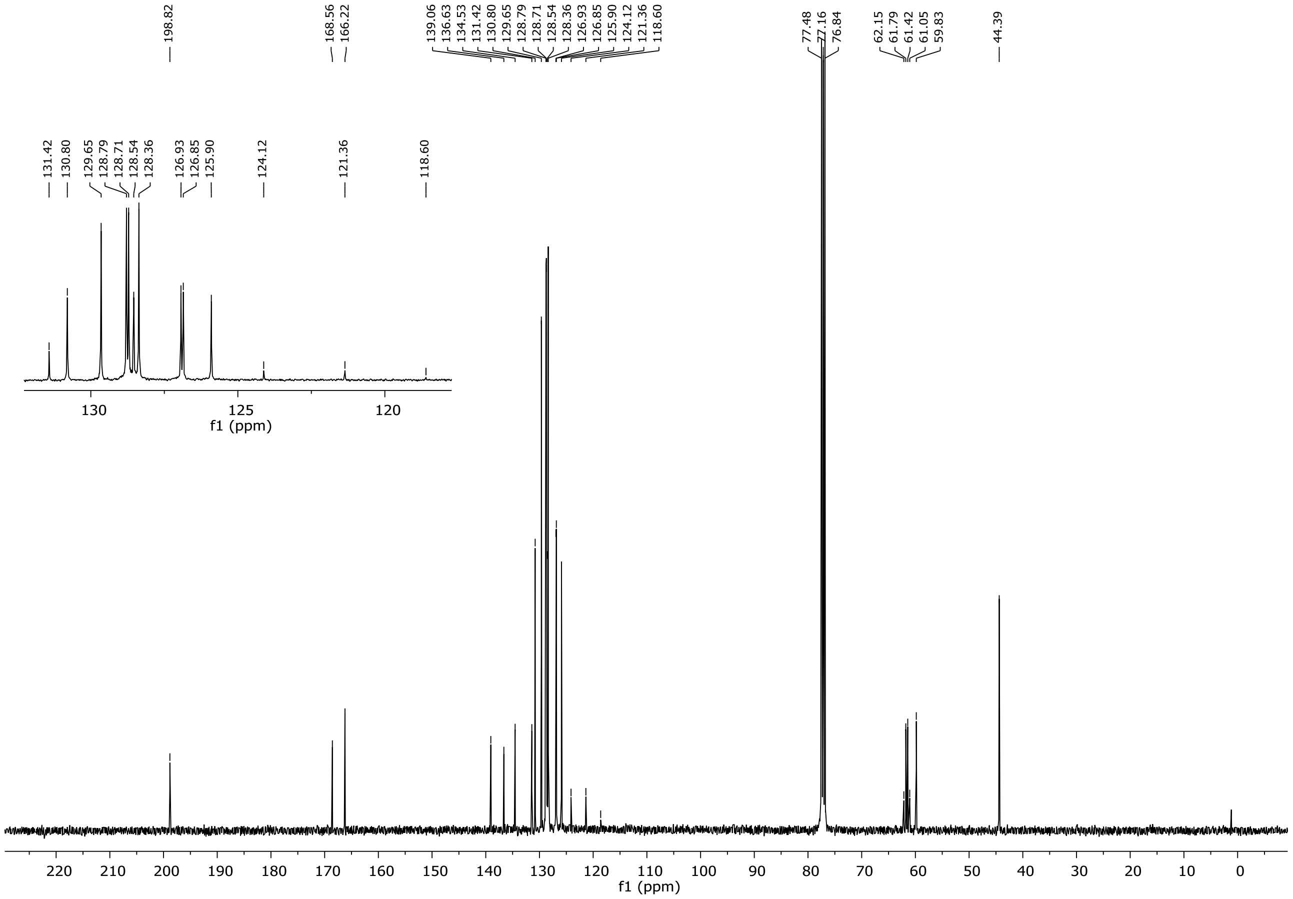
198.82
168.56
166.22
139.06
136.63
134.53
131.42
130.80
129.65
128.79
128.71
128.54
128.36
126.93
126.85
125.90
124.12
121.36
118.60
77.48
77.16
76.84
62.15
61.79
61.42
61.05
59.83
44.39
f1 (ppm)

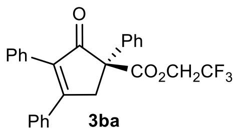
O
Ph
Ph
$CO_2CH_2CF_3$
Ph

3ba

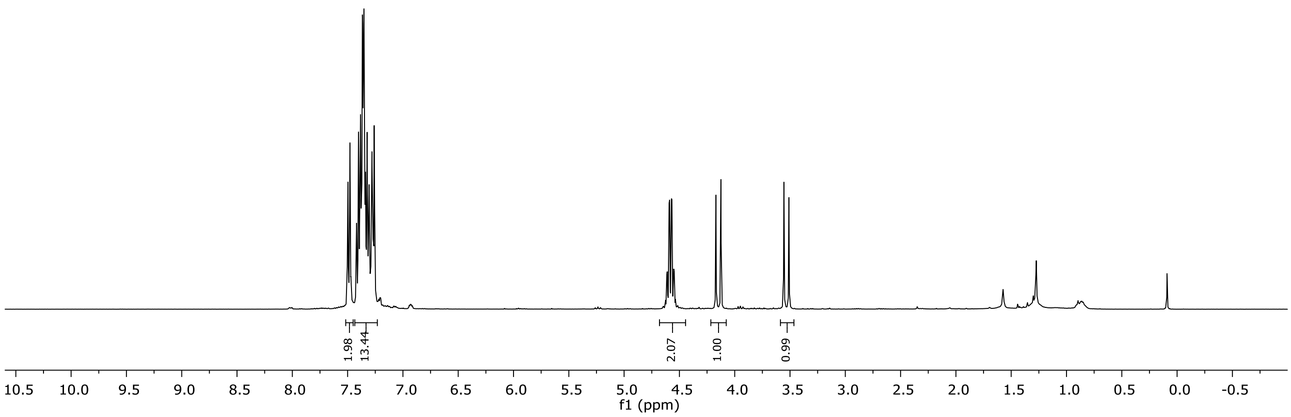
1.98
13.44
2.07
1.00
0.99
f1 (ppm)

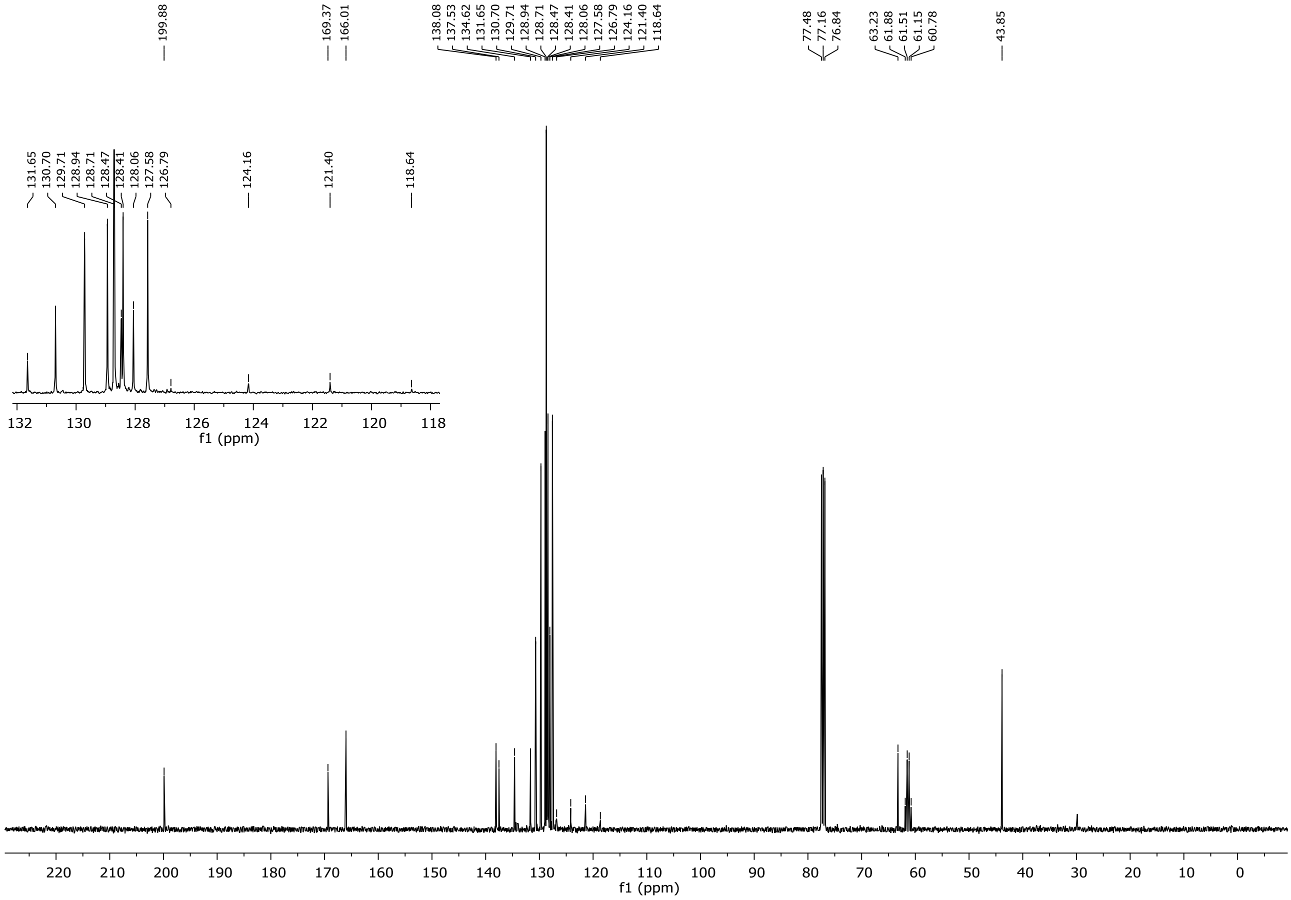
199.88
169.37
166.01
138.08
137.53
134.62
131.65
130.70
129.71
128.94
128.71
128.47
128.41
128.06
127.58
126.79
124.16
121.40
118.64
77.48
77.16
76.84
63.23
61.88
61.51
61.15
60.78
43.85
f1 (ppm)

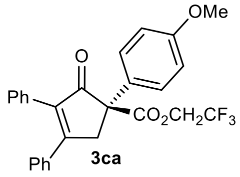
OMe
O
Ph
Ph
$CO_2CH_2CF_3$

3ca

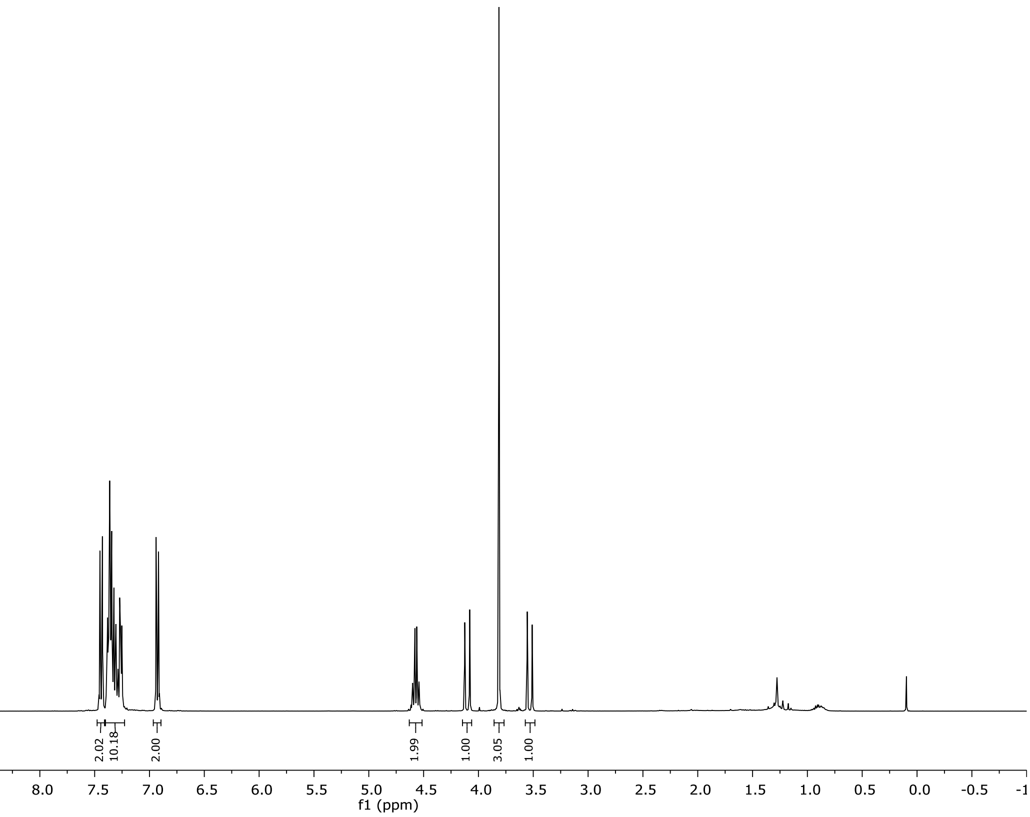
2.02
10.18
2.00
1.99
1.00
3.05
1.00
f1 (ppm)

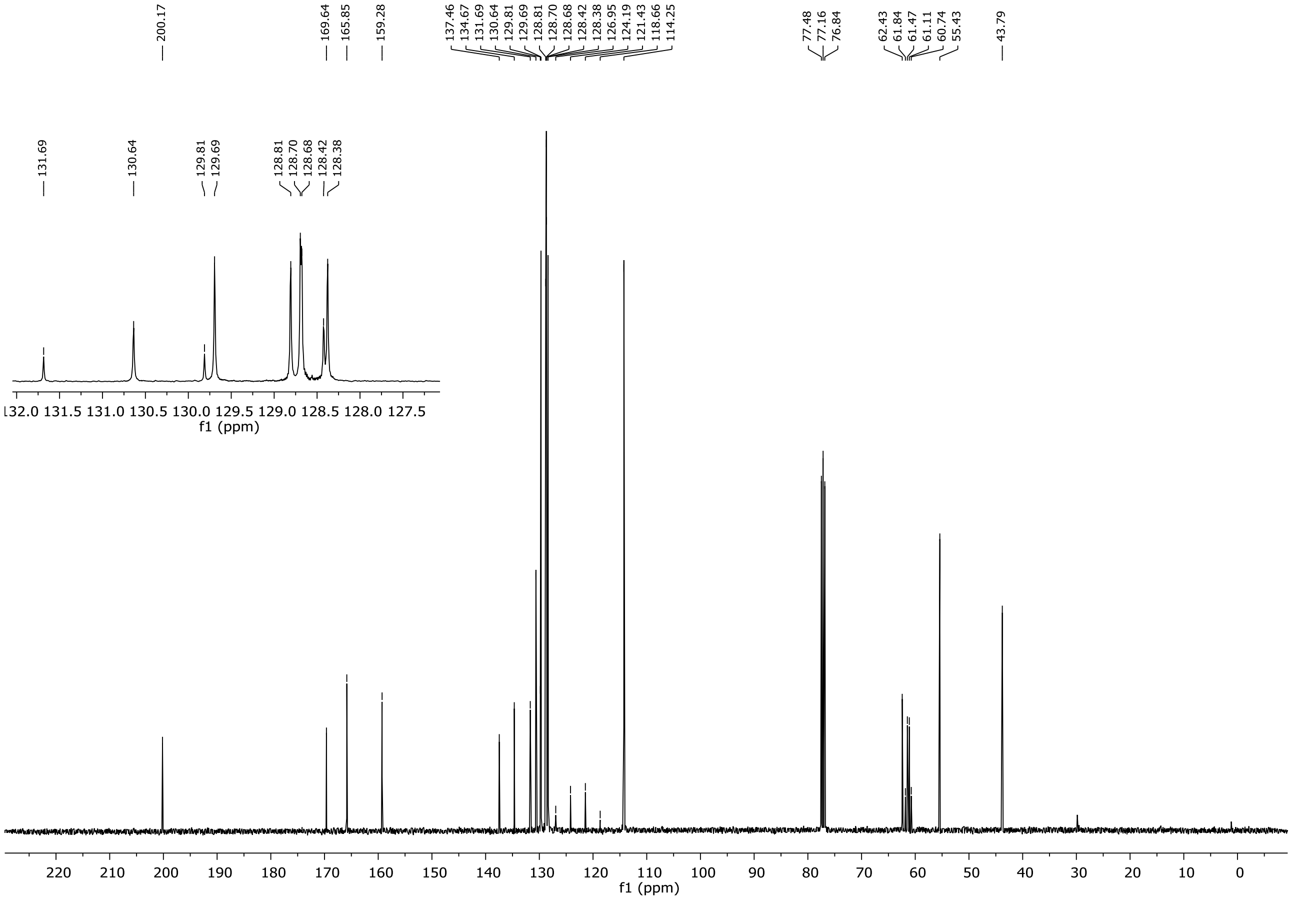
200.17
169.64
165.85
159.28
137.46
134.67
131.69
130.64
129.81
129.69
128.81
128.70
128.68
128.42
128.38
126.95
124.19
121.43
118.66
114.25
77.48
77.16
76.84
62.43
61.84
61.47
61.11
60.74
55.43
43.79
f1 (ppm)

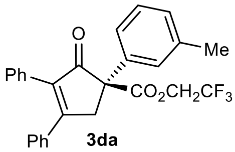
O
Me
Ph
$CO_2CH_2CF_3$
Ph

3da

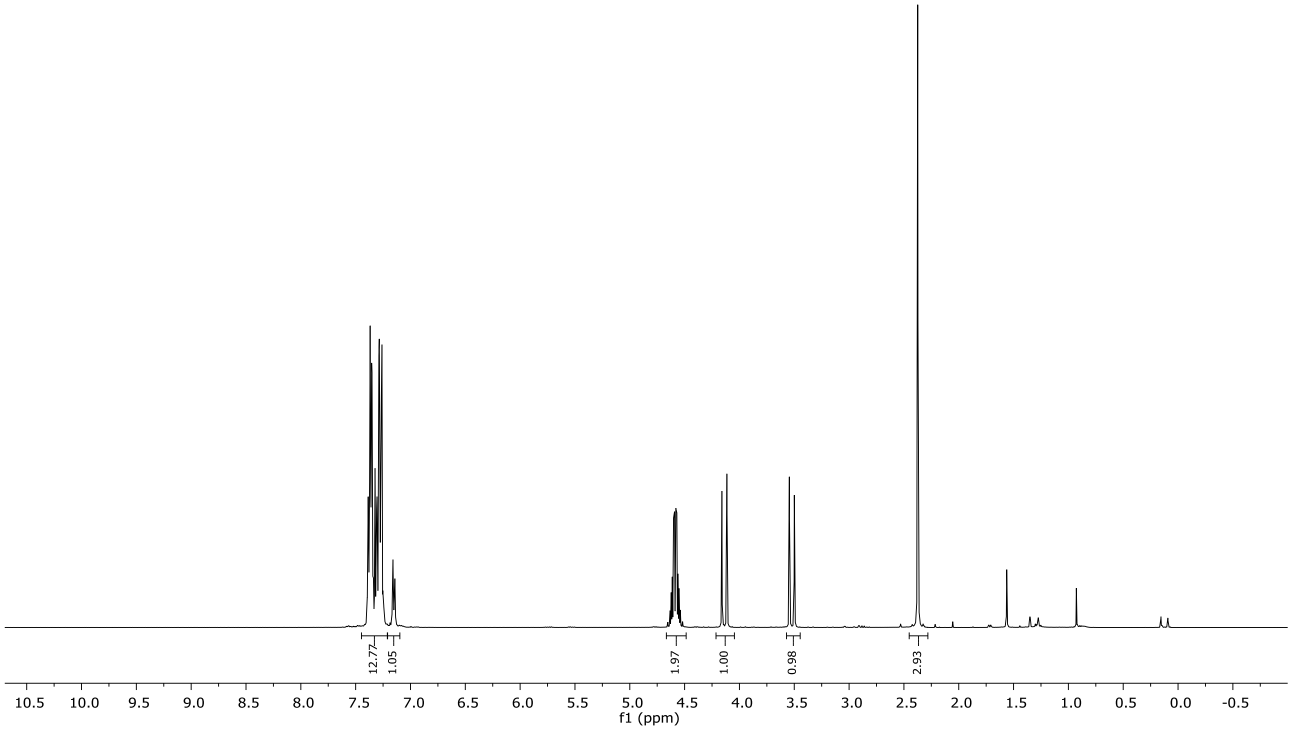
12.77
1.05
1.97
1.00
0.98
2.93
10.5 10.0 9.5 9.0 8.5 8.0 7.5 7.0 6.5 6.0 5.5 5.0 4.5 4.0 3.5 3.0 2.5 2.0 1.5 1.0 0.5 0.0 -0.5
f1 (ppm)

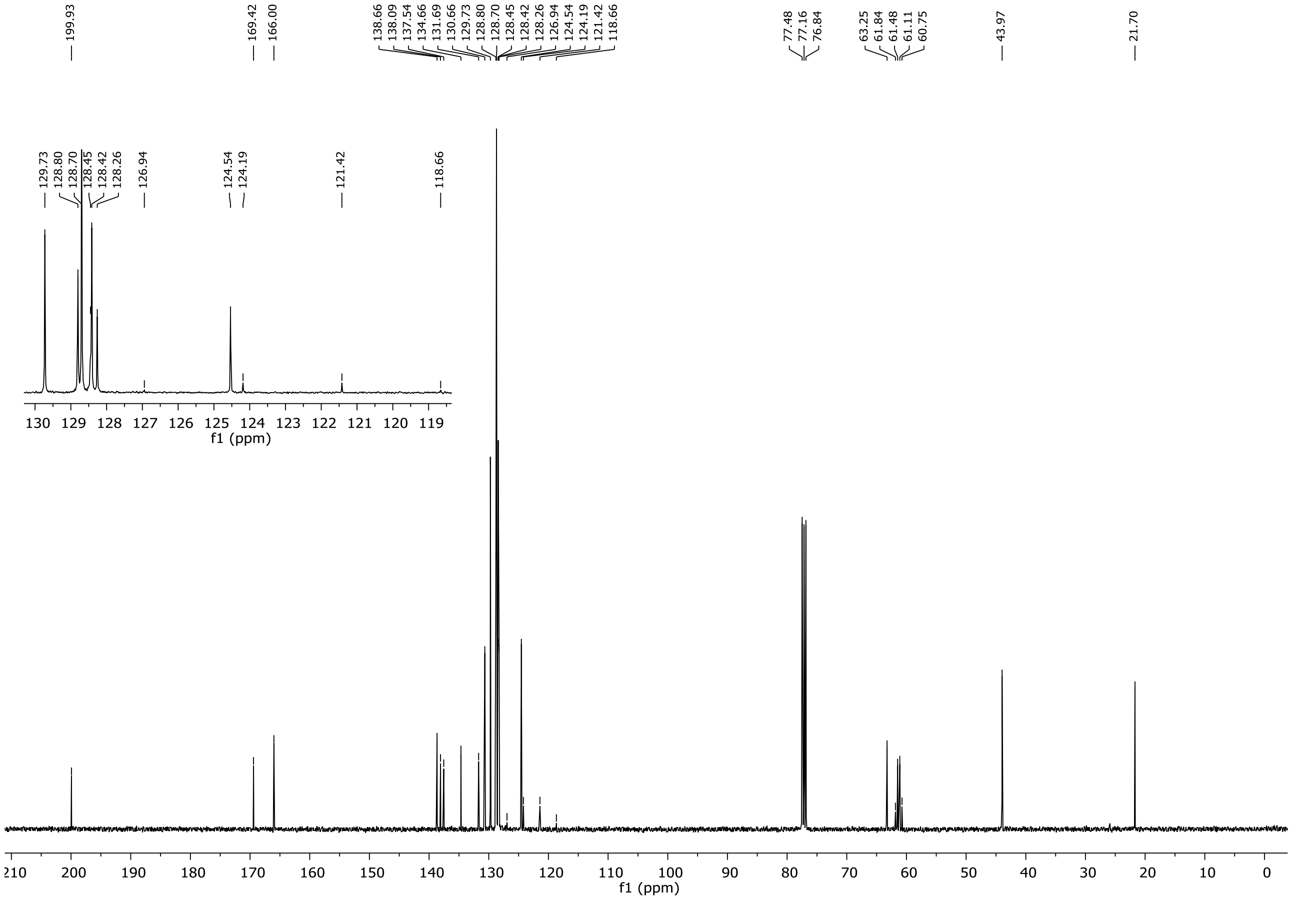
199.93
169.42
166.00
138.66
138.09
137.54
134.66
131.69
130.66
129.73
128.80
128.70
128.45
128.42
128.26
126.94
124.54
124.19
121.42
118.66
77.48
77.16
76.84
63.25
61.84
61.48
61.11
60.75
43.97
21.70
129.73
128.80
128.70
128.45
128.42
128.26
126.94
124.54
124.19
121.42
118.66
130 129 128 127 126 125 124 123 122 121 120 119
f1 (ppm)
210 200 190 180 170 160 150 140 130 120 110 100 90 80 70 60 50 40 30 20 10 0
f1 (ppm)

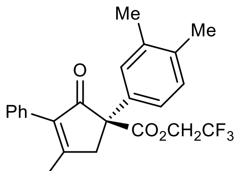
Me
Me
O
Ph
CO2CH2CF3
Ph

3ea

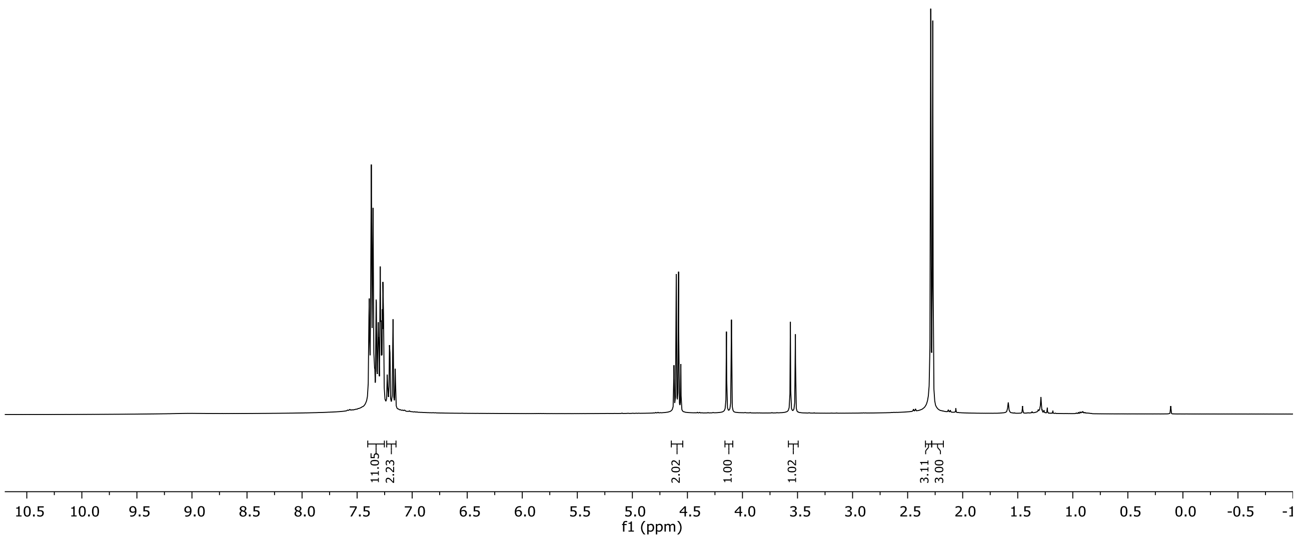
11.05
2.23
2.02
1.00
1.02
3.11
3.00
f1 (ppm)

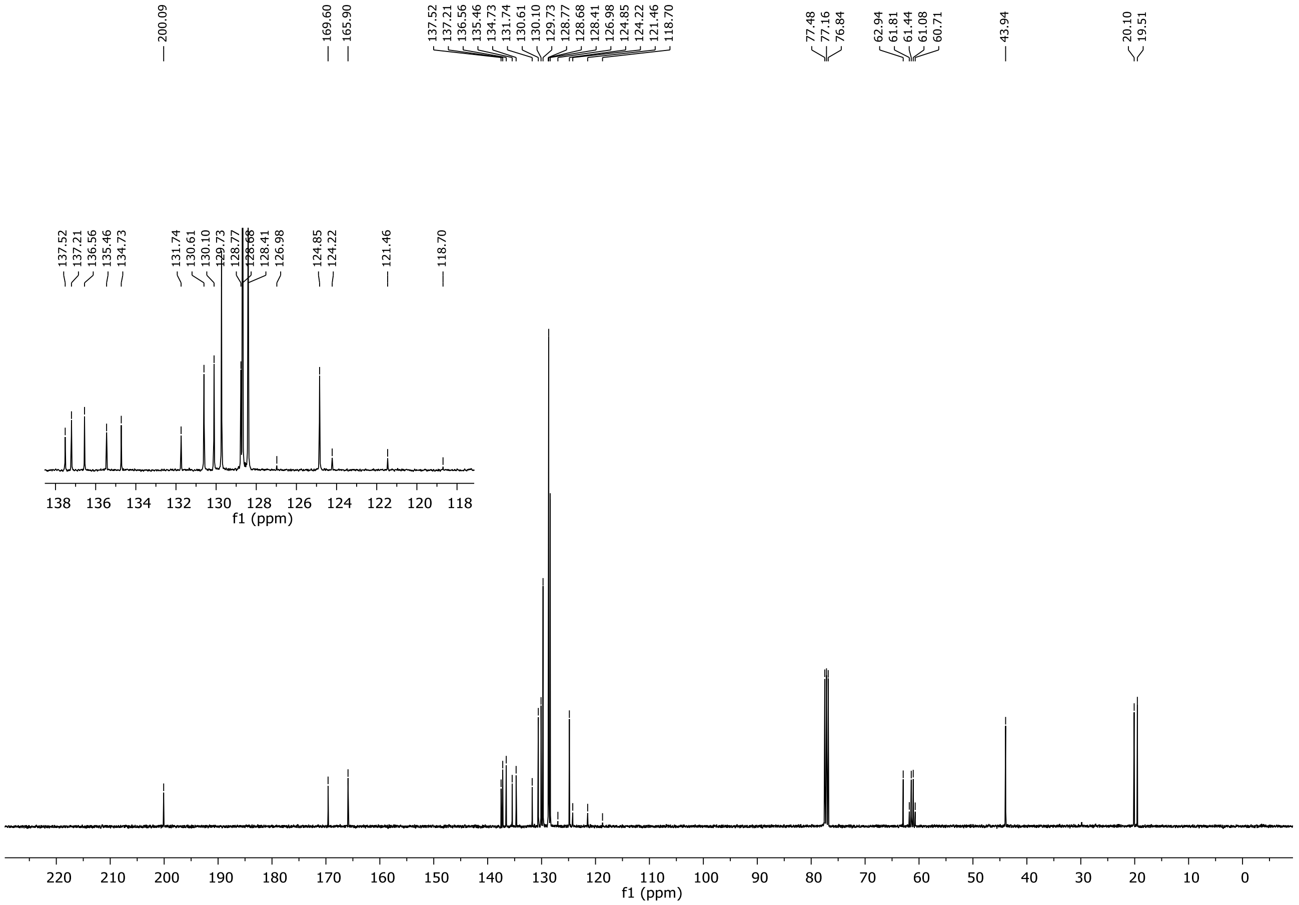
200.09
169.60
165.90
137.52
137.21
136.56
135.46
134.73
131.74
130.61
130.10
129.73
128.77
128.68
128.41
126.98
124.85
124.22
121.46
118.70
77.48
77.16
76.84
62.94
61.81
61.44
61.08
60.71
43.94
20.10
19.51
f1 (ppm)

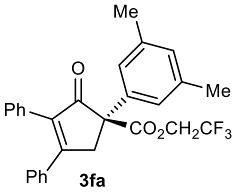
Me
O
Me
Ph
CO2CH2CF3
Ph
3fa

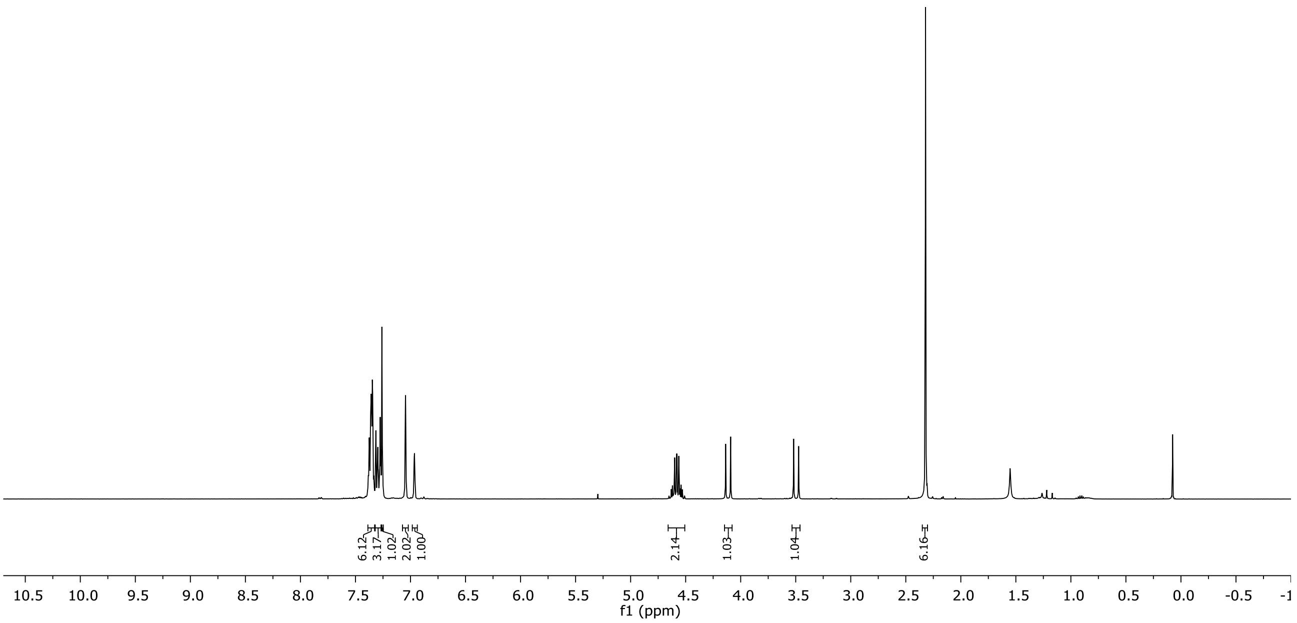

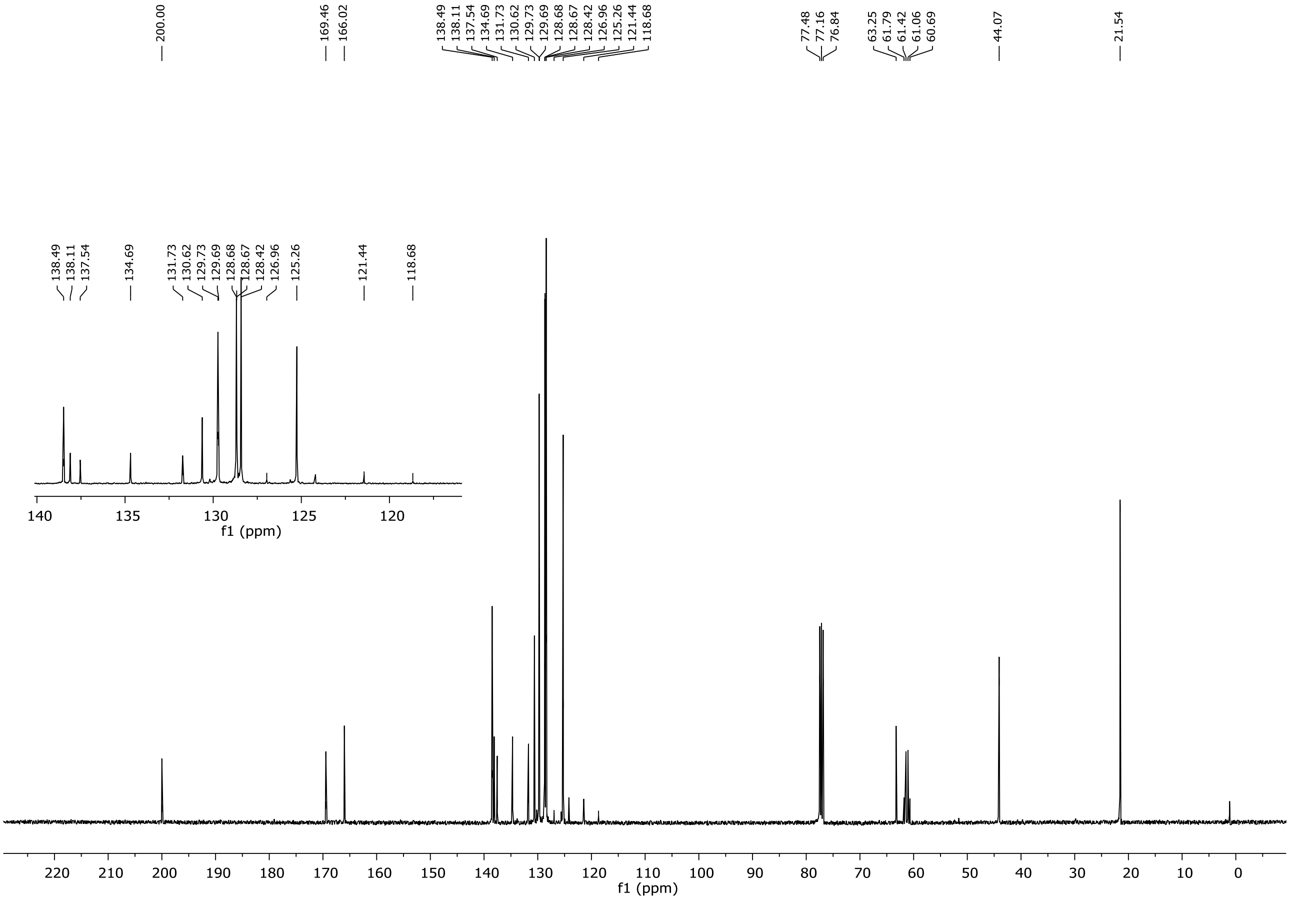

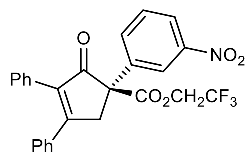
O
NO2
Ph
CO2CH2CF3
Ph

3ga

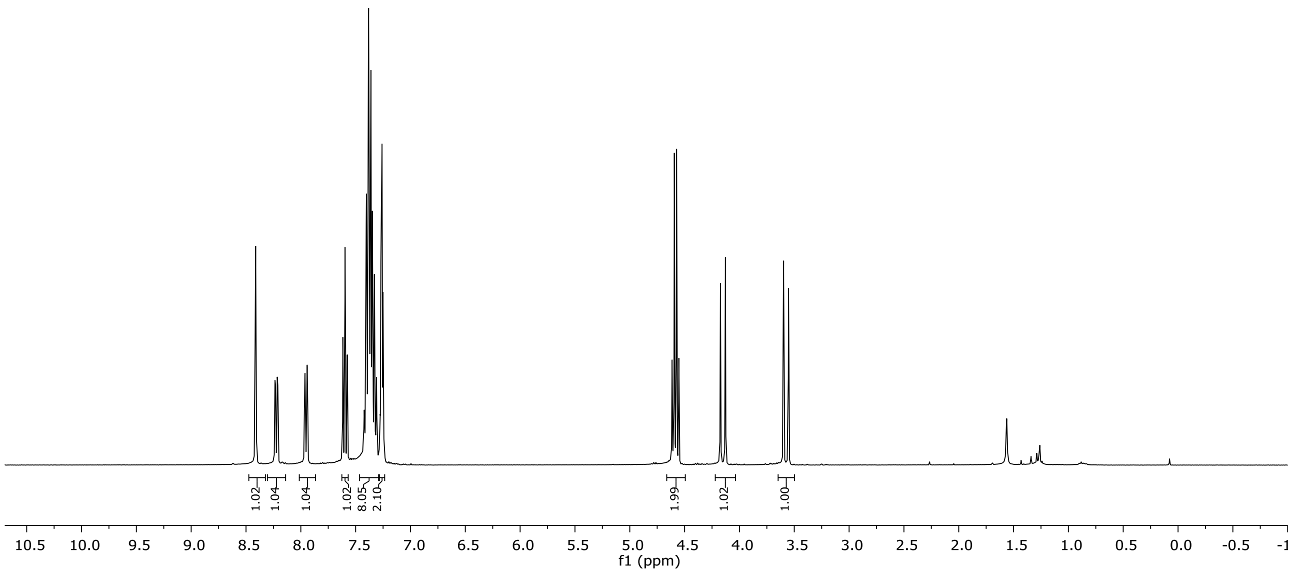
1.02
1.04
1.04
1.02
8.05
2.10
1.99
1.02
1.00
10.5 10.0 9.5 9.0 8.5 8.0 7.5 7.0 6.5 6.0 5.5 5.0 4.5 4.0 3.5 3.0 2.5 2.0 1.5 1.0 0.5 0.0 -0.5 -1
f1 (ppm)

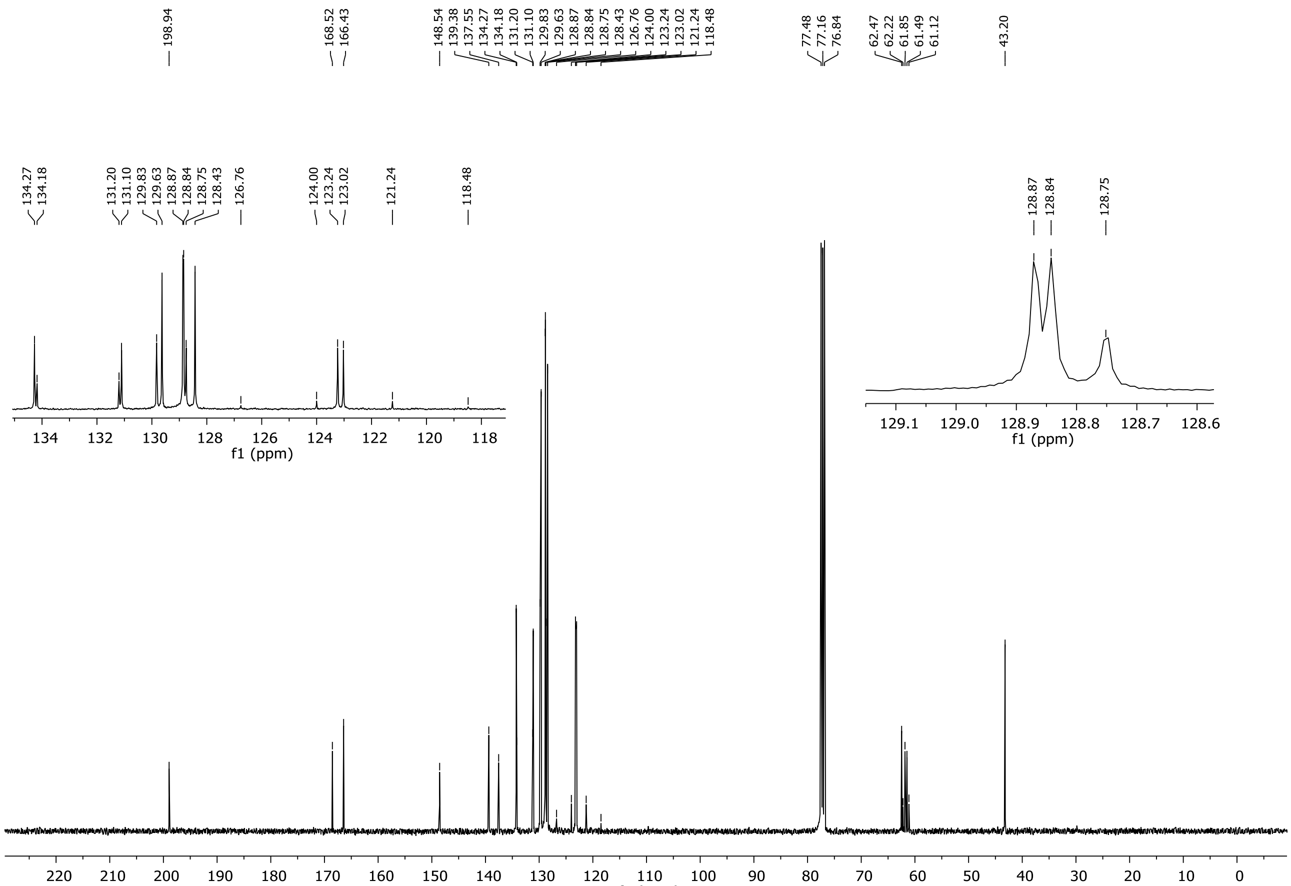
198.94
168.52
166.43
148.54
139.38
137.55
134.27
134.18
131.20
131.10
129.83
129.63
128.87
128.84
128.75
128.43
126.76
124.00
123.24
123.02
121.24
118.48
77.48
77.16
76.84
62.47
62.22
61.85
61.49
61.12
43.20
134 132 130 128 126 124 122 120 118
f1 (ppm)
129.1 129.0 128.9 128.8 128.7 128.6
f1 (ppm)
220 210 200 190 180 170 160 150 140 130 120 110 100 90 80 70 60 50 40 30 20 10 0
f1 (ppm)

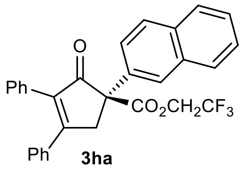
O
Ph
Ph
$CO_2CH_2CF_3$

3ha

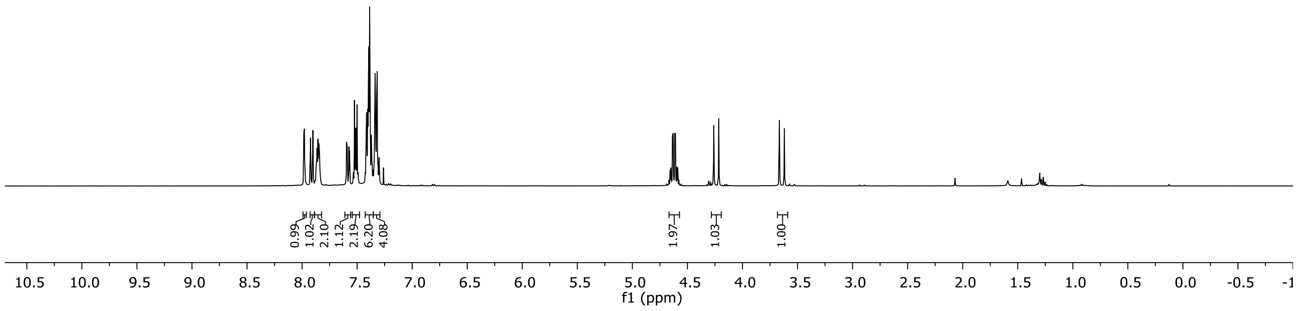
0.99
1.02
2.10
1.12
2.19
6.20
4.08
1.97
1.03
1.00
f1 (ppm)

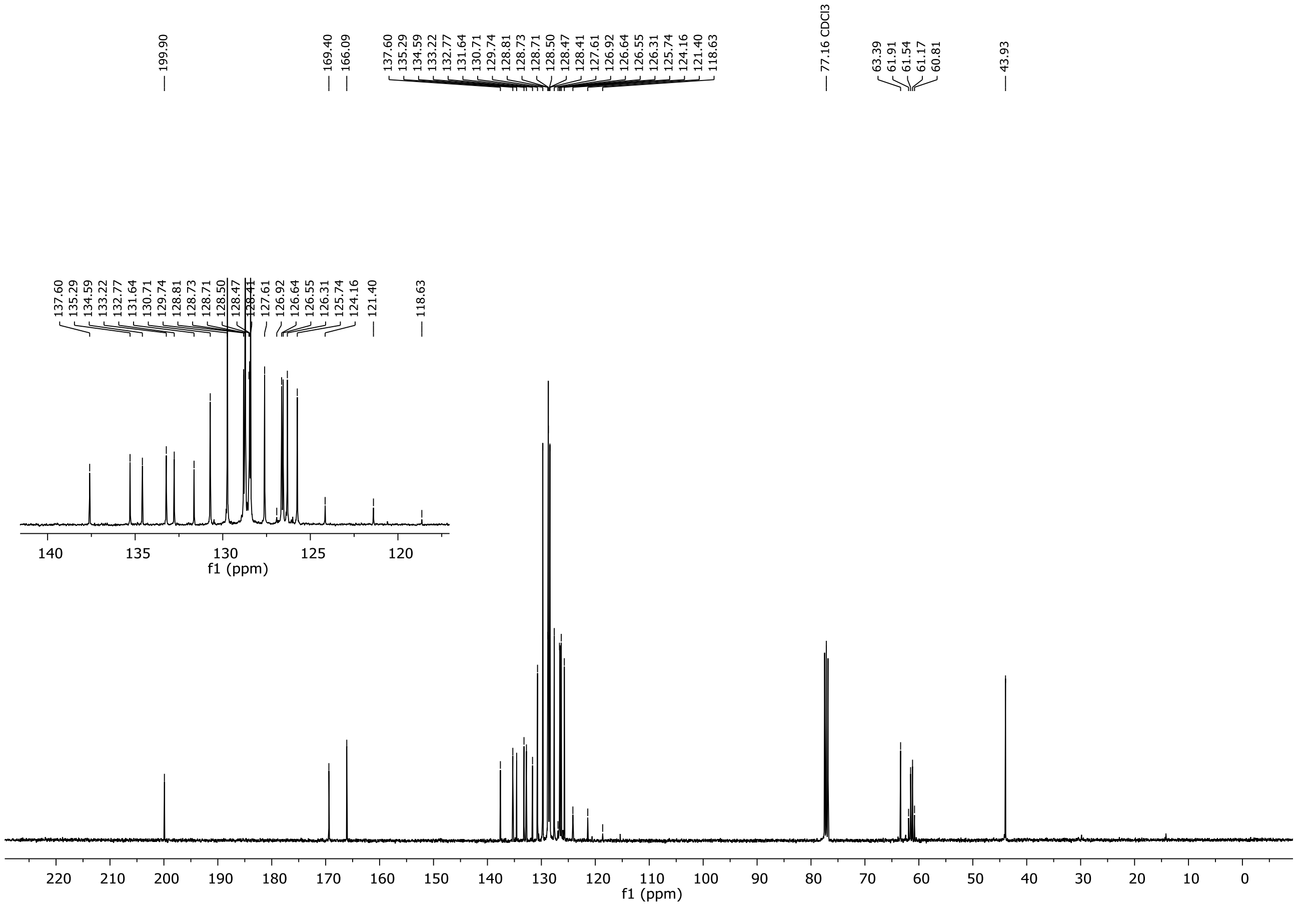
199.90
169.40
166.09
137.60
135.29
134.59
133.22
132.77
131.64
130.71
129.74
128.81
128.73
128.71
128.50
128.47
128.41
127.61
126.92
126.64
126.55
126.31
125.74
124.16
121.40
118.63
77.16 CDCl3
63.39
61.91
61.54
61.17
60.81
43.93
f1 (ppm)

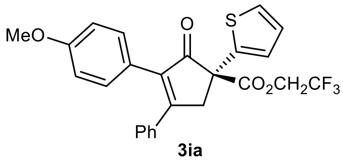
MeO
O
S
$CO_2CH_2CF_3$
Ph

3ia

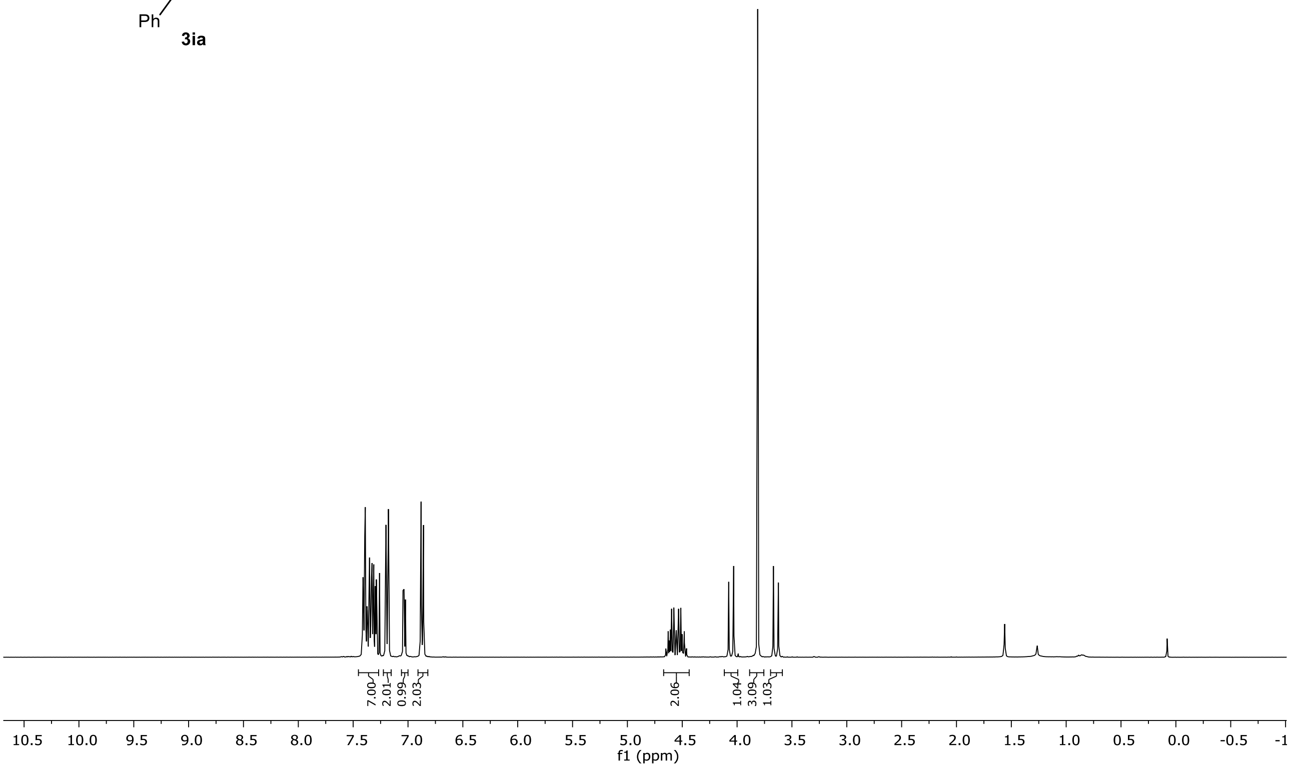
7.00
2.01
0.99
2.03
2.06
1.04
3.09
1.03
10.5 10.0 9.5 9.0 8.5 8.0 7.5 7.0 6.5 6.0 5.5 5.0 4.5 4.0 3.5 3.0 2.5 2.0 1.5 1.0 0.5 0.0 -0.5 -1
f1 (ppm)

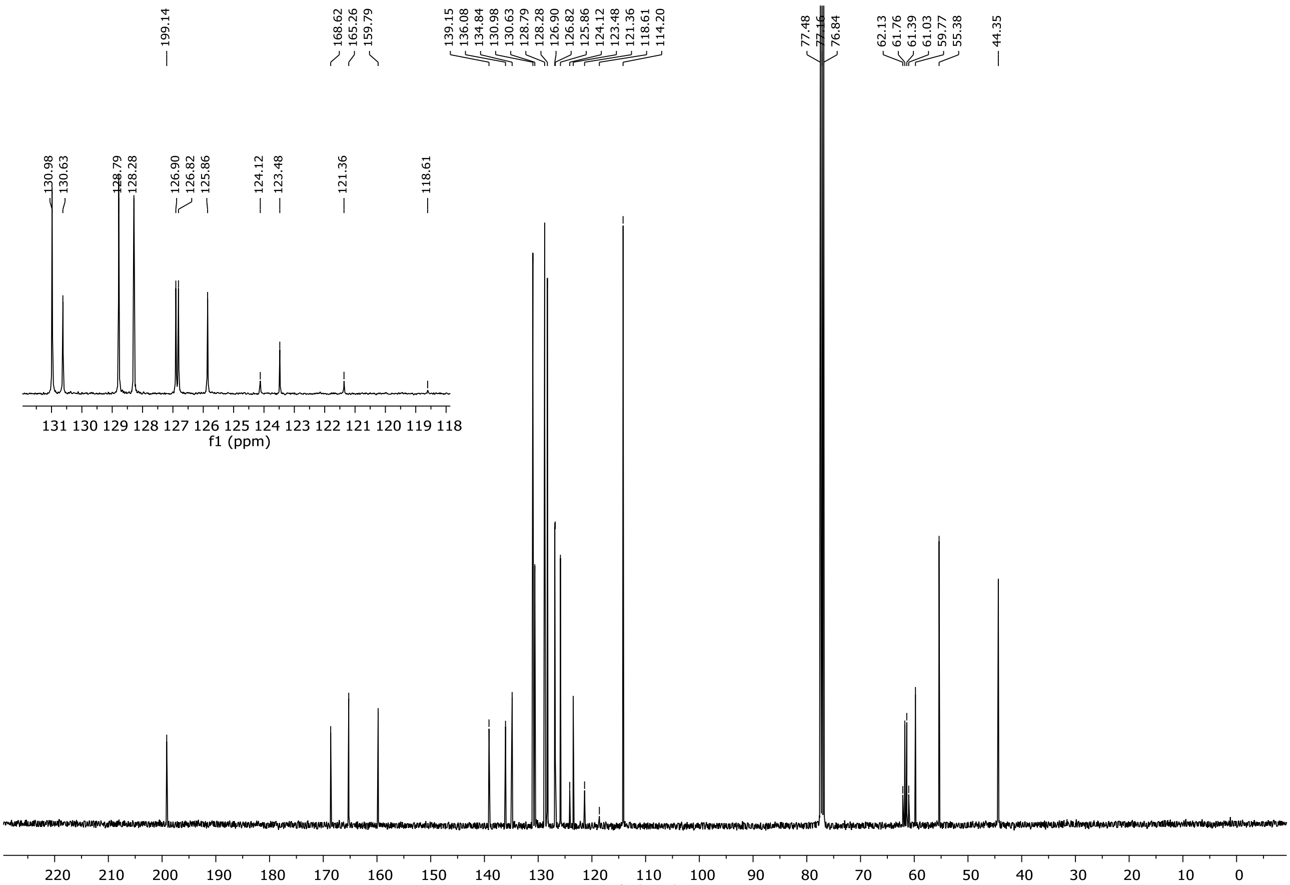
199.14
168.62
165.26
159.79
139.15
136.08
134.84
130.98
130.63
128.79
128.28
126.90
126.82
125.86
124.12
123.48
121.36
118.61
114.20
77.48
76.84
62.13
61.76
61.39
61.03
59.77
55.38
44.35
131 130 129 128 127 126 125 124 123 122 121 120 119 118
f1 (ppm)
220 210 200 190 180 170 160 150 140 130 120 110 100 90 80 70 60 50 40 30 20 10 0
f1 (ppm)

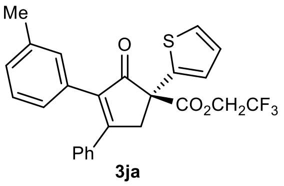
Me
O
S
CO2CH2CF3
Ph

3ja

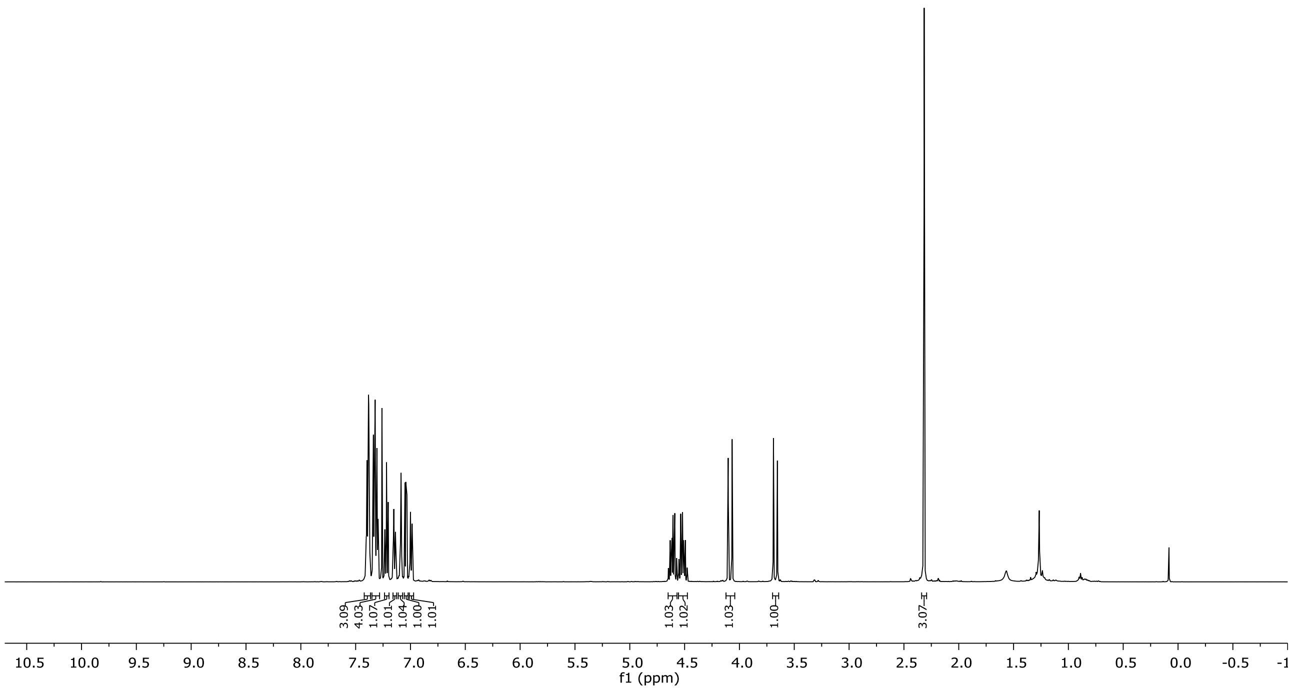
3.09
4.03
1.07
1.01
1.04
1.00
1.01
1.03
1.02
1.03
1.00
3.07
10.5 10.0 9.5 9.0 8.5 8.0 7.5 7.0 6.5 6.0 5.5 5.0 4.5 4.0 3.5 3.0 2.5 2.0 1.5 1.0 0.5 0.0 -0.5 -1
f1 (ppm)

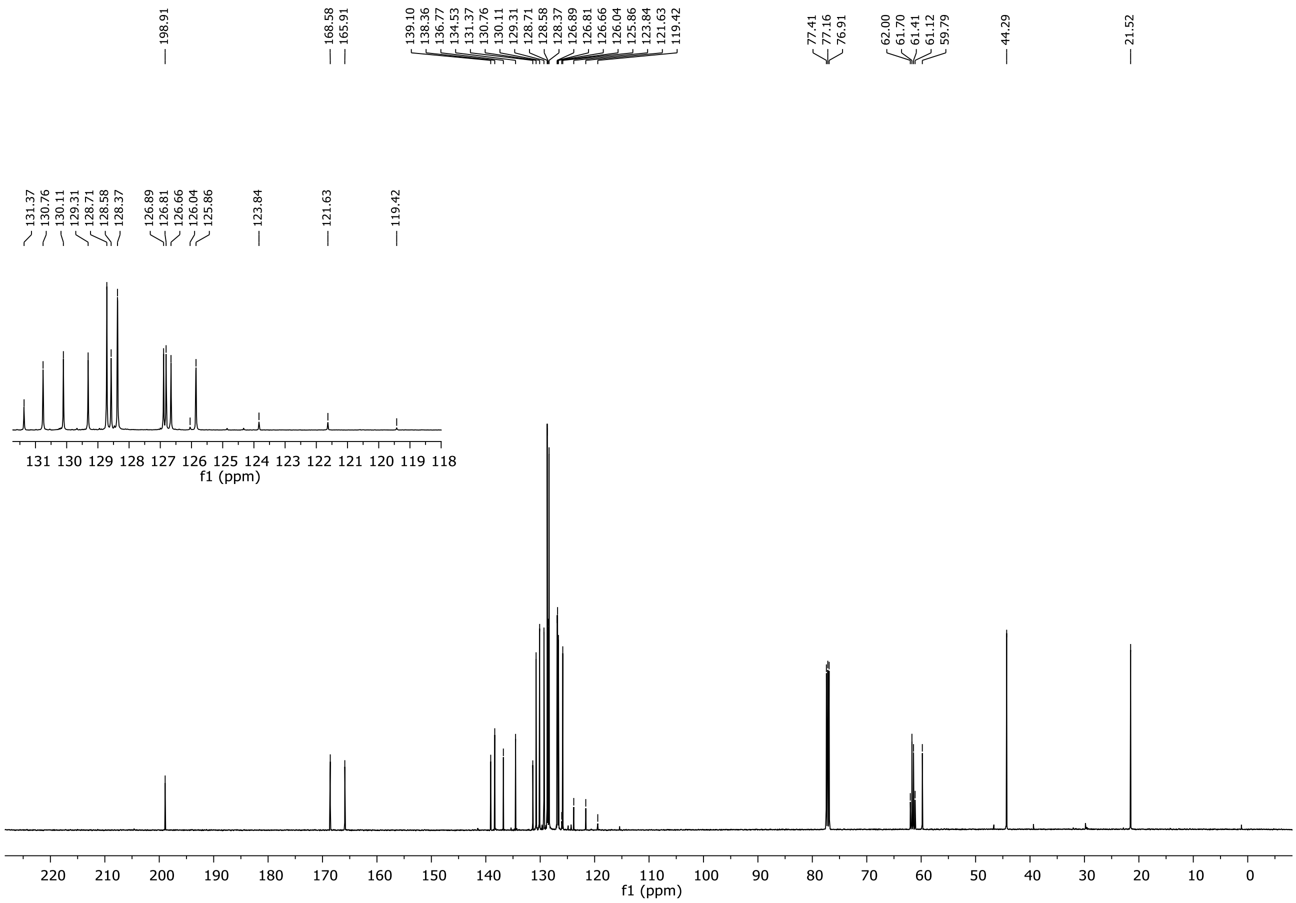
198.91
168.58
165.91
139.10
138.36
136.77
134.53
131.37
130.76
130.11
129.31
128.71
128.58
128.37
126.89
126.81
126.66
126.04
125.86
123.84
121.63
119.42
77.41
77.16
76.91
62.00
61.70
61.41
61.12
59.79
44.29
21.52
131.37
130.76
130.11
129.31
128.71
128.58
128.37
126.89
126.81
126.66
126.04
125.86
123.84
121.63
119.42
131 130 129 128 127 126 125 124 123 122 121 120 119 118
f1 (ppm)
220 210 200 190 180 170 160 150 140 130 120 110 100 90 80 70 60 50 40 30 20 10 0
f1 (ppm)

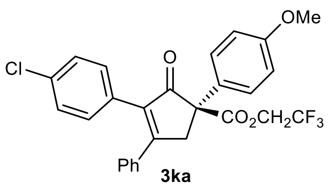
OMe
Cl
O
CO2CH2CF3
Ph

3ka

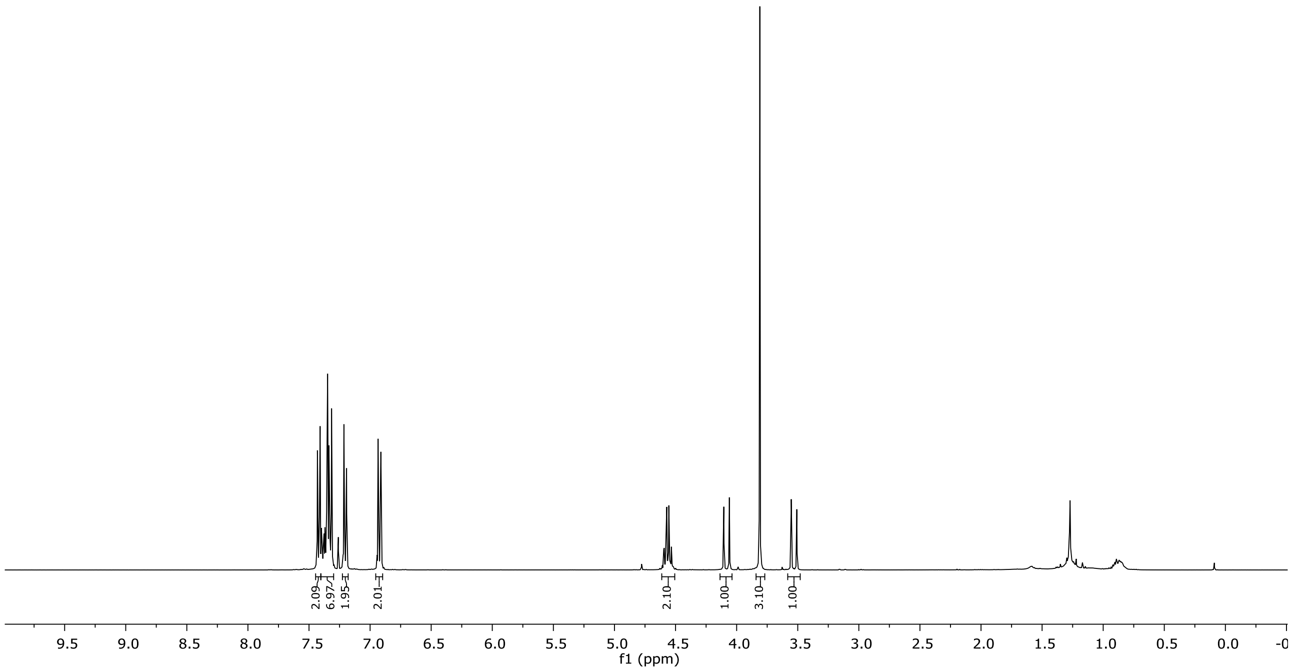
2.09
6.97
1.95
2.01
2.10
1.00
3.10
1.00
f1 (ppm)

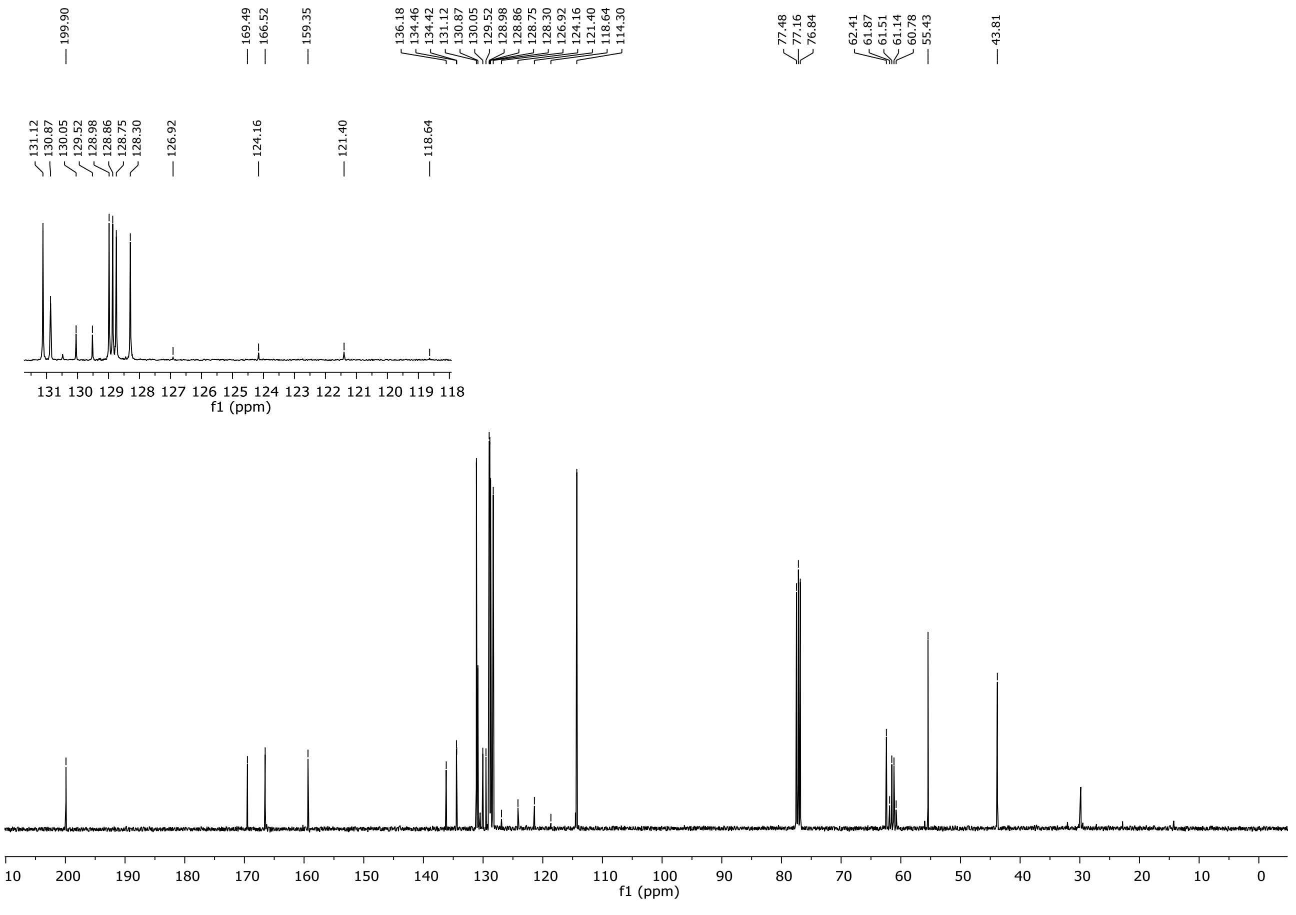
199.90
169.49
166.52
159.35
136.18
134.46
134.42
131.12
130.87
130.05
129.52
128.98
128.86
128.75
128.30
126.92
124.16
121.40
118.64
114.30
77.48
77.16
76.84
62.41
61.87
61.51
61.14
60.78
55.43
43.81
f1 (ppm)

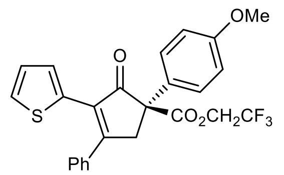
OMe
O
S
CO2CH2CF3
Ph

3la

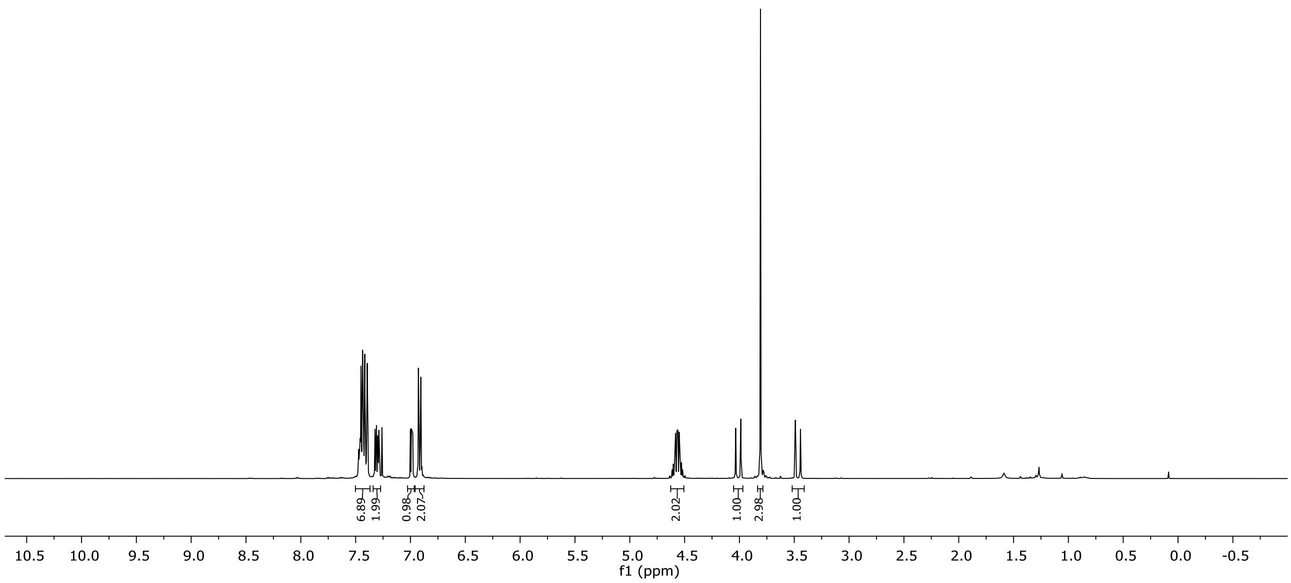
6.89
1.99
0.98
2.07
2.02
1.00
2.98
1.00
10.5 10.0 9.5 9.0 8.5 8.0 7.5 7.0 6.5 6.0 5.5 5.0 4.5 4.0 3.5 3.0 2.5 2.0 1.5 1.0 0.5 0.0 -0.5
f1 (ppm)

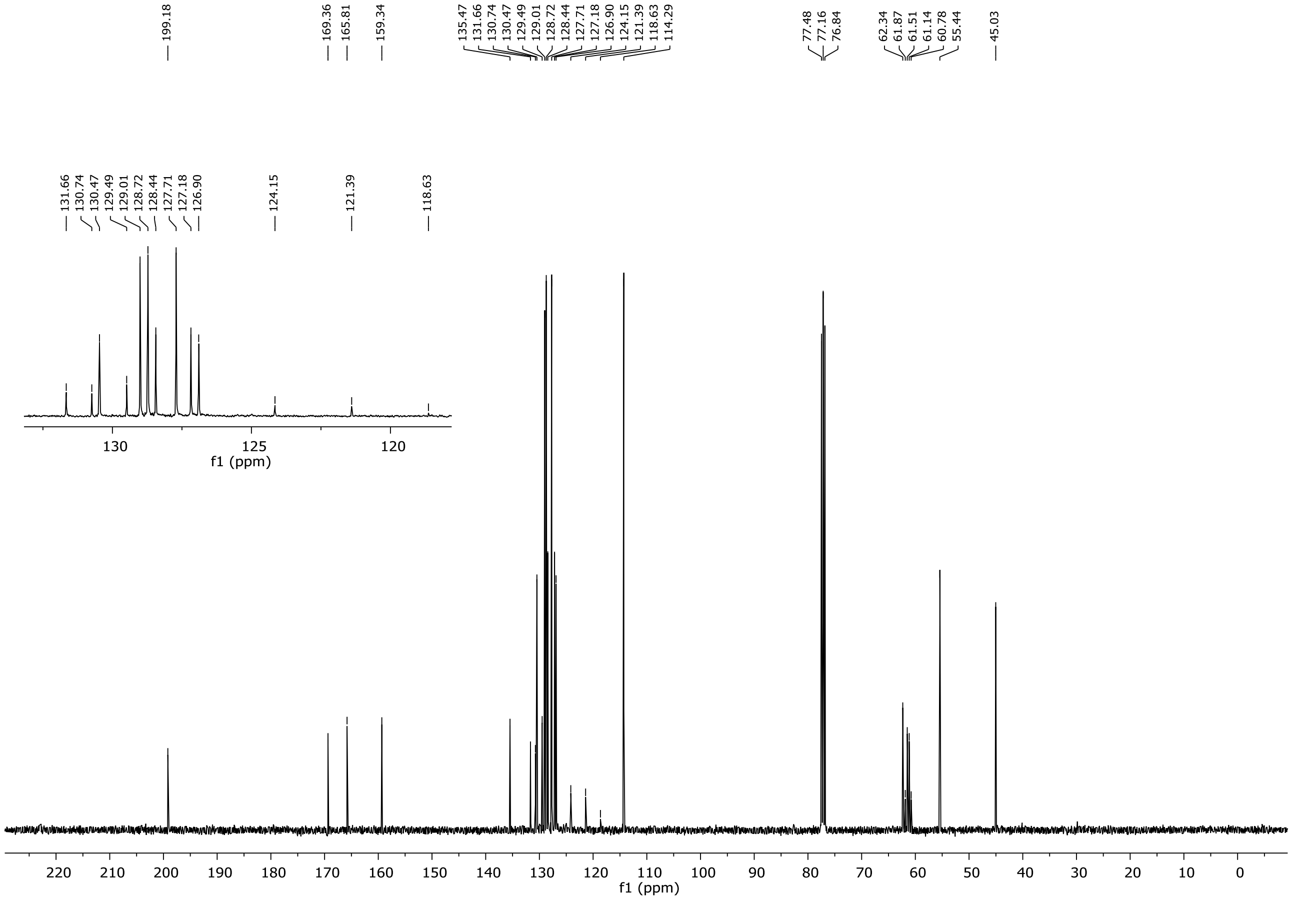
199.18
169.36
165.81
159.34
135.47
131.66
130.74
130.47
129.49
129.01
128.72
128.44
127.71
127.18
126.90
124.15
121.39
118.63
114.29
77.48
77.16
76.84
62.34
61.87
61.51
61.14
60.78
55.44
45.03
131.66
130.74
130.47
129.49
129.01
128.72
128.44
127.71
127.18
126.90
124.15
121.39
118.63
130 125 120
f1 (ppm)
220 210 200 190 180 170 160 150 140 130 120 110 100 90 80 70 60 50 40 30 20 10 0
f1 (ppm)

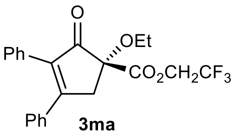
O
Ph
OEt
$CO_2CH_2CF_3$
Ph

3ma

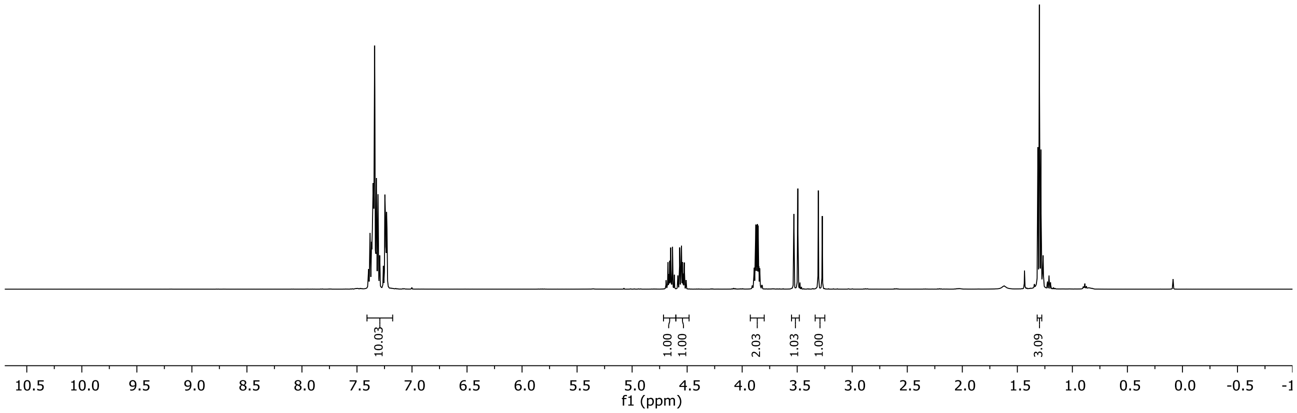
10.03
1.00
1.00
2.03
1.03
1.00
3.09
10.5 10.0 9.5 9.0 8.5 8.0 7.5 7.0 6.5 6.0 5.5 5.0 4.5 4.0 3.5 3.0 2.5 2.0 1.5 1.0 0.5 0.0 -0.5 -1
f1 (ppm)

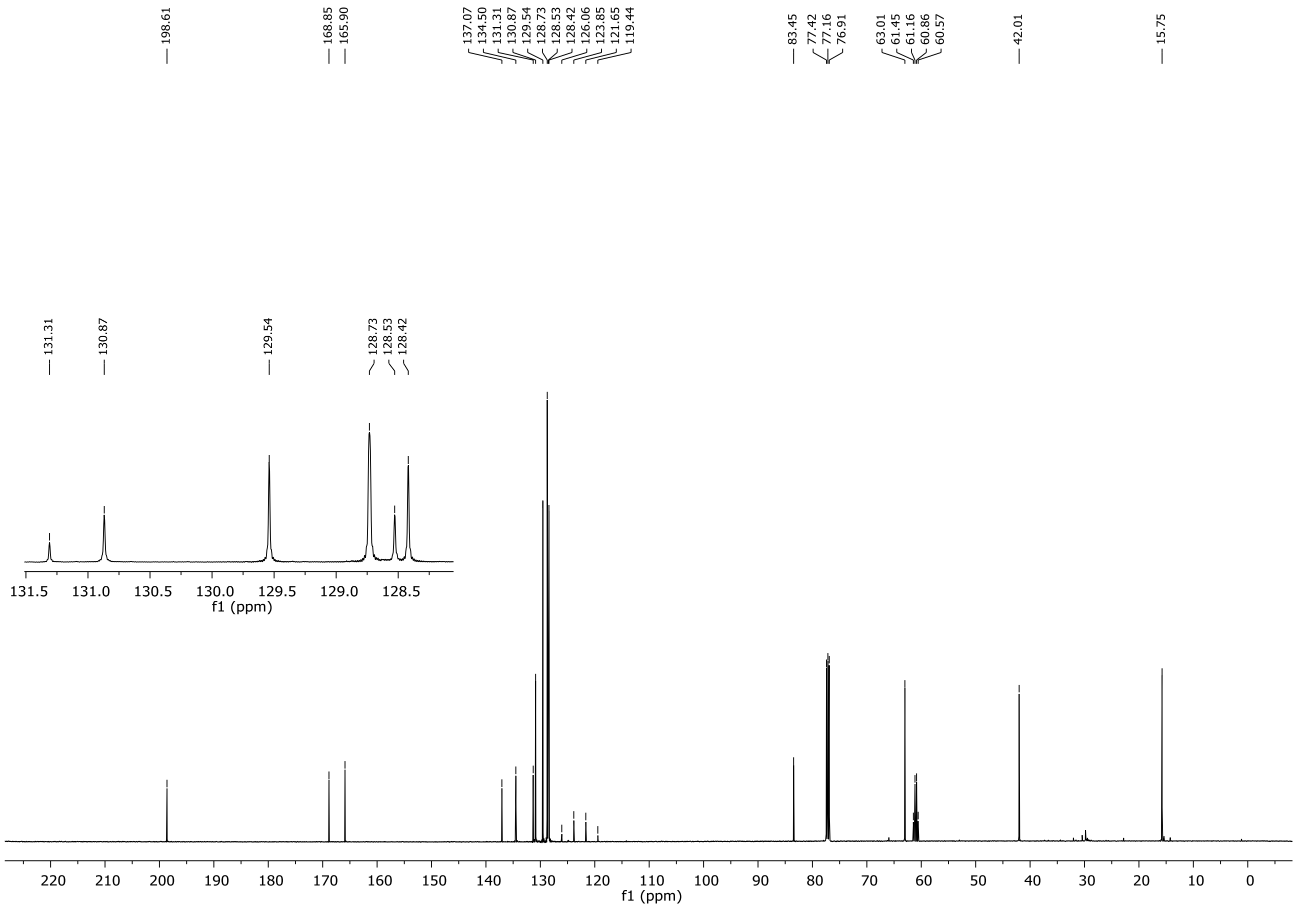
198.61
168.85
165.90
137.07
134.50
131.31
130.87
129.54
128.73
128.53
128.42
126.06
123.85
121.65
119.44
83.45
77.42
77.16
76.91
63.01
61.45
61.16
60.86
60.57
42.01
15.75
131.31
130.87
129.54
128.73
128.53
128.42
131.5 131.0 130.5 130.0 129.5 129.0 128.5
f1 (ppm)
220 210 200 190 180 170 160 150 140 130 120 110 100 90 80 70 60 50 40 30 20 10 0
f1 (ppm)

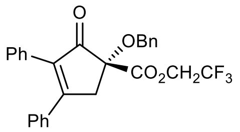

3na

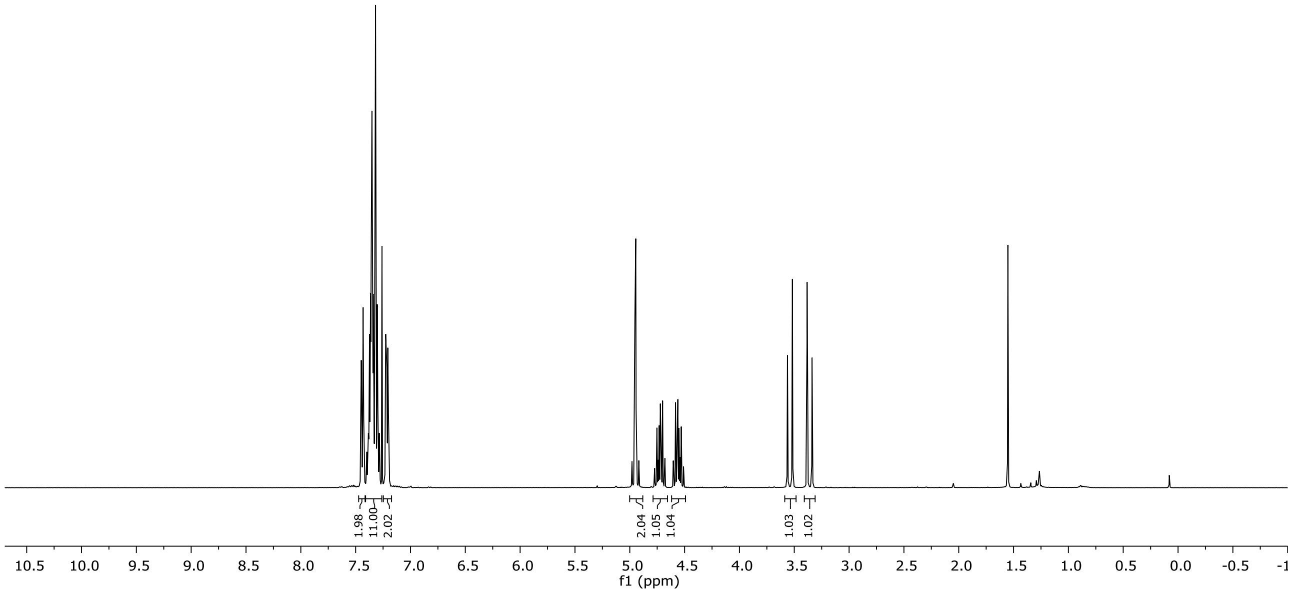

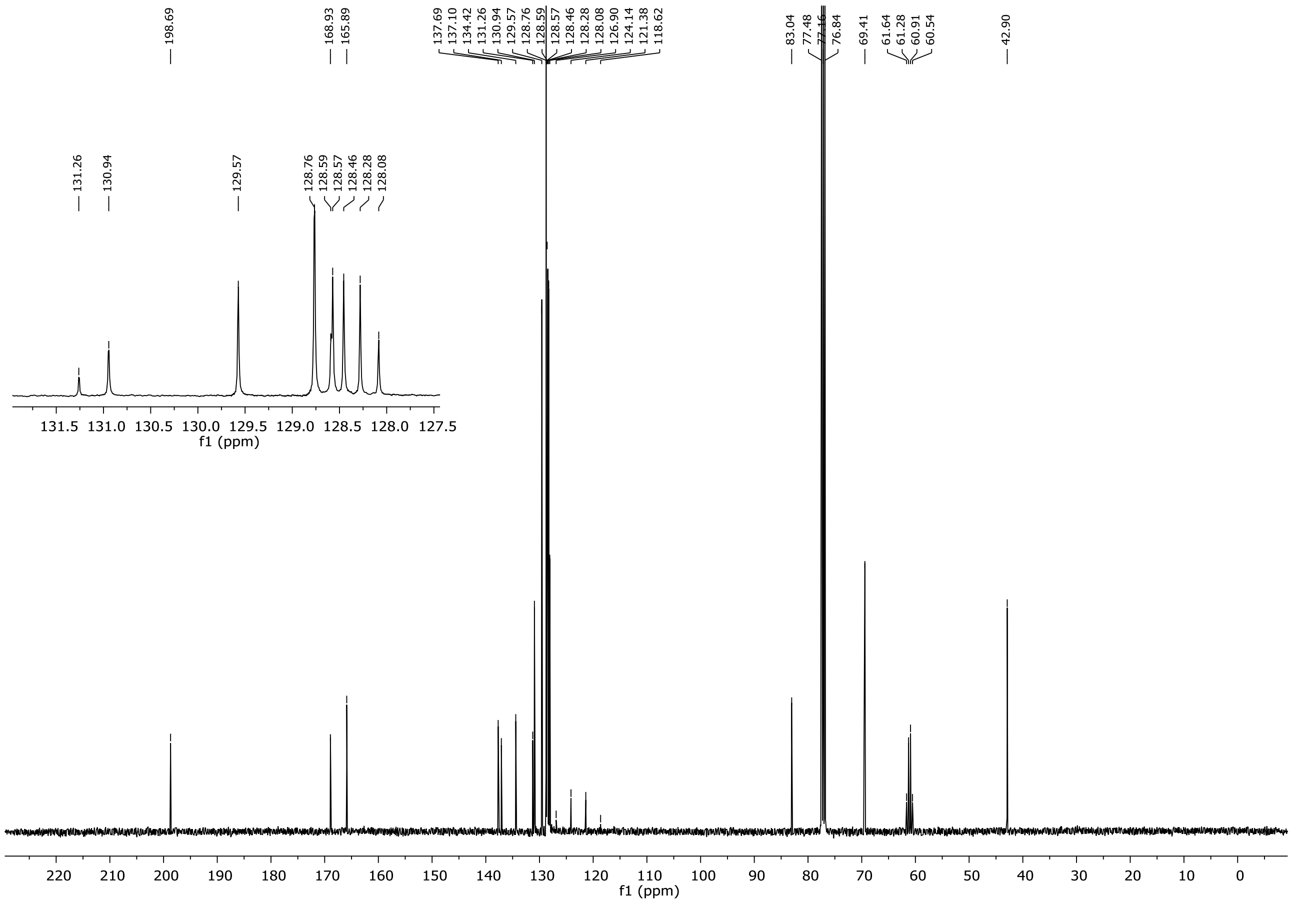

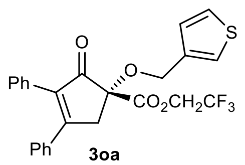
O
Ph
O
S
CO2CH2CF3
Ph

3oa

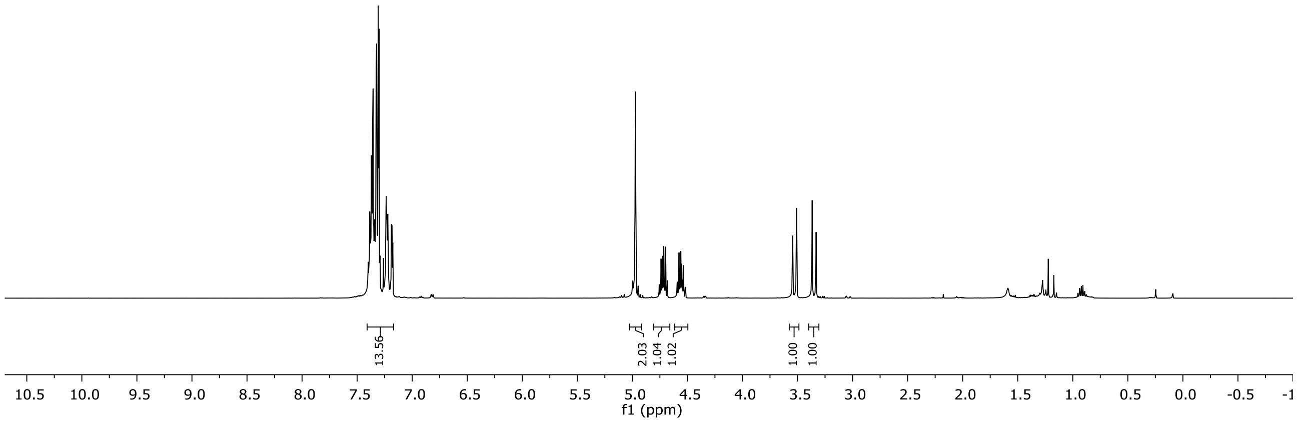
13.56
2.03
1.04
1.02
1.00
1.00
f1 (ppm)

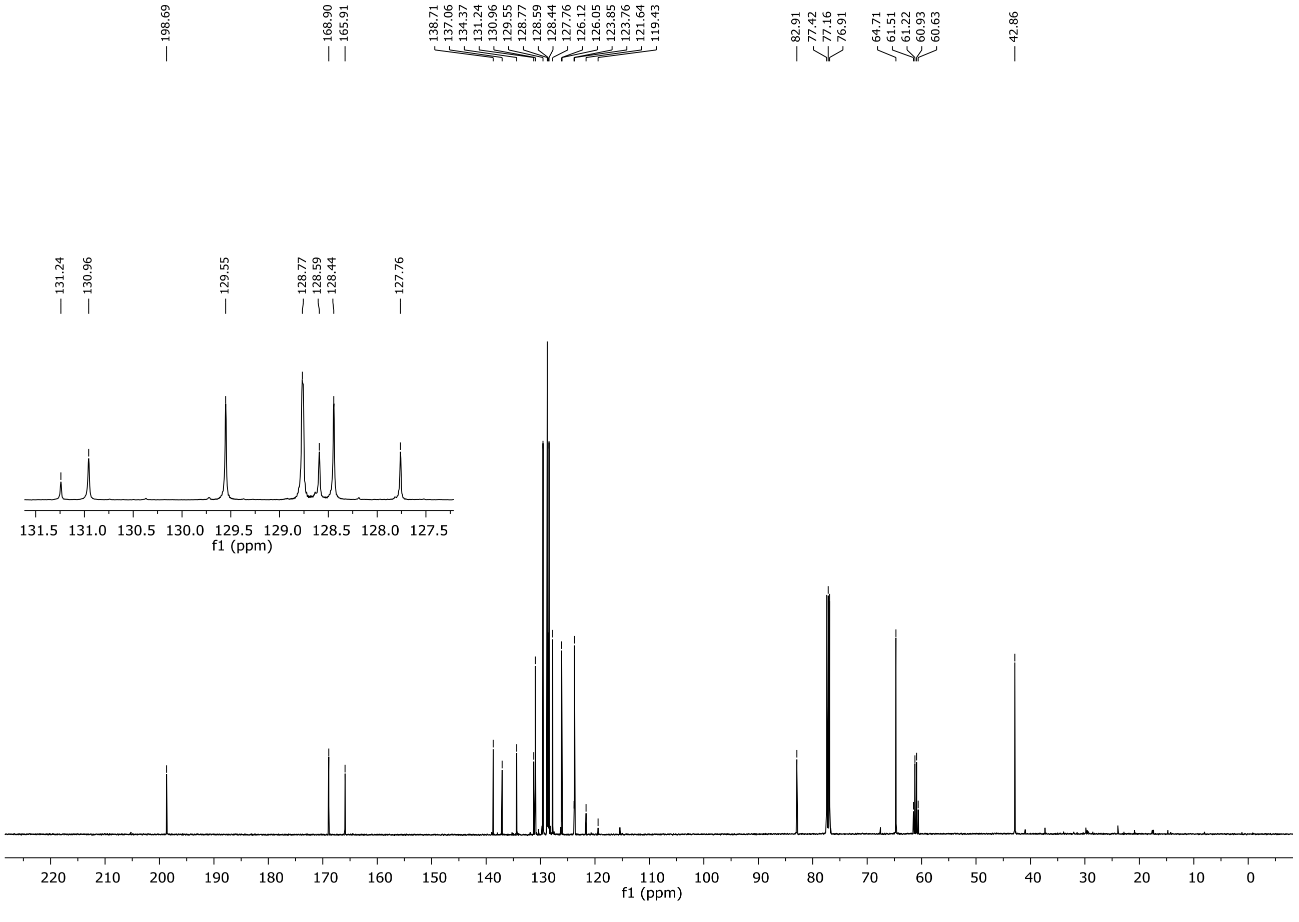
198.69
168.90
165.91
138.71
137.06
134.37
131.24
130.96
129.55
128.77
128.59
128.44
127.76
126.12
126.05
123.85
123.76
121.64
119.43
82.91
77.42
77.16
76.91
64.71
61.51
61.22
60.93
60.63
42.86
131.24
130.96
129.55
128.77
128.59
128.44
127.76
f1 (ppm)
f1 (ppm)

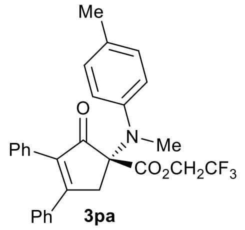
Me
O
N
Me
Ph
CO2CH2CF3
Ph

3pa

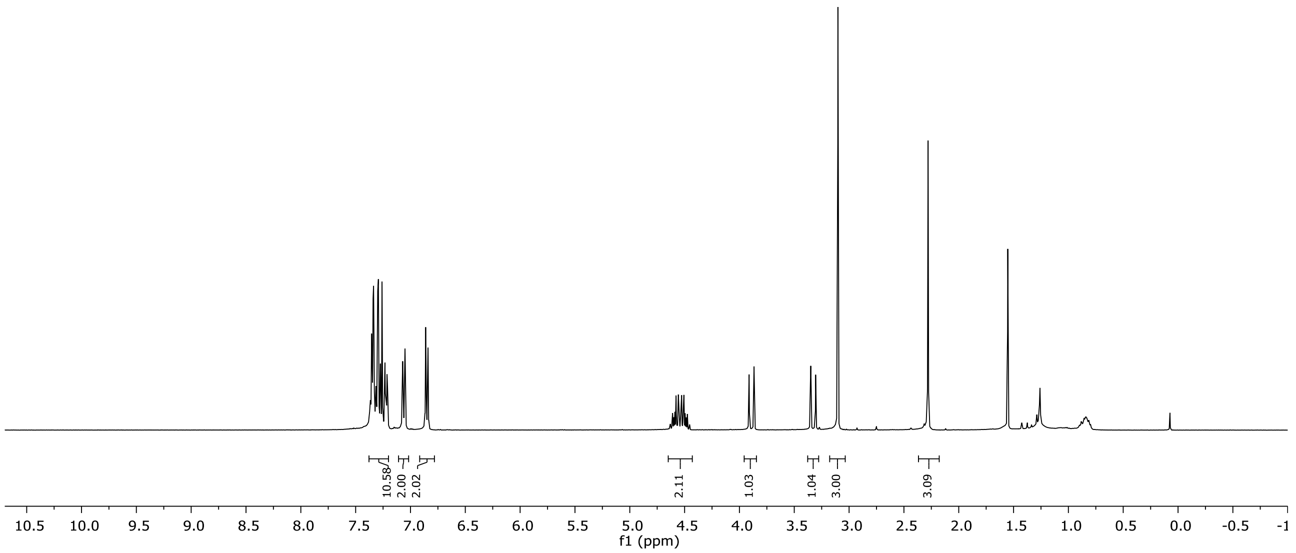
10.58
2.00
2.02
2.11
1.03
1.04
3.00
3.09
f1 (ppm)

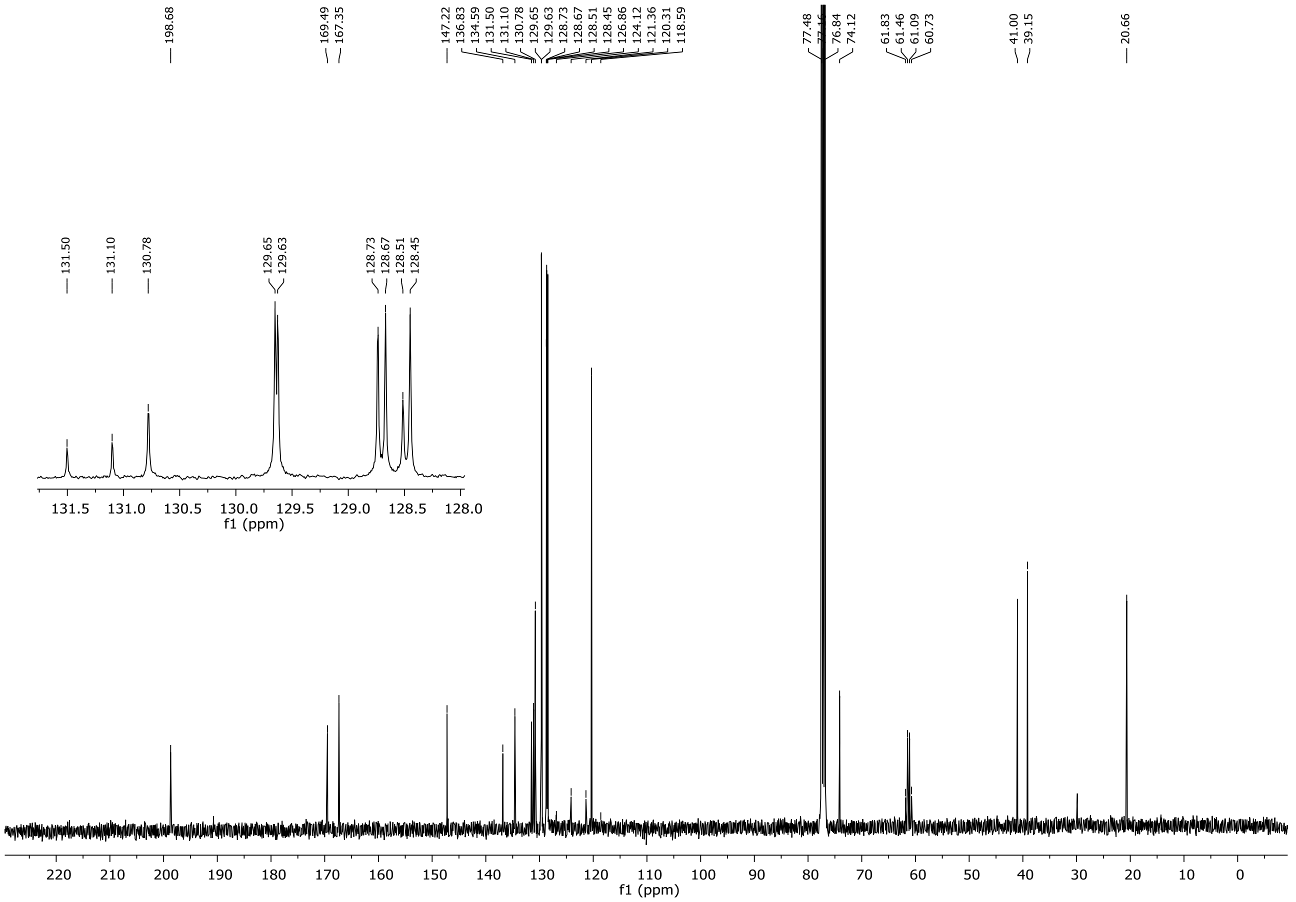
198.68
169.49
167.35
147.22
136.83
134.59
131.50
131.10
130.78
129.65
129.63
128.73
128.67
128.51
128.45
126.86
124.12
121.36
120.31
118.59
77.48
76.84
74.12
61.83
61.46
61.09
60.73
41.00
39.15
20.66
f1 (ppm)

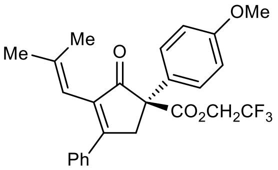
OMe
Me
Me
O
CO2CH2CF3
Ph

7

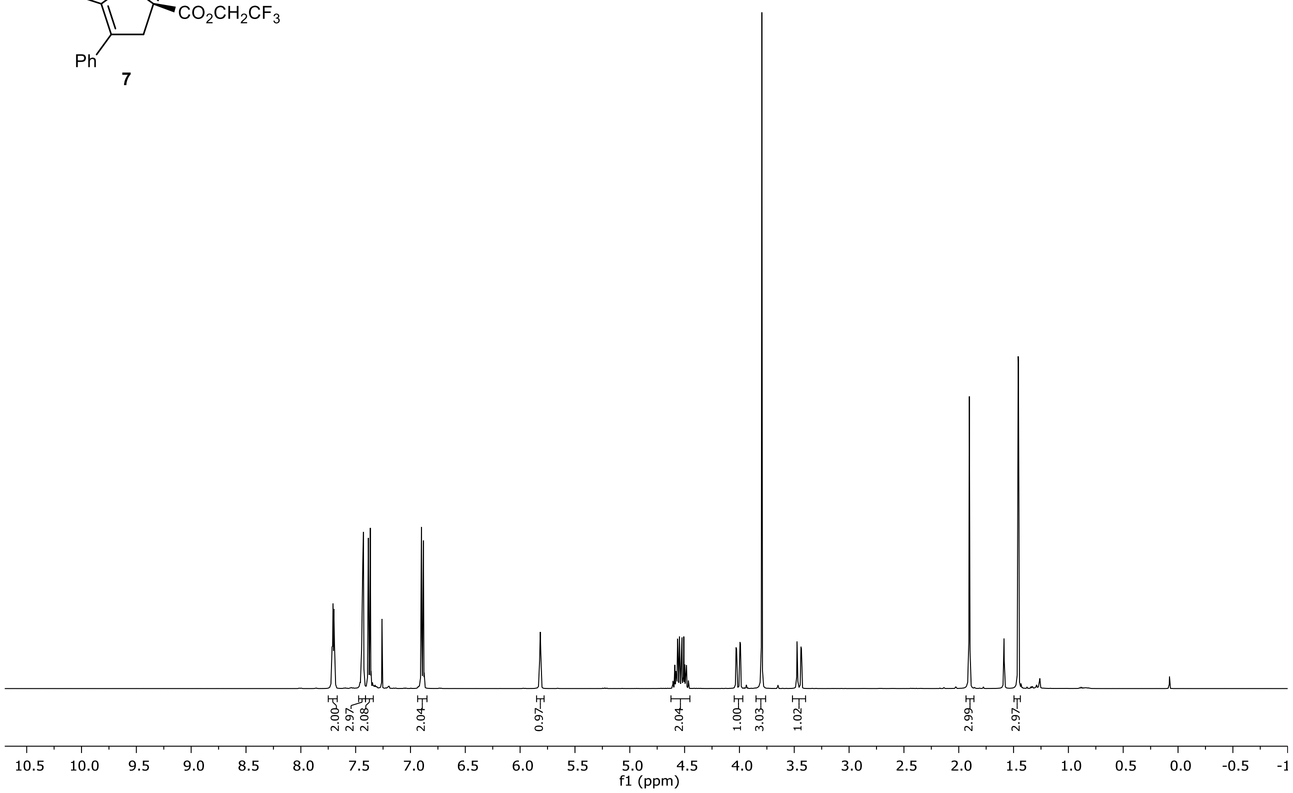
2.00
2.97
2.08
2.04
0.97
2.04
1.00
3.03
1.02
2.99
2.97
f1 (ppm)

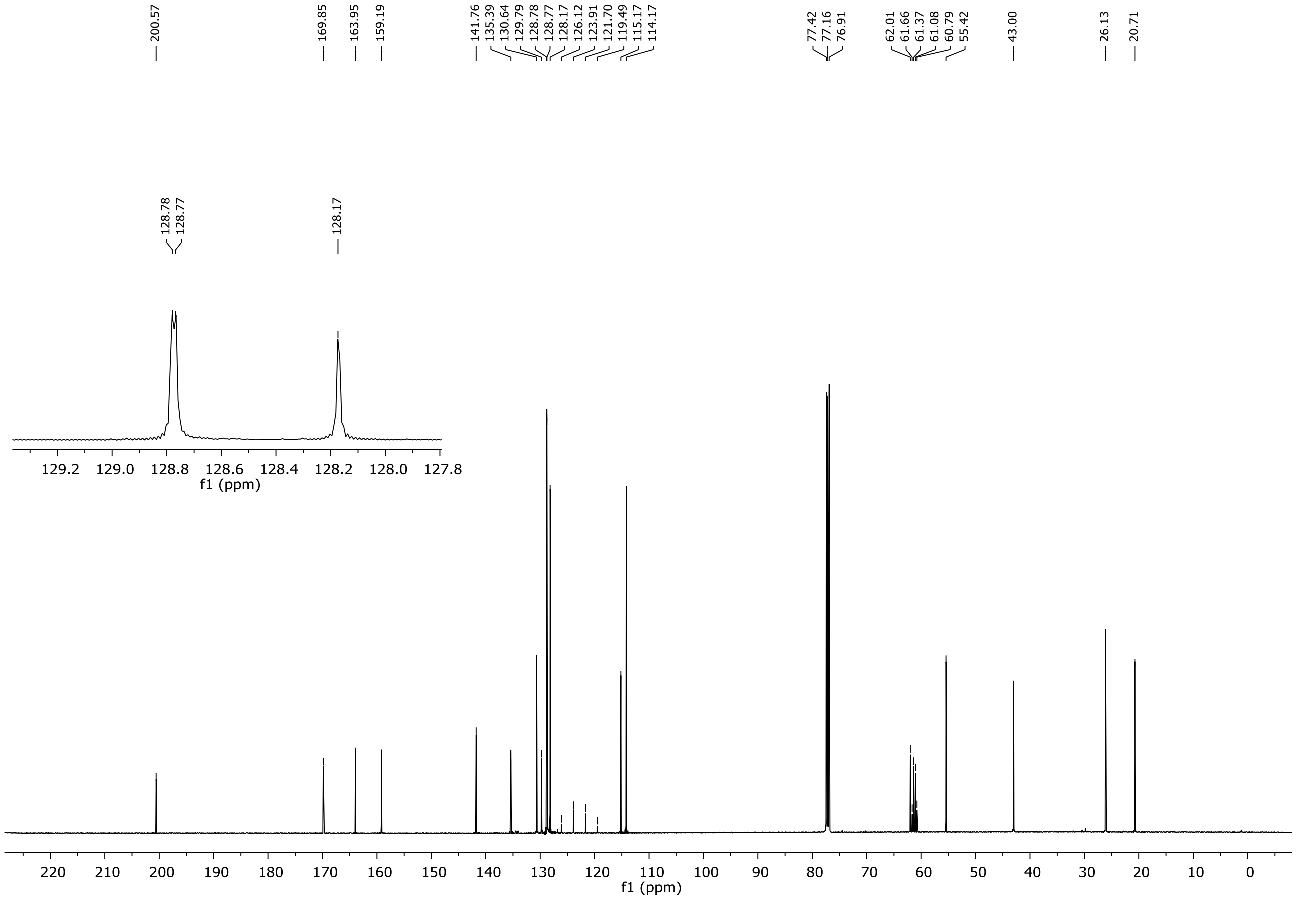
200.57
169.85
163.95
159.19
141.76
135.39
130.64
129.79
128.78
128.77
128.17
126.12
123.91
121.70
119.49
115.17
114.17
77.42
77.16
76.91
62.01
61.66
61.37
61.08
60.79
55.42
43.00
26.13
20.71
128.78
128.77
128.17
f1 (ppm)
f1 (ppm)

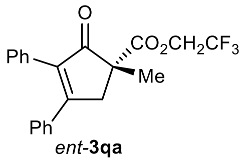
O
Ph
CO2CH2CF3
Me
Ph

ent-**3qa**

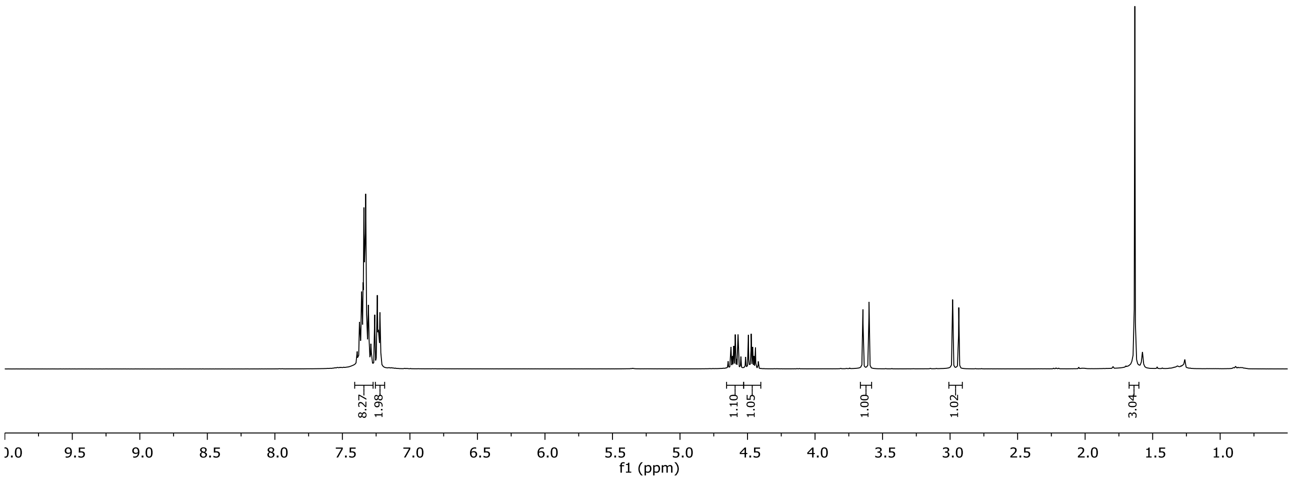
8.27
1.98
1.10
1.05
1.00
1.02
3.04
f1 (ppm)

.

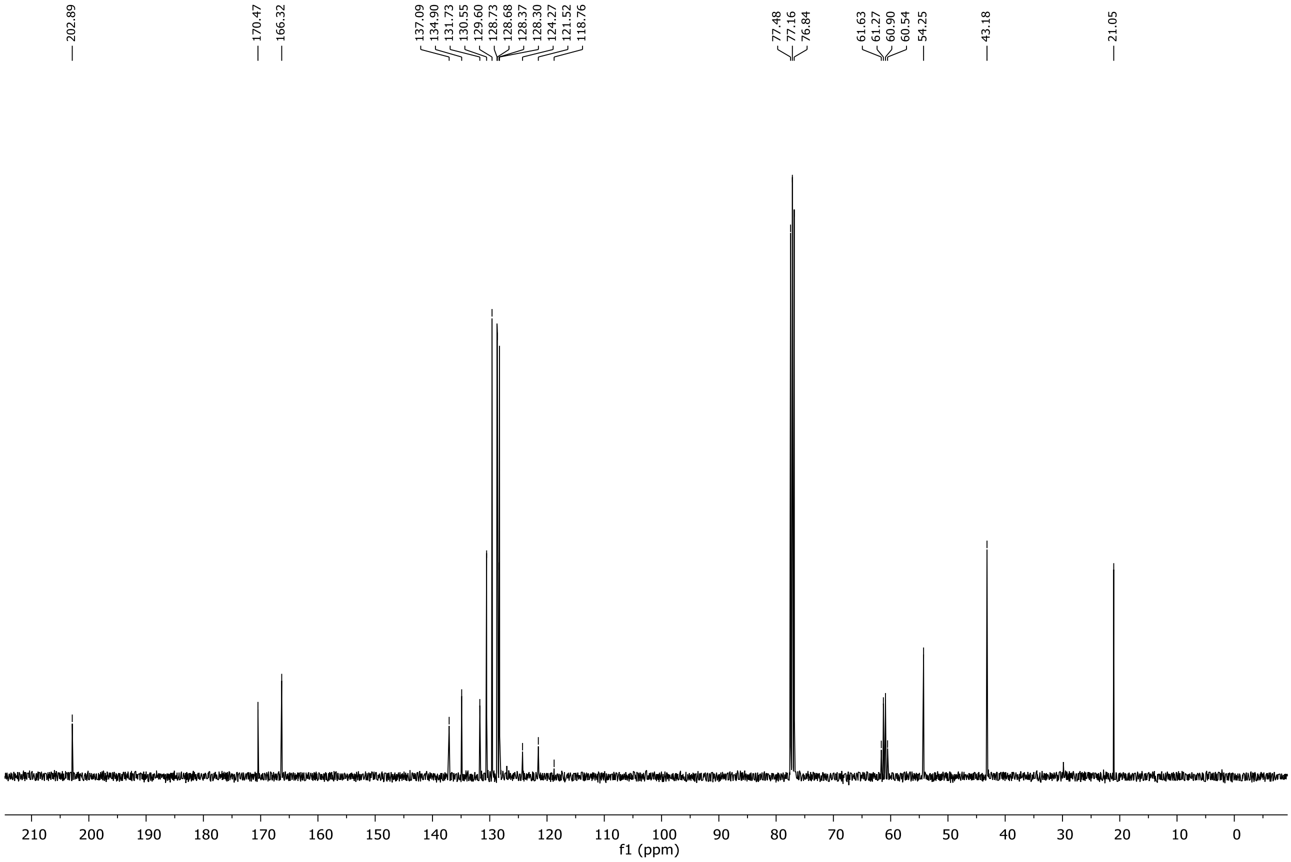
202.89
170.47
166.32
137.09
134.90
131.73
130.55
129.60
128.73
128.68
128.37
128.30
124.27
121.52
118.76
77.48
77.16
76.84
61.63
61.27
60.90
60.54
54.25
43.18
21.05
f1 (ppm)

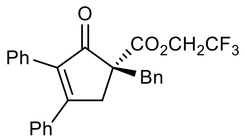
O
Ph
Ph
CO2CH2CF3
Bn

ent-**3ra**

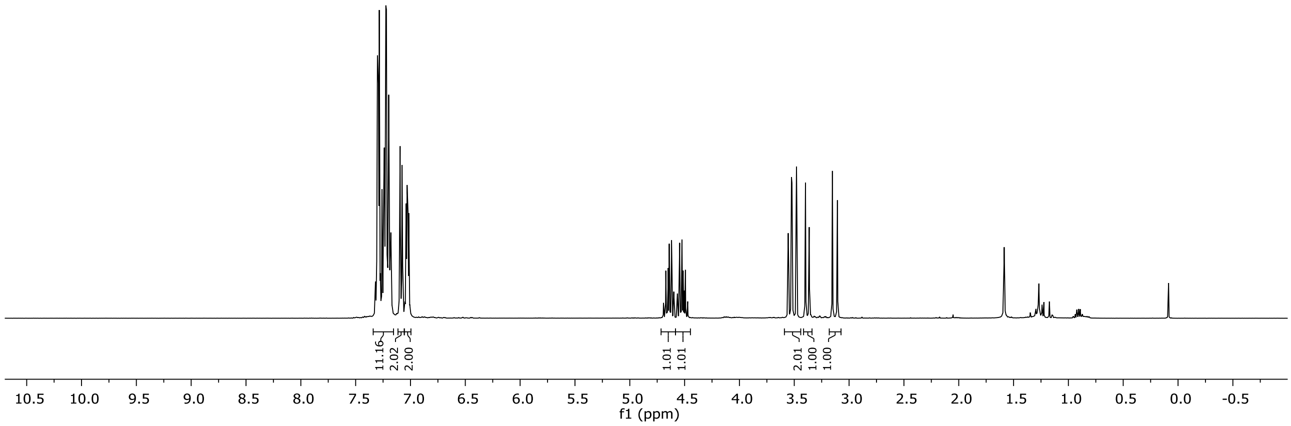
11.16
2.02
2.00
1.01
1.01
2.01
1.00
1.00
10.5 10.0 9.5 9.0 8.5 8.0 7.5 7.0 6.5 6.0 5.5 5.0 4.5 4.0 3.5 3.0 2.5 2.0 1.5 1.0 0.5 0.0 -0.5
f1 (ppm)

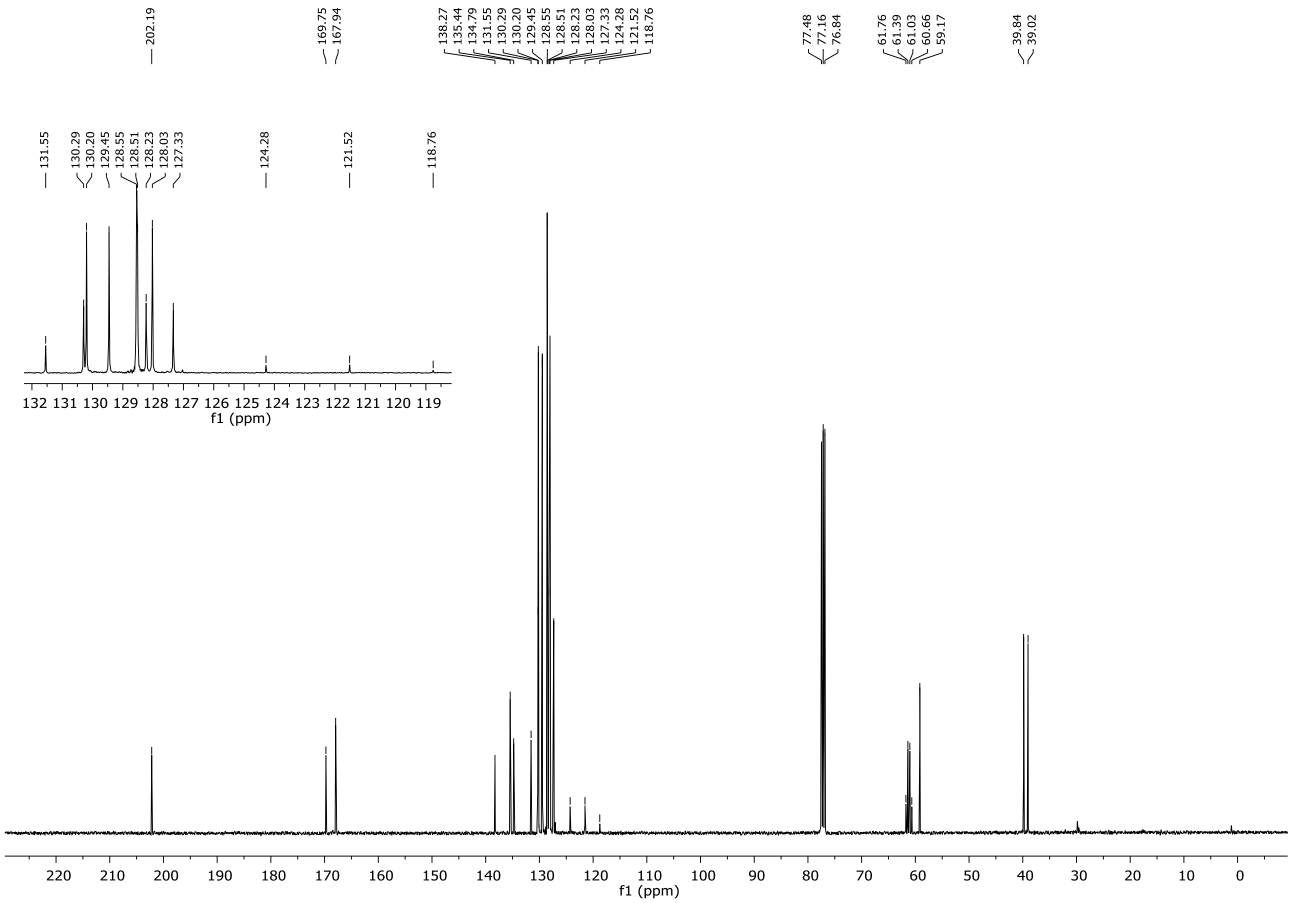
202.19
169.75
167.94
138.27
135.44
134.79
131.55
130.29
130.20
129.45
128.55
128.51
128.23
128.03
127.33
124.28
121.52
118.76
77.48
77.16
76.84
61.76
61.39
61.03
60.66
59.17
39.84
39.02
132 131 130 129 128 127 126 125 124 123 122 121 120 119
f1 (ppm)
220 210 200 190 180 170 160 150 140 130 120 110 100 90 80 70 60 50 40 30 20 10 0
f1 (ppm)

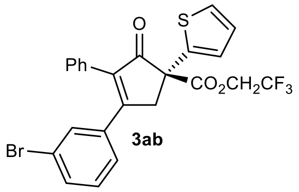

O
S
Ph
CO2CH2CF3
Br

3ab

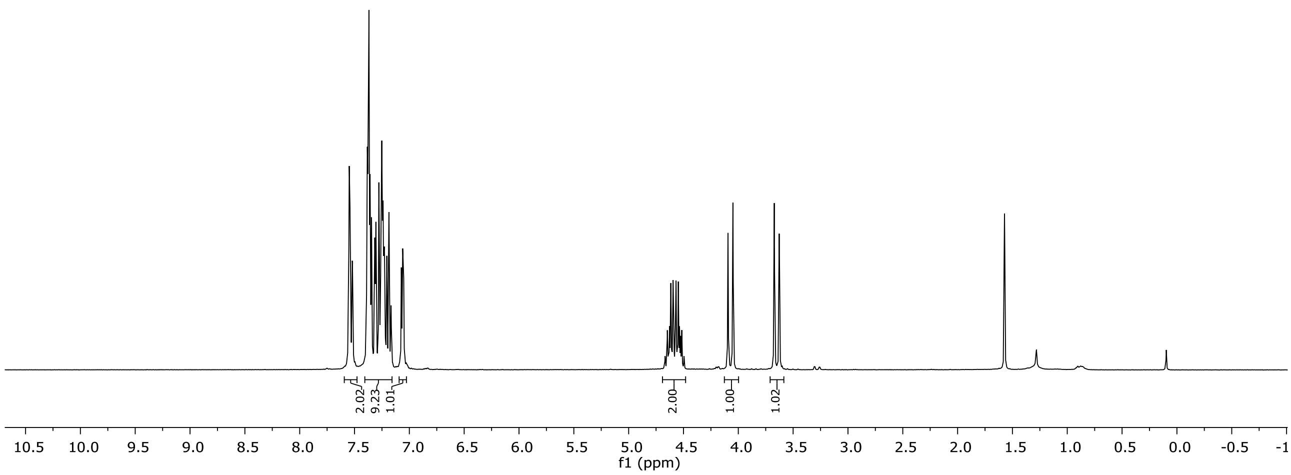

2.02
9.23
1.01
2.00
1.00
1.02
f1 (ppm)

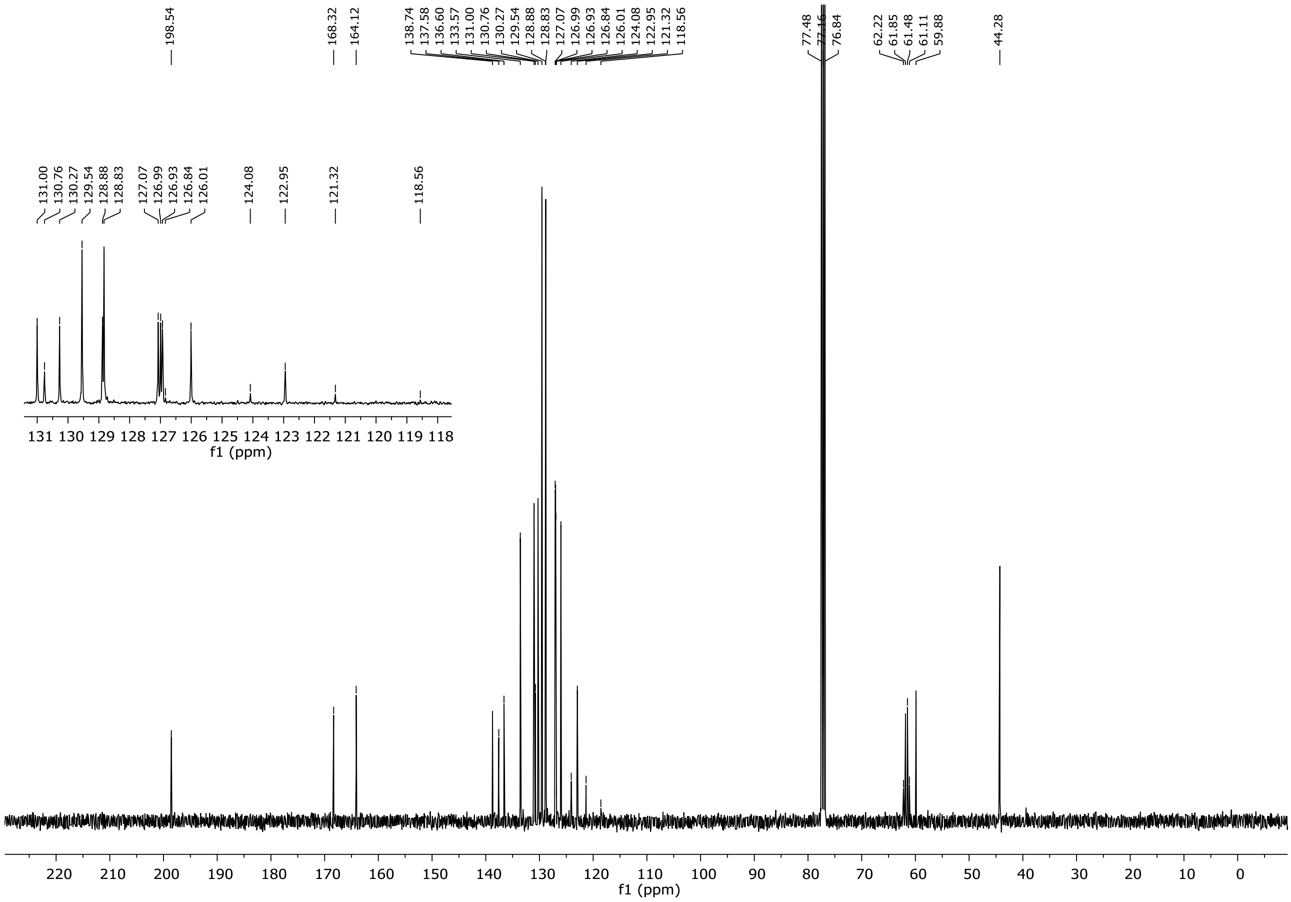

198.54
168.32
164.12
138.74
137.58
136.60
133.57
131.00
130.76
130.27
129.54
128.88
128.83
127.07
126.99
126.93
126.84
126.01
124.08
122.95
121.32
118.56
77.48
76.84
62.22
61.85
61.48
61.11
59.88
44.28
f1 (ppm)

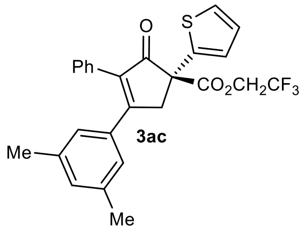
O
S
Ph
CO2CH2CF3
3ac
Me
Me

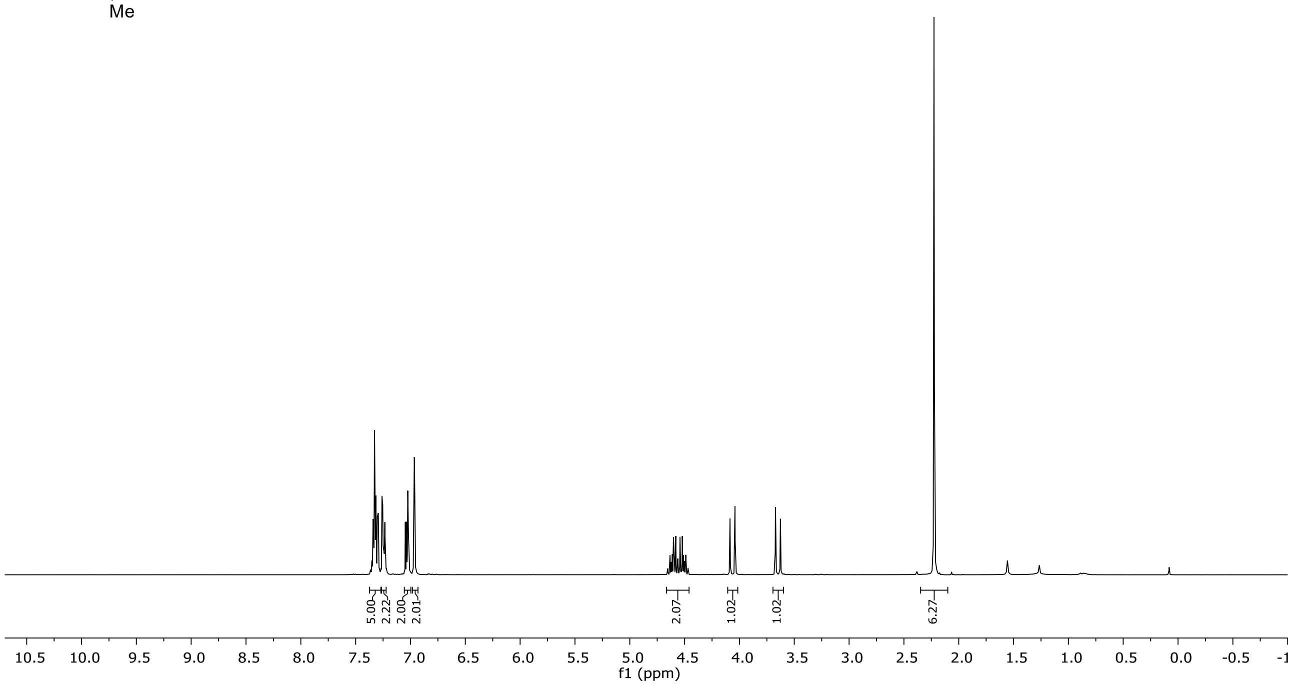
5.00
2.22
2.00
2.01
2.07
1.02
1.02
6.27
f1 (ppm)

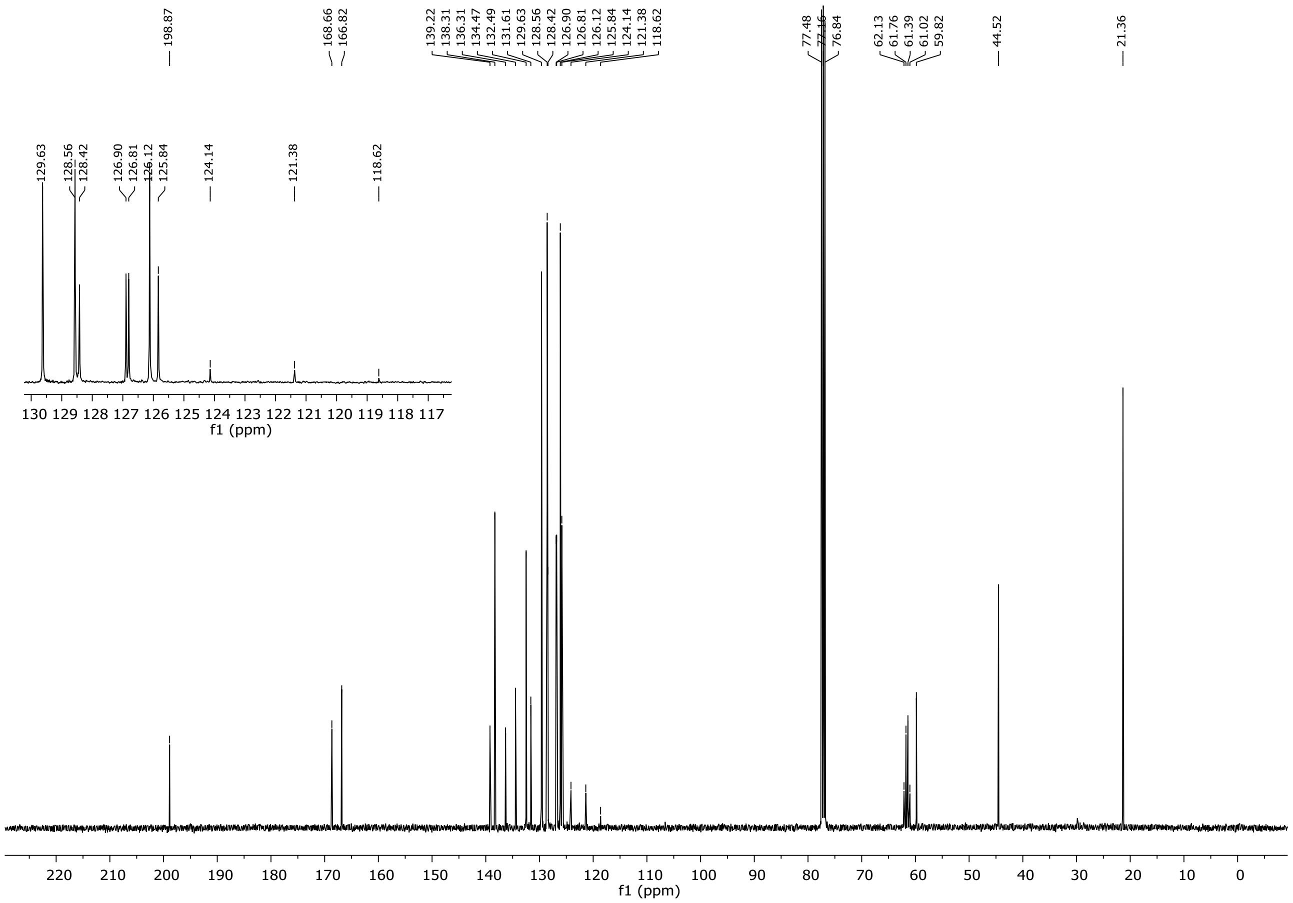
198.87
168.66
166.82
139.22
138.31
136.31
134.47
132.49
131.61
129.63
128.56
128.42
126.90
126.81
126.12
125.84
124.14
121.38
118.62
77.48
77.16
76.84
62.13
61.76
61.39
61.02
59.82
44.52
21.36
f1 (ppm)

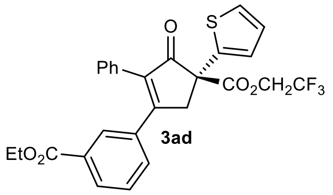
Ph
O
S
$CO_2CH_2CF_3$
EtO_2C

3ad

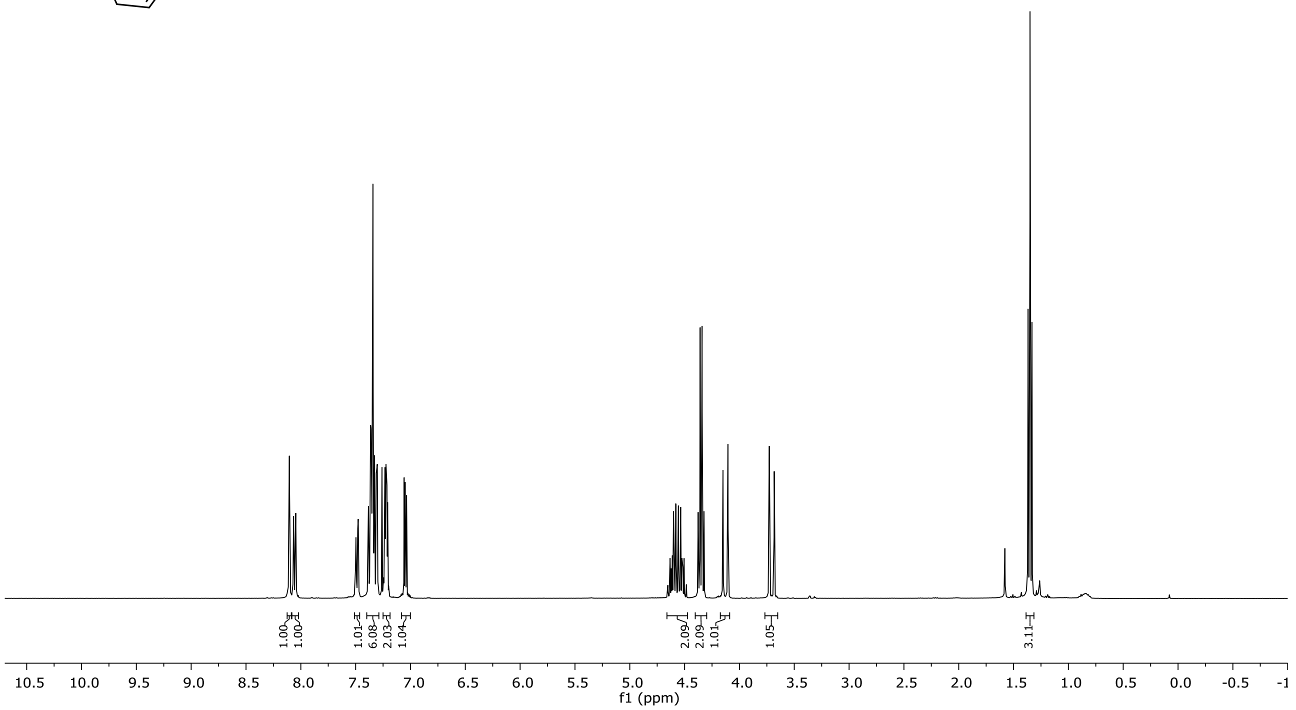
1.00
1.00
1.01
6.08
2.03
1.04
2.09
2.09
1.01
1.05
3.11
10.5 10.0 9.5 9.0 8.5 8.0 7.5 7.0 6.5 6.0 5.5 5.0 4.5 4.0 3.5 3.0 2.5 2.0 1.5 1.0 0.5 0.0 -0.5 -1
f1 (ppm)

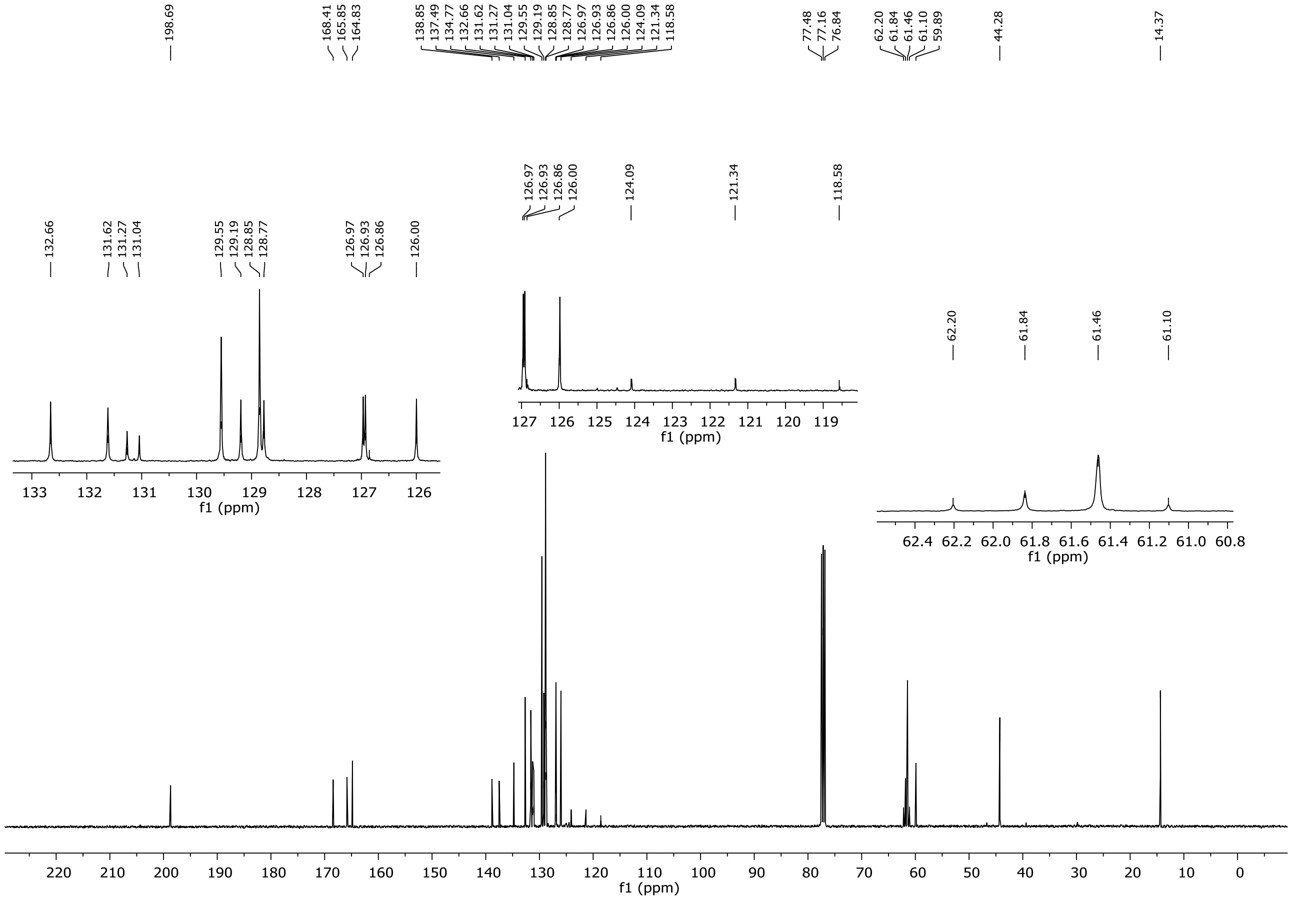
198.69
168.41
165.85
164.83
138.85
137.49
134.77
132.66
131.62
131.27
131.04
129.55
129.19
128.85
128.77
126.97
126.93
126.86
126.00
124.09
121.34
118.58
77.48
77.16
76.84
62.20
61.84
61.46
61.10
59.89
44.28
14.37
132.66
131.62
131.27
131.04
129.55
129.19
128.85
128.77
126.97
126.93
126.86
126.00
133 132 131 130 129 128 127 126
f1 (ppm)
126.97
126.93
126.86
126.00
124.09
121.34
118.58
127 126 125 124 123 122 121 120 119
f1 (ppm)
62.20
61.84
61.46
61.10
62.4 62.2 62.0 61.8 61.6 61.4 61.2 61.0 60.8
f1 (ppm)
220 210 200 190 180 170 160 150 140 130 120 110 100 90 80 70 60 50 40 30 20 10 0
f1 (ppm)

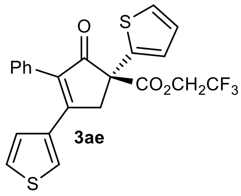
O
S
Ph
CO2CH2CF3
S

3ae

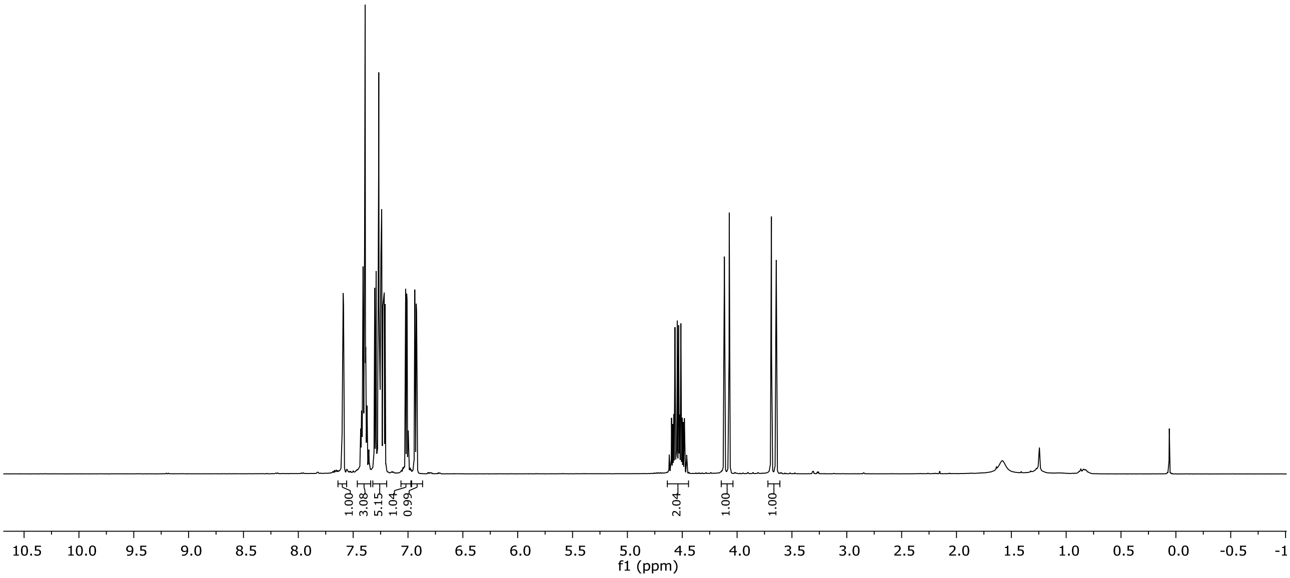
1.00
3.08
5.15
1.04
0.99
2.04
1.00
1.00
10.5 10.0 9.5 9.0 8.5 8.0 7.5 7.0 6.5 6.0 5.5 5.0 4.5 4.0 3.5 3.0 2.5 2.0 1.5 1.0 0.5 0.0 -0.5 -1
f1 (ppm)

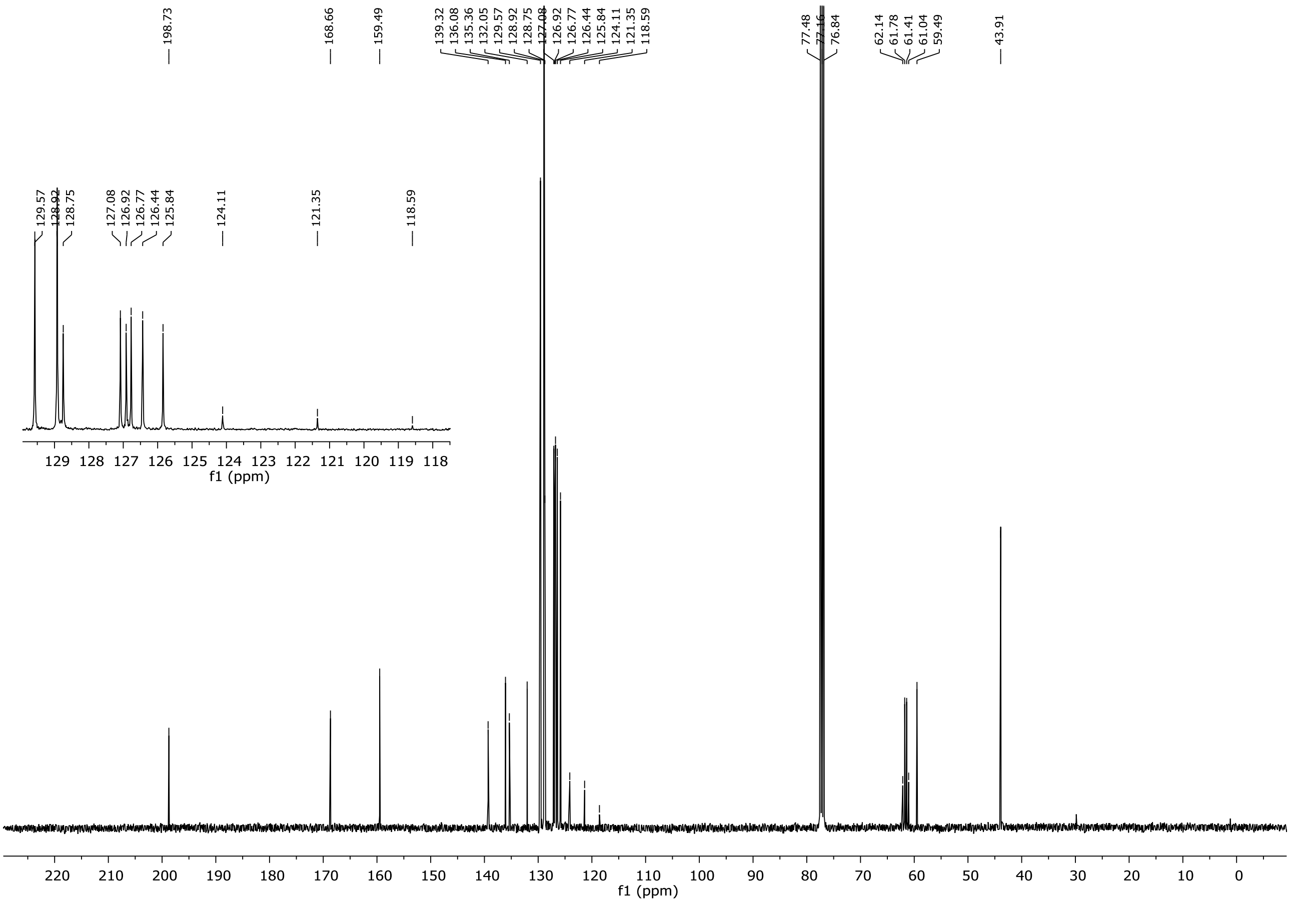
198.73
168.66
159.49
139.32
136.08
135.36
132.05
129.57
128.92
128.75
127.08
126.92
126.77
126.44
125.84
124.11
121.35
118.59
77.48
77.16
76.84
62.14
61.78
61.41
61.04
59.49
43.91
129.57
128.92
128.75
127.08
126.92
126.77
126.44
125.84
124.11
121.35
118.59
129 128 127 126 125 124 123 122 121 120 119 118
f1 (ppm)
220 210 200 190 180 170 160 150 140 130 120 110 100 90 80 70 60 50 40 30 20 10 0
f1 (ppm)

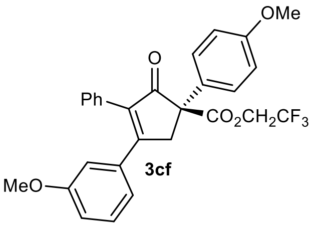

3cf

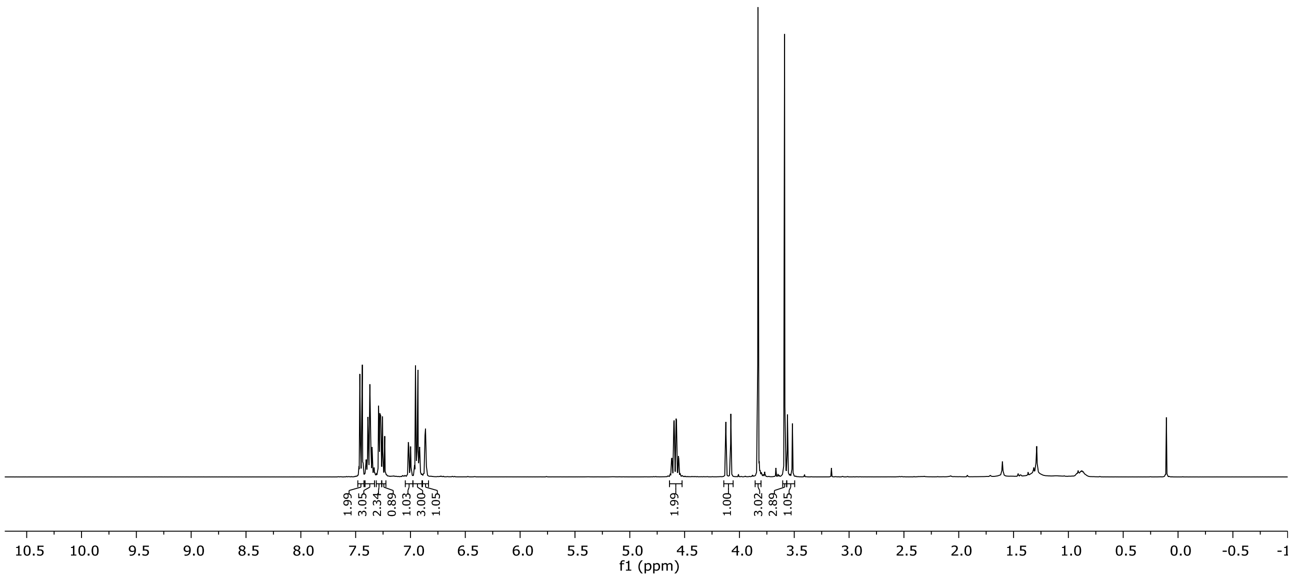

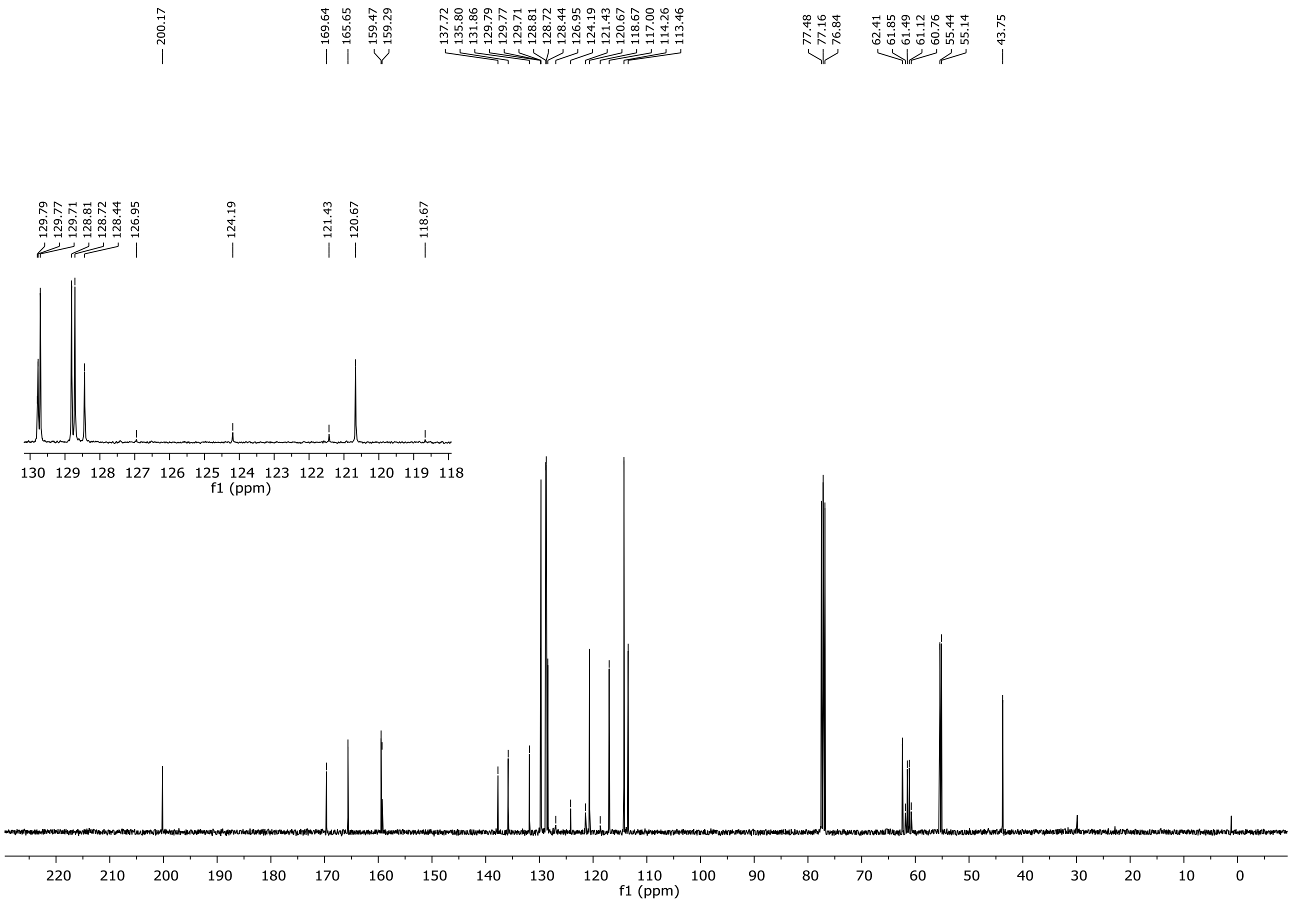

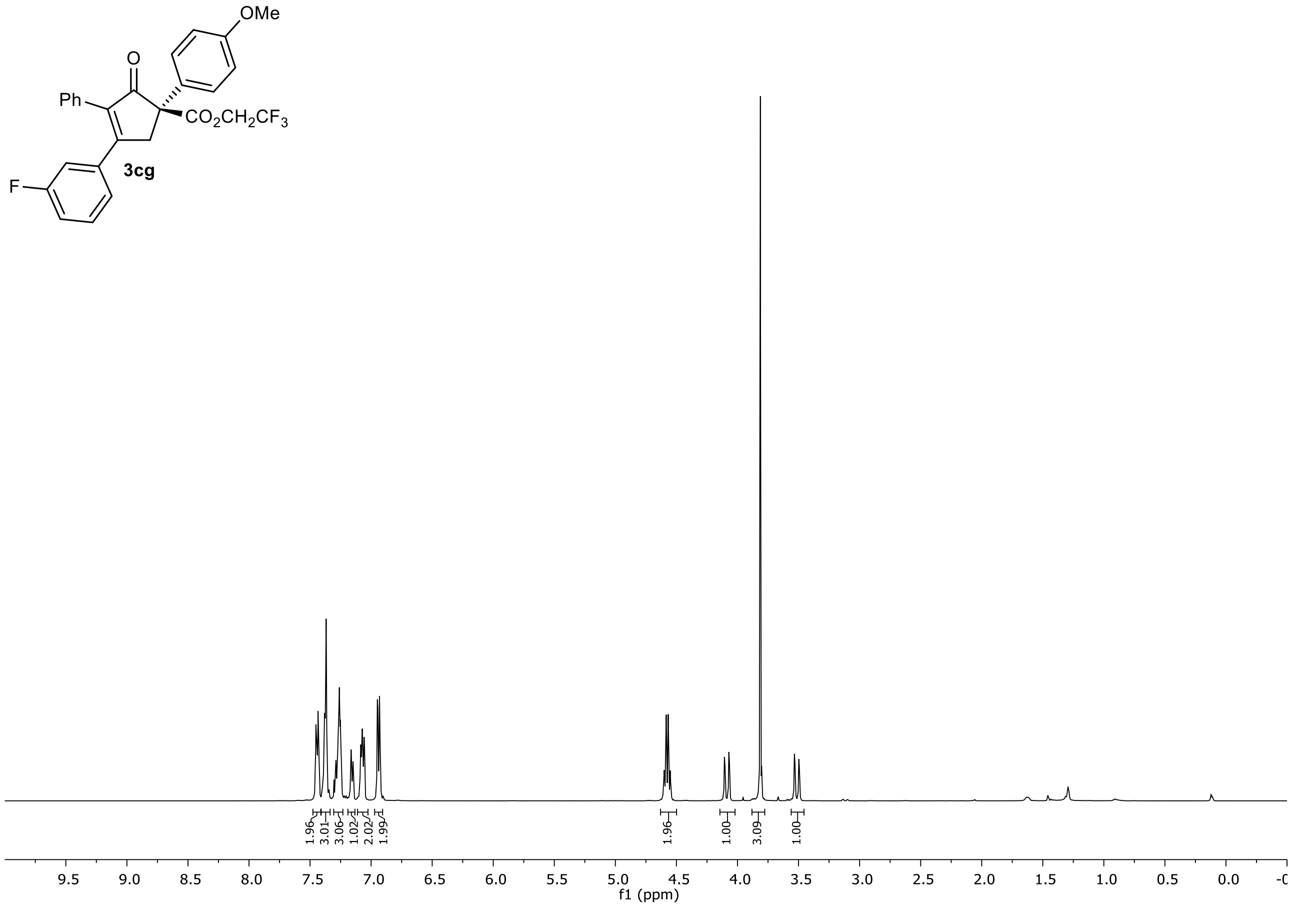
OMe
O
Ph
$CO_2CH_2CF_3$
F
3cg
f1 (ppm)

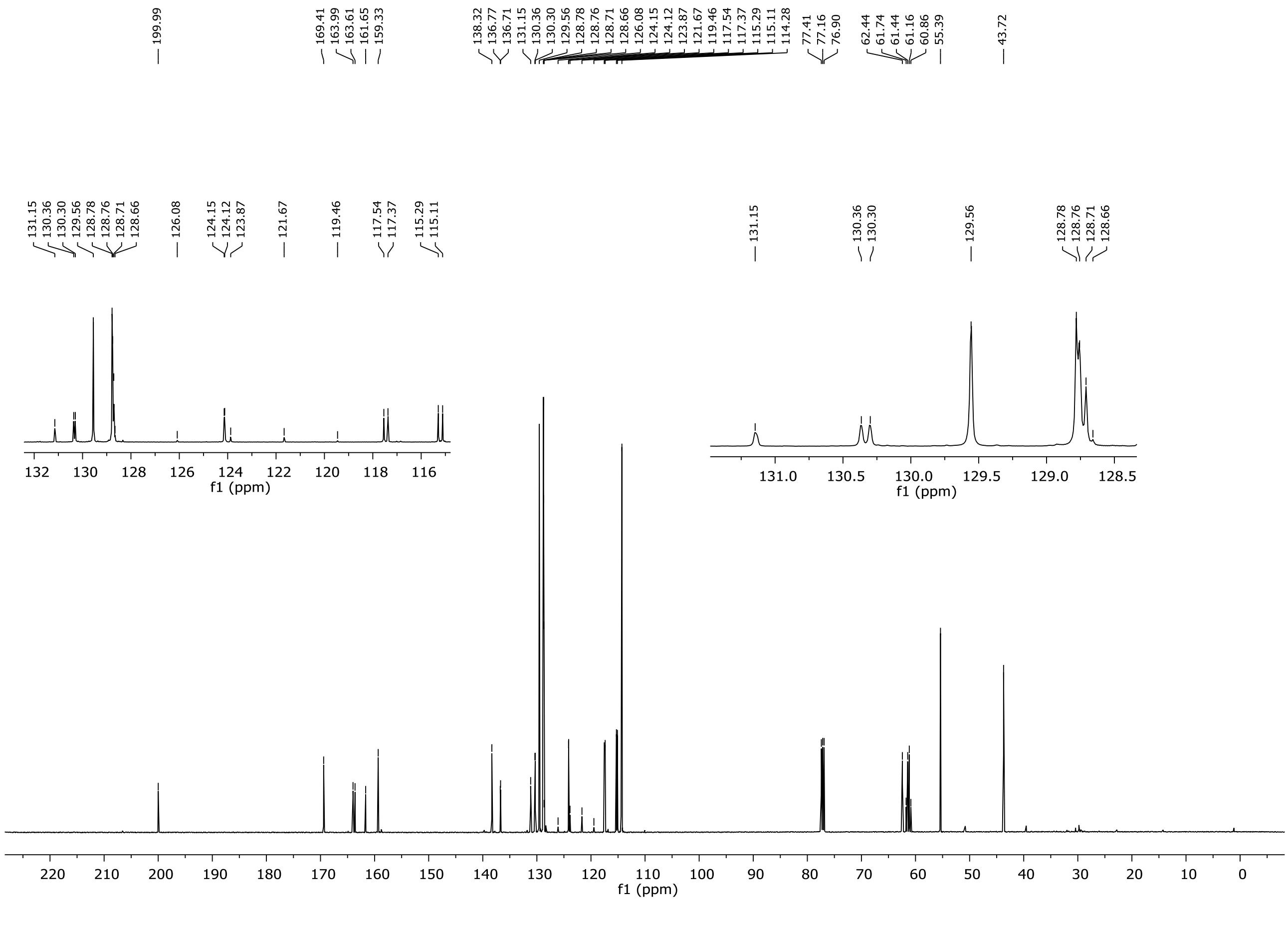
f1 (ppm)

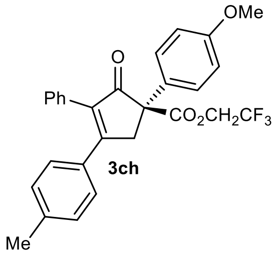
OMe
O
Ph
$CO_2CH_2CF_3$
3ch
Me

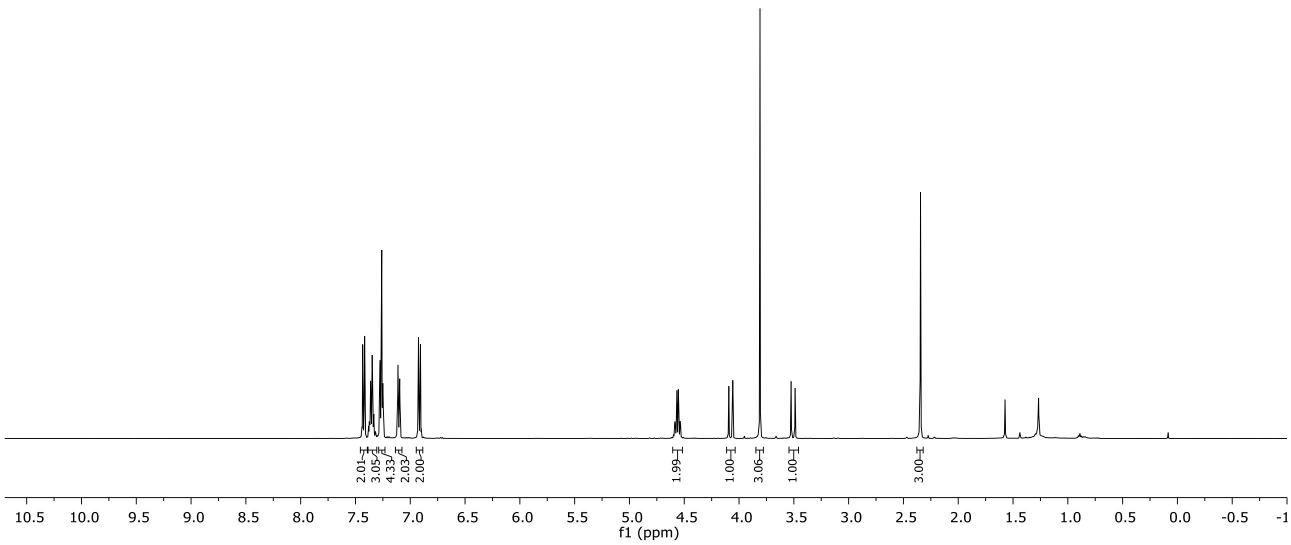
2.01
3.05
4.33
2.03
2.00
1.99
1.00
3.06
1.00
3.00
f1 (ppm)

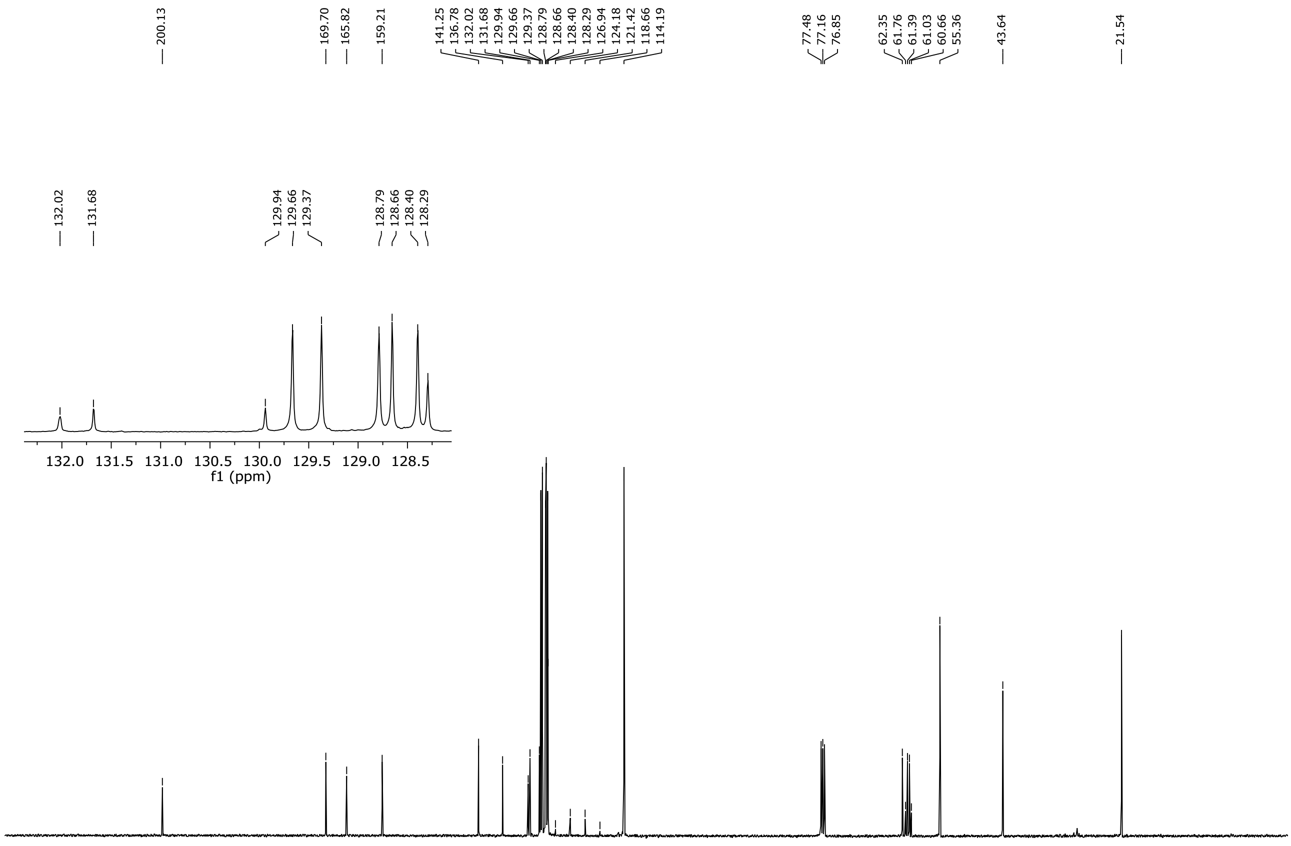
200.13
169.70
165.82
159.21
141.25 136.78 132.02 131.68 129.94 129.66 129.37 128.79 128.66 128.40 128.29 126.94 124.18 121.42 118.66 114.19
77.48 77.16 76.85
62.35 61.76 61.39 61.03 60.66 55.36
43.64
21.54
132.02 131.68
129.94 129.66 129.37
128.79 128.66 128.40 128.29
f1 (ppm)

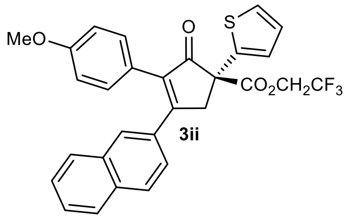
MeO
O
S
$CO_2CH_2CF_3$

3ii

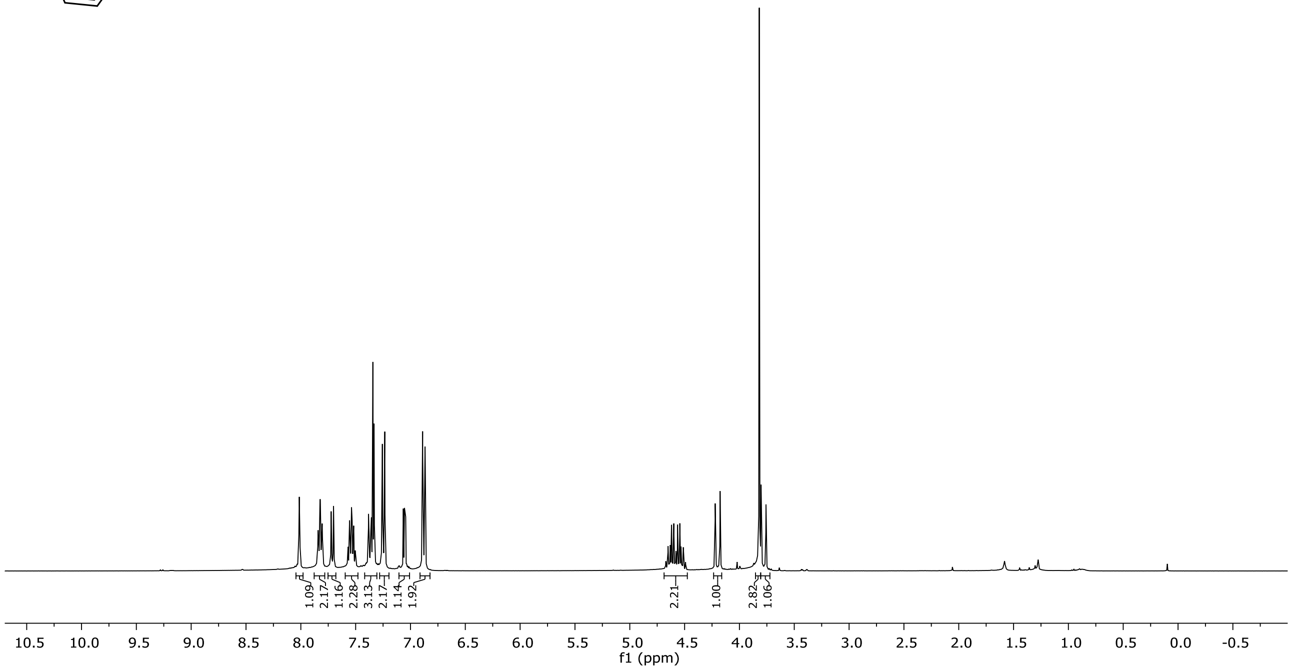
1.09
2.17
1.16
2.28
3.13
2.17
1.14
1.92
2.21
1.00
2.82
1.06
f1 (ppm)

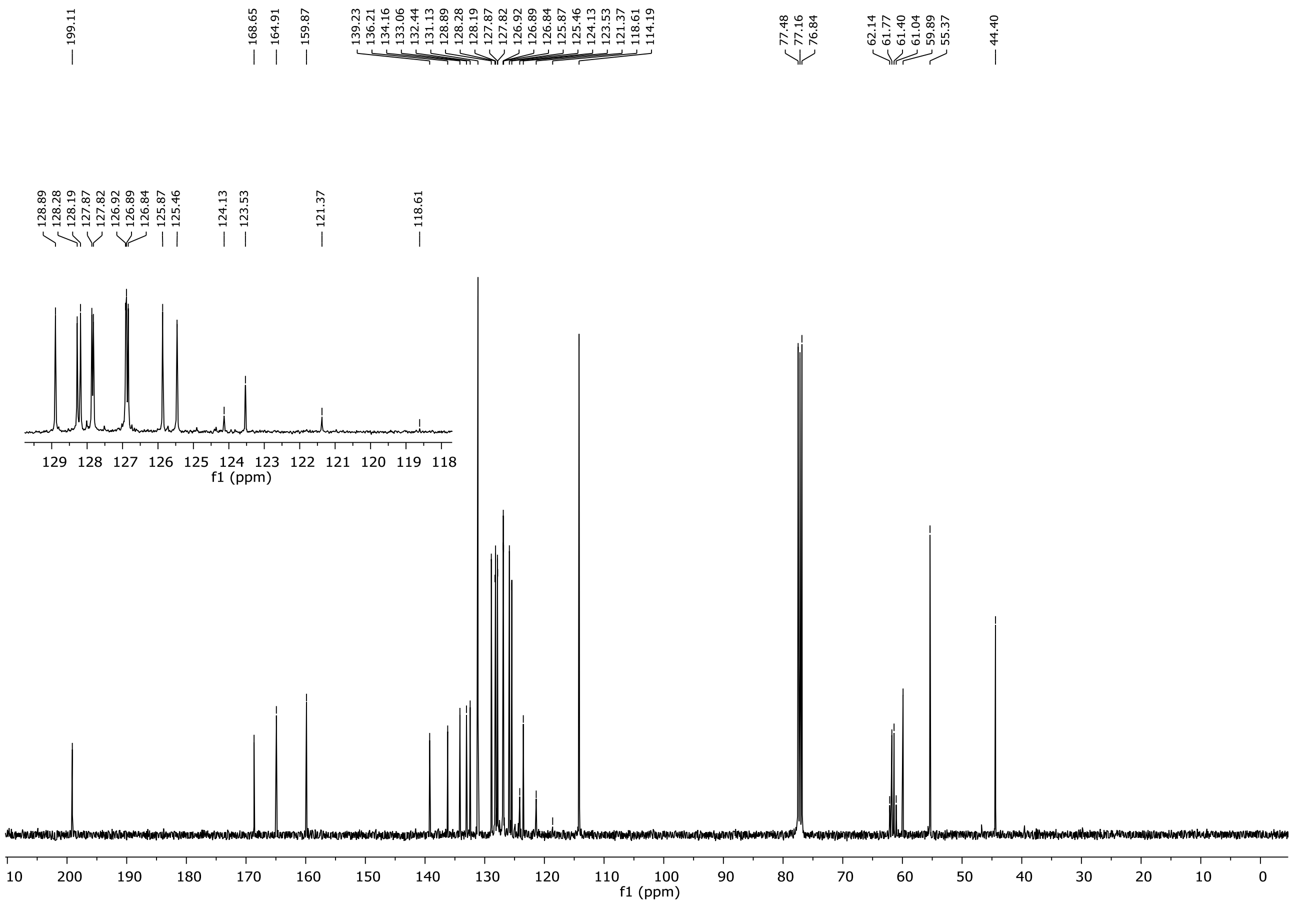
199.11
168.65
164.91
159.87
139.23
136.21
134.16
133.06
132.44
131.13
128.89
128.28
128.19
127.87
127.82
126.92
126.89
126.84
125.87
125.46
124.13
123.53
121.37
118.61
114.19
77.48
77.16
76.84
62.14
61.77
61.40
61.04
59.89
55.37
44.40
f1 (ppm)

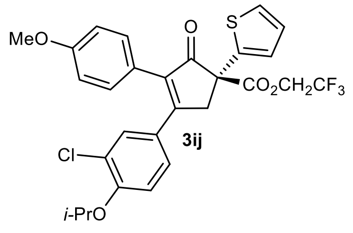
MeO
O
S
CO2CH2CF3
Cl
i-PrO

3ij

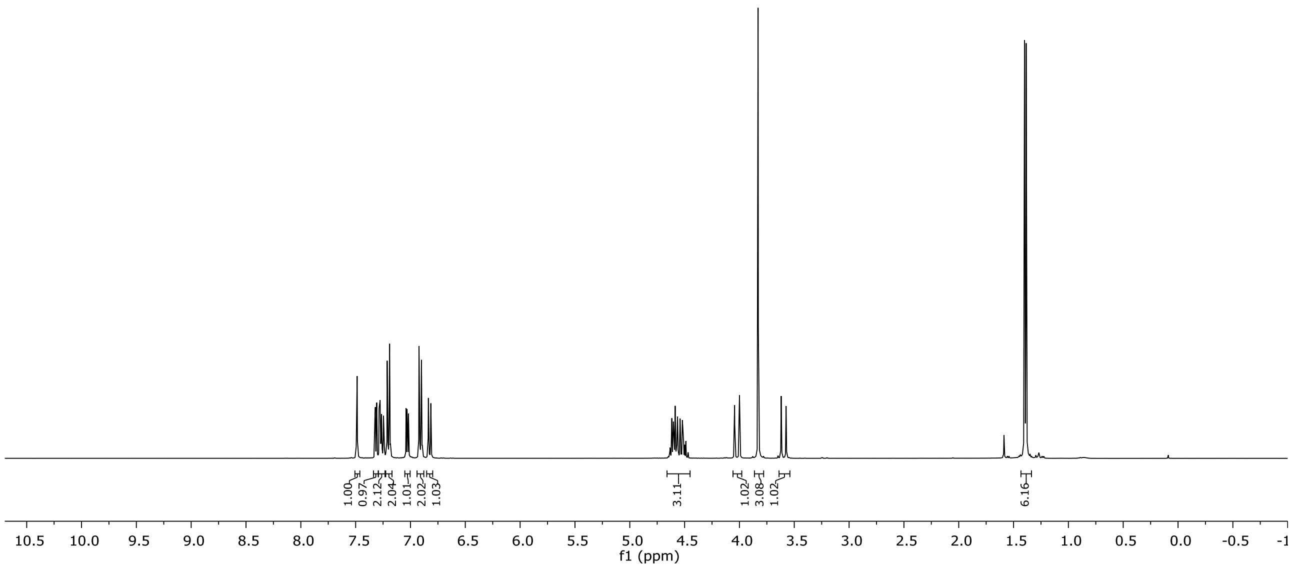
1.00
0.97
2.12
2.04
1.01
2.02
1.03
3.11
1.02
3.08
1.02
6.16
10.5 10.0 9.5 9.0 8.5 8.0 7.5 7.0 6.5 6.0 5.5 5.0 4.5 4.0 3.5 3.0 2.5 2.0 1.5 1.0 0.5 0.0 -0.5 -1
f1 (ppm)

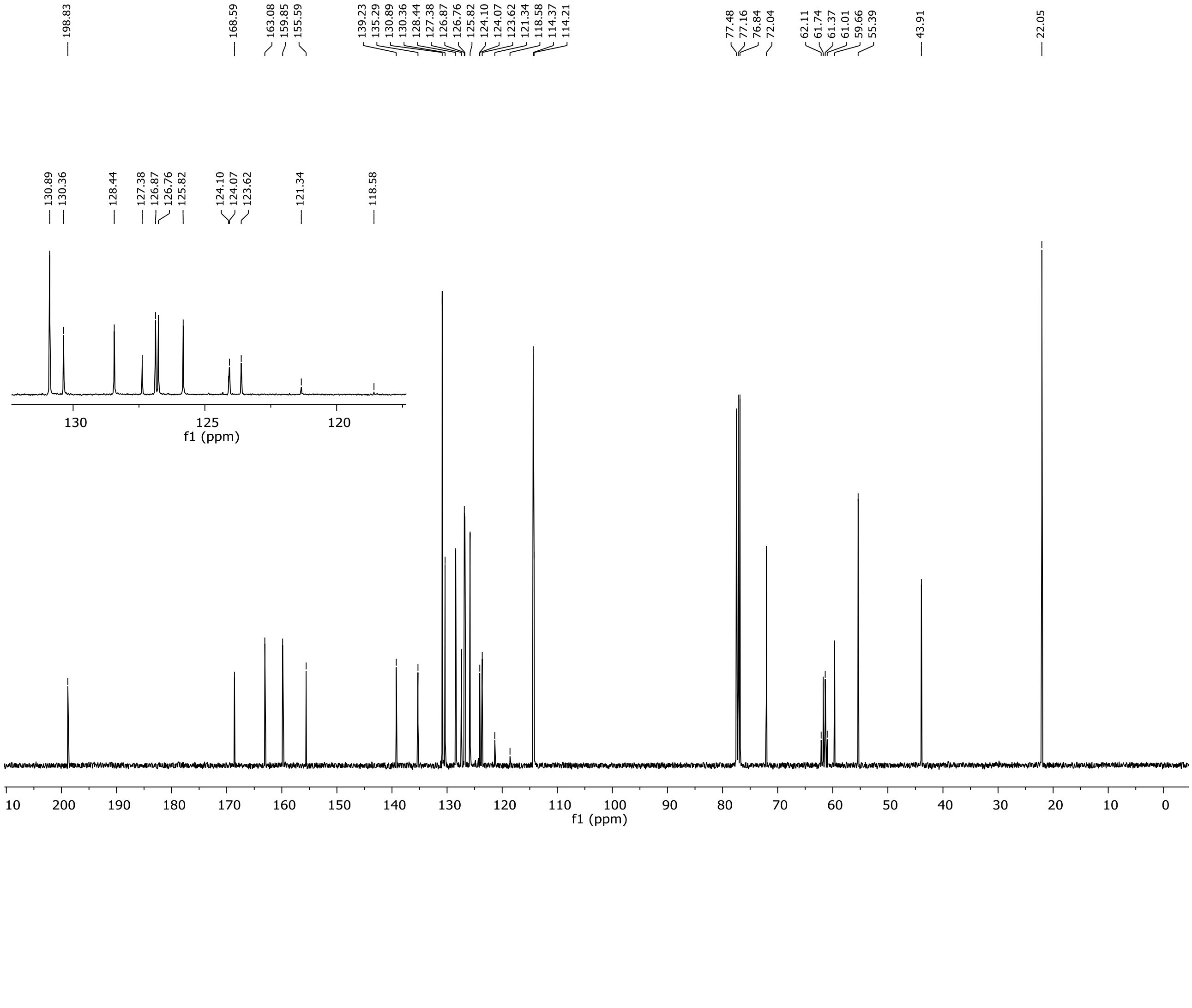
198.83
168.59
163.08
159.85
155.59
139.23
135.29
130.89
130.36
128.44
127.38
126.87
126.76
125.82
124.10
124.07
123.62
121.34
118.58
114.37
114.21
77.48
77.16
76.84
72.04
62.11
61.74
61.37
61.01
59.66
55.39
43.91
22.05
130.89
130.36
128.44
127.38
126.87
126.76
125.82
124.10
124.07
123.62
121.34
118.58
130 125 120
f1 (ppm)
10 200 190 180 170 160 150 140 130 120 110 100 90 80 70 60 50 40 30 20 10 0
f1 (ppm)

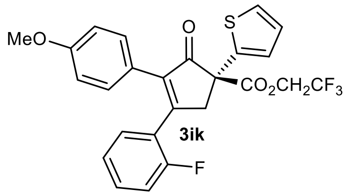
MeO
O
S
$CO_2CH_2CF_3$
3ik
F

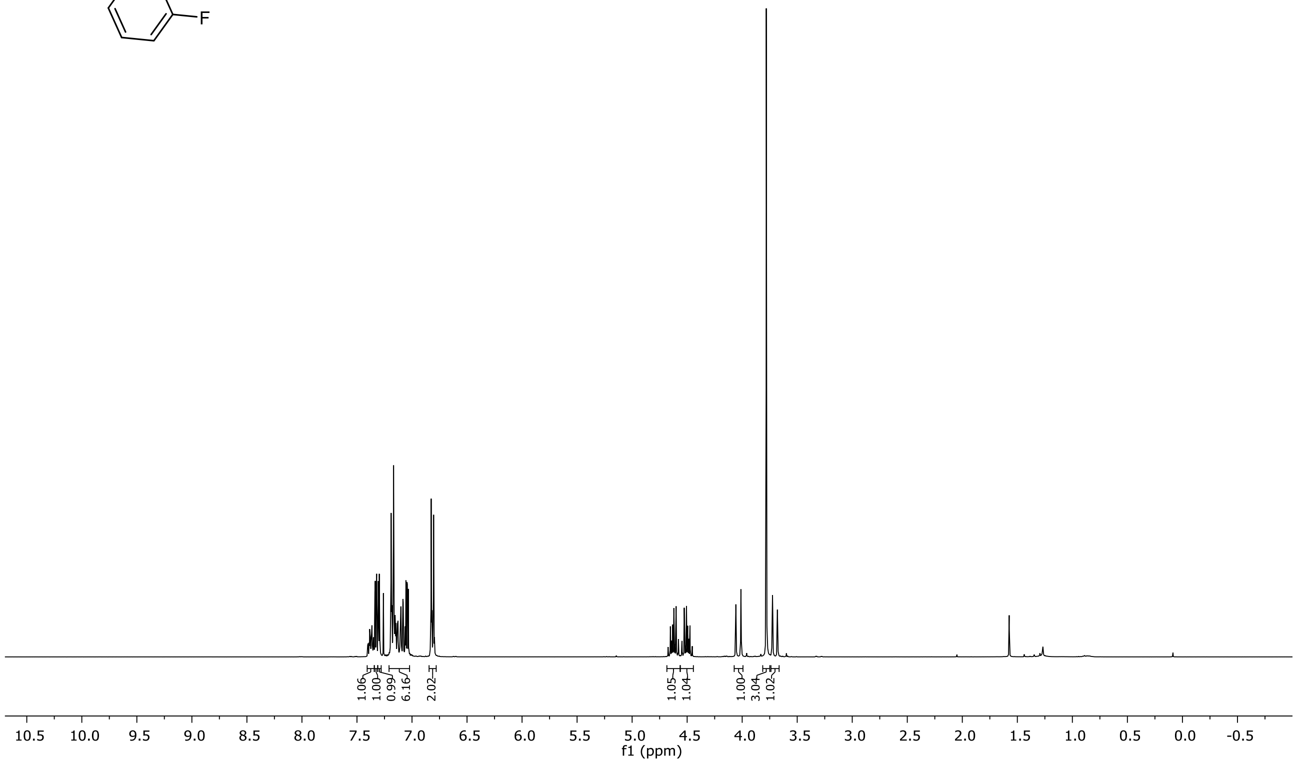
1.06
1.00
0.99
6.16
2.02
1.05
1.04
1.00
3.04
1.02
10.5 10.0 9.5 9.0 8.5 8.0 7.5 7.0 6.5 6.0 5.5 5.0 4.5 4.0 3.5 3.0 2.5 2.0 1.5 1.0 0.5 0.0 -0.5
f1 (ppm)

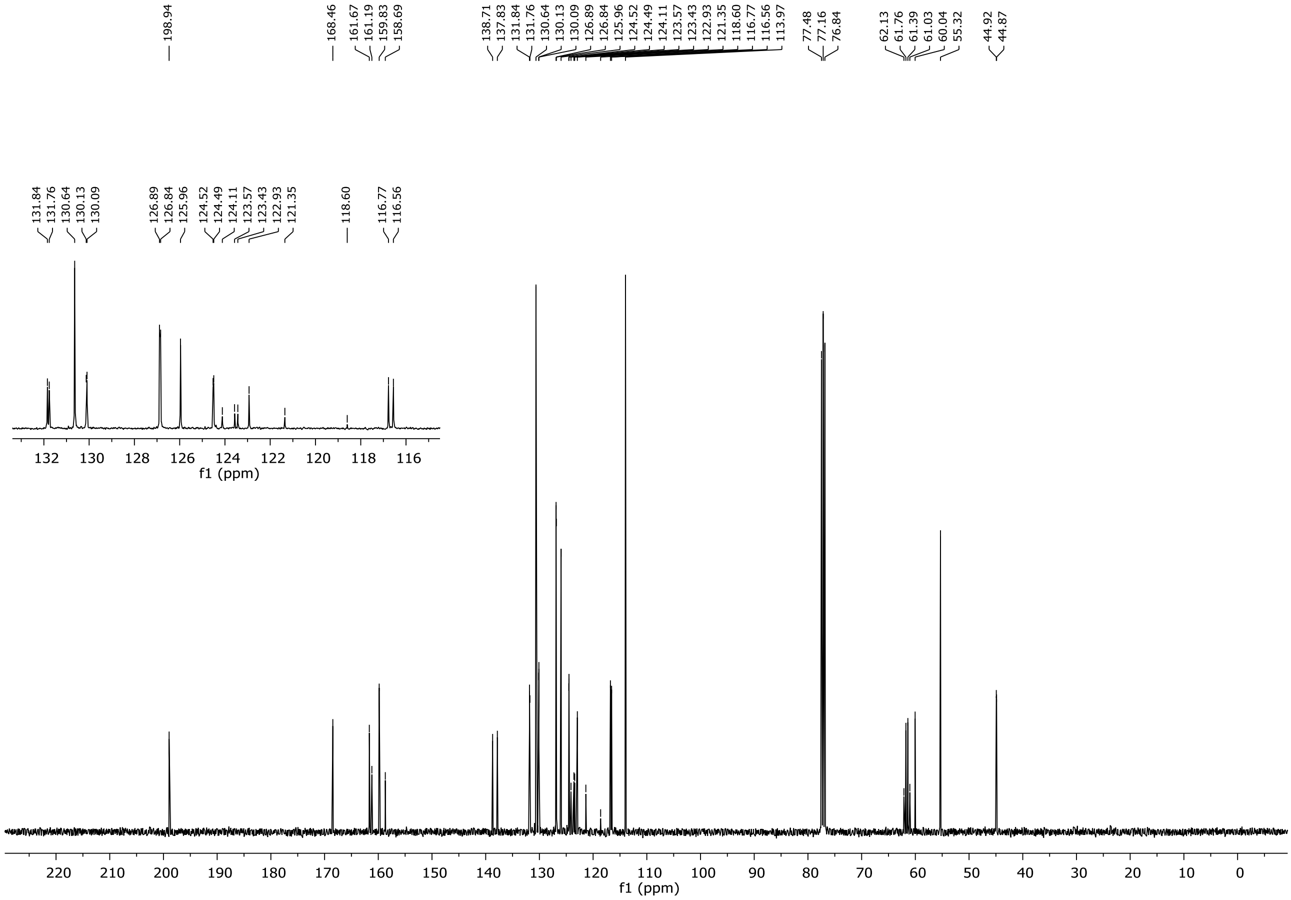
198.94
168.46
161.67
161.19
159.83
158.69
138.71
137.83
131.84
131.76
130.64
130.13
130.09
126.89
126.84
125.96
124.52
124.49
124.11
123.57
123.43
122.93
121.35
118.60
116.77
116.56
113.97
77.48
77.16
76.84
62.13
61.76
61.39
61.03
60.04
55.32
44.92
44.87
132 130 128 126 124 122 120 118 116
f1 (ppm)
220 210 200 190 180 170 160 150 140 130 120 110 100 90 80 70 60 50 40 30 20 10 0
f1 (ppm)

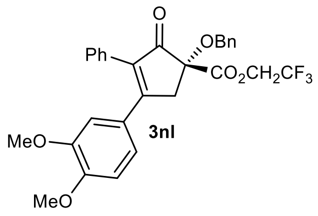

3nl

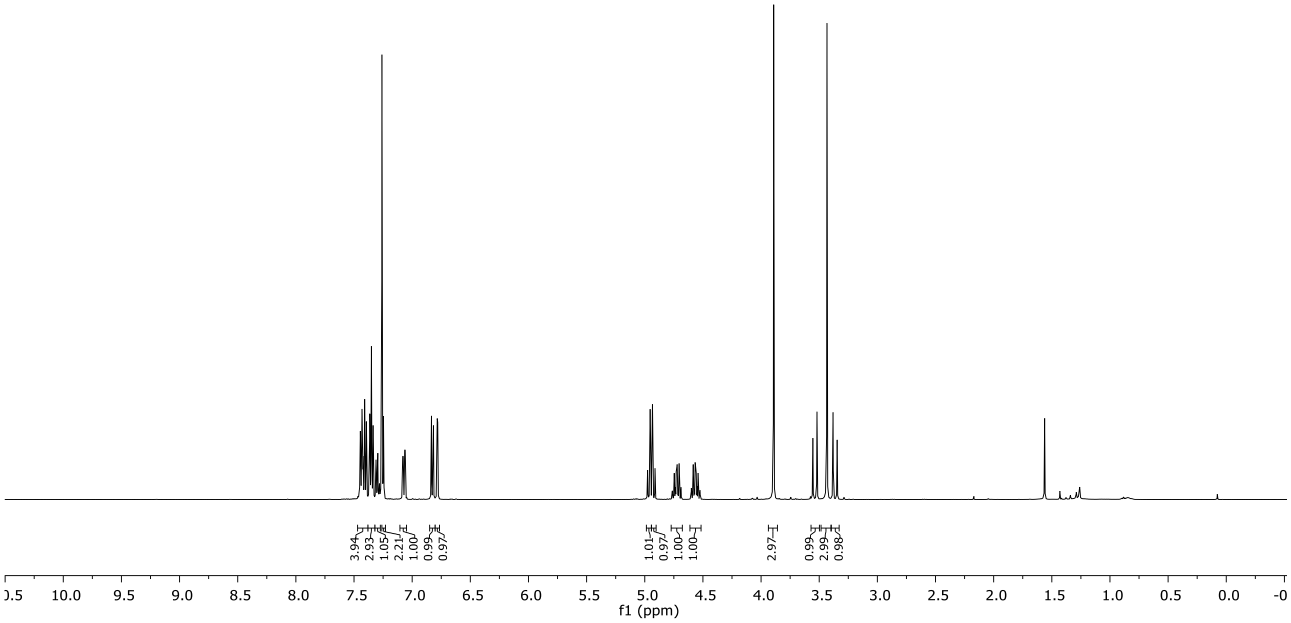

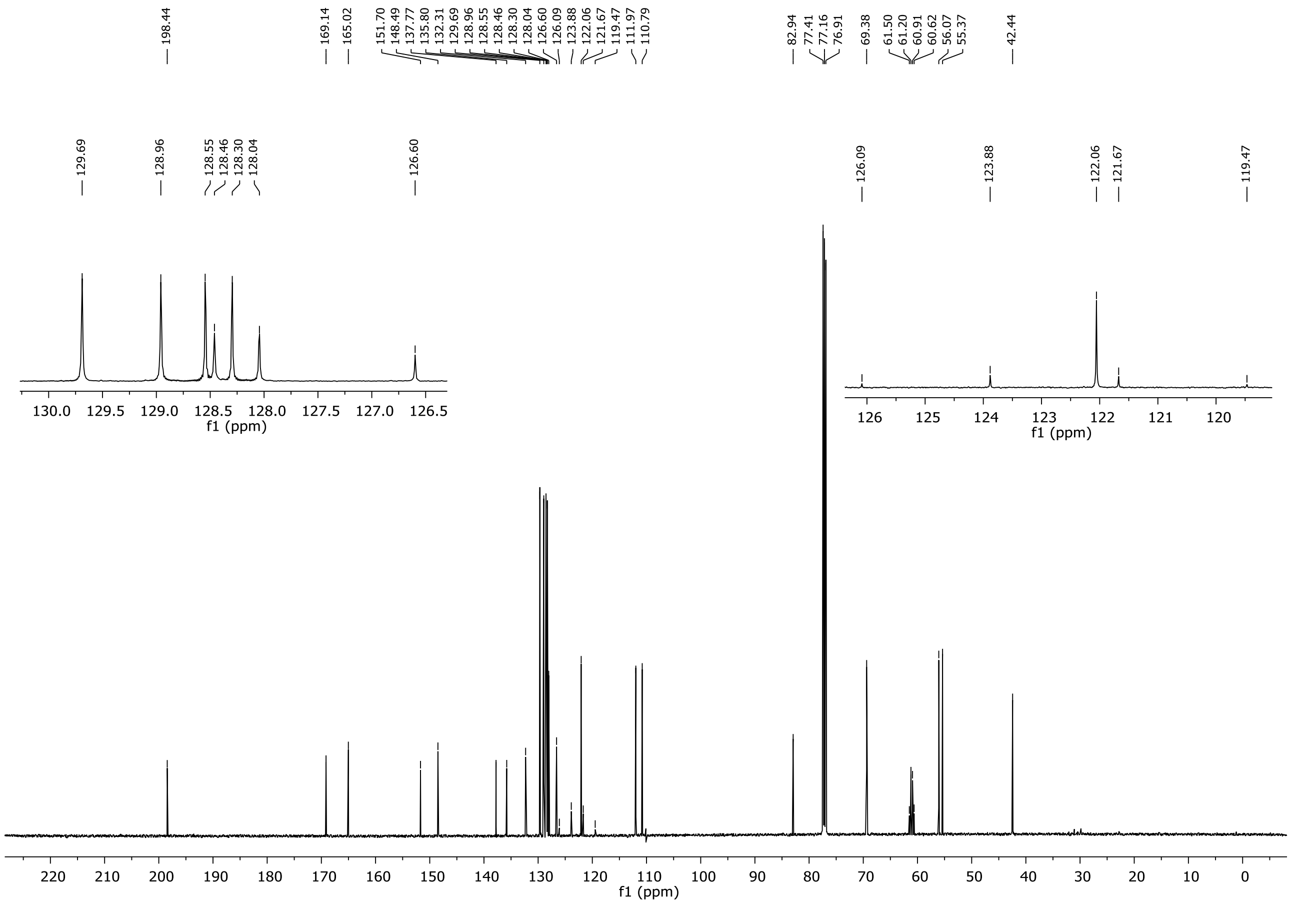

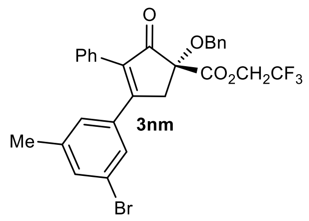
O
OBn
Ph
CO2CH2CF3
3nm
Me
Br

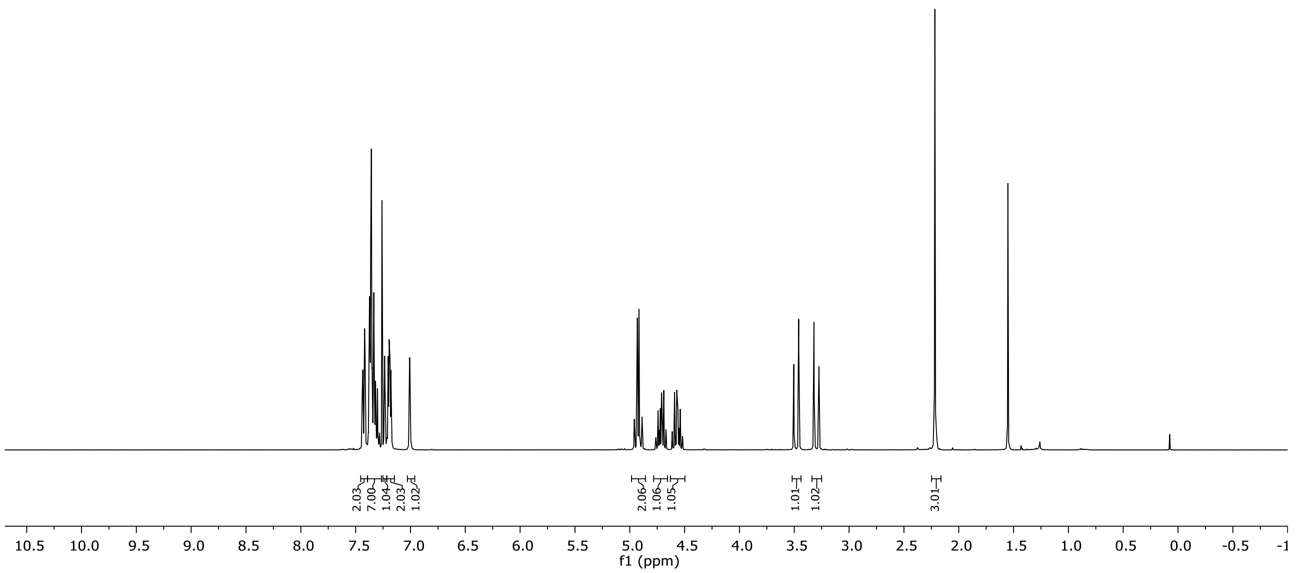
2.03
7.00
1.04
2.03
1.02
2.06
1.06
1.05
1.01
1.02
3.01
10.5 10.0 9.5 9.0 8.5 8.0 7.5 7.0 6.5 6.0 5.5 5.0 4.5 4.0 3.5 3.0 2.5 2.0 1.5 1.0 0.5 0.0 -0.5 -1
f1 (ppm)

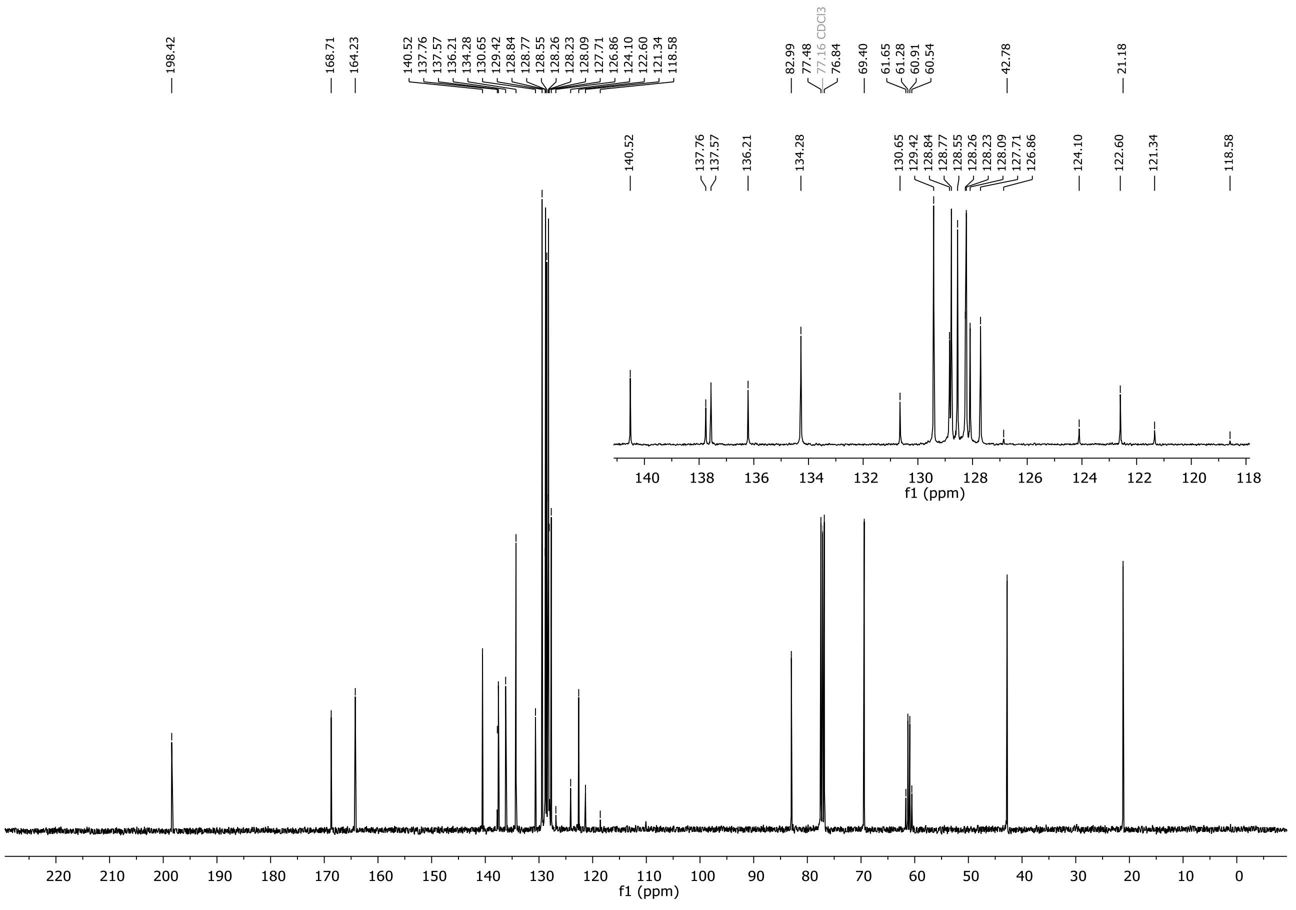
198.42
168.71
164.23
140.52
137.76
137.57
136.21
134.28
130.65
129.42
128.84
128.77
128.55
128.26
128.23
128.09
127.71
126.86
124.10
122.60
121.34
118.58
82.99
77.48
77.16 CDCl3
76.84
69.40
61.65
61.28
60.91
60.54
42.78
21.18
140 138 136 134 132 130 128 126 124 122 120 118
f1 (ppm)
220 210 200 190 180 170 160 150 140 130 120 110 100 90 80 70 60 50 40 30 20 10 0
f1 (ppm)

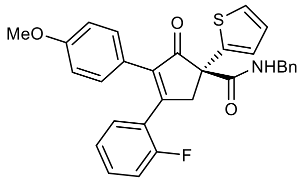

8

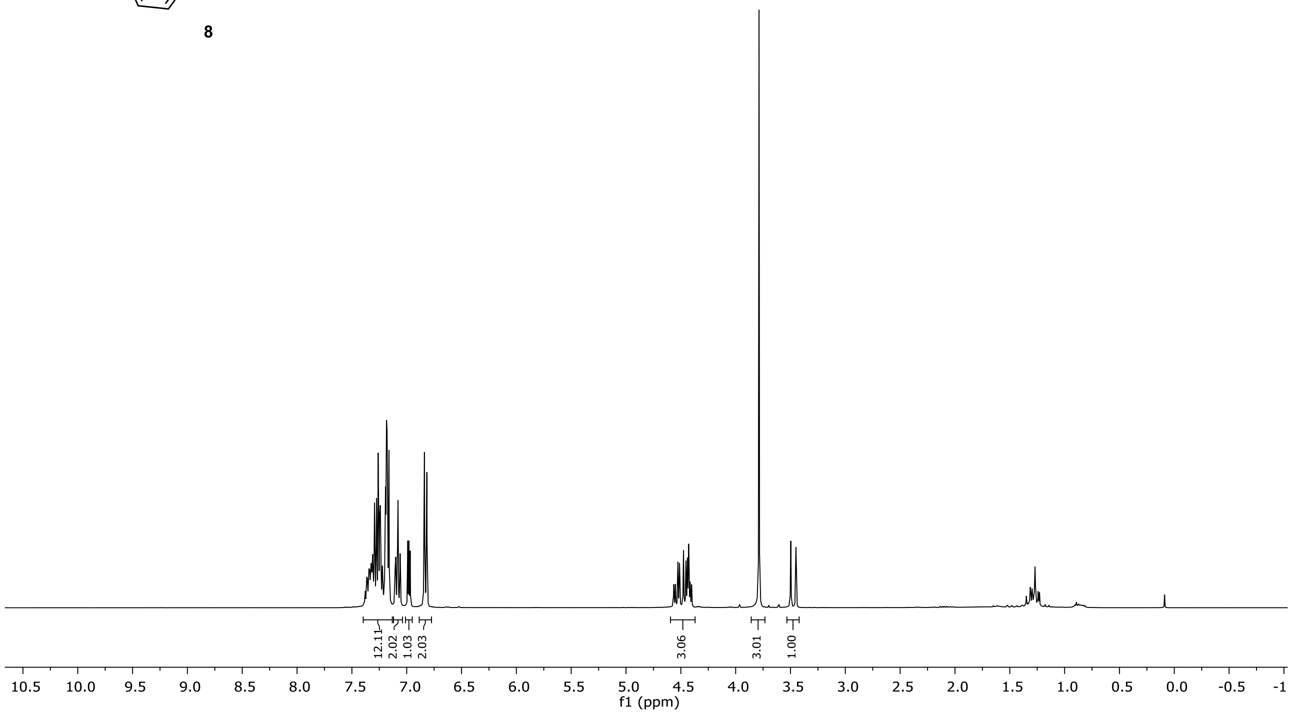

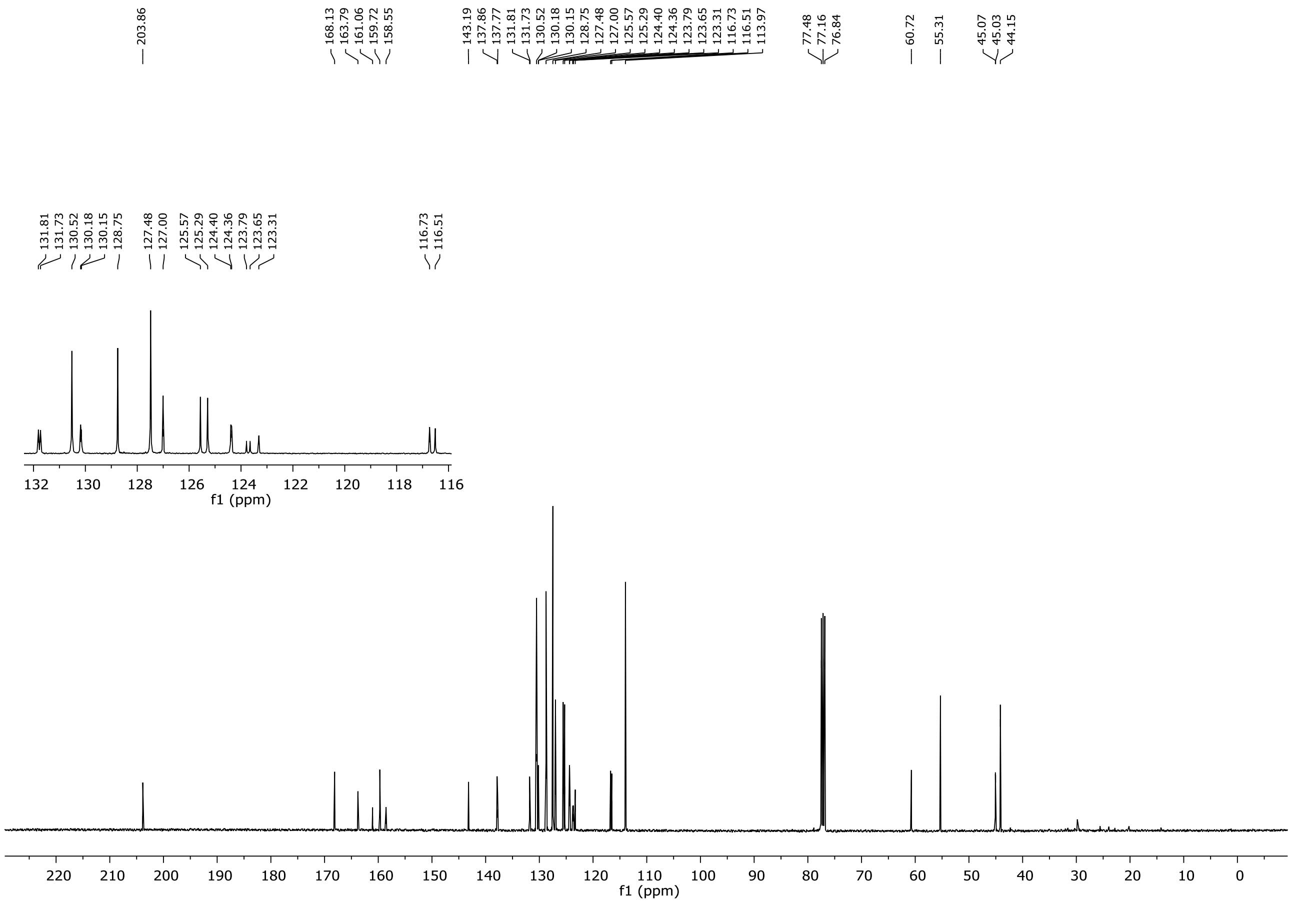

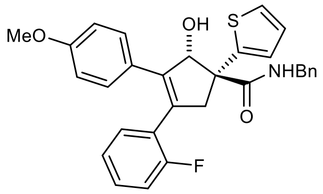

9

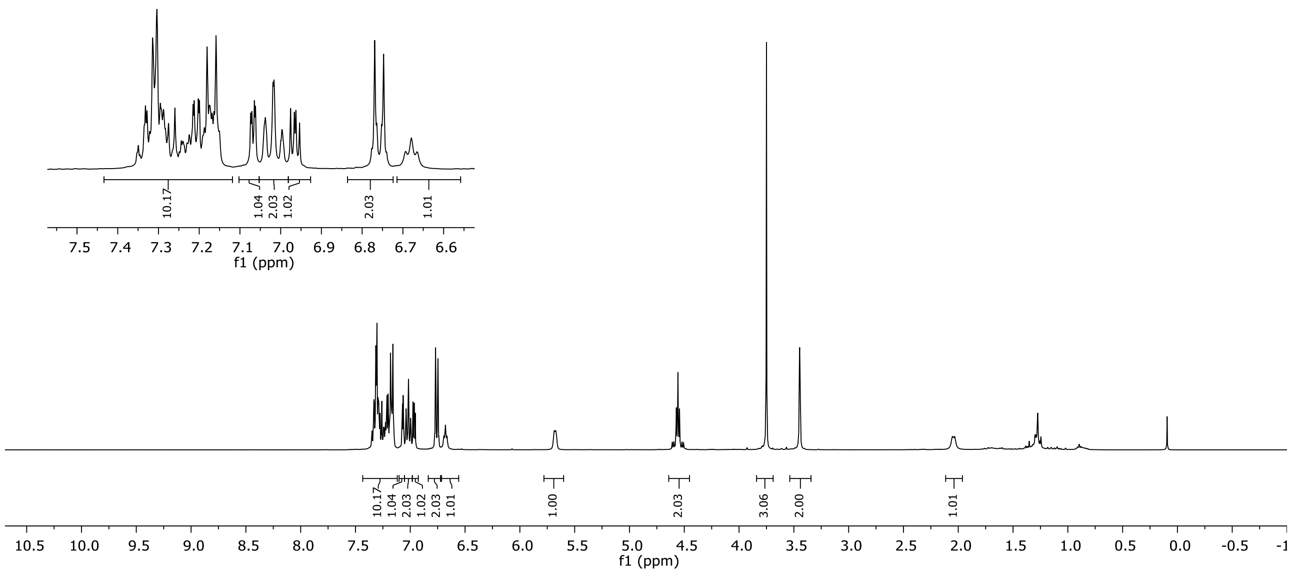

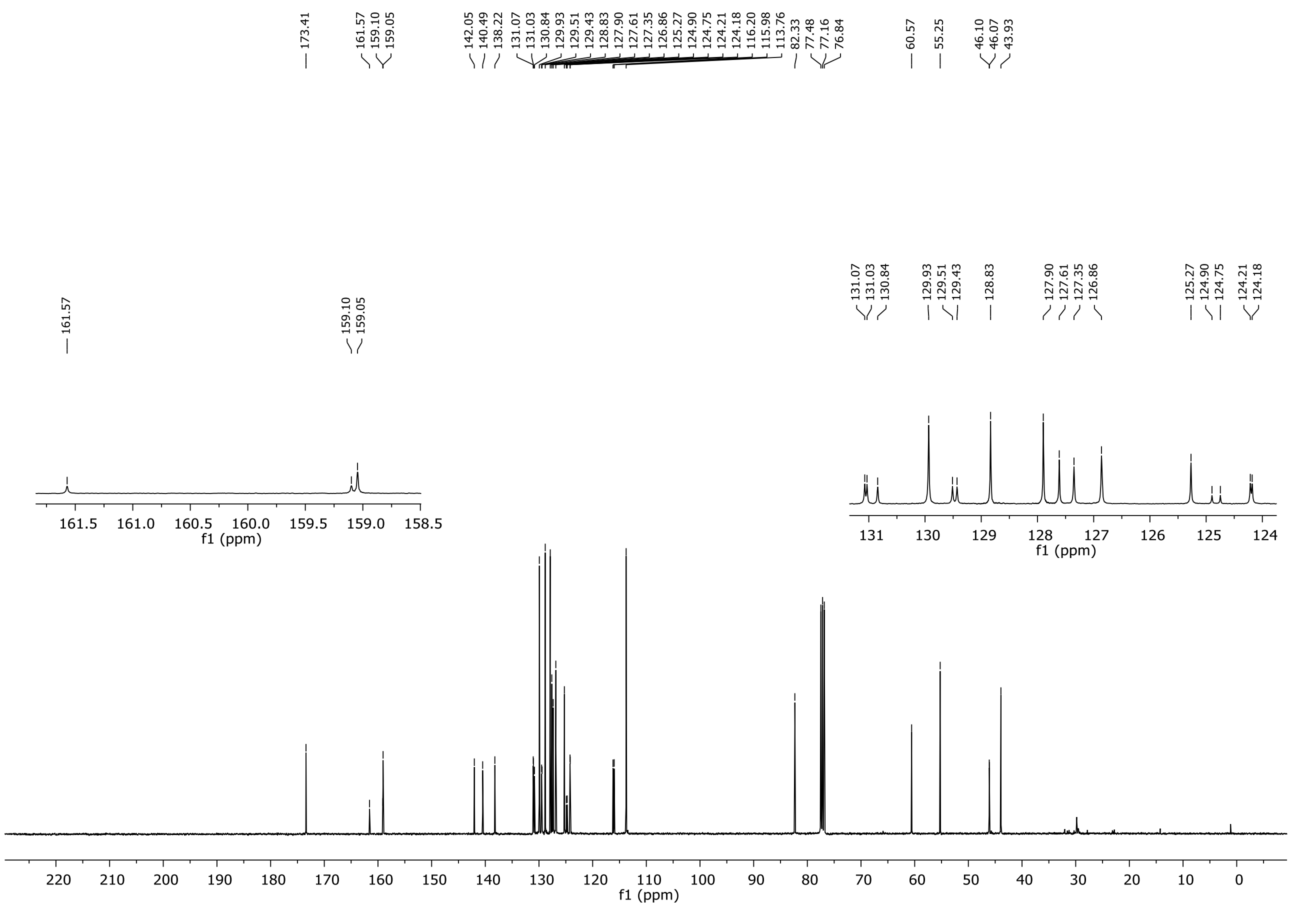

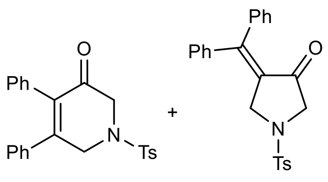

11 **12**

(27:1 mixture)

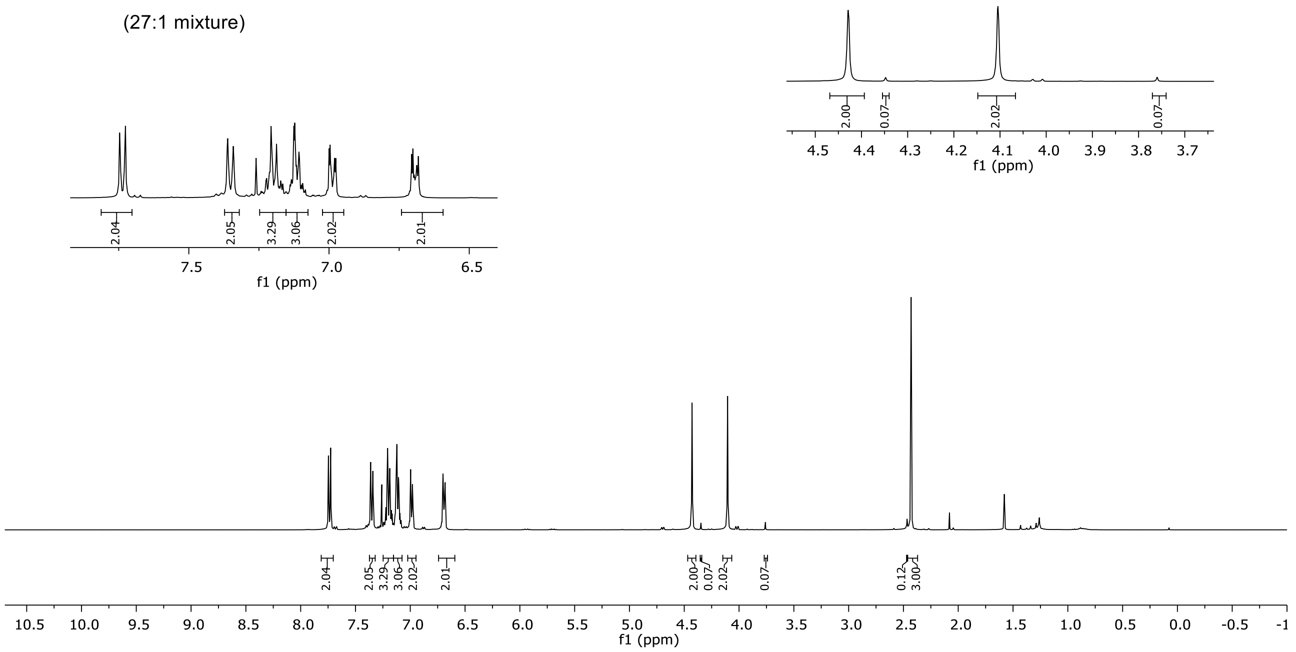

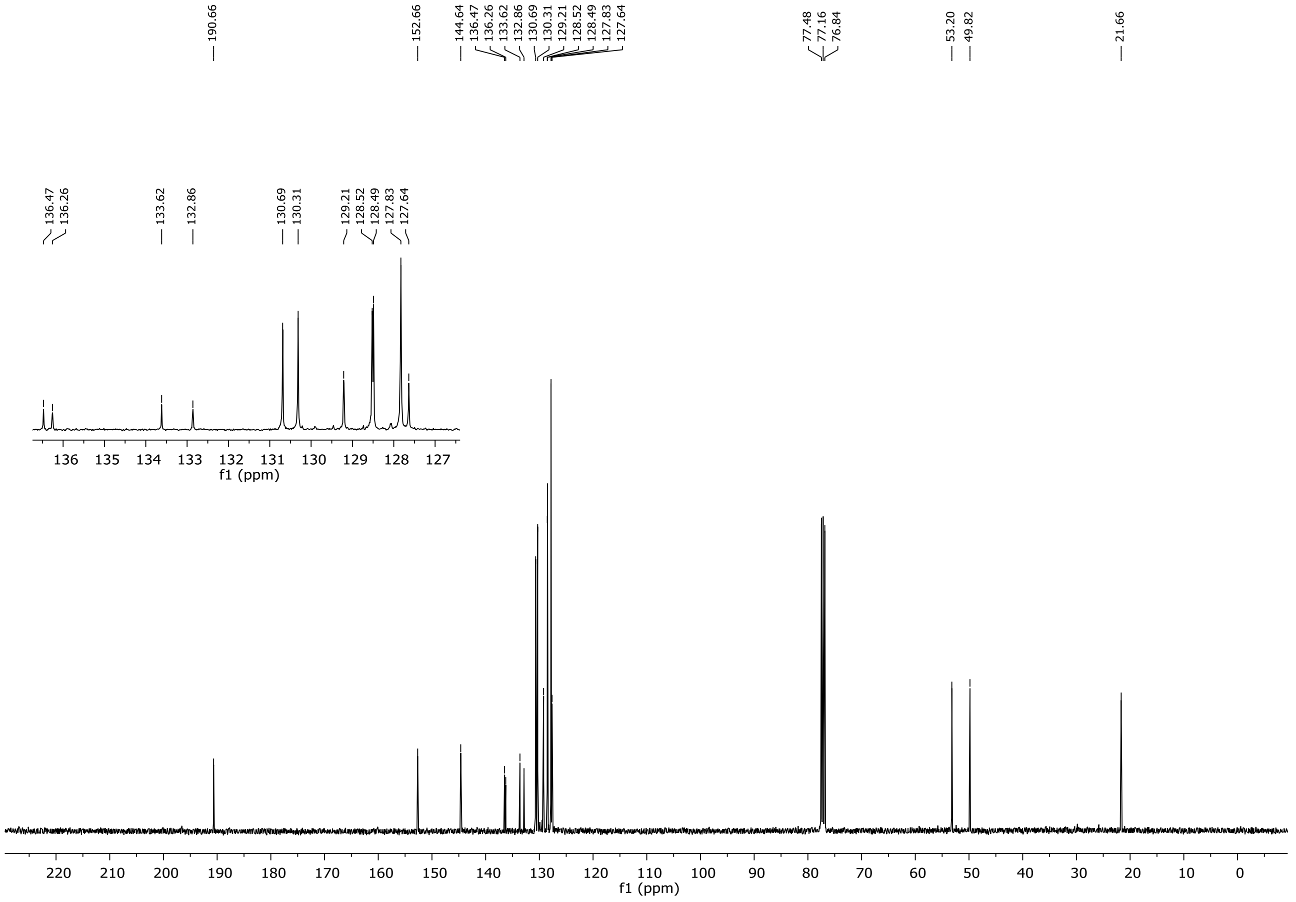

7. HPLC Traces

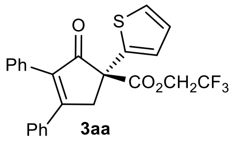

3aa

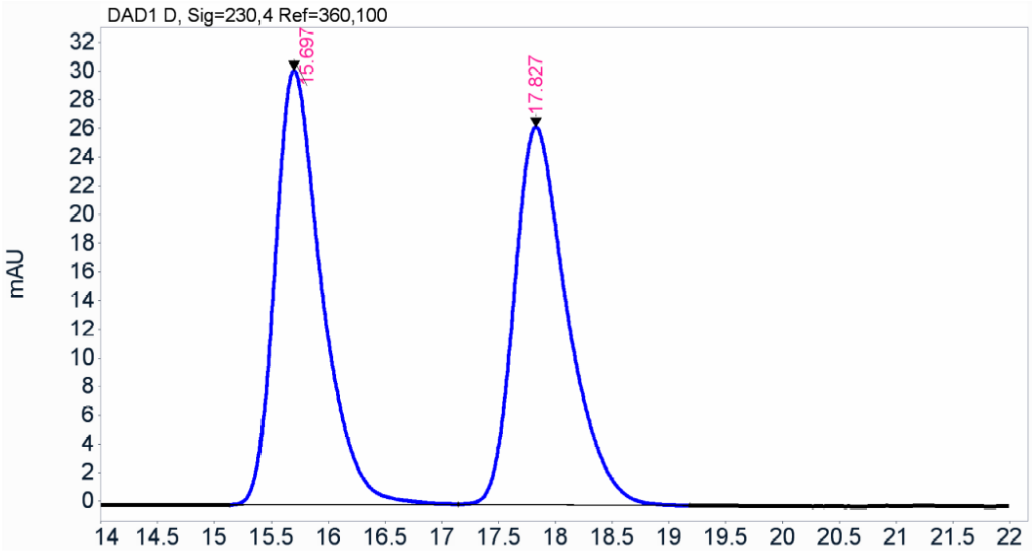

Signal: DAD1 D, Sig=230,4 Ref=360,100

RT [min]	Type	Width [min]	Area	Height	Area%
15.697	BB	0.4239	861.850	30.2982	50.19
17.827	BB	0.4868	855.494	26.3457	49.81

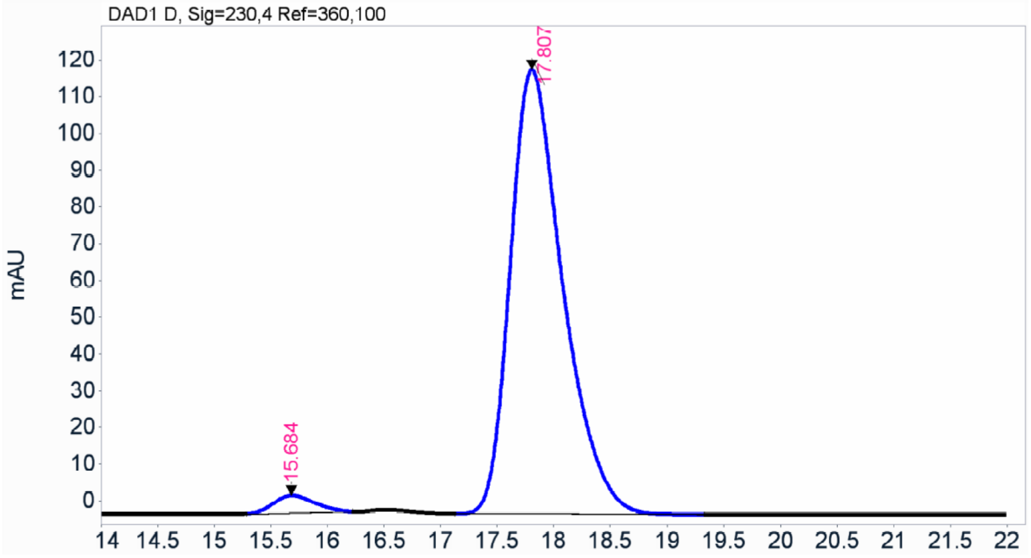

Signal: DAD1 D, Sig=230,4 Ref=360,100

RT [min]	Type	Width [min]	Area	Height	Area%
15.684	MM T	0.4235	124.801	4.9119	3.10
17.807	BB	0.4867	3903.760	120.9016	96.90

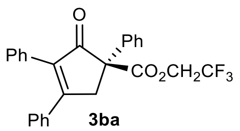

3ba

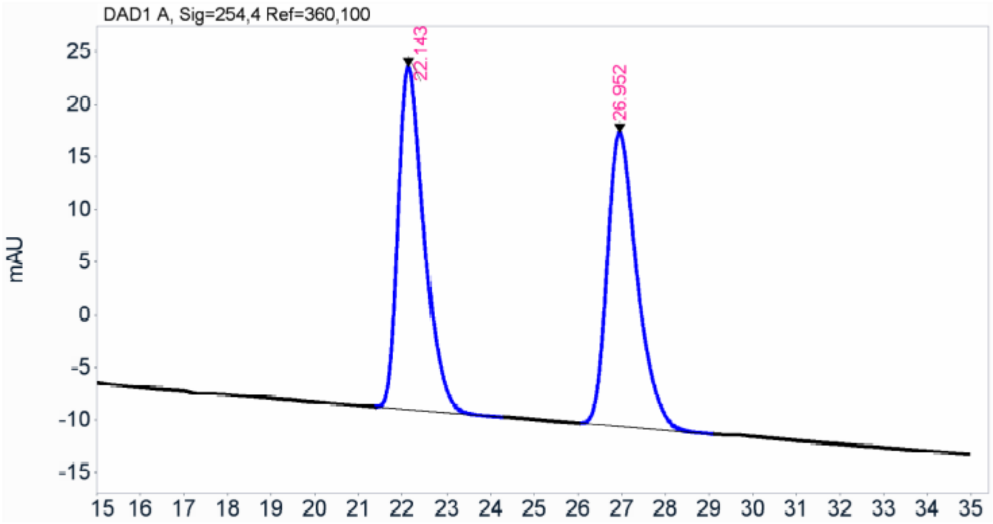

Signal:	DAD1 A, Sig=254,4 Ref=360,100				
RT [min]	Type	Width [min]	Area	Height	Area%
22.143	BB	0.6217	1334.022	32.5827	50.06
26.952	BB	0.7179	1330.905	27.9307	49.94

On a 0.30 mmol scale:

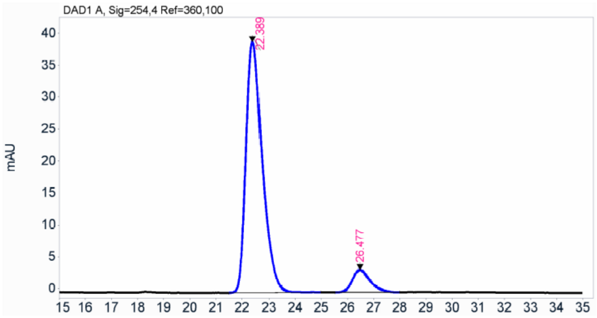

Signal:	DAD1 A, Sig=254,4 Ref=360,100				
RT [min]	Type	Width [min]	Area	Height	Area%
22.389	MM	0.7036	1659.120	39.2980	90.82
26.477	MM	0.7900	167.647	3.5370	9.18

On a 3.00 mmol scale:

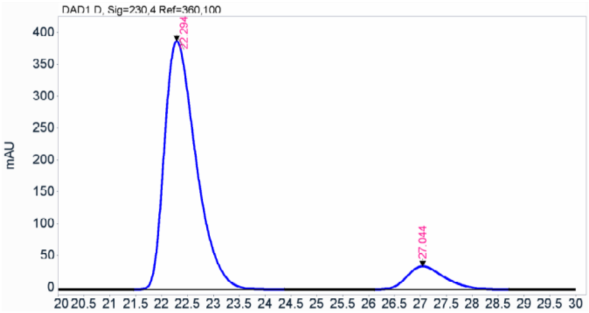

Signal:	DAD1 D, Sig=230,4 Ref=360,100				
RT [min]	Type	Width [min]	Area	Height	Area%
22.294	BB	0.6535	16502.934	390.1068	89.93
27.044	MM T	0.8481	1847.720	36.3114	10.07

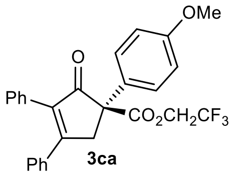

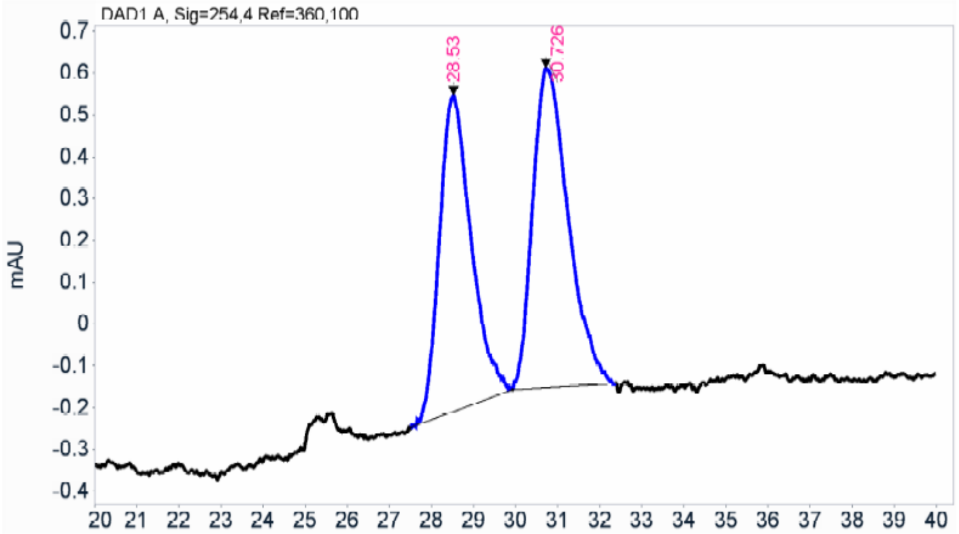

Signal: DAD1 A, Sig=254,4 Ref=360,100

RT [min]	Type	Width [min]	Area	Height	Area%
28.530	MM	0.8752	39.733	0.7566	46.95
30.726	MM	0.9782	44.894	0.7649	53.05

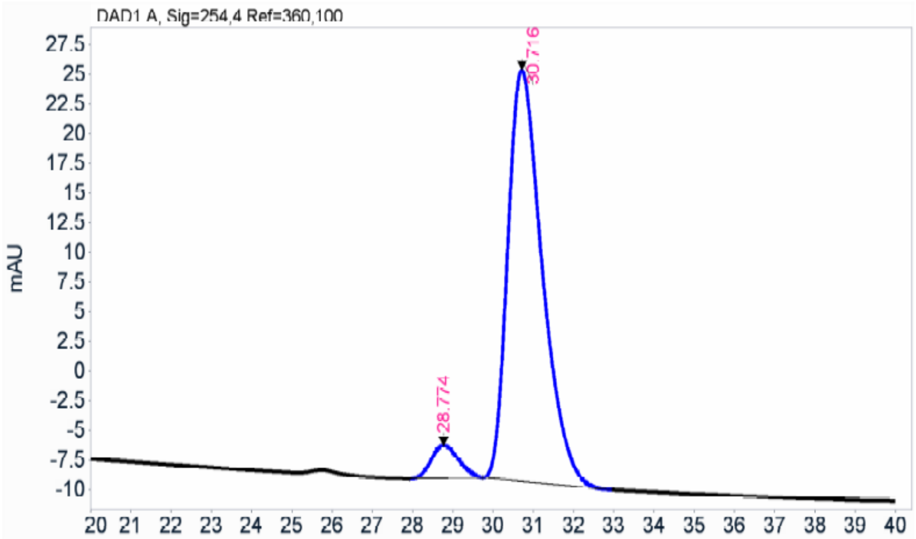

Signal: DAD1 A, Sig=254,4 Ref=360,100

RT [min]	Type	Width [min]	Area	Height	Area%
28.774	MM	0.7879	130.344	2.7573	5.95
30.716	BB	0.8975	2059.277	34.6443	94.05

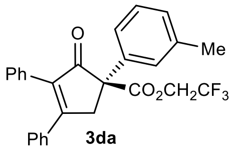

3da

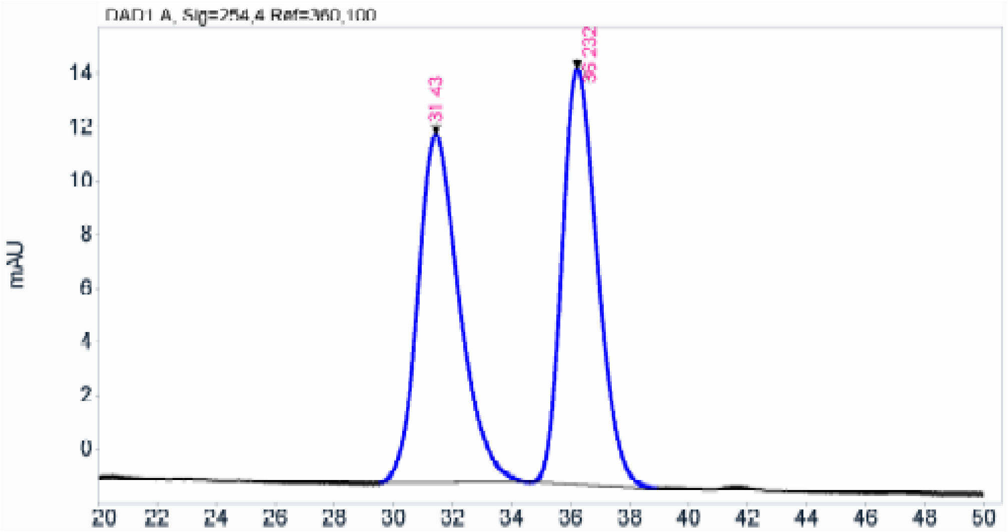

Signal:	DAD1 A, Sig=254,4 Ref=360,100				
RT [min]	Type	Width [min]	Area	Height	Area%
31.430	BB	1.3251	1266.616	12.9503	49.64
36.232	BB	1.1981	1284.836	15.4841	50.36

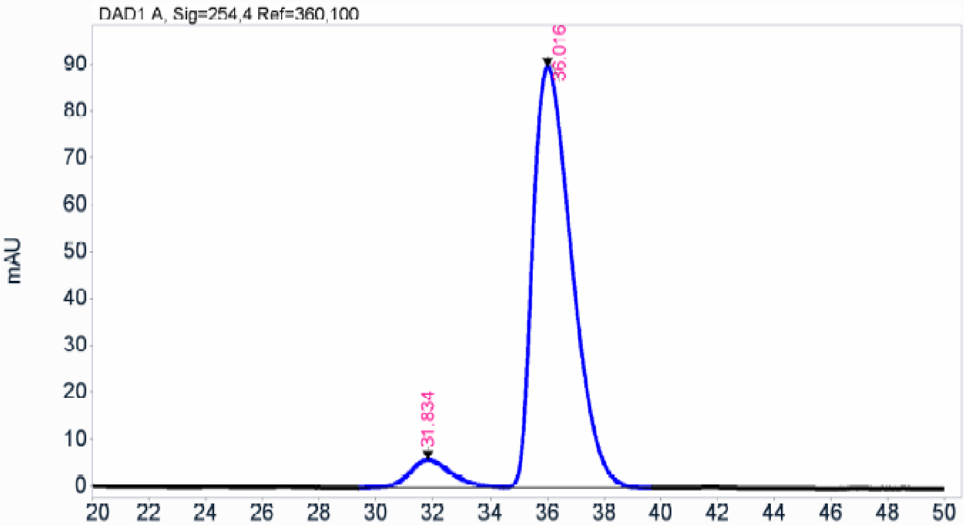

Signal:	DAD1 A, Sig=254,4 Ref=360,100				
RT [min]	Type	Width [min]	Area	Height	Area%
31.834	MM	1.6817	591.171	5.8590	6.49
36.016	MM	1.5798	8514.479	89.8278	93.51

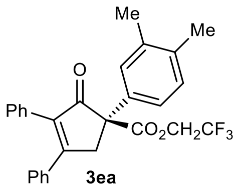

3ea

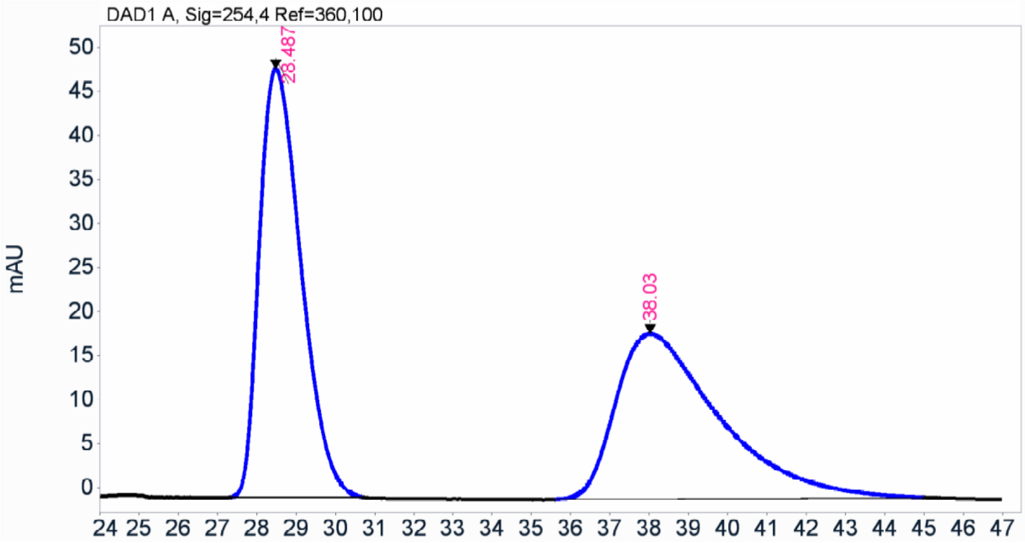

Signal: DAD1 A, Sig=254,4 Ref=360,100

RT [min]	Type	Width [min]	Area	Height	Area%
28.487	MM T	1.2235	3570.854	48.6417	51.08
38.030	BB	2.1453	3419.324	18.7351	48.92

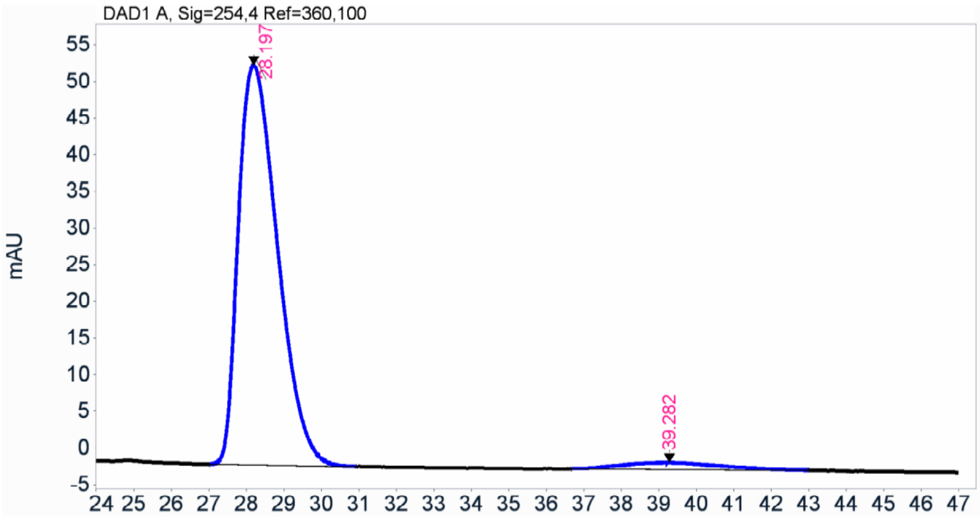

Signal: DAD1 A, Sig=254,4 Ref=360,100

RT [min]	Type	Width [min]	Area	Height	Area%
28.197	BB	1.1141	3972.237	54.5153	96.00
39.282	MM T	3.0472	165.486	0.9051	4.00

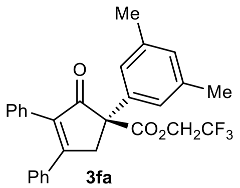

3fa

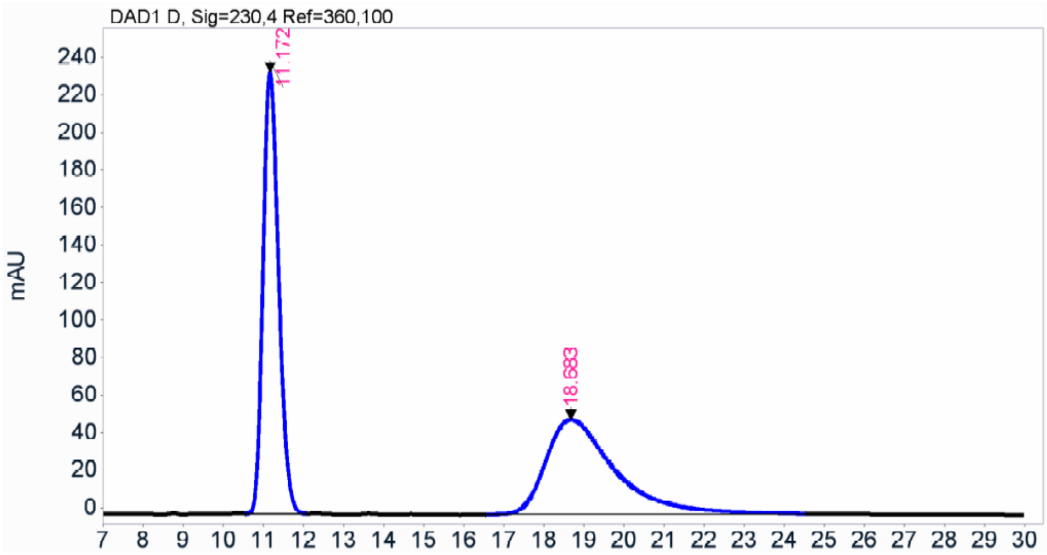

Signal: DAD1 D, Sig=230,4 Ref=360,100

RT [min]	Type	Width [min]	Area	Height	Area%
11.172	BB	0.4207	6345.623	235.2628	51.27
18.683	BB	1.7090	6030.103	50.4312	48.73

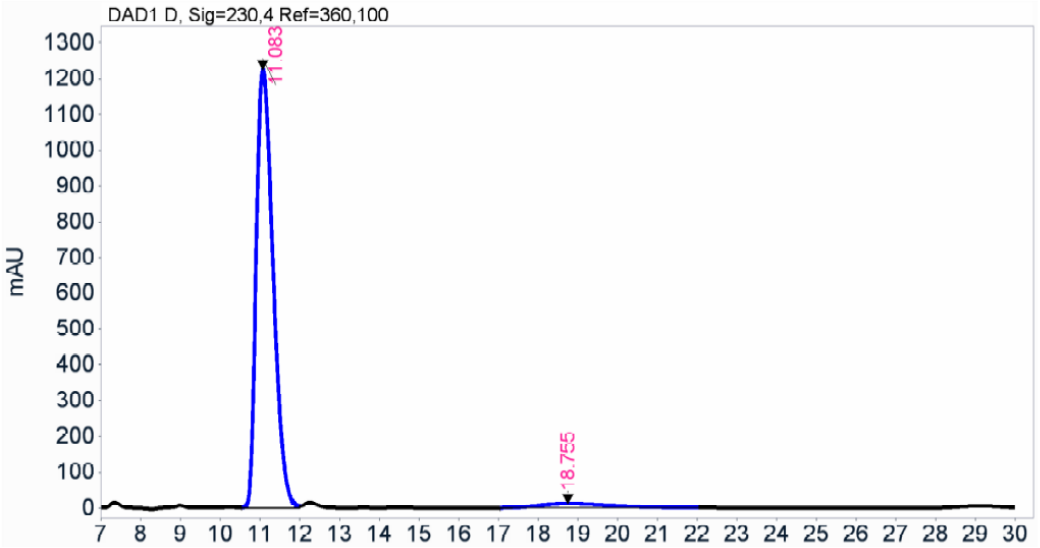

Signal: DAD1 D, Sig=230,4 Ref=360,100

RT [min]	Type	Width [min]	Area	Height	Area%
11.083	VV	0.4628	35984.730	1224.7623	96.00
18.755	MM	2.1252	1501.127	11.7726	4.00

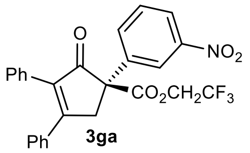

3ga

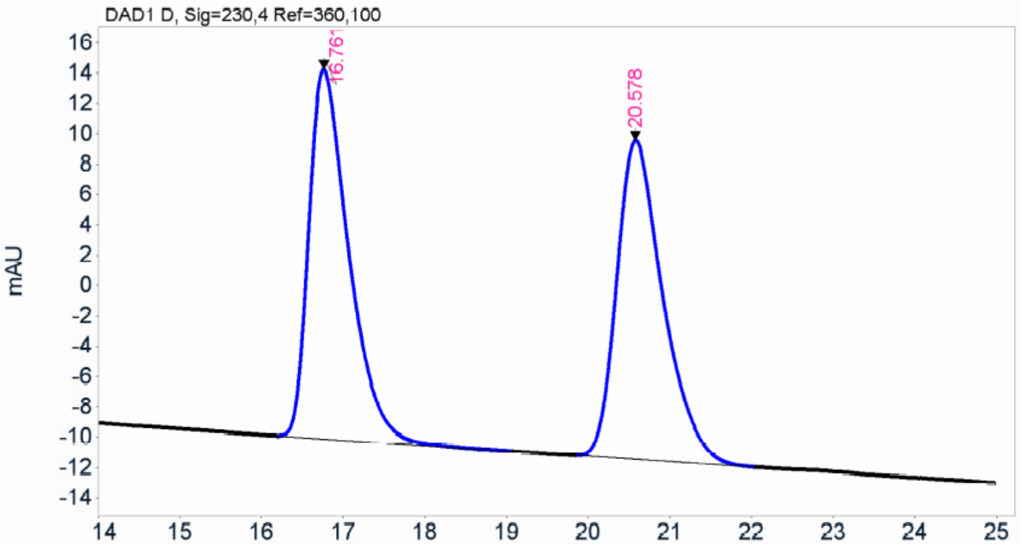

Signal:	DAD1 D, Sig=230,4 Ref=360,100				
RT [min]	Type	Width [min]	Area	Height	Area%
16.761	BB	0.5105	827.025	24.3331	50.04
20.578	BBA	0.5846	825.685	21.0055	49.96

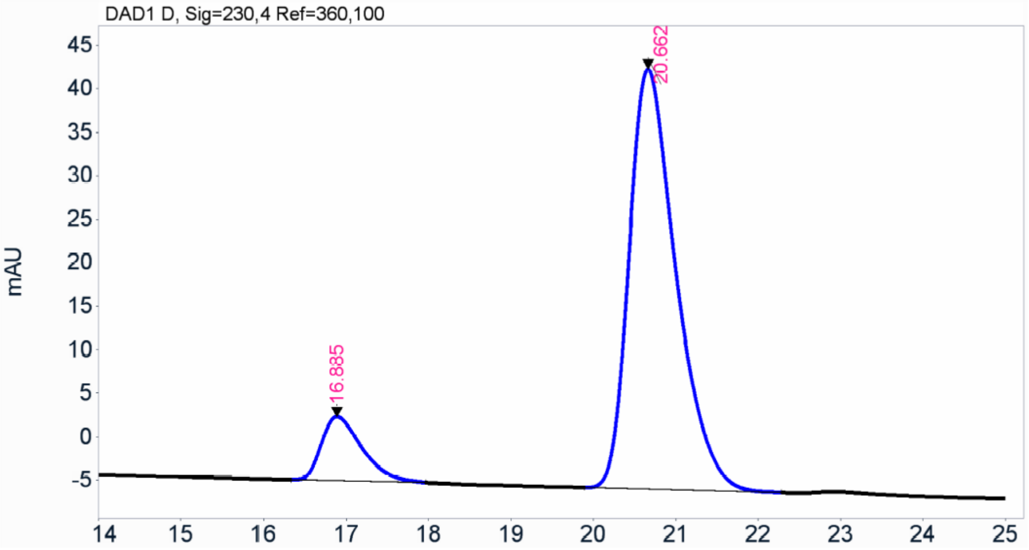

Signal:	DAD1 D, Sig=230,4 Ref=360,100				
RT [min]	Type	Width [min]	Area	Height	Area%
16.885	MM T	0.5691	251.301	7.3601	11.64
20.662	BB	0.5990	1907.546	48.2785	88.36

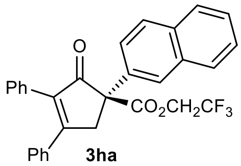

3ha

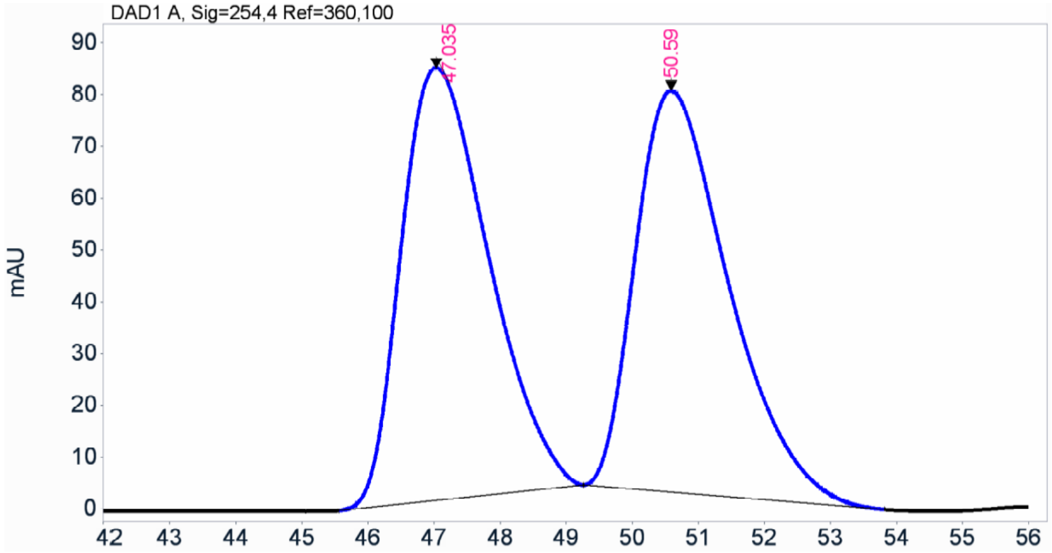

Signal:	DAD1 A, Sig=254,4 Ref=360,100				
RT [min]	Type	Width [min]	Area	Height	Area%
47.035	BB	1.4006	7674.342	83.4730	50.17
50.590	BB	1.4763	7622.403	77.4430	49.83

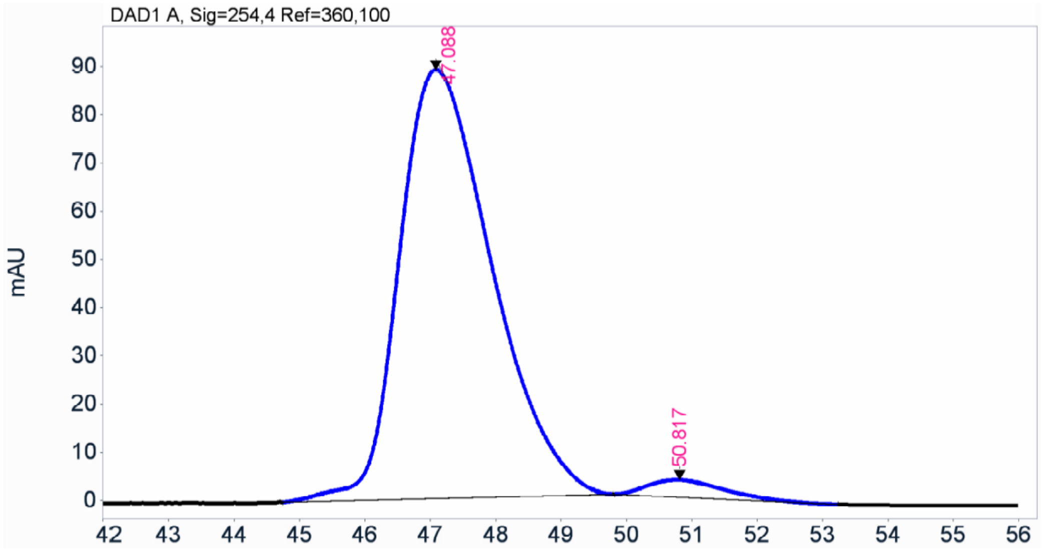

Signal:	DAD1 A, Sig=254,4 Ref=360,100				
RT [min]	Type	Width [min]	Area	Height	Area%
47.088	BB	1.4090	8737.271	88.8725	96.77
50.817	MM T	1.3497	291.567	3.6005	3.23

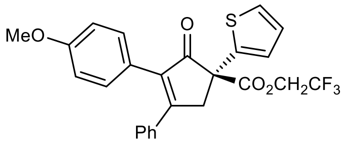

3ia

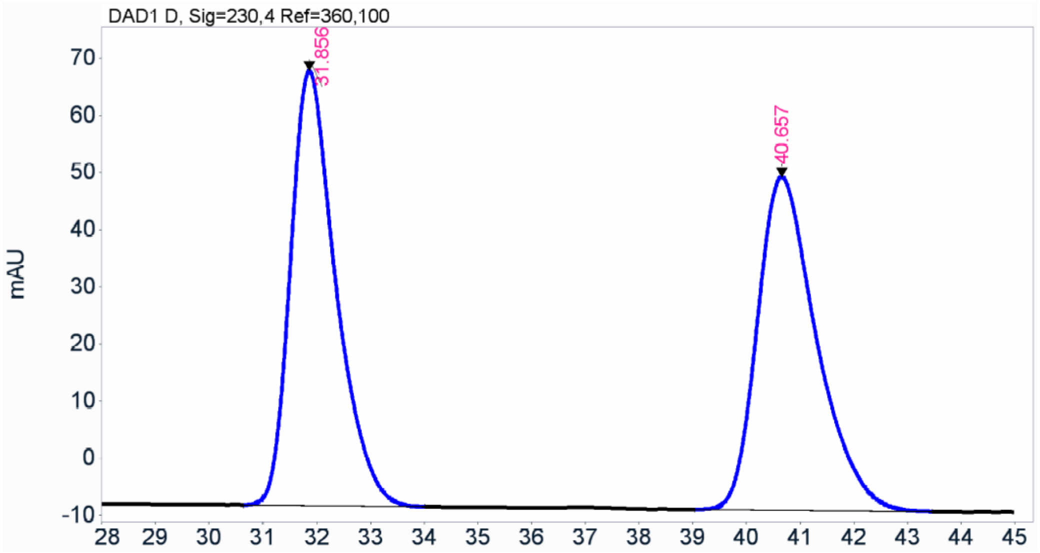

Signal: DAD1 D, Sig=230,4 Ref=360,100

RT [min]	Type	Width [min]	Area	Height	Area%
31.856	BB	0.8821	4472.105	76.0447	49.94
40.657	BB	1.1408	4482.116	58.3089	50.06

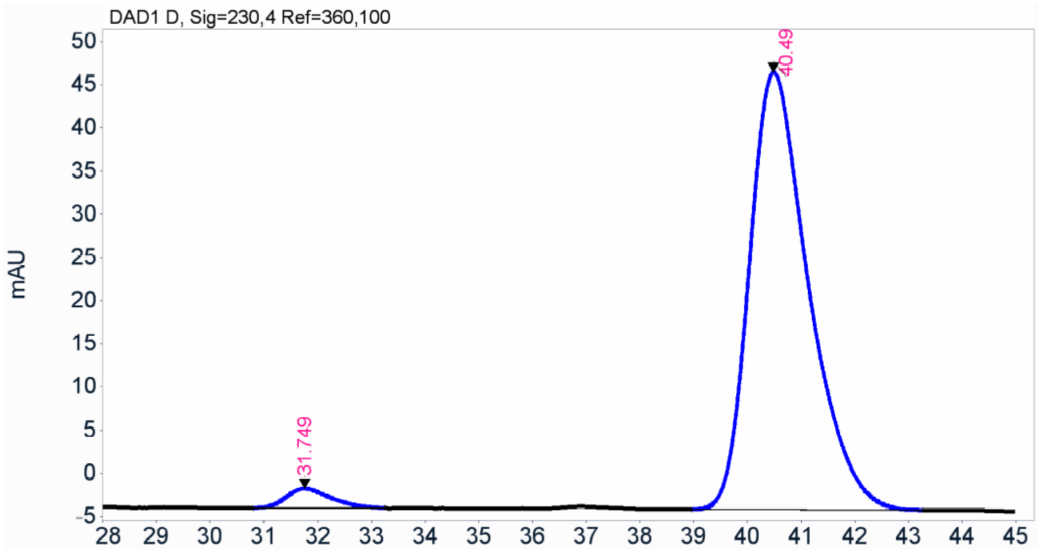

Signal: DAD1 D, Sig=230,4 Ref=360,100

RT [min]	Type	Width [min]	Area	Height	Area%
31.749	MM T	0.9434	128.589	2.2717	3.22
40.490	MM T	1.4709	3863.515	50.6702	96.78

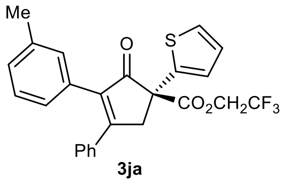

3ja

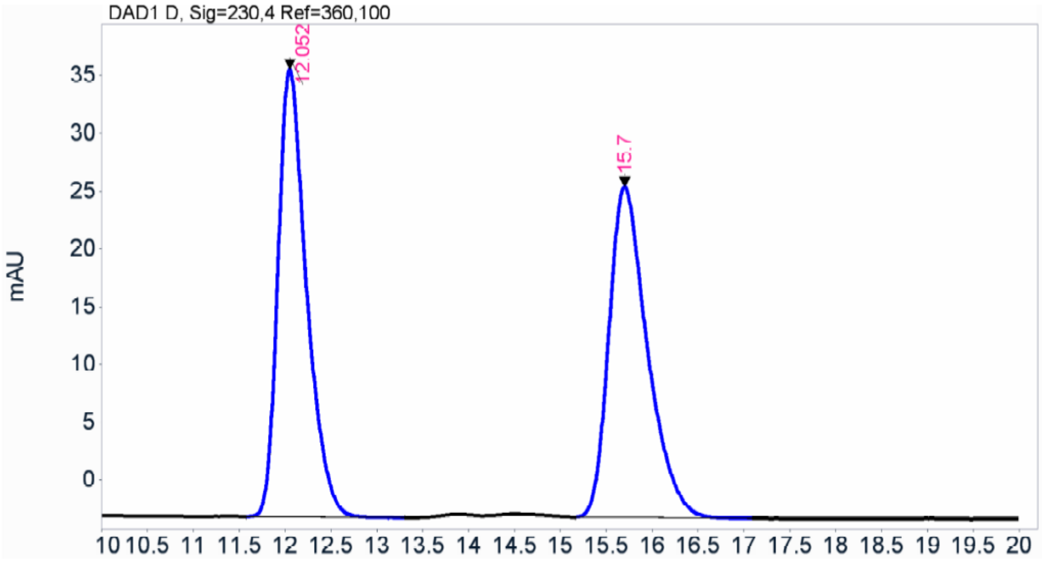

Signal:	DAD1 D, Sig=230,4 Ref=360,100				
RT [min]	Type	Width [min]	Area	Height	Area%
12.052	BB	0.3221	837.867	38.7715	50.18
15.700	BB	0.4394	831.792	28.5948	49.82

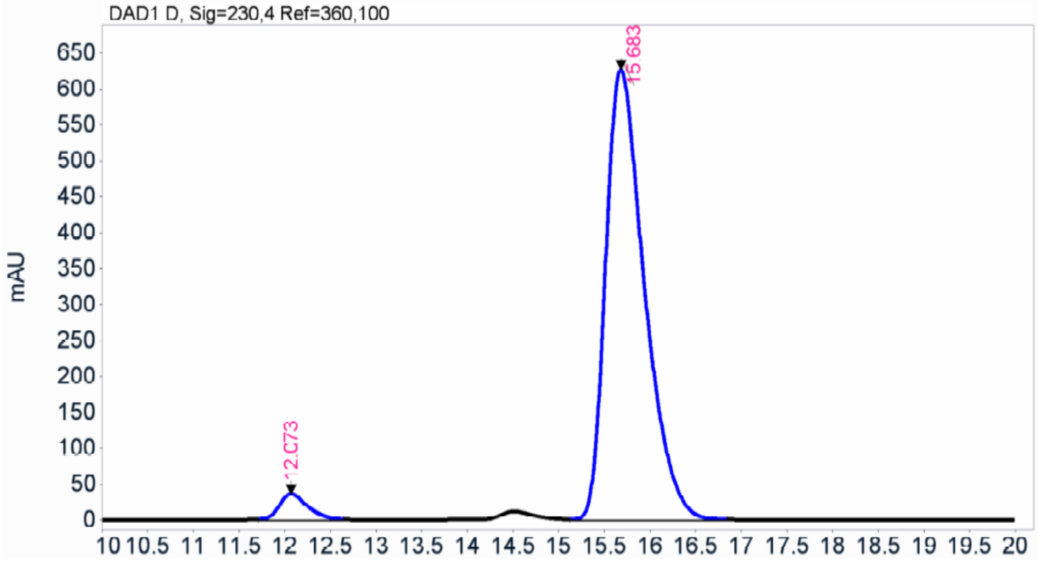

Signal:	DAD1 D, Sig=230,4 Ref=360,100				
RT [min]	Type	Width [min]	Area	Height	Area%
12.073	MM	0.3793	840.647	36.9376	4.33
15.683	MM	0.4933	18575.090	627.5728	95.67

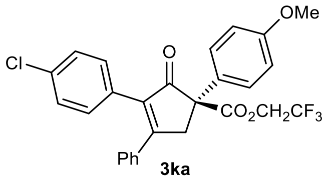

3ka

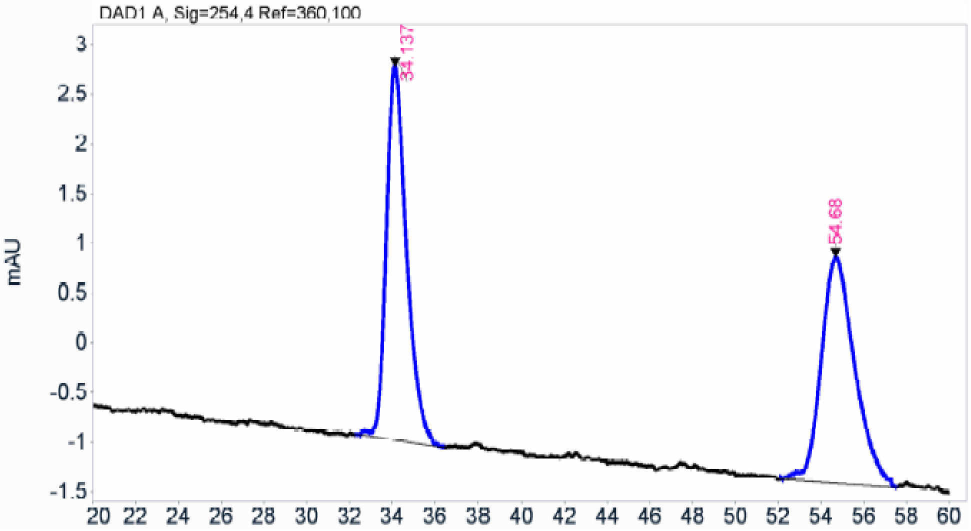

Signal:	DAD1 A, Sig=254,4 Ref=360,100				
RT [min]	Type	Width [min]	Area	Height	Area%
34.137	MM	1.0987	247.909	3.7607	49.97
54.680	MM	1.8233	248.189	2.2686	50.03

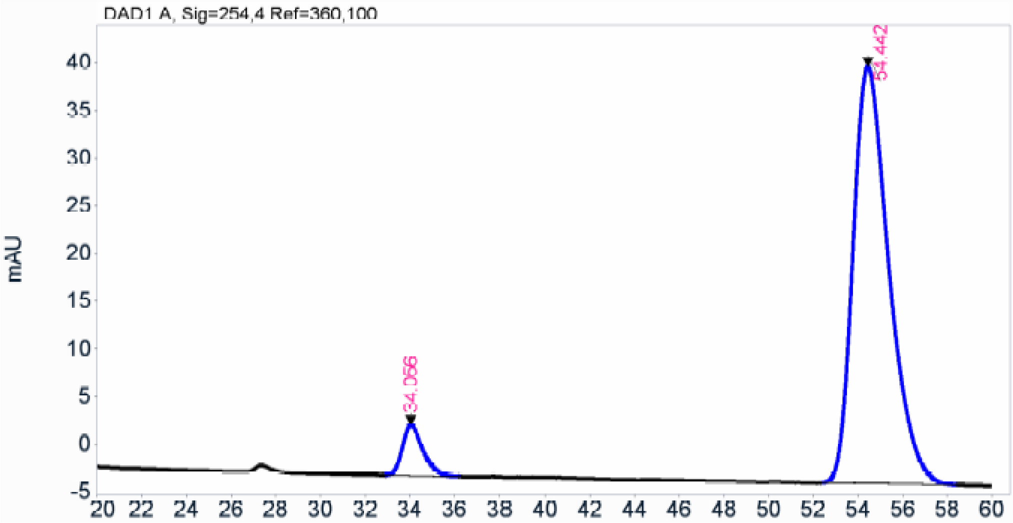

Signal:	DAD1 A, Sig=254,4 Ref=360,100				
RT [min]	Type	Width [min]	Area	Height	Area%
34.056	BB	0.9248	343.123	5.3035	6.61
54.442	BB	1.6342	4844.918	43.7430	93.39

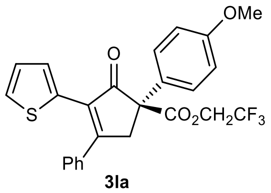

3la

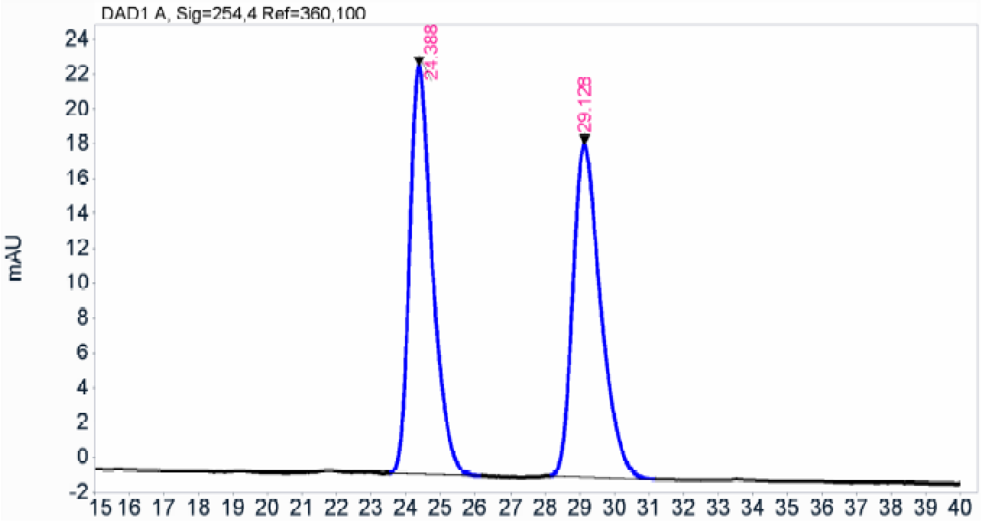

Signal:	DAD1 A, Sig=254,4 Ref=360,100				
RT [min]	Type	Width [min]	Area	Height	Area%
24.388	BB	0.6892	1071.255	23.4400	50.02
29.128	BB	0.8506	1070.315	19.0748	49.98

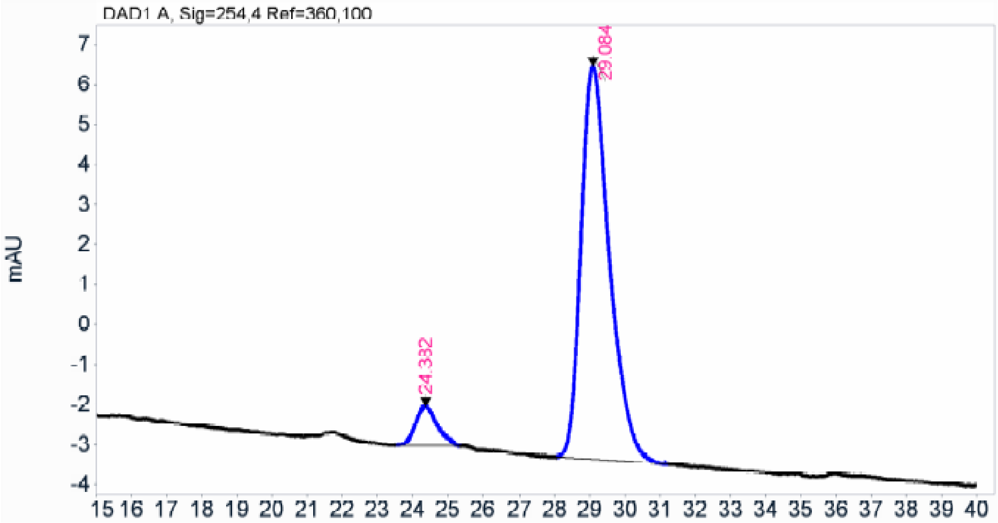

Signal:	DAD1 A, Sig=254,4 Ref=360,100				
RT [min]	Type	Width [min]	Area	Height	Area%
24.382	MM	0.7233	42.141	0.9710	7.10
29.084	BB	0.8272	551.749	9.8240	92.90

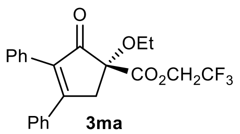

3ma

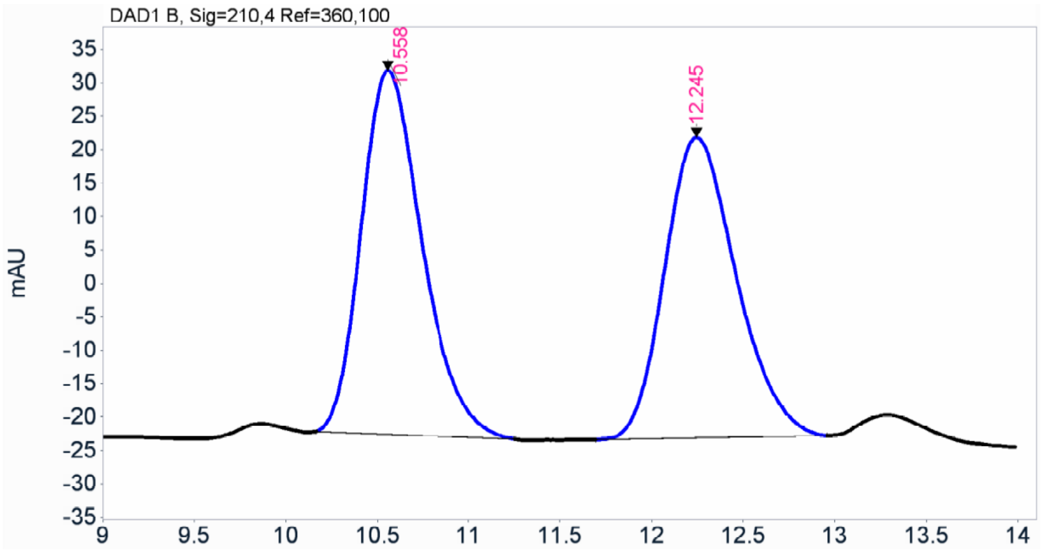

**Signal:**	DAD1 B, Sig=210,4 Ref=360,100				
**RT [min]**	**Type**	**Width [min]**	**Area**	**Height**	**Area%**
10.558	MM T	0.3870	1264.368	54.4478	50.93
12.245	BB	0.4183	1218.156	44.9315	49.07

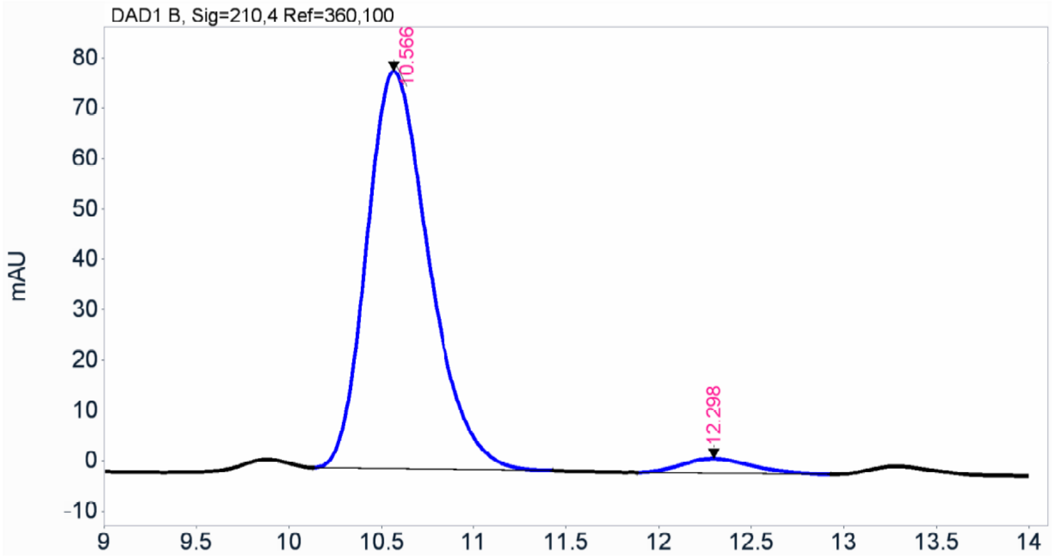

**Signal:**	DAD1 B, Sig=210,4 Ref=360,100				
**RT [min]**	**Type**	**Width [min]**	**Area**	**Height**	**Area%**
10.566	MM T	0.3909	1851.000	78.9208	96.00
12.298	BB	0.3287	77.184	2.8981	4.00

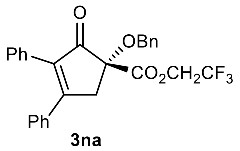

3na

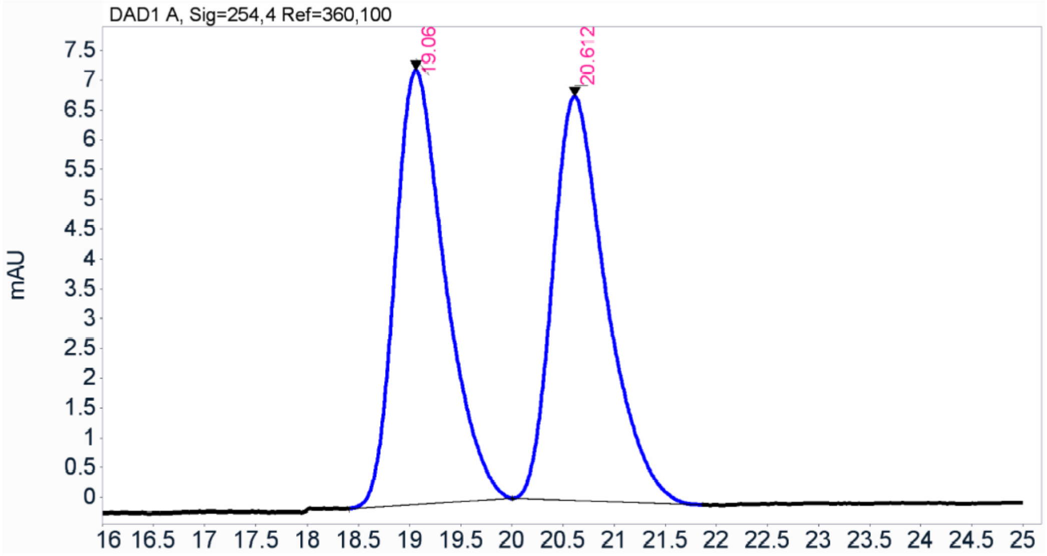

Signal: DAD1 A, Sig=254,4 Ref=360,100

RT [min]	Type	Width [min]	Area	Height	Area%
19.060	BB	0.5069	247.952	7.2887	49.97
20.612	BB	0.5432	248.279	6.7810	50.03

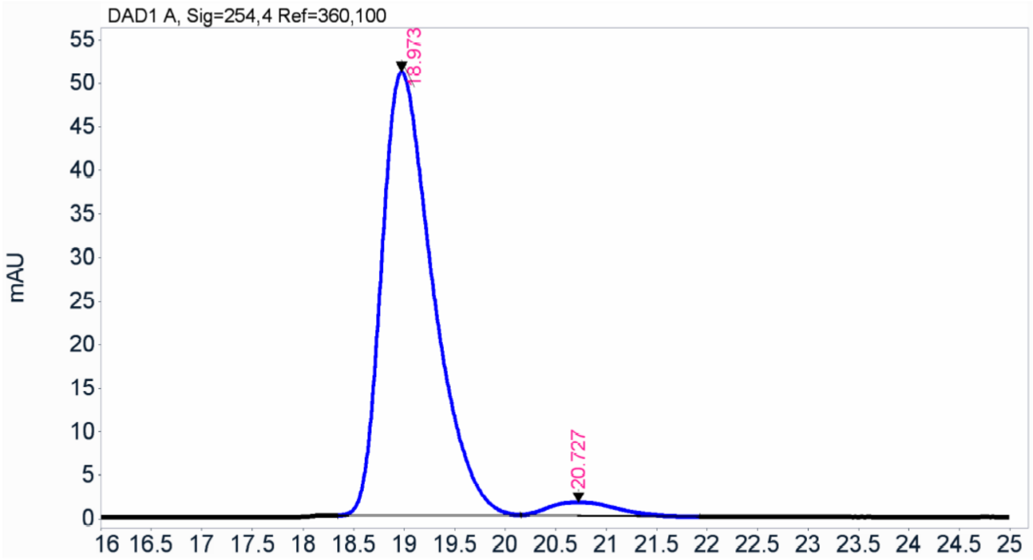

Signal: DAD1 A, Sig=254,4 Ref=360,100

RT [min]	Type	Width [min]	Area	Height	Area%
18.973	BB	0.5342	1789.724	50.8981	96.32
20.727	MM T	0.7530	68.469	1.5154	3.68

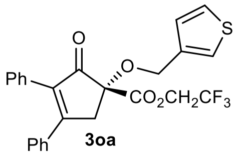

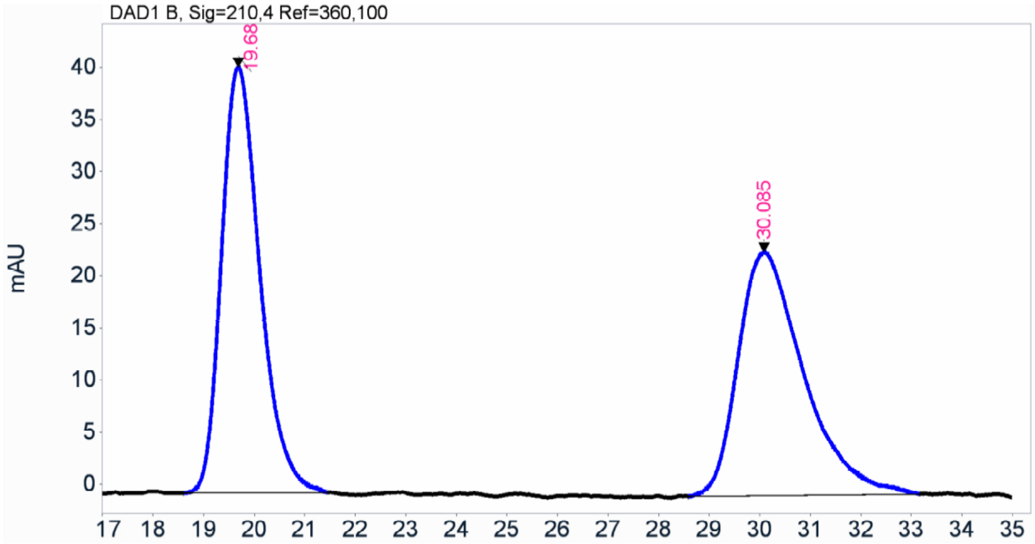

Signal: DAD1 B, Sig=210,4 Ref=360,100

RT [min]	Type	Width [min]	Area	Height	Area%
19.680	BB	0.7700	2156.098	40.8321	50.68
30.085	BB	1.0658	2098.460	23.3202	49.32

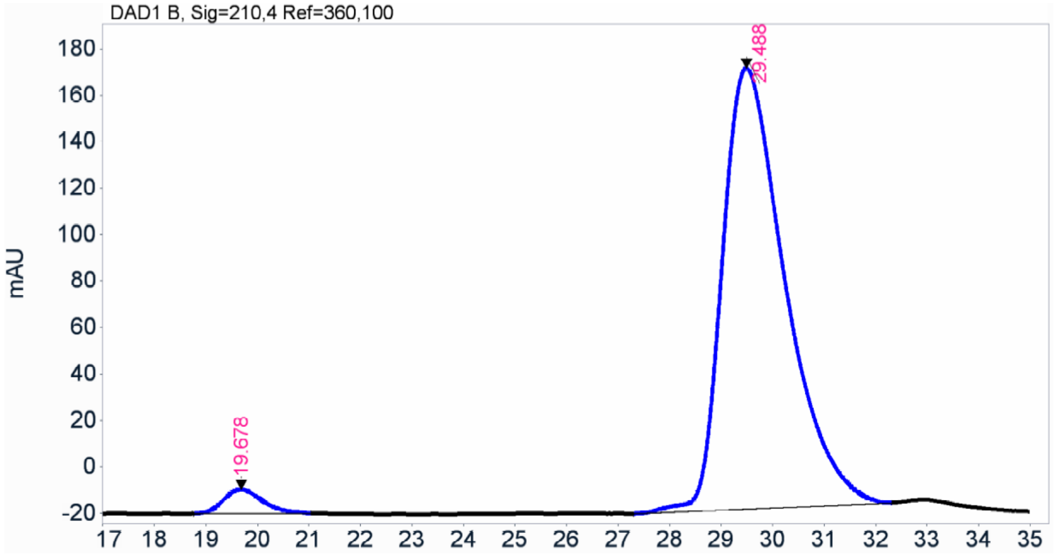

Signal: DAD1 B, Sig=210,4 Ref=360,100

RT [min]	Type	Width [min]	Area	Height	Area%
19.678	BB	0.6795	550.825	10.4412	3.33
29.488	BB	1.2474	15999.565	189.8048	96.67

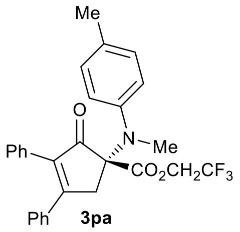

3pa

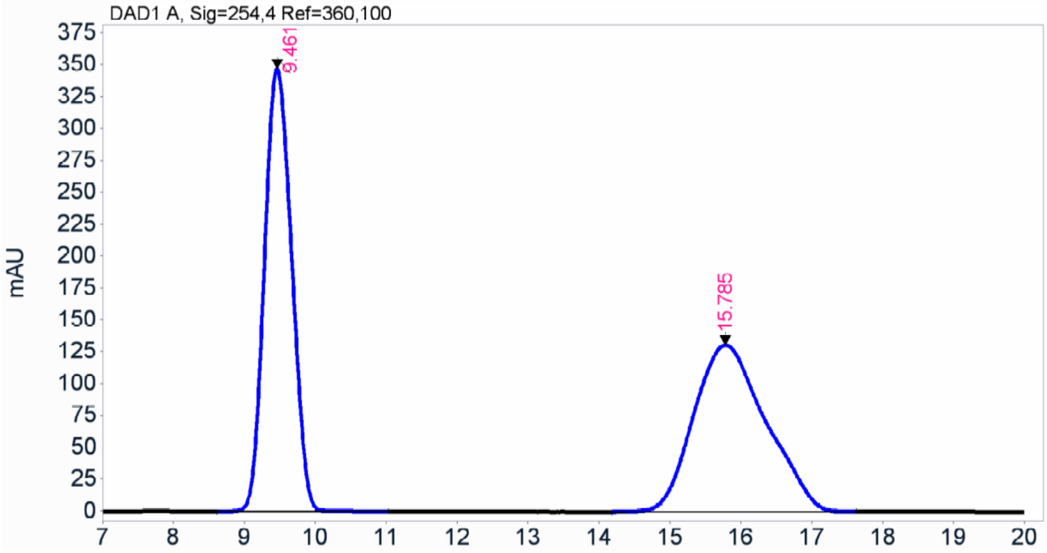

**Signal:**	DAD1 A, Sig=254,4 Ref=360,100				
**RT [min]**	**Type**	**Width [min]**	**Area**	**Height**	**Area%**
9.461	BB	0.4288	9252.585	347.3269	50.04
15.785	BB	1.0805	9237.588	130.7179	49.96

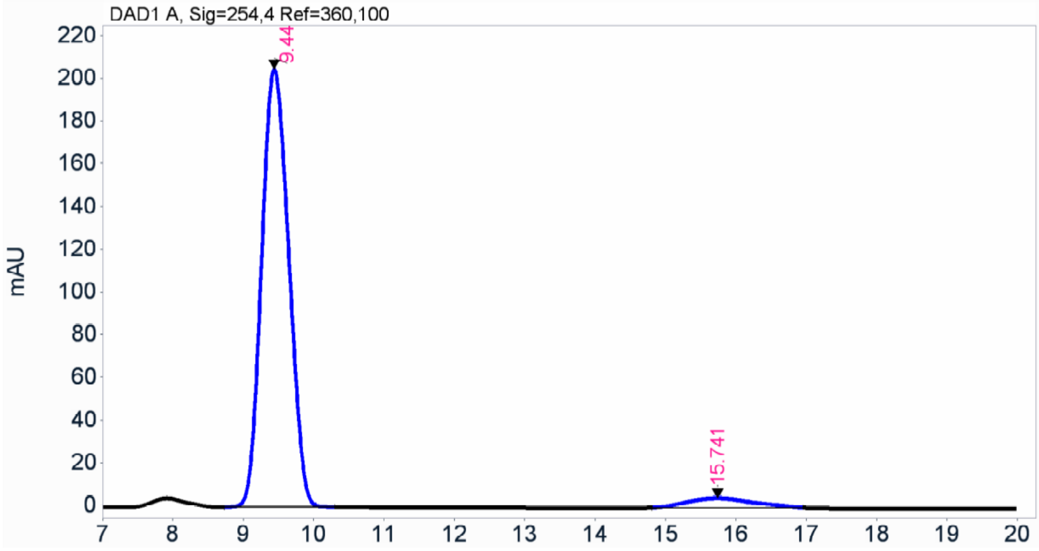

**Signal:**	DAD1 A, Sig=254,4 Ref=360,100				
**RT [min]**	**Type**	**Width [min]**	**Area**	**Height**	**Area%**
9.440	BB	0.4295	5474.698	205.0374	94.85
15.741	MM T	1.1090	297.383	4.4692	5.15

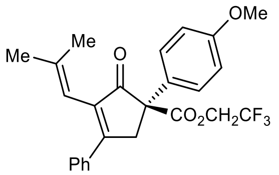

7

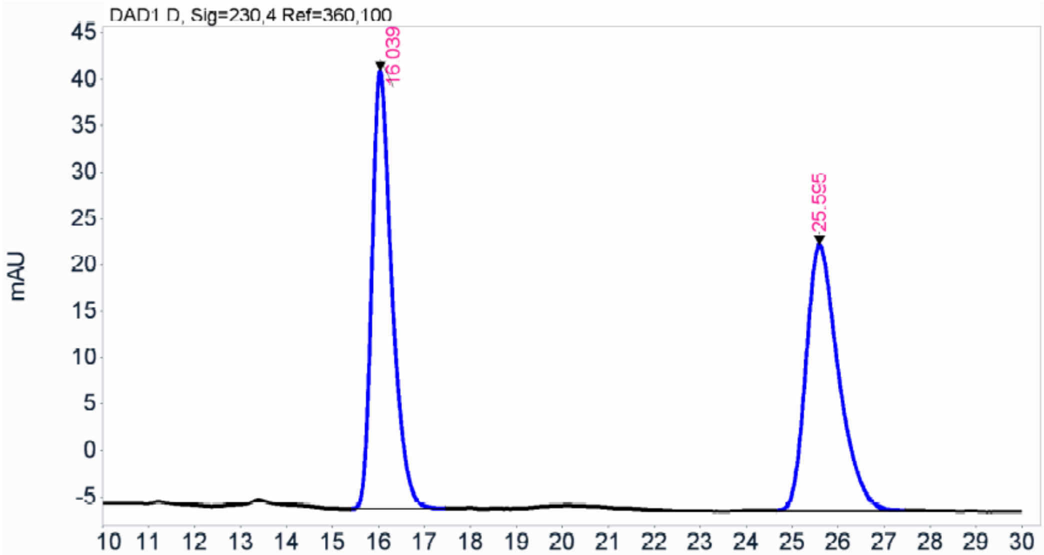

Signal: DAD1 D, Sig=230,4 Ref=360,100

RT [min]	Type	Width [min]	Area	Height	Area%
16.039	BB	0.4546	1426.477	47.1995	50.35
25.595	BB	0.7356	1406.880	28.6157	49.65

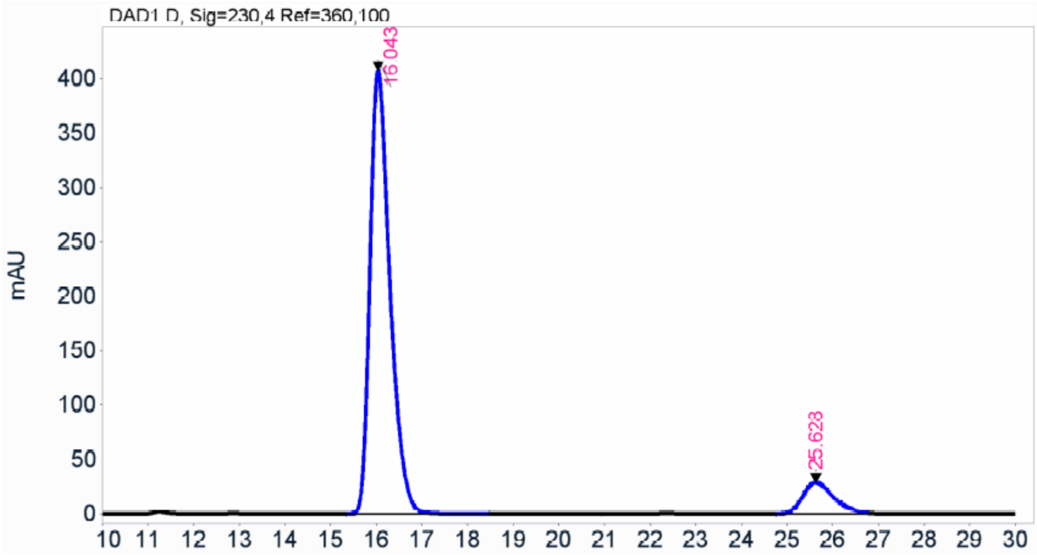

Signal: DAD1 D, Sig=230,4 Ref=360,100

RT [min]	Type	Width [min]	Area	Height	Area%
16.043	BB	0.4588	12400.387	407.7034	89.93
25.628	MM T	0.8062	1388.450	28.7048	10.07

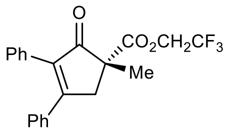

ent-**3qa**

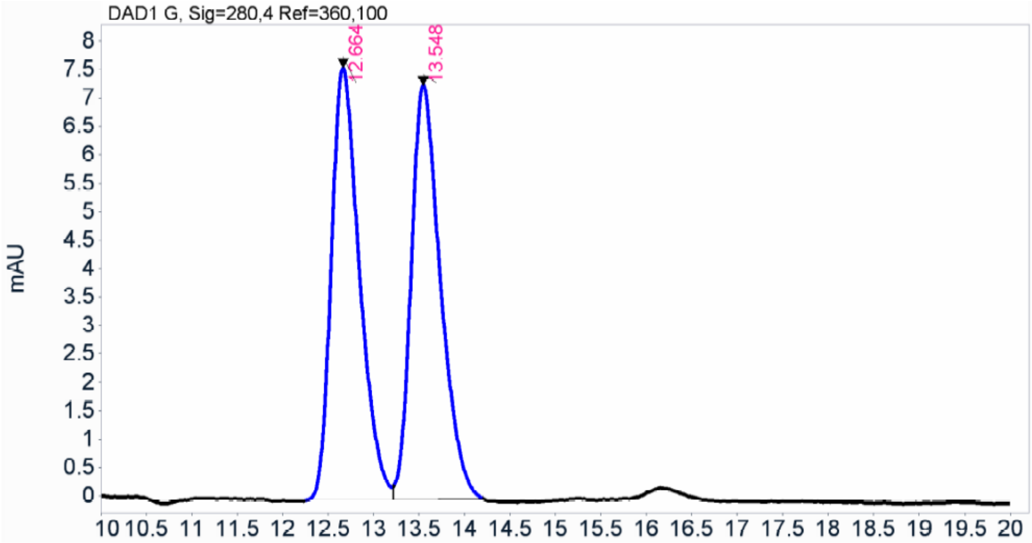

**Signal:**	DAD1 G, Sig=280,4 Ref=360,100				
**RT [min]**	**Type**	**Width [min]**	**Area**	**Height**	**Area%**
12.664	MF	0.3597	164.127	7.6042	50.15
13.548	FM	0.3735	163.118	7.2786	49.85

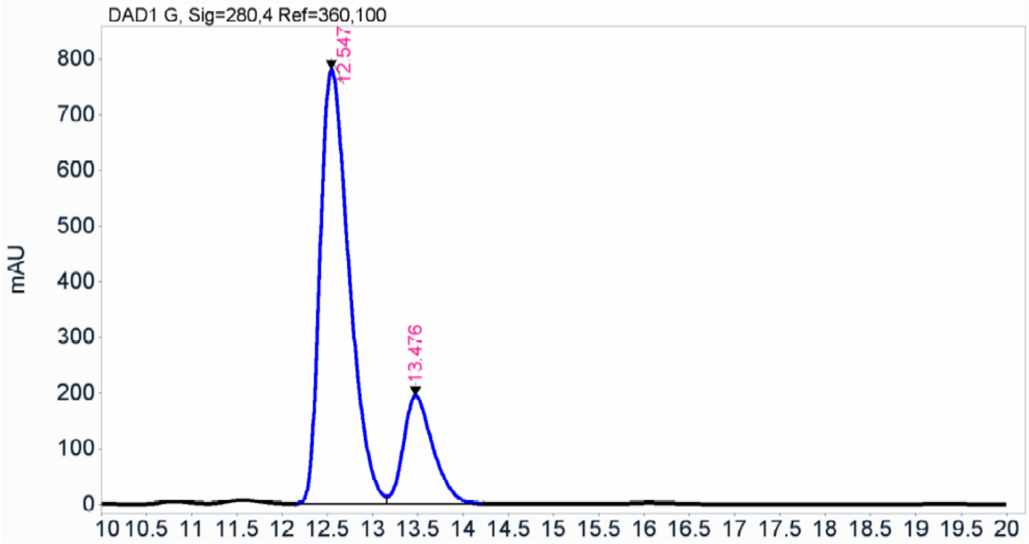

**Signal:**	DAD1 G, Sig=280,4 Ref=360,100				
**RT [min]**	**Type**	**Width [min]**	**Area**	**Height**	**Area%**
12.547	MF	0.3715	17402.414	780.7458	79.63
13.476	FM	0.3812	4452.681	194.6742	20.37

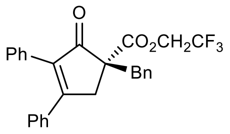

ent-**3ra**

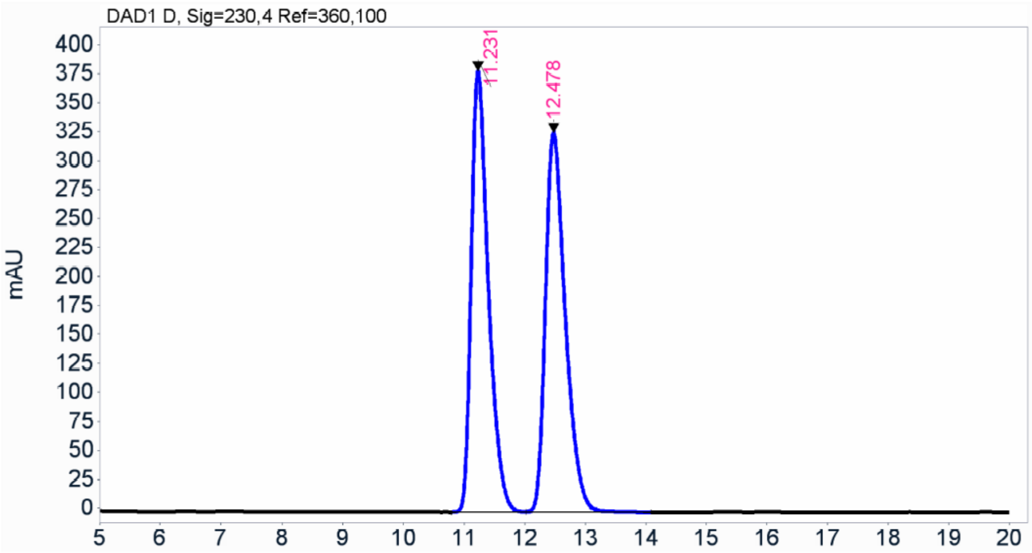

Signal:	DAD1 D, Sig=230,4 Ref=360,100				
RT [min]	Type	Width [min]	Area	Height	Area%
11.231	BB	0.2928	7461.796	380.4515	49.95
12.478	BB	0.3425	7477.133	327.2975	50.05

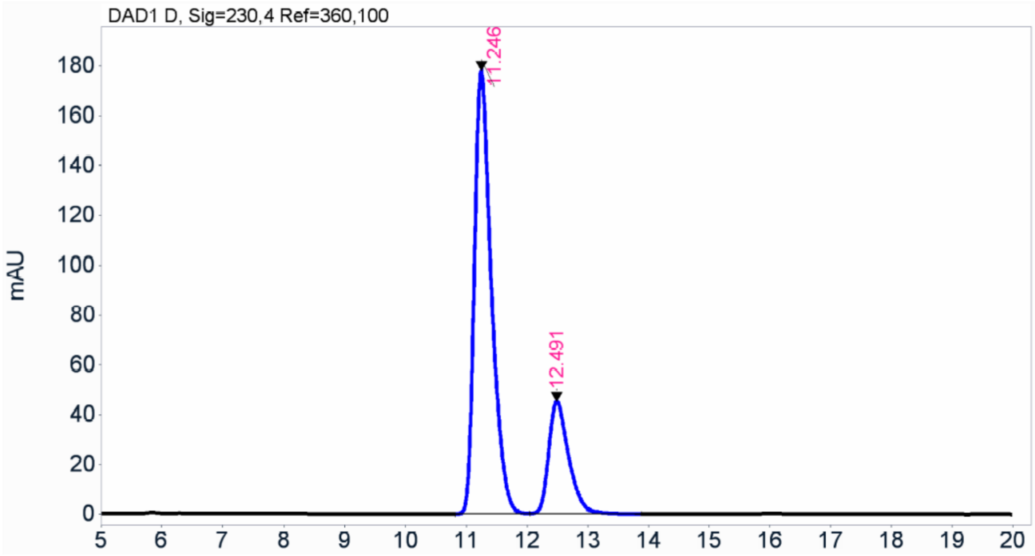

Signal:	DAD1 D, Sig=230,4 Ref=360,100				
RT [min]	Type	Width [min]	Area	Height	Area%
11.246	BB	0.2908	3458.887	177.9083	76.97
12.491	BB	0.3454	1035.050	45.1575	23.03

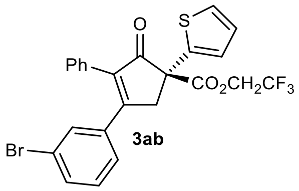

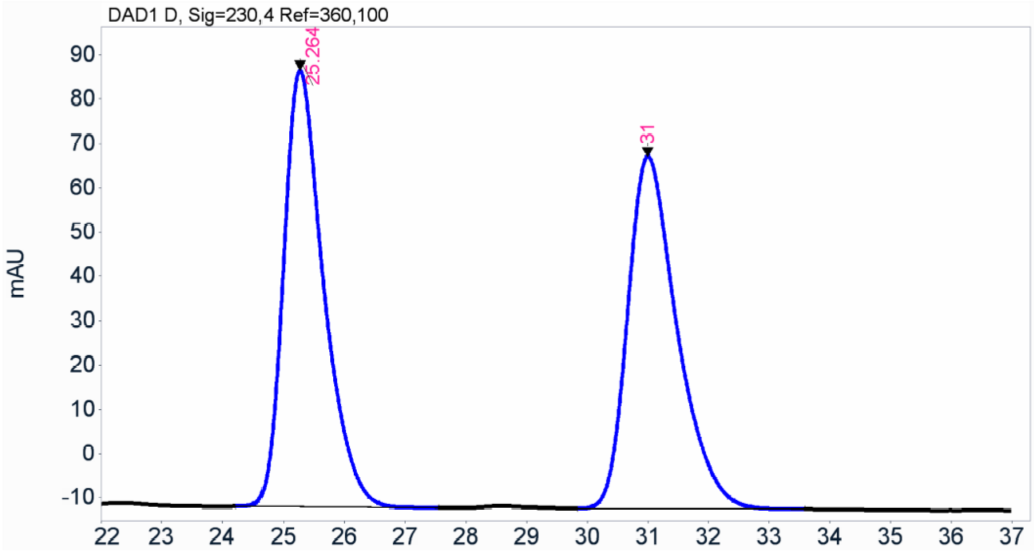

Signal: DAD1 D, Sig=230,4 Ref=360,100

RT [min]	Type	Width [min]	Area	Height	Area%
25.264	BB	0.6867	4498.959	98.5344	50.03
31.000	BB	0.8556	4493.862	79.4827	49.97

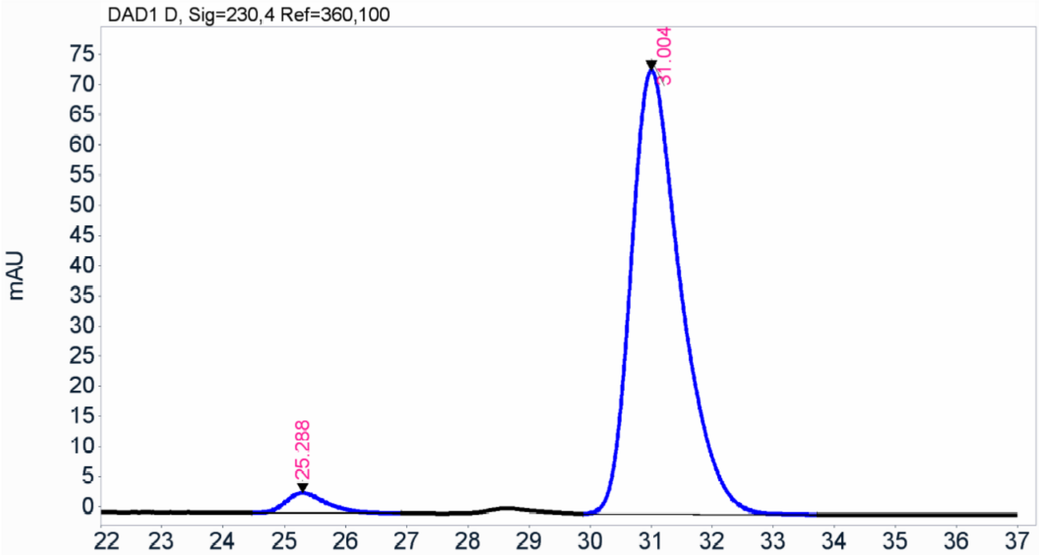

Signal: DAD1 D, Sig=230,4 Ref=360,100

RT [min]	Type	Width [min]	Area	Height	Area%
25.288	BB	0.6362	152.241	3.3497	3.53
31.004	BB	0.8496	4160.370	73.5858	96.47

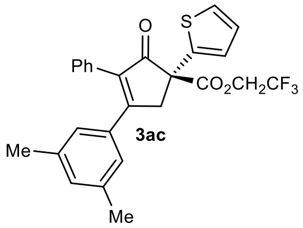

3ac

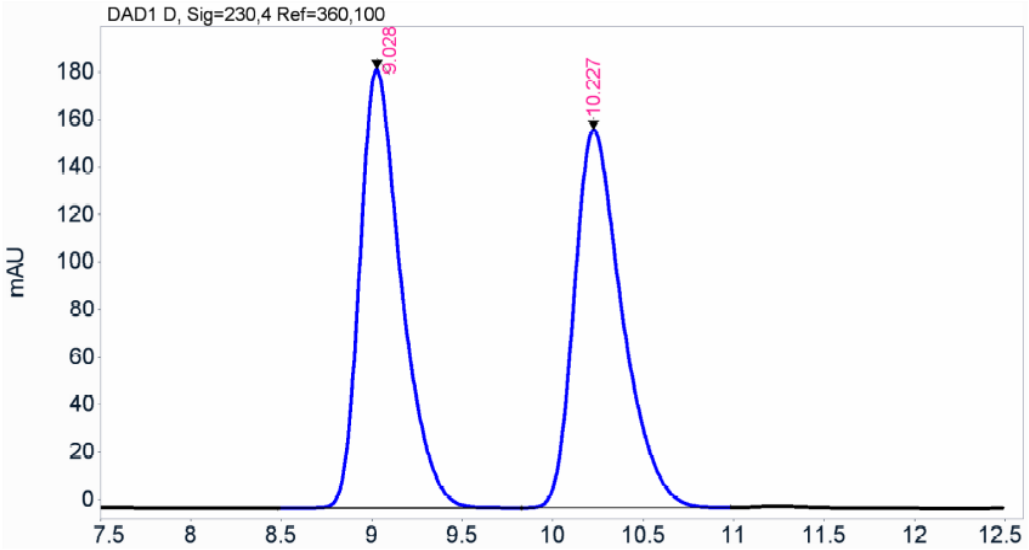

Signal:	DAD1 D, Sig=230,4 Ref=360,100				
RT [min]	Type	Width [min]	Area	Height	Area%
9.028	BB	0.2378	2936.557	184.3945	50.08
10.227	BB	0.2767	2926.594	159.0366	49.92

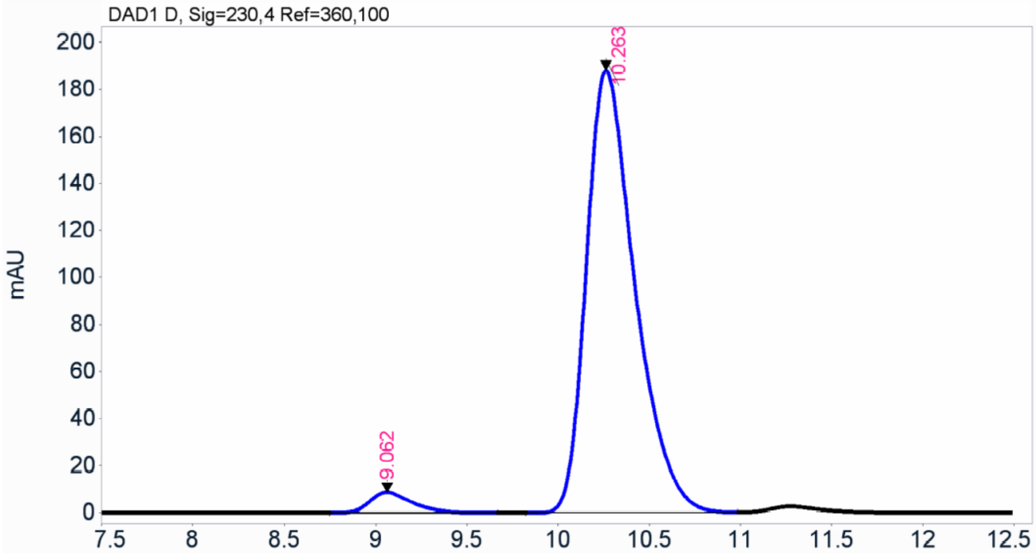

Signal:	DAD1 D, Sig=230,4 Ref=360,100				
RT [min]	Type	Width [min]	Area	Height	Area%
9.062	BB	0.2381	139.937	8.7729	3.87
10.263	BB	0.2773	3473.923	188.2115	96.13

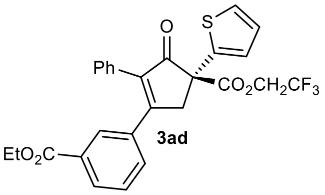

3ad

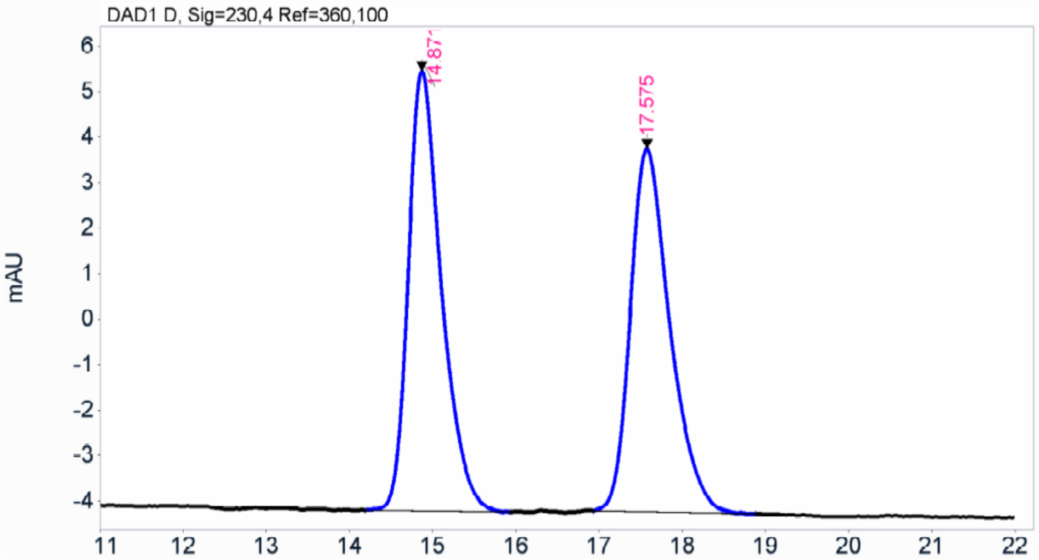

Signal:	DAD1 D, Sig=230,4 Ref=360,100				
RT [min]	Type	Width [min]	Area	Height	Area%
14.871	BB	0.4135	268.130	9.6700	50.51
17.575	BB	0.4941	262.668	7.9775	49.49

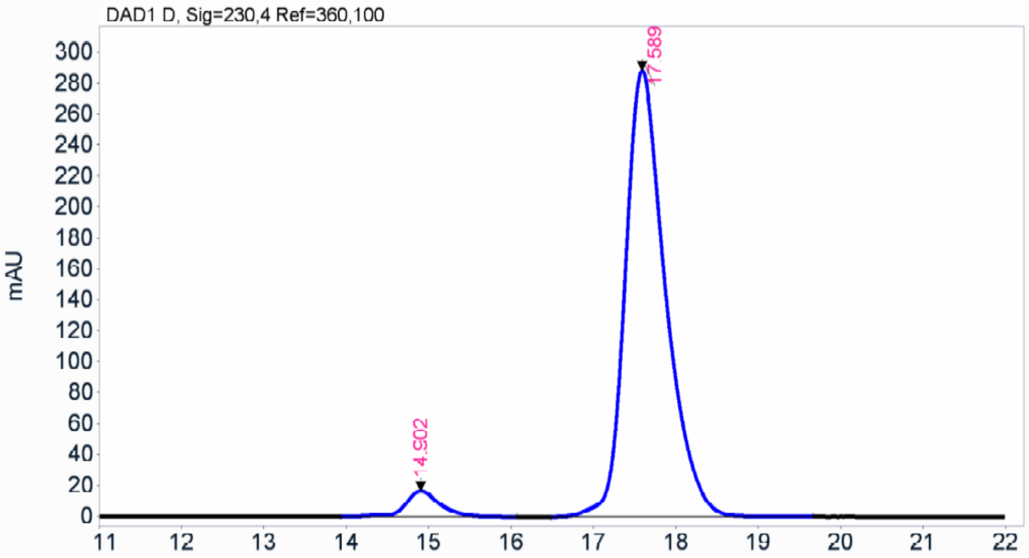

Signal:	DAD1 D, Sig=230,4 Ref=360,100				
RT [min]	Type	Width [min]	Area	Height	Area%
14.902	BB	0.4315	483.215	16.6065	4.70
17.589	BB	0.5105	9805.579	288.5377	95.30

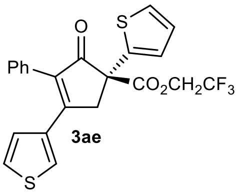

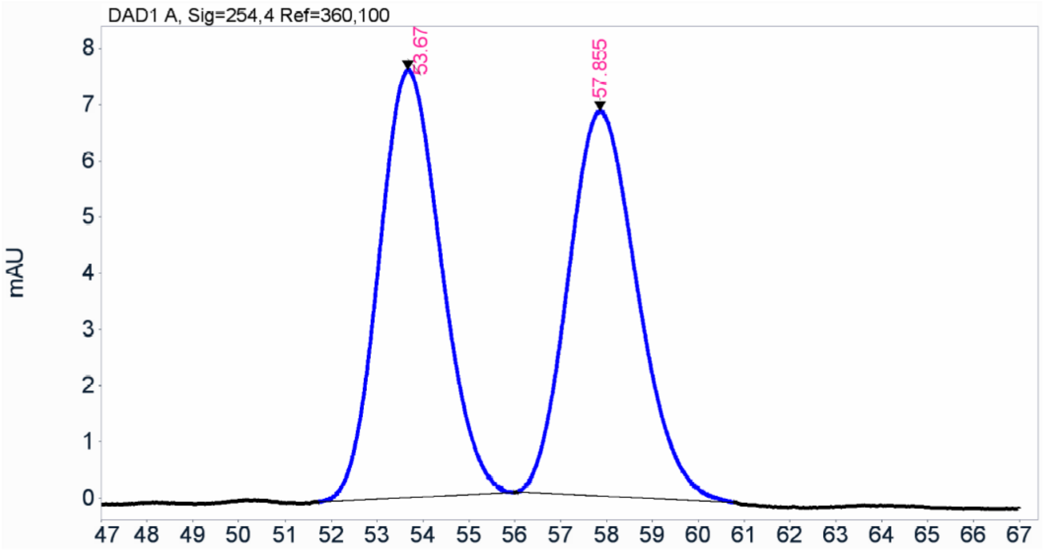

Signal: DAD1 A, Sig=254,4 Ref=360,100

RT [min]	Type	Width [min]	Area	Height	Area%
53.670	BB	1.1748	714.756	7.6029	49.99
57.855	BB	1.2341	715.048	6.8374	50.01

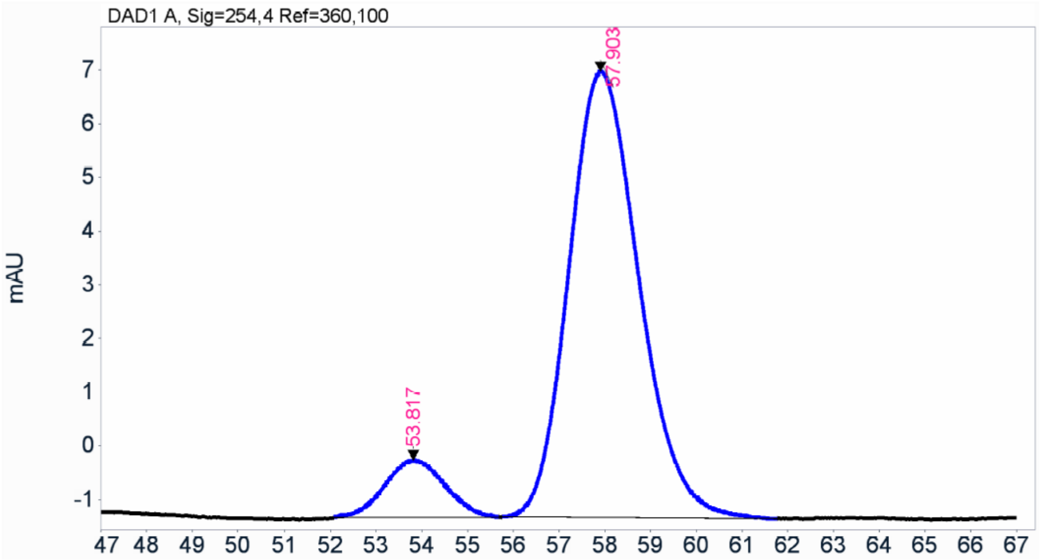

Signal: DAD1 A, Sig=254,4 Ref=360,100

RT [min]	Type	Width [min]	Area	Height	Area%
53.817	MM T	1.5348	97.912	1.0633	9.99
57.903	MM T	1.7723	882.301	8.2970	90.01

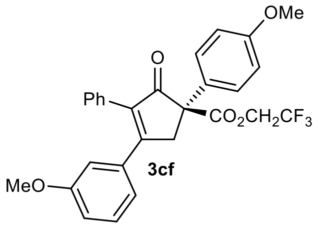

3cf

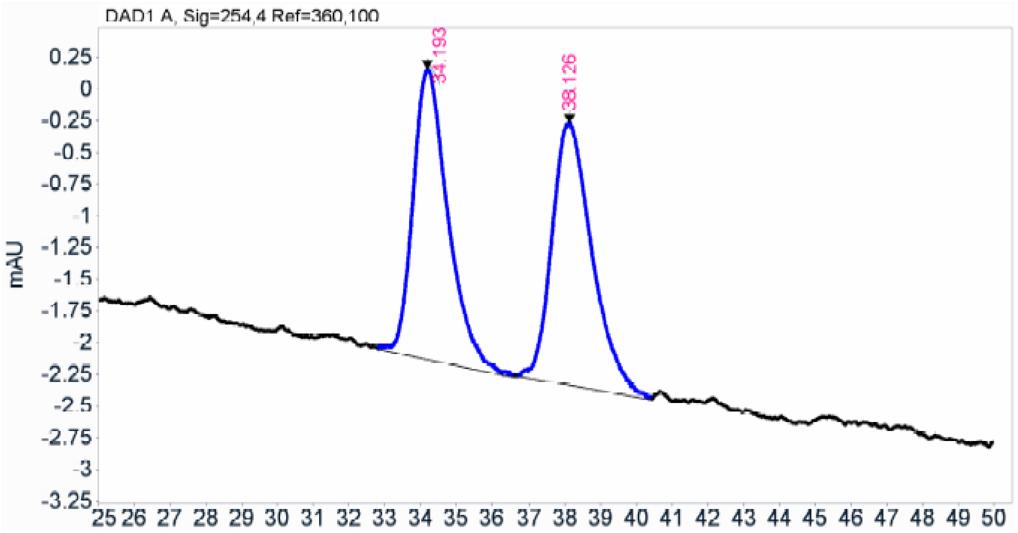

**Signal:**	DAD1 A, Sig=254,4 Ref=360,100				
**RT [min]**	**Type**	**Width [min]**	**Area**	**Height**	**Area%**
34.193	MM	1.1456	157.103	2.2857	49.25
38.126	MM	1.3063	161.881	2.0653	50.75

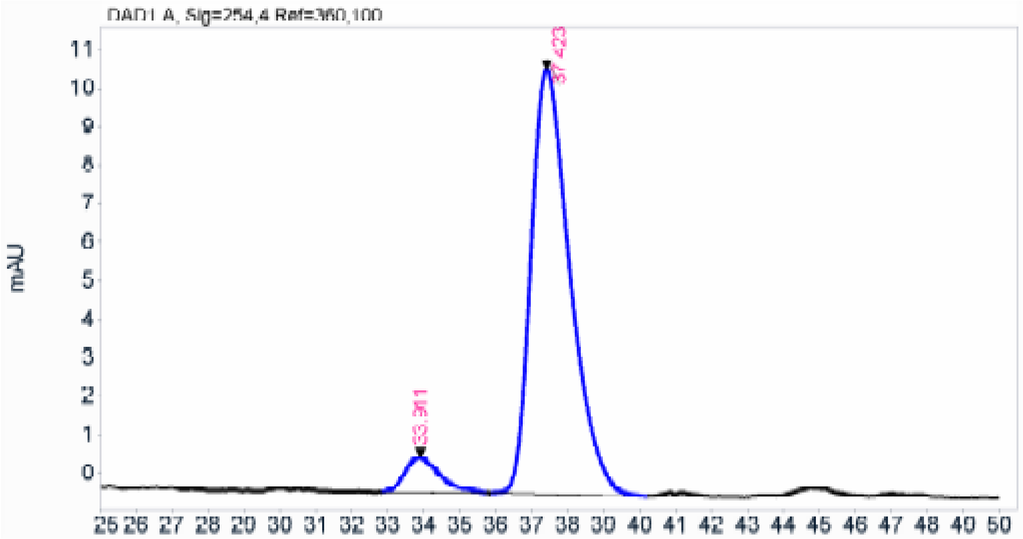

**Signal:**	DAD1 A, Sig=254,4 Ref=360,100				
**RT [min]**	**Type**	**Width [min]**	**Area**	**Height**	**Area%**
33.911	MM	1.1312	60.561	0.8922	6.79
37.423	MM	1.2565	830.942	11.0217	93.21

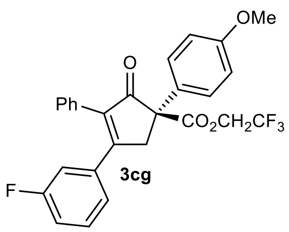

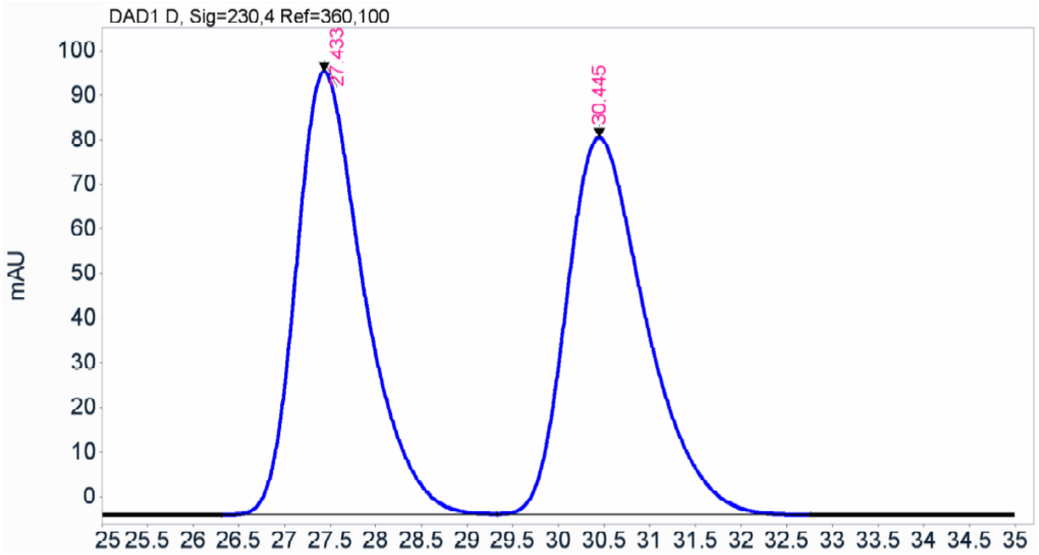

Signal:	DAD1 D, Sig=230,4 Ref=360,100				
RT [min]	Type	Width [min]	Area	Height	Area%
27.433	BB	0.7733	5085.316	99.3239	50.02
30.445	BB	0.9156	5080.803	84.4834	49.98

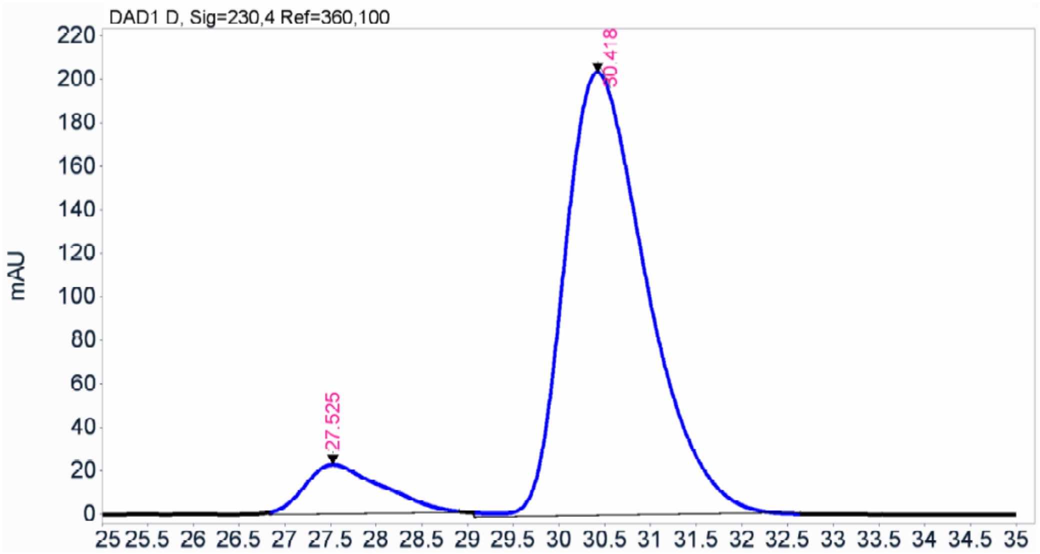

Signal:	DAD1 D, Sig=230,4 Ref=360,100				
RT [min]	Type	Width [min]	Area	Height	Area%
27.525	MM	0.9808	1328.451	22.5739	9.49
30.418	MM	1.0374	12671.773	203.5783	90.51

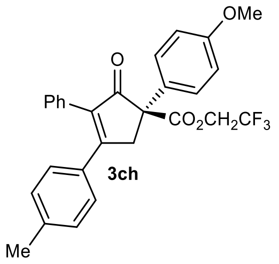

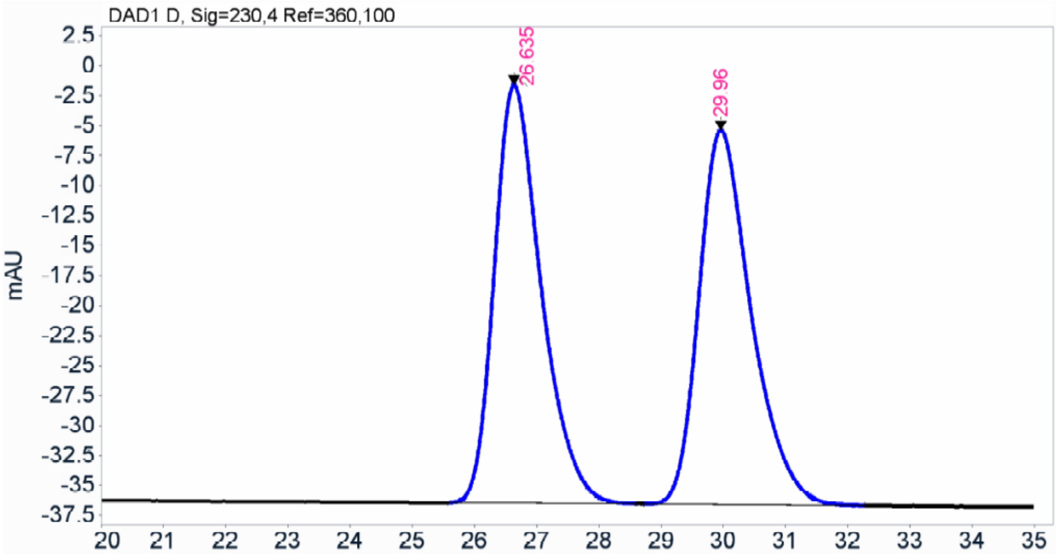

Signal:	DAD1 D, Sig=230,4 Ref=360,100				
RT [min]	Type	Width [min]	Area	Height	Area%
26.635	BB	0.7830	1823.942	34.8256	50.07
29.960	BB	0.8760	1818.661	31.2040	49.93

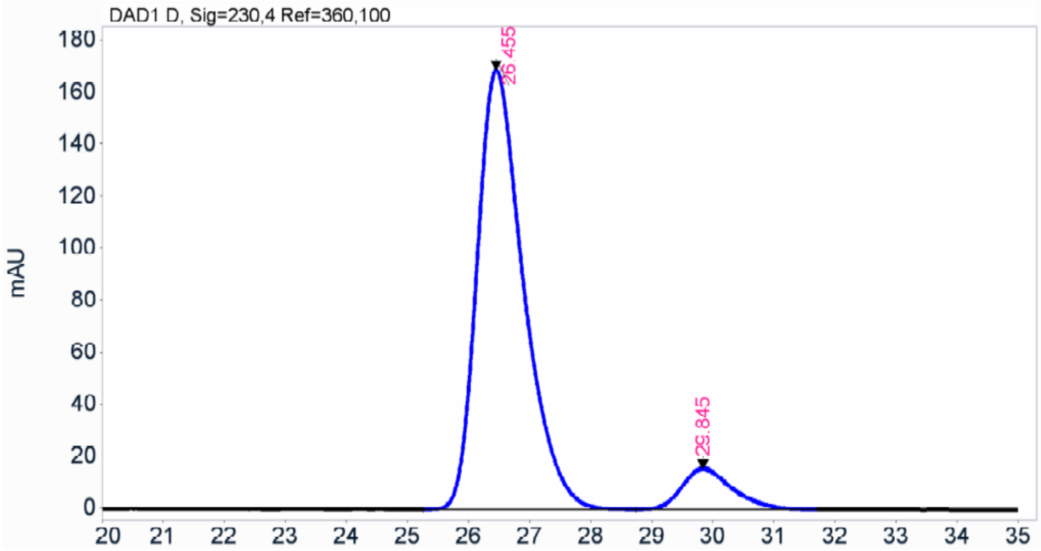

Signal:	DAD1 D, Sig=230,4 Ref=360,100				
RT [min]	Type	Width [min]	Area	Height	Area%
26.455	BB	0.7954	8905.395	168.8298	90.81
29.845	BB	0.8469	900.897	15.5737	9.19

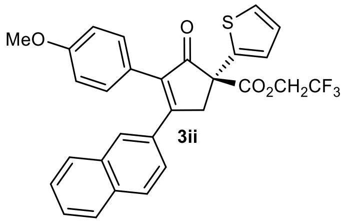

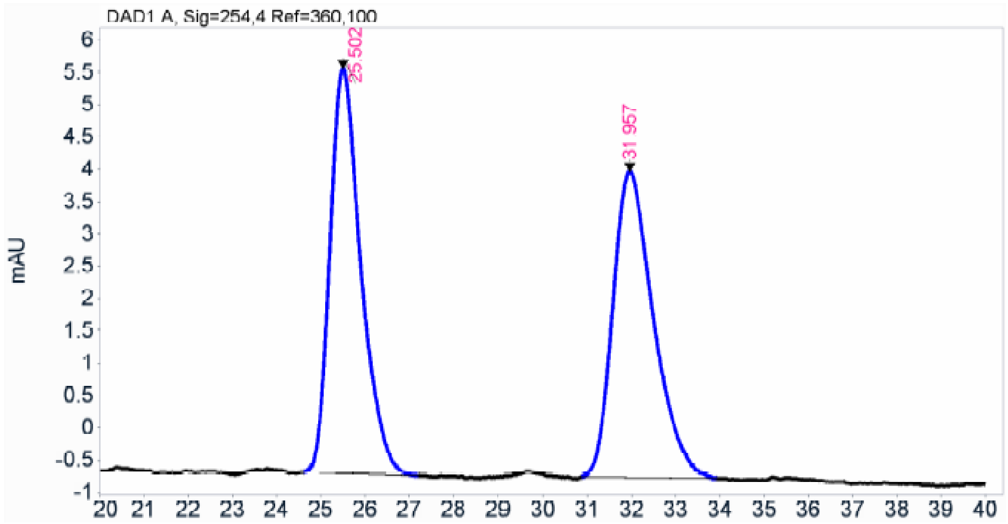

Signal: DAD1 A, Sig=254,4 Ref=360,100

RT [min]	Type	Width [min]	Area	Height	Area%
25.502	BB	0.7084	304.025	6.2623	50.16
31.957	BB	0.8403	302.121	4.7359	49.84

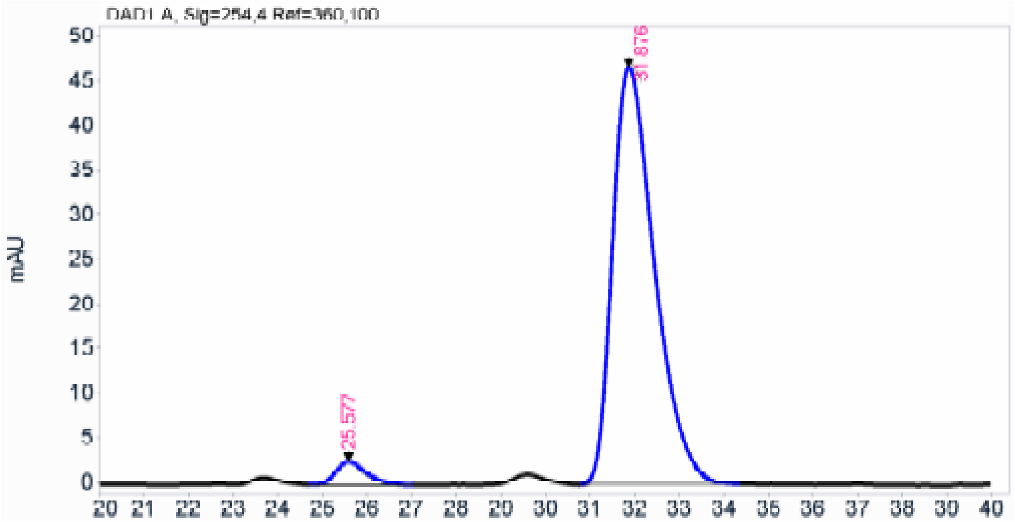

Signal: DAD1 A, Sig=254,4 Ref=360,100

RT [min]	Type	Width [min]	Area	Height	Area%
25.577	MM	0.8116	126.024	2.5880	3.99
31.876	BB	0.9853	3033.272	46.7268	96.01

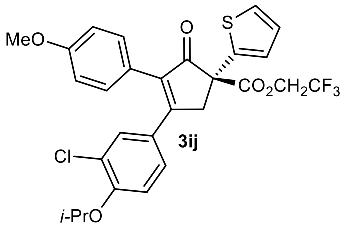

3ij

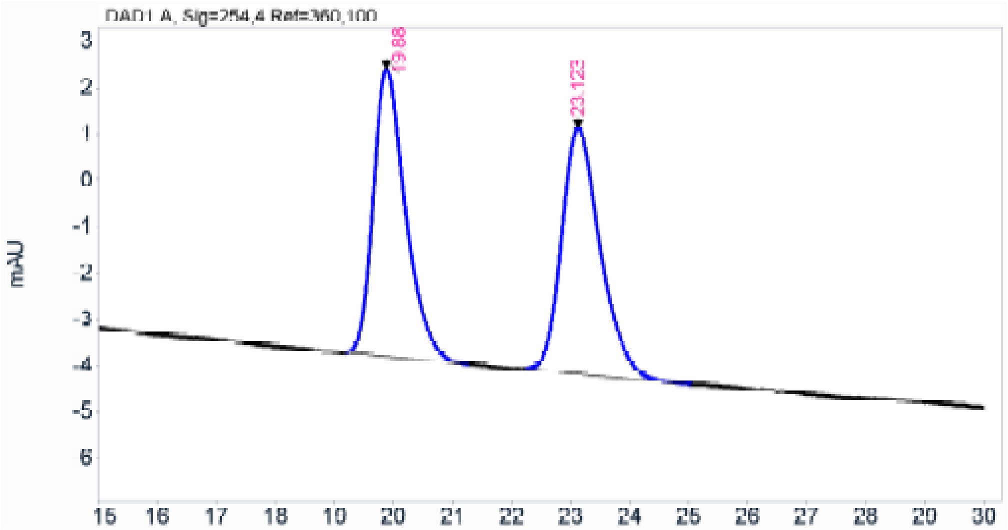

Signal:	DAD1 A, Sig=254,4 Ref=360,100				
RT [min]	Type	Width [min]	Area	Height	Area%
19.880	BB	0.5661	238.617	6.2129	49.77
23.123	BB	0.6643	240.866	5.2990	50.23

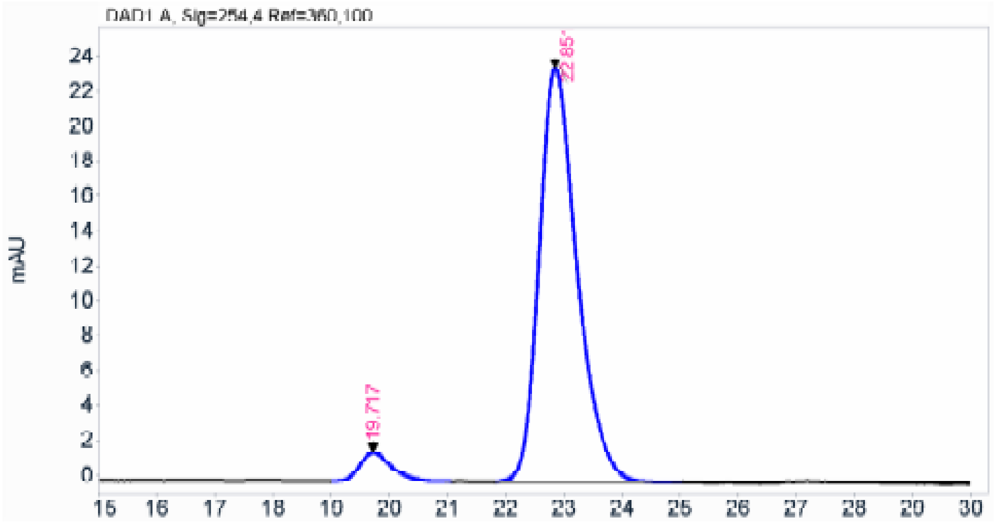

Signal:	DAD1 A, Sig=254,4 Ref=360,100				
RT [min]	Type	Width [min]	Area	Height	Area%
19.717	MM	0.6298	61.195	1.6195	5.39
22.851	MM	0.7550	1074.178	23.7127	94.61

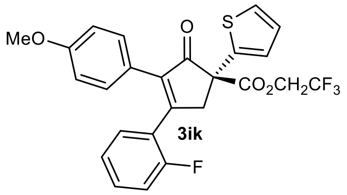

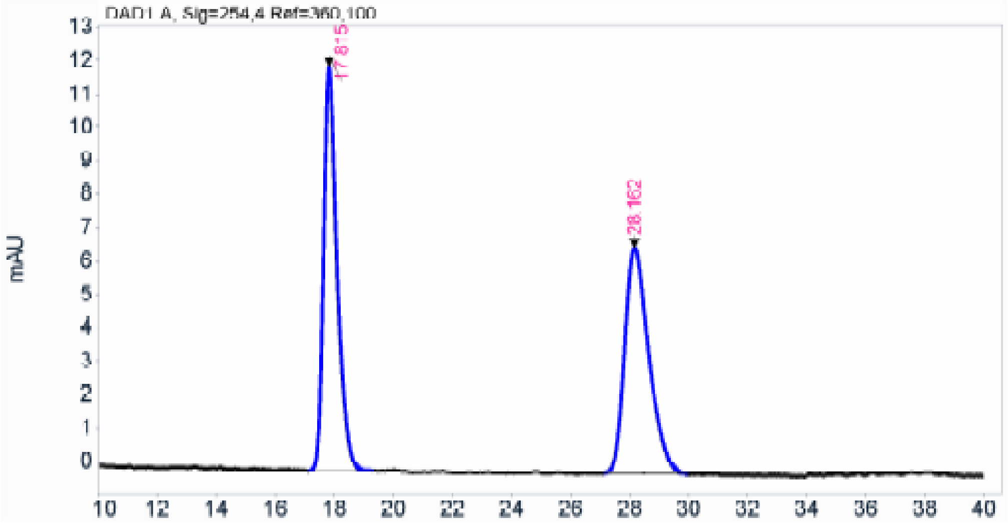

Signal: DAD1 A, Sig=254,4 Ref=360,100

RT [min]	Type	Width [min]	Area	Height	Area%
17.815	BB	0.4825	388.605	12.1055	50.45
28.162	BB	0.7849	381.643	6.7575	49.55

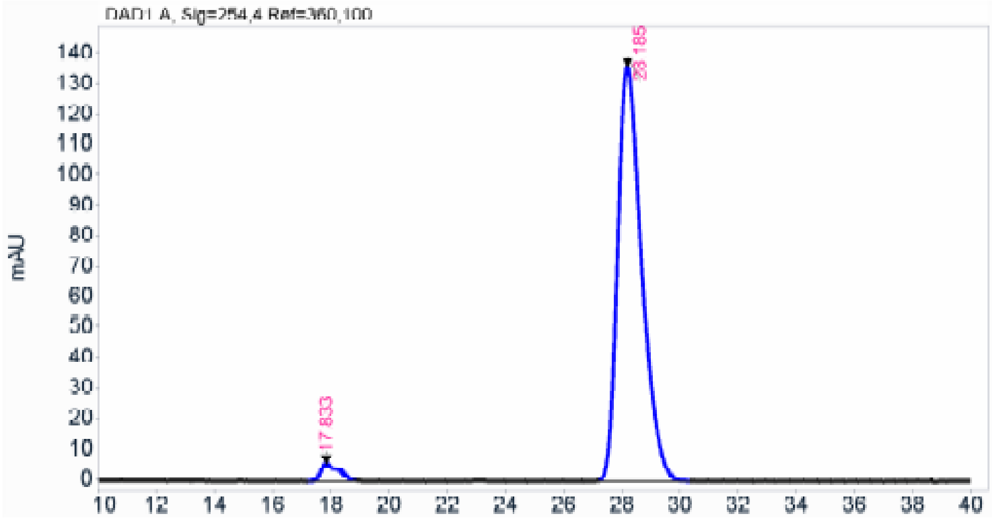

Signal: DAD1 A, Sig=254,4 Ref=360,100

RT [min]	Type	Width [min]	Area	Height	Area%
17.833	BV	0.6055	244.135	5.4621	3.03
28.185	VB	0.8153	7806.243	136.0712	96.97

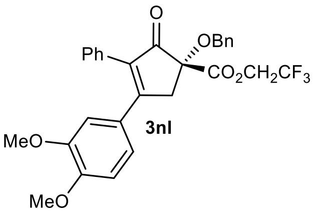

3nl

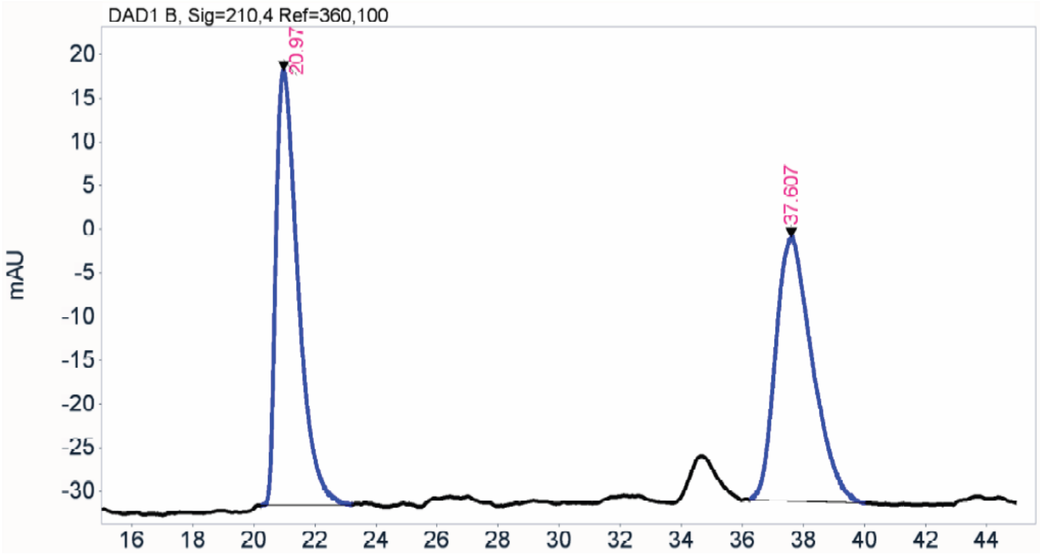

Signal:	DAD1 B, Sig=210,4 Ref=360,100				
RT [min]	Type	Width [min]	Area	Height	Area%
20.970	BB	0.7670	2502.328	49.7332	49.92
37.607	BB	1.0850	2510.119	30.0962	50.08

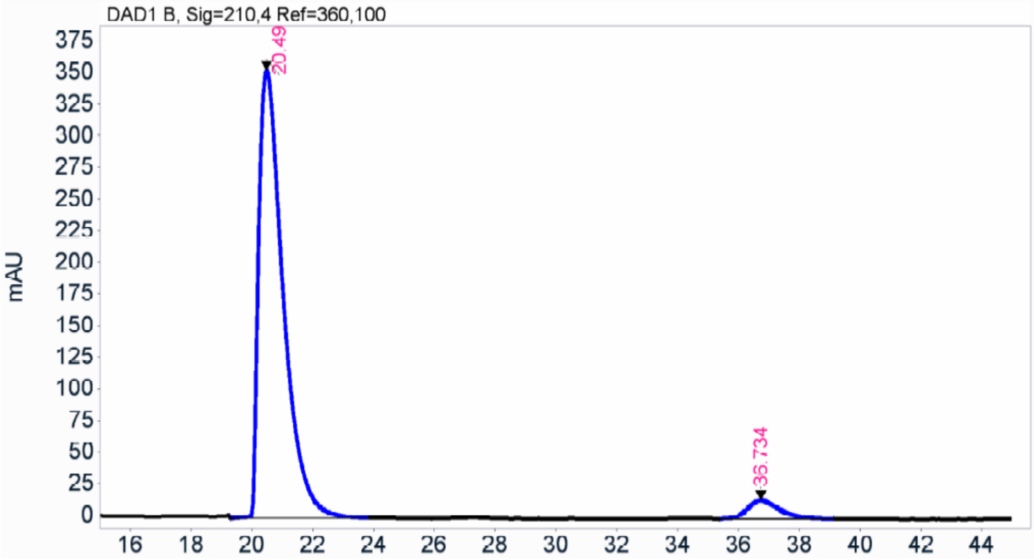

Signal:	DAD1 B, Sig=210,4 Ref=360,100				
RT [min]	Type	Width [min]	Area	Height	Area%
20.490	BB	0.8577	19755.350	353.6317	94.89
36.734	BB	0.9090	1063.474	14.7183	5.11

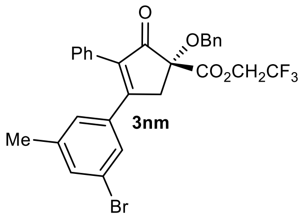

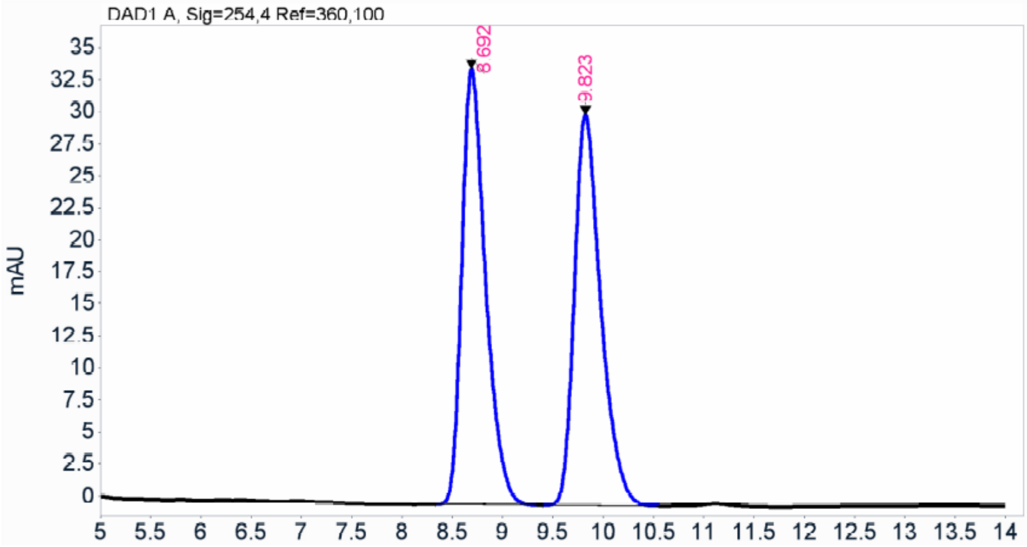

Signal: DAD1 A, Sig=254,4 Ref=360,100

RT [min]	Type	Width [min]	Area	Height	Area%
8.692	BB	0.2472	556.924	33.9875	49.98
9.823	BB	0.2755	557.460	30.4616	50.02

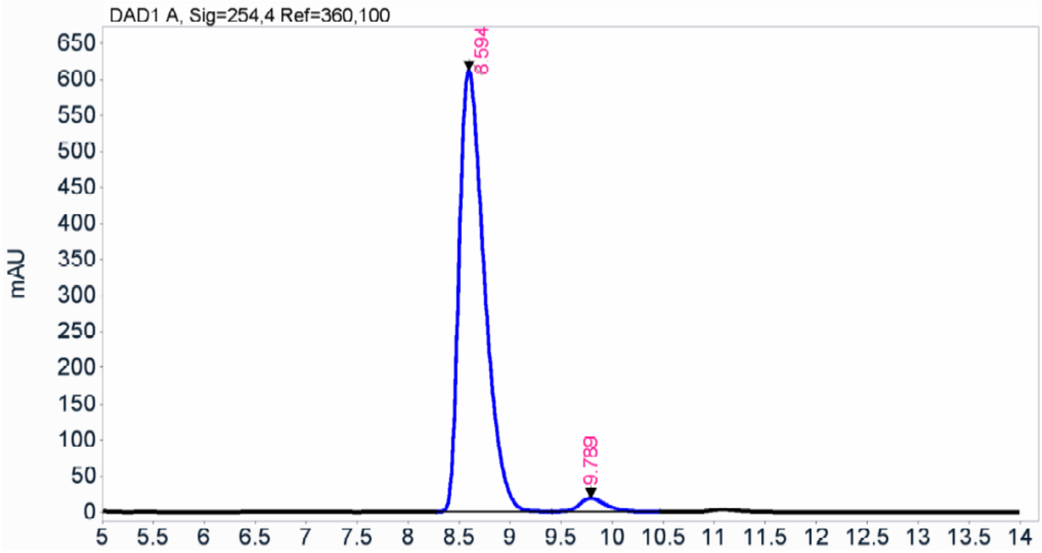

Signal: DAD1 A, Sig=254,4 Ref=360,100

RT [min]	Type	Width [min]	Area	Height	Area%
8.594	BB	0.2638	10483.550	612.0304	96.89
9.789	BB	0.2705	336.140	18.6321	3.11

8. References

1. Schrems, M. G.; Pfaltz, A. *Chem. Commun.* **2009**, 6210–6212.
2. de Nanteuil, F.; Loup, J.; Waser, J. *Org. Lett.* **2013**, *15*, 3738–3741.
3. Hennessy, E. J.; Buchwald, S. L. *Org. Lett.* **2002**, *4*, 269–272.
4. Yip, S. F.; Cheung, H. Y.; Zhou, Z.; Kwong, F. Y. *Org. Lett.* **2007**, *9*, 3469–3472.
5. Alkynyl bromides **S12** and **S15** were prepared according to: Oliva, A. I.; Christmann, U.; Font, D.; Cuevas, F.; Ballester, P.; Buschmann, H.; Torrens, A.; Yenes, S.; Pericas, M. A. *Org. Lett.* **2008**, *10*, 1617–1619.
6. Alkynyl bromides **S13** and **S14** were prepared according to: Parmar, D.; Matsubara, H.; Price, K.; Spain, M.; Procter, D. J. *J. Am. Chem. Soc.* **2012**, *134*, 12751–12757.
7. Alkynyl bromide **S16** was prepared according to: Okitsu, T.; Sato, K.; Potewar, T. M.; Wada, A. *J. Org. Chem.* **2011**, *76*, 3438−3449.
8. Beare, N. A.; Hartwig J. F. *J. Org. Chem.* **2002**, *67*, 541–555.
9. Boyd, S.; Davies, C. D. *Tetrahedron Lett.* **2014**, *55*, 4117–4119.
10. 5-Methylhex-4-en-2-yn-1-ol was prepared according to: B. M. Trost and R. C. Livingston, *J. Am. Chem. Soc.* **2008**, *130*, 11970–11978.
11. Best, D.; Jean, M.; van de Weghe, P. *J. Org. Chem.* **2016**, *81*, 7760–7770.
12. 2-(*m*-Tolyl)malonic acid was prepared according to: (a) Puente, Á.; He, S.; Corral-Bautista, F.; Ofial, A. R.; Mayr, H. *Eur. J. Org. Chem.* **2016**, 1841–1848. (b) Hayashi, K; *Chem. Pharm. Bull.* **1959**, *7*, 912–916. (c) Pai, G.; Chattopadhyay, A. P. *Synthesis*, **2013**, *45*, 1475–1482.
13. 4-Methyl-*N*-(3-phenylprop-2-yn-1-yl)benzenesulfonamide was prepared according to C. Chen, Y. Huang, Z. Zhang, X.-Q. Dong, X. Zhang, *Chem. Commun.* **2017**, *53*, 4612–4615.
